# Seed protein biotyping in *Amaranthus* species: a tool for rapid identification of weedy amaranths of concern

**DOI:** 10.1186/s13007-023-01116-9

**Published:** 2023-12-11

**Authors:** Maxime Murphy, Julia Hubert, Ruojing Wang, Leonardo Galindo-González

**Affiliations:** 1 Ottawa Plant Laboratory, Canadian Food Inspection Agency, 3851 Fallowfield Road, Ottawa, ON K2J 4S1 Canada; 2 Saskatoon Laboratory, Canadian Food Inspection Agency, 421 Downey Road, Saskatoon, SK S7N 4L8 Canada

**Keywords:** *Amaranthus*, *Amaranthus palmeri*, *Amaranthus tuberculatus*, Protein biotyping, MALDI, Seed identification

## Abstract

**Background:**

The *Amaranthus* genus contains at least 20 weedy and invasive species, including *Amaranthus palmeri* (palmer’s amaranth) and *Amaranthus tuberculatus* (tall waterhemp), two species of regulatory concern in North America, impacting production and yield in crops like corn, soybean and cotton. *Amaranthus tuberculatus* is regulated in Canada with limited establishment, while current climate models predict a range expansion of *A. palmeri* impacting crop growing areas in Ontario, Quebec and Manitoba. Since many *Amaranthus* species are similar in their morphology, especially at the seed stage, this demands the development of additional methods that can efficiently aid in the detection and identification of these species. Protein biotyping using Matrix-Assisted Laser Desorption Ionization Time of Flight Mass Spectrometry (MALDI-TOF-MS) has been traditionally used to identify microorganism species, races and pathotypes. Major protein fractions extracted from an organism, ionized and run through a biotyper using mass spectrometry, result in protein spectra that represent a fingerprint at the species or lower taxonomic rank, providing an efficient molecular diagnostics method. Here we use a modified protein biotyping protocol to extract major protein fractions from seeds of the family Brassicaceae to test our protocol, and then implemented the standardized approach in seeds from *Amaranthus* species. We then created a database of *Amaranthus* protein spectra that can be used to test blind samples for a quick identification of species of concern.

**Results:**

We generated a protein spectra database with 16 *Amaranthus* species and several accessions per species, spanning target species of regulatory concern and species which are phylogenetically related or easily confused at the seed stage due to phenotypic plasticity. Testing of two *Amaranthus* blind sample seed sets against this database showed accuracies of 100% and 87%, respectively.

**Conclusions:**

Our method is highly efficient in identifying *Amaranthus* species of regulatory concern. The mismatches between our protein biotyping approach and phenotypic identification of seeds are due to absence of the species in the database or close phylogenetic relationship between the species. While *A. palmeri* cannot be distinguished from *A. watsonii*, there is evidence these two species have the same native range and are closely related.

**Supplementary Information:**

The online version contains supplementary material available at 10.1186/s13007-023-01116-9.

## Background

Identification of plant species is an important part of biodiversity studies and the regulatory framework in many countries. Federally regulated species and invasive species pose a risk to the agricultural production and natural habitats or ecosystems, can affect human health and cause significant economic loss in trade. In Canada regulation of invasive plant species or noxious weeds is done under the authority of the Plant Protection Act [1], Seeds Act [2] and Feeds Act [3]. Identification of seeds or plants can be performed using morphological characterization by expert botanists. However, while expert analysts can identify species from seeds or plants, the plasticity and similarity of weedy species could make the identification very challenging. In these cases, molecular tools can aid in classifying the specimens.

The *Amaranthus* genus contains over 70 species, including species cultivated for their grain or edible leaves, but also highly weedy and invasive species [4] that impact yield and production of major crops in North America including soybean, corn and cotton [5–8]. Losses associated with *A. palmeri* for 2015 were estimated to be 250 million USD for cotton, 1.3 billion USD for maize and 2.5 billion USD for soybean [8]. A 91% yield reduction was calculated for maize in Kansas when the density of *A. palmeri* was 10.5 plants per square meter [9]. And yield losses for soybean reached 78.7%, 56.2% and 38% at a density of 8 plants per square meter for palmer amaranth, tall waterhemp and redroot pigweed (*A. retroflexus*), respectively [10].

A major concern of weedy species like *A. tuberculatus* and *A. palmeri* is their increased resistance to multiple herbicide modes of action [5, 8, 11, 12], resulting in limiting the number of strategies for control. Additionally, trade can be jeopardized when contaminating seed is found in commodity shipments. For example, *A. palmeri* glyphosate-resistant populations have been established in less than 10 years in Japan from contaminated grain seed imported from the United States [13].

While not federally regulated in the US, *A. palmeri* is regulated as a noxious weed in Delaware, Iowa, Minnesota, North Dakota, Ohio, and Pennsylvania [14], and it is present in 32 states [8]. Canada regulates *A. tuberculatus*, but not *A. palmeri*, for which risk assessment is ongoing. However, while no established populations have been yet found in Canada, *A. palmeri* has been reported in Ontario and Manitoba. Viable seeds have been reported as part of import shipments [15]. Furthermore, an import ban from China that started in 2019, on two of the largest Canadian-based canola exporters (Viterra and Richardson) was based on seed contamination with quarantine seeds and pathogens, among which palmer amaranth was allegedly present [8]. Since China buys 40% of the Canadian canola exports, this has resulted in losses of over 2 billion dollars for the Canadian economy.

Climate change modeling and the strong phenotypic plasticity of *A. palmeri* [4], shows that the most likely scenario in the next few years is a constant expanding range into Canada for this species, affecting growing regions of corn and soybean in Ontario, Quebec and Manitoba [4, 6, 16, 17]. These factors demand effective diagnostic tools to distinguish species of concern from related species to avoid negative impacts on the environment and the economy.

Molecular identification of plant species has traditionally been done using DNA barcodes, which entail short DNA regions (e.g., 300 – 800 bp) that work as a fingerprint bearing specific polymorphisms that are unique to the species in question. Many barcodes have been tested for plants as potential regions for identification of most taxa, including several chloroplast barcodes (*trnH-psbA, rbcL, matK*) and the internal transcribed spacers from ribosomal DNA (*ITS1* and *ITS2*) [18–24]. However, these regions can fail to provide resolution for specific taxa as when distinguishing regulated species from their non-regulated counterparts. One solution to this issue it to continue exploring taxa-specific genomic regions with strong polymorphic signals. Usually this can be done by sequencing full chloroplast genomes or full nuclear rDNA regions that constitute reservoirs of diversity where new DNA barcodes can be found. The exploration of these larger regions for new sources of polymorphism has been facilitated by approaches like genome skimming [25–29], a technique that uses low-pass sequencing of a full genome to achieve a high read number covering the highly repeated fractions of the genome (e.g., chloroplast and nrDNA). Alternatively, specific nuclear genes/regions used in phylogenetic studies [30–35], can also be explored as sources of polymorphic markers when nrDNA or chloroplast DNA do not suffice to provide polymorphic signal among the studied species. Finally, the increased use of long-read sequencing technology (Nanopore and PacBio) has open new doors not only to mine genomes for new polymorphic regions, but to generate DNA barcodes in the kilobase range [36, 37], thus allowing to compile multiple barcodes in a single read and test thousands of samples concurrently.

In *Amaranthus* the study of traditional barcoding regions and other genomic regions, has resulted in the development of species-specific barcodes and PCR-based assays, allowing identification of some species in the genus [12, 38, 39]. While these methodologies are effective in detecting some of the species, the process of finding sequences for DNA barcoding can be long, and PCR based analysis require multiple primers and sometimes specific conditions to detect specific species.

Among alternative methodologies to DNA barcoding and PCR-based assays for species identification is protein biotyping. The technique is based on a simple protocol for rapid extraction of the major acid soluble protein fractions of the biological samples (including ribosomal proteins), followed by immobilization on a solid matrix and ionization of the proteins followed by mass spectrometry using Matrix-Assisted Laser Desorption Ionization or MALDI [40]. The result of this process is a protein mass spectra per each tested sample, providing a fingerprint for the species. Protein biotyping has been used often in the identification of microorganisms, especially in pathogen identification [40–44], and also specifically in plant pathogen identification [45–49]. Recently, some research has been developed to test protein biotyping in plants. Importantly, work was performed to distinguish *Impatiens glandulifera* from other species in the genus, and also to distinguish its regional biotypes using leaves and seeds [50–52]. Using MALDI-TOF MS spectra from acid-soluble proteins, four different *I. glandulifera* biotypes differing in susceptibility to a rust biological control agent, were identified [52]. Extraction of proteins from seed material [51, 52], seemed to contain a more stable protein fraction compared to tissue undergoing development (e.g., young leaves) [50]. Further work studied protein biotyping of tomato varieties, showing good reproducibility for the technique, but low accuracy in distinguishing the test varieties [53].

In the current study we tested protein biotyping on seeds from the family Brassicaceae from different samples and years, to demonstrate consistency on the generation of protein spectra from single seeds. Then we applied the protocol to seeds from *Amaranthus* species, which comprise species of regulatory concern, including the two species of highest concern in Canada due to their weediness and invasiveness (*A. palmeri* and *A. tuberculatus*). Our results showed high accuracy in identifying two sets of 15 and 60 blind sample batches, using a database of 16 *Amaranthus* species. Protein biotyping using MALDI-TOF-MS is promising as a suitable, cheap and efficient technique to identify *Amaranthus* species of regulatory concern. The method has the potential to be easily transferred to other taxa to aid in identification of species that impact trade and weed management.

## Results

### Brassicaceae preliminary tests

To test our protein biotyping methodology we set out to complete both protein extractions and biotyping using seeds from four species of the family Brassicaceae. These seeds were used because they were readily available and in large amounts at the National Archive of Legal Reference Material at the CFIA Seed Science Unit. This allowed for multiple testing to standardize conditions before testing *Amaranthus* spp. where material was initially more scarce.

**Fig. 1:**
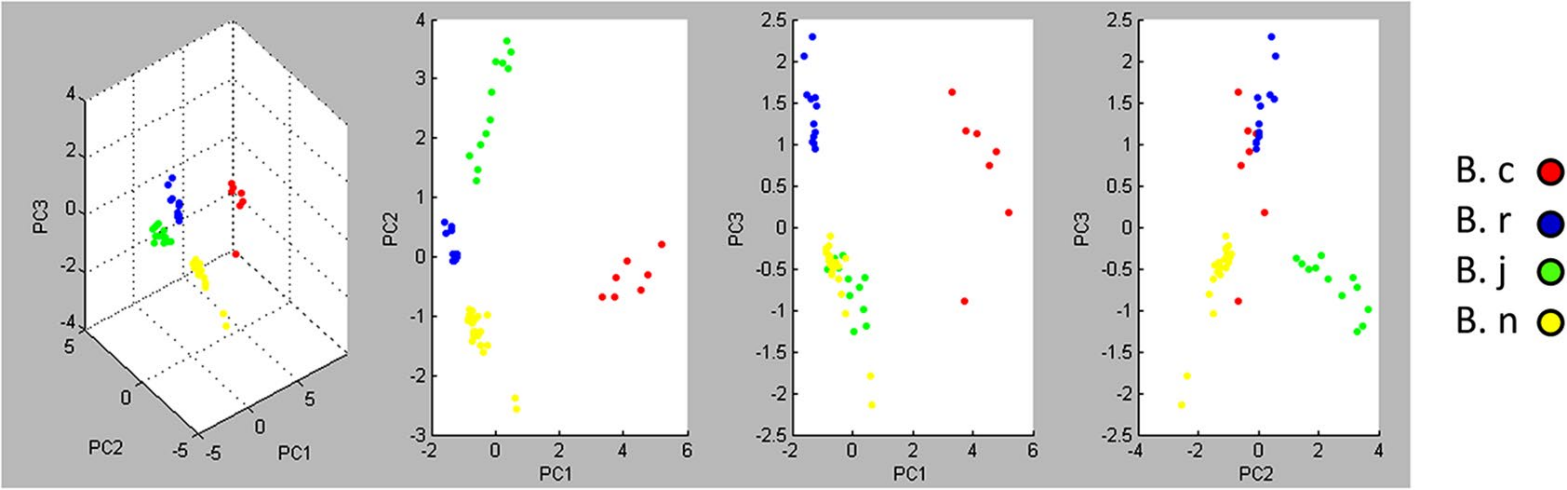
Principal Component Analysis (PCA) of protein spectra corresponding to four *Brassica* species. B. c (*Brassica carinata*), B. r (*Brassica rapa*), B. j (*Brassica juncea*), B. n (*Brassica napus*)

We performed a preliminary test with small modifications to previously established protocols (see materials and methods) on five seed batches corresponding to two varieties of *Brassica napus* (spring and winter), and one variety of each of other three species (*B. rapa, B. juncea* and *B. carinata*). Three seeds of each batch, with two different solvent dilutions (1:1 and 1:10 - see methods) and two technical replicates were obtained. Out of the 12 potential protein spectral profiles expected for each seed batch, we obtained 12 spectra for *B. napus* (spring), 11 for *B. napus* (winter), 12 for *B. rapa*, 7 for *B. carinata* and 12 for *B. juncea.* Both a composite Principal Component Analysis (PCA) (Fig. 1), and a dendrogram analysis (Additional file 1 A), showed that all seeds belonging to the same species had similar protein spectra and clustered together. There was no specific clustering linked to dilution or technical replicates.

To test potential variability among seed batches from the same species but from different years we conducted a second run where we compared seed batches from three different years in *B*. *napus* (spring) and *B. rapa* along with the available single-year batches of *B. napus (*winter), *B. juncea* and *B. carinata.* To increase the probability of obtaining successful protein spectra we improved the protocol by using a more reliable approach to grind the seeds (tissue lyzer), and modified the preliminary protocol so that spotting of the samples in the target plate would require less manipulation (compare preliminary test with final protocol in materials and methods). Out of 45 total seeds (5 per accession) we obtained protein spectra for 43, with only two seeds failing from *B. carinata.* Samples clustered correctly for each one of the species (Additional File 1B). While our pilot study did not show that the age of the seeds constitutes a factor that could introduce variability into the protein spectral profiles, conditions of storage of seeds and the access to moisture could trigger oxidative processes on different biomolecules including proteins [54]. Therefore, we recommend having this in mind when testing the technology

### *Amaranthus* species protein biotyping

**Fig. 2:**
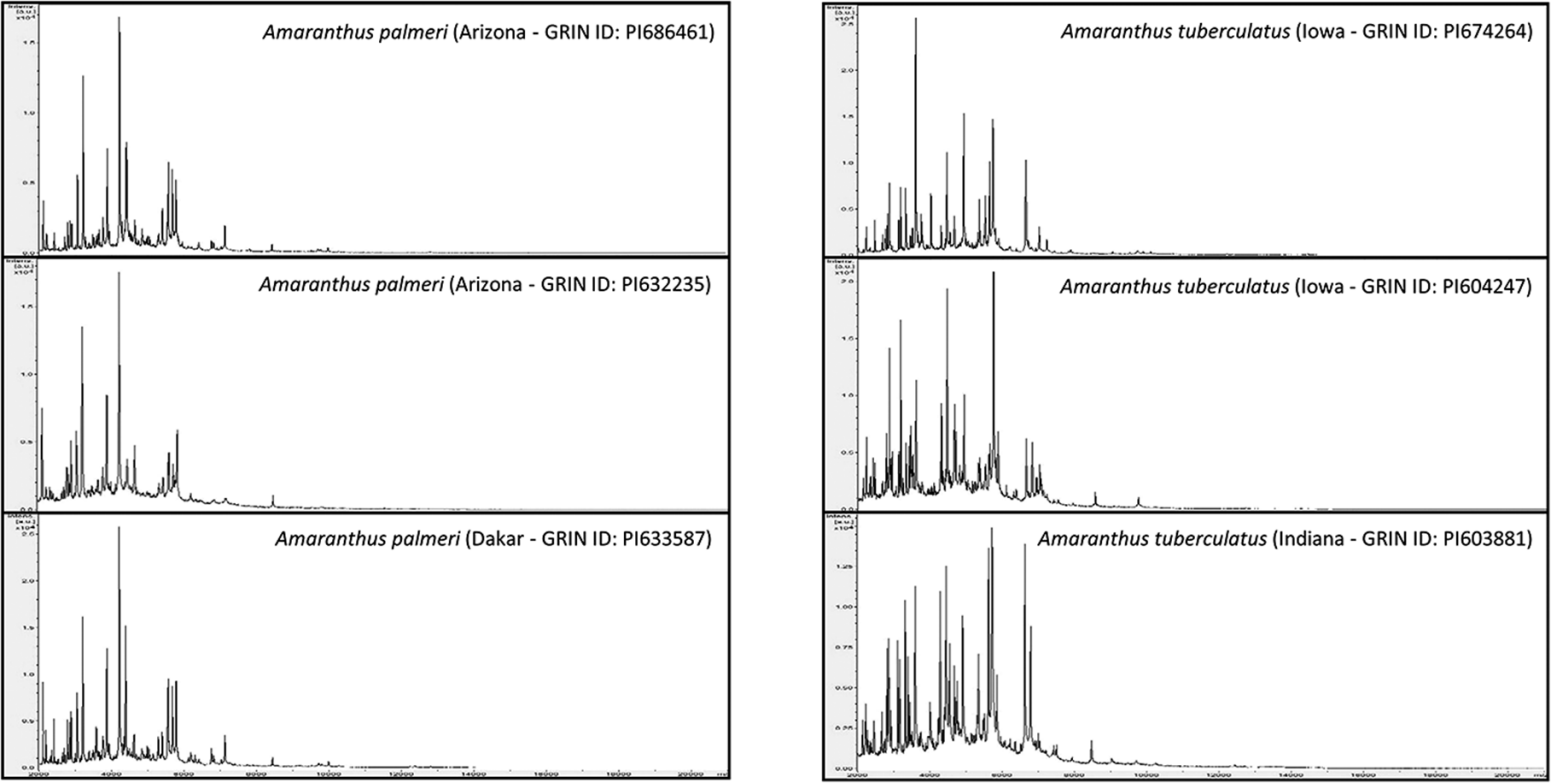
Protein spectra of two *Amaranthus* species. Three different *A. palmeri* accessions and three different *A. tuberculatus* accessions are shown. GRIN ID refers to their identification number from: https://npgsweb.ars-grin.gov/gringlobal/search

Protein biotyping is based on differences and similarities of protein spectra of major protein fractions of the different species. Figure 2 shows the difference and similarities between protein spectra generated from different accessions of *A. palmeri* and *A. tuberculatus* (two species of regulatory concern).

**Fig. 3:**
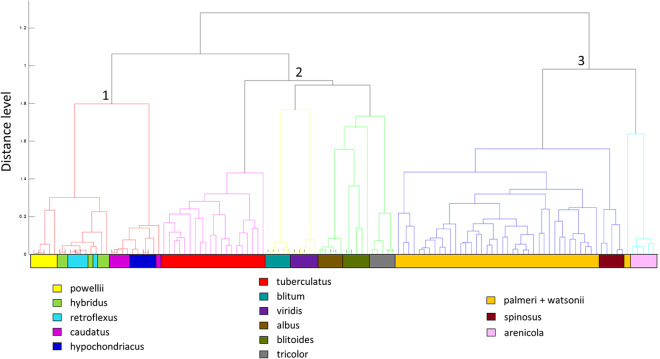
Clustering of protein spectra corresponding to 15 *Amaranthus* species. Species were clustered into three major clusters, with sub-clusters within each cluster

We performed protein biotyping with 15 *Amaranthus* species using five biological replicates (5 seeds) per accession. For some species like *A. tuberculatus* and *A. palmeri* we tested more than one accession due to regulatory concern of these two species in Canada or the United States. The protein spectra were clustered in three major groups with sub-clusters within the clusters (Fig. 3). Cluster 1 had two sub-clusters grouping the protein profiles corresponding to *A. powellii - A.hybridus - A. retroflexus*, and *A.hypochondriacus* - *A. caudatus*, respectively. However, the subgroups from this first cluster could not be clearly delimited by species. Cluster 2 had six species which could all be separated by their own sub-cluster. Importantly, a regulated species in Canada (*A. tuberculatus*) was separated from all other species on this cluster. In Cluster 3 we found *A. palmeri, A. watsonii, A. spinosus* and *A. arenicola.* While *A. spinosus* samples can be distinguished in a subgroup, the spectra from the two phylogenetically sister species (*A. palmeri* and *A. watsonii*) cannot be distinguished. A single spectra from *A. palmeri* was clustered with *A. arenicola.*

### Database generation

While spectra clustering is a rapid way of visualizing relationships between spectra of individual samples, PCA and dendrograms are not based on structured phylogenetic algorithms. Protein biotyping is meant to be used to characterize samples for which a phenotypic identification is not possible, to confirm phenotypes and to classify blind samples, using a spectral database of known samples to which unknown samples can be compared to. Enrichment of this database with multiple accessions per species (e.g., coming from different geographical regions), provides a way of accounting for potential intra-species variability and increases accuracy of determination of test samples. Furthermore, even when a dendrogram or PCA analysis shows different species clustering together, a rich database comprising several accessions of each of the species to be identified, will increase accuracy in sample identification [55, 56].

We generated a database with 16 *Amaranthus* species (seeds obtained from the Germplasm Resource Information Centre, U.S. National Plant Germplasm System – see methods), including species of regulatory concern, weedy species, and species that show phenotypic plasticity at the seed level that may be easily confused when performing phenotypic characterization (Additional file 2). To increase accuracy and resolution power of our database we included at least three different accessions per species when available, and used three seeds per accession (each one with 30 spectral readings – see methods), to account for biological and technical variation. We also had a larger number of accessions for species which pose the largest regulatory concern in Canada (*A. tuberculatus* and *A. palmeri*).

We generated spectral information for each seed, and produced a consensus Main Spectra (MSP) from at least 20 spectra per sample (Additional file 2). Each newly produced MSP was compared to the full database of MSPs to test if each MSP matched itself as the highest hit, and if the secondary hit (second highest similarity) also corresponded to another accession of the same species. This analysis showed that when the generated MSPs are used as unknown samples, they match themselves as top hits and match other accessions of the same species as secondary hits (Additional File 3). There were only four exceptions where the second best hit was not the expected species: *A. watsonii* (PI633593-1) second hit was *A. spinosus*, *A. palmeri* (PI667167-2) second hit was *A. spinosus*, *A. watsonii* (PI633593-RE2) second hit was *A. palmeri*, and A. *caudatus* (PI553073-1) second hit = was *A. hybridus*. This phenomena could be attributed to similarity in the protein spectra among species which are closely related phylogenetically. For these four exceptions, the second hits are phylogenetically related [7] to the first hits in all cases.

### Blind sample testing

We received blind samples from three different labs doing work in *Amaranthus* spp. Six blind samples were received from AAFC Saint-Jean-sur-Richelieu, 9 blind samples from AAFC Harrow and 60 blind samples from CFIA’s Seed Science and Technology Sections in Saskatoon. From the 6 samples received from Saint-Jean-sur-Richelieu, using 3 seeds per accession, we obtained 100% correct identification with matching average scores > 2 in most cases (Table 1, samples MIRL22-A.unknown-07 to 12). In one case a batch that was identified as *A. tuberculatus* (MIRL22-A.unknown-09) had one out of three seeds identified as *A. arenicola.* However, since two out of the three seeds for the respective accession were correctly identified, the final assigned identification matched the original identification uncovered by the providers after the protein biotyping analysis was completed. A similar situation happened with sample MIRL22.A.unknown-11, which had one of three seeds identified as *A. rudis* instead of *A. tuberculatus.* While historically these two were at times identified as different species, the latest consensus is that both are the same species, or varieties of the same species (Waterhemp | CALS (cornell.edu), Amaranthus rudis J.D.Sauer — The Plant List), which supports our identification. In this sense we classified all samples that were *A. rudis* or *A. tuberculatus* as *A. tuberculatus*. Two samples that were originally sent by the provider as *A. powellii* and *A. viridis* (see superscript information 3–4 from Table 1) were identified by us as *A. retroflexus.* After the provider of the seeds grew plants from the seeds of these two accessions, their phenotypic characterization confirmed our identification by protein biotyping, showing that our method was effective in correcting initial misidentifications from the seed batches from the provider.

In the case of the samples obtained from AAFC Harrow (Table 1, samples MIRL22-A.unknown-14 to 23), all samples were correctly identified according to the identification uncovered by the seed providers after our analyses were complete. We learned that a sample which was originally part of the blind samples (MIRL22-A.unknown-13 – Additional file 4), and initially classified as *A. caudatus*, corresponds to seeds whose correct taxonomic identification could not be confirmed by the provider (the sample was provided to them by an external collaborator years ago without verification). Therefore, this sample was excluded from our analysis as there was no morphological confirmation. Finally, MIRL22-A.unknown-17, identified as *A. hypochondriacus*, had technical replicates which diverged from the consensus identification (Additional file 4), but was nevertheless identified correctly by our majority rule.

Overall the application of our methodology to identify blind samples obtained from AAFC centres in Quebec and Ontario was 100% accurate when using our majority rule (at least 2 of the 3 tested seeds matched the expected species). When accounting for all biological replicates (3 seeds per accession) our success rate was 96%, due to a single seed from 3 replicates resulting in incorrect identification in two cases (Table 1 - MIRL22-A.unknown-09 and MIRL22-A.unknown-11).

**Table 1:** Identification of AAFC blind samples. MIRL22-A.unknown-07 to 12 were provided by AAFC Saint-Jean-sur-Richelieu. MIRL22-A.unknown-13 to 23 were provided by AAFC from Harrow. The MIRL22-A.unknown-13 was not included due to lack of valid morphological identification and MIRL22-A.unknown-15 ID was not assigned to any samples

Sample Name	Identification Call^1^	Average Bruker score^2^	Original Identification
MIRL22-A.unknown-07	*Amaranthus retroflexus*	2.62	*A. retroflexus*
	*Amaranthus retroflexus*	2.59	
	*Amaranthus retroflexus*	2.64	
MIRL22-A.unknown-08	*Amaranthus retroflexus*	2.69	*A. retroflexus* ^3^
	*Amaranthus retroflexus*	2.66	
	*Amaranthus retroflexus*	2.65	
MIRL22-A.unknown-09	*Amaranthus tuberculatus*	2.21	*Amaranthus tuberculatus*
	*Amaranthus tuberculatus*	2.26	
	*Amaranthus arenicola*	1.79	
MIRL22-A.unknown-10	*Amaranthus arenicola*	2.05	*Amaranthus arenicola*
	*Amaranthus arenicola*	1.95	
	*Amaranthus arenicola*	2.04	
MIRL22-A.unknown-11	*Amaranthus tuberculatus*	2.28	*Amaranthus tuberculatus/rudis*
	*Amaranthus tuberculatus*	2.03	
	*Amaranthus rudis*	2.14	
MIRL22-A.unknown-12	*Amaranthus retroflexus*	2.69	*A. retroflexus* ^4^
	*Amaranthus retroflexus*	2.65	
	*Amaranthus retroflexus*	2.57	
MIRL22-A.unknown-14	*Amaranthus hybridus*	2.45	*Amaranthus hybridus*
	*Amaranthus hybridus*	2.31	
	*Amaranthus hybridus*	2.34	
MIRL22-A.unknown-16	*Amaranthus palmeri*	2.28	*Amaranthus palmeri*
	*Amaranthus palmeri*	2.12	
	*Amaranthus palmeri*	2.25	
MIRL22-A.unknown-17	*Amaranthus hypochondriacus*	1.81	*Amaranthus hypochondriacus*
	*Amaranthus hypochondriacus*	1.80	
	*Amaranthus hypochondriacus*	1.88	
MIRL22-A.unknown-18	*Amaranthus spinosus*	2.44	*Amaranthus spinosus*
	*Amaranthus spinosus*	2.40	
	*Amaranthus spinosus*	2.39	
MIRL22-A.unknown-19	*Amaranthus tuberculatus*	2.33	*Amaranthus tuberculatus/rudis*
	*Amaranthus tuberculatus*	2.18	
	*Amaranthus tuberculatus*	1.95	
MIRL22-A.unknown-20	*Amaranthus retroflexus*	2.43	*Amaranthus retroflexus*
	*Amaranthus retroflexus*	2.43	
	*Amaranthus retroflexus*	2.38	
MIRL22-A.unknown-21	*Amaranthus powelli*	2.49	*Amaranthus powelli*
	*Amaranthus powelli*	2.51	
	*Amaranthus powelli*	2.47	
MIRL22-A.unknown-22	*Amaranthus albus*	2.27	*Amaranthus albus*
	*Amaranthus albus*	2.30	
	*Amaranthus albus*	2.31	
MIRL22-A.unknown-23	*Amaranthus blitoides*	2.57	*Amaranthus blitoides*
	*Amaranthus blitoides*	2.53	
	*Amaranthus blitoides*	2.63	

^1^ Each one of the three rows per sample corresponds to a single seed from the accession batch send to us for identification. The identification of each one of these seeds used a majority rule for 3 technical replicates per seed (if two technical replicates indicated one species, the sample was catalogued as such). Original data with technical replicates can be found in Additional file 4

^2^ Average score from 3 technical replicates on the same seed. When using 2 technical replicates for the majority rule identification, the average was done between the 2 matching reps

^3^ This was initially provided to us as *A. powellii*. After the providers grew the plants they confirmed the plants actually matched our identification (*A. retroflexus*)

^4^ This was initially provided to us as *A. viridis*. After the providers grew the plants they confirmed the plants actually matched our identification (*A. retroflexus*)

We then examined 60 blind samples sent from the CFIA SSST (Saskatoon Seed Science and Technology) unit. Out of 60 individual seeds, only two failed to produce a protein spectra in the three technical replicates (MIRL22-A.unknown 68 and 70 in Table 2 and Additional file 5); which can be most likely attributed to a technical error during processing of the samples. This means that our method was 97% effective in producing protein spectral profiles in this second set of blind samples. Out of 60 seeds where a phenotypic identification was performed by seed analysts, our protein biotyping assay was able to correctly predict the species for 52 samples, which means an accuracy of 87%. Three samples provided by the SSST but not identified by seed analysts, were tested by our protein biotyping assay but excluded from the validation analysis (MIRL22-A.unknown-37, 49 and 71 – Additional file 5). Out of the 8 samples where we could not correctly identify the source sample species, two were the samples where we did not obtain protein spectra. On the 6 samples where our biotyping identification did not match the phenotypic identification, there was no apparent relationship to the Brukker score, with some mismatches scoring below 2 and some scoring above 2 (Table 2). Three of the mismatches corresponded to seeds phenotypically identified as *A. palmeri*, where our identification matched *A. watsonii* (*A. palmeri’s* sister species) in two of those cases. In two cases a seed classified as *A. cruentus* matched *A. hypochondriacus* protein spectra, but our database did not have *A. cruentus* protein profiles, so this misidentification is expected. And in one case, a seed classified as *A. caudatus* matched a *A. hypochondriacus* MSP from our database.

**Table 2:** Identification of SSST blind samples. MIRL22-A.unknown-24 to 86 were provided by the Saskatoon Seed Science and Technology unit. The samples corresponding to MIRL22-A.unknown-37, 49 and 71 were not included due to lack of valid morphological identification

Sample Name	Identification Call^1^	Average Bruker score^2^	Original phenotypic Identification^3^
MIRL22-A.unknown-24	*Amaranthus spinosus*	1.30	*Amaranthus palmeri* atypical^5^
MIRL22-A.unknown-25	*Amaranthus retroflexus*	2.47	*Amaranthus retroflexus*
MIRL22-A.unknown-26	*Amaranthus tricolor*	1.92	*Amaranthus tricolor*
MIRL22-A.unknown-27	*Amaranthus powelli*	2.14	*Amaranthus powellii* subsp. *powellii*
MIRL22-A.unknown-28	*Amaranthus caudatus*	2.31	*Amaranthus caudatus*
MIRL22-A.unknown-29	*Amaranthus palmeri*	2.11	*Amaranthus palmeri*
MIRL22-A.unknown-30	*Amaranthus arenicola*	1.95	*Amaranthus arenicola*
MIRL22-A.unknown-31	*Amaranthus albus*	2.04	*Amaranthus albus*
MIRL22-A.unknown-32	*Amaranthus tuberculatus*	2.16	*Amaranthus tuberculatus* atypical
MIRL22-A.unknown-33	*Amaranthus californicus*	2.25	*Amaranthus californicus*
MIRL22-A.unknown-34	*Amaranthus californicus*	2.26	*Amaranthus californicus*
MIRL22-A.unknown-35	*Amaranthus spinosus*	2.24	*Amaranthus spinosus*
MIRL22-A.unknown-36	*Amaranthus caudatus*	2.34	*Amaranthus caudatus*
MIRL22-A.unknown-38	*Amaranthus tuberculatus*	2.09	*Amaranthus tuberculatus*
MIRL22-A.unknown-39	*Amaranthus albus*	1.92	*Amaranthus albus*
MIRL22-A.unknown-40	*Amaranthus californicus*	2.30	*Amaranthus californicus*
MIRL22-A.unknown-41	*Amaranthus powelli*	2.16	*Amaranthus powellii*
MIRL22-A.unknown-42	*Amaranthus albus*	2.19	*Amaranthus albus* atypical
MIRL22-A.unknown-43	*Amaranthus hypochondriacus*	1.80	*Amaranthus cruentus* ^5^
MIRL22-A.unknown-44	*Amaranthus hybridus*	2.42	*Amaranthus hybridus*
MIRL22-A.unknown-45	*Amaranthus albus*	2.08	*Amaranthus albus*
MIRL22-A.unknown-46	*Amaranthus retroflexus*	2.05	*Amaranthus retroflexus* atypical
MIRL22-A.unknown-47	*Amaranthus powelli*	2.41	*Amaranthus powellii* subsp. *bouchonii*
MIRL22-A.unknown-48	*Amaranthus powelli*	2.23	*Amaranthus powellii* subsp. *powellii*
MIRL22-A.unknown-50	*Amaranthus hypochondriacus*	2.03	*Amaranthus cruentus* ^5^
MIRL22-A.unknown-51	*Amaranthus tuberculatus*	2.17	*Amaranthus tuberculatus* atypical
MIRL22-A.unknown-52	*Amaranthus tuberculatus*	2.27	*Amaranthus tuberculatus*
MIRL22-A.unknown-53	*Amaranthus spinosus*	2.20	*Amaranthus spinosus*
MIRL22-A.unknown-54	*Amaranthus albus*	1.72	*Amaranthus albus*
MIRL22-A.unknown-55	*Amaranthus palmeri*	1.49	*Amaranthus palmeri* atypical
MIRL22-A.unknown-56	*Amaranthus tricolor*	2.22	*Amaranthus tricolor*
MIRL22-A.unknown-57	*Amaranthus watsonii*	2.00	*Amaranthus palmeri* ^5^
MIRL22-A.unknown-58	*Amaranthus powelli*	1.92	*Amaranthus powellii* subsp. *powellii*
MIRL22-A.unknown-59	*Amaranthus hybridus*	2.21	*Amaranthus hybridus*
MIRL22-A.unknown-60	*Amaranthus hypochondriacus*	1.97	*Amaranthus caudatus* ^5^
MIRL22-A.unknown-61	*Amaranthus arenicola*	2.14	*Amaranthus arenicola*
MIRL22-A.unknown-62	*Amaranthus tricolor*	2.14	*Amaranthus tricolor*
MIRL22-A.unknown-63	*Amaranthus powelli*	2.32	*Amaranthus powellii* subsp. *bouchonii*
MIRL22-A.unknown-64	*Amaranthus retroflexus*	2.35	*Amaranthus retroflexus*
MIRL22-A.unknown-65	*Amaranthus retroflexus*	1.97	*Amaranthus retroflexus* atypical
MIRL22-A.unknown-66	*Amaranthus retroflexus*	2.02	*Amaranthus retroflexus*
MIRL22-A.unknown-67	*Amaranthus retroflexus*	2.40	*Amaranthus retroflexus*
MIRL22-A.unknown-68	Flatline^4^	0.00	*Amaranthus palmeri* atypical
MIRL22-A.unknown-69	*Amaranthus arenicola*	2.03	*Amaranthus arenicola*
MIRL22-A.unknown-70	Flatline^4^	0.00	*Amaranthus tuberculatus* atypical
MIRL22-A.unknown-72	*Amaranthus palmeri*	2.05	*Amaranthus palmeri*
MIRL22-A.unknown-73	*Amaranthus watsonii*	2.02	*Amaranthus palmeri* ^5^
MIRL22-A.unknown-74	*Amaranthus hybridus*	2.43	*Amaranthus hybridus*
MIRL22-A.unknown-75	*Amaranthus powelli*	1.90	*Amaranthus powellii* subsp. *bouchonii*
MIRL22-A.unknown-76	*Amaranthus californicus*	2.14	*Amaranthus californicus*
MIRL22-A.unknown-77	*Amaranthus retroflexus*	2.30	*Amaranthus retroflexus*
MIRL22-A.unknown-78	*Amaranthus retroflexus*	2.16	*Amaranthus retroflexus*
MIRL22-A.unknown-79	*Amaranthus powelli*	2.52	*Amaranthus powellii*
MIRL22-A.unknown-80	*Amaranthus tricolor*	2.09	*Amaranthus tricolor*
MIRL22-A.unknown-81	*Amaranthus caudatus*	2.43	*Amaranthus caudatus*
MIRL22-A.unknown-82	*Amaranthus spinosus*	2.04	*Amaranthus spinosus*
MIRL22-A.unknown-83	*Amaranthus retroflexus*	2.12	*Amaranthus retroflexus*
MIRL22-A.unknown-84	*Amaranthus powelli*	2.40	*Amaranthus powellii* subsp. *bouchonii*
MIRL22-A.unknown-85	*Amaranthus albus*	1.97	*Amaranthus albus* atypical
MIRL22-A.unknown-86	*Amaranthus retroflexus*	2.41	*Amaranthus retroflexus*

^1^ Each row corresponds to a single seed selected from a single source. The bioyping identification call of each one of these seeds used a majority rule for 3 technical replicates per seed (if two technical replicates indicated one species, the sample was catalogued as such). Original data with technical replicates can be found in Additional file 5

^2^ Average score from 3 technical replicates on the same seed. When using 2 technical replicates for the majority rule identification, the average was done between the 2 matching reps

^3^ Samples marked as ‘atypical’ correspond to seeds that are less mature or outside the range of variation for typical seeds and would result in uncertain identification

^4^ Flatlined samples did not produce a protein spectra in any of the three technical replicates

^5^ Mismatches between protein biotyping and phenotypic identification

## Discussion

Protein biotyping using MALDI-TOF MS has commonly been used to identify pathogens [47, 49, 57–59], due to its high efficiency, throughput and manageable cost. In pathogens, protein profiles generated by species and strains [47, 49, 60] show a reliable identification methodology which can be efficiently used to classify samples and cluster related organisms. In plants, protein biotyping was tested to distinguish *Impatiens* species and regional biotypes of the invasive weed *Impatiens glandulifera* [52], yielding accuracy in identification of two sets of 12 blind samples of 100% and 92% respectively. Our methodology, which was derived from the method reported on [52], resulted in species identification accuracies of 100% and 87% for the two sets of 15 and 60 blind samples from the genus *Amaranthus*.

One of our main objectives in testing biotyping to identify *Amaranthus* seeds is to find a rapid diagnostics test to support seed identification during trade under the Canadian regulatory framework for international export. At times, phenotypic plasticity and similarity from seeds will result in difficulties to accurately identify an *Amaranthus* seed using morphological methods. While DNA sequencing has uncovered polymorphisms that can be utilized to differentiate species in *Amaranthus* species, diagnostic assays are usually targeted towards one species [39, 61, 62], and do not examine related species. Comparatively, the identification of *Amaranthus* samples using protein biotyping is more efficient than developing and using DNA barcodes. Protocols tested in our lab show that two 96-well plates can be processed in a day, covering seed grinding, protein extraction, dilution, plate spotting and MALDI biotyping. In the meantime, obtaining a barcode comprises performing a PCR, gel electrophoresis, PCR purification, sequencing, sequence purification, and a sequencing run, which can take at least 2–3 days for the same number of samples. While both methodologies would require the generation of a database to identify blind samples or validate known samples, the generation of the protein spectra itself does not require any previous knowledge to obtain the protein profile. DNA barcoding requires some previous knowledge of the target region to design primers that can amplify the barcode. While traditional chloroplast and Internal Transcribed Spacer (ITS) regions can use conserved primers, these regions do not always provide the best resolution in all plant genera. This has also been seen in studies outside the plant realm, where the resolution of traditional 16 S rDNA markers at the species level was below the power of MALDI-TOF biotyping [63]. In our case, our lab has used genome skimming [29] to assemble full chloroplast genomes to find new *Amaranthus* spp. barcodes. However, the generation of the protein spectra database does require some time investment since it is necessary to have several accessions per species that represent the variability of a species, so that blind samples can be accurately identified. While we did not test infra-species resolution, previous studies show that genotype and variety discrimination is possible [52, 53]. Additionally, we standardized our methodology for seeds since previous research has shown that other tissue under development (like young leaves) might not have a stable protein composition [50]. However, we expect to test other plant tissues in the future. A comparison of protein biotyping and DNA barcoding for molecular identification is shown below (Table 3).

**Table 3:** Comparison of protein biotyping and DNA barcoding. Comparisons are based on work in our lab with *Amaranthus* species

	Protein Biotyping	DNA barcoding
**Throughput**	> 96 samples	> 96 samples
**Time to run 2 96-well plates**	1 day	2-3days
**Species Accuracy**	87–100%	~ 100%
**Infra-species resolution**	Non-tested on this study	High
**Database investment time**	1 week	2 weeks – 1 month (genome skimming)
**Tissue**	seed	All plant tissue

Our preliminary dendrograms did not show distinctive clustering for samples of some of the species in cluster 1 (Fig. 3), that traditionally belong to the Hybridus clade [7]. While the protein spectra dendrograms are not based on a phylogenetics algorithm, many studies of rapidly evolving markers have produced polytomies of the Hybridus clade, and have concluded a sister-lineage relationship of *A. powellii* and *A. retroflexus* to the *A. hybridus* group [7, 12]. In fact, a previous study using Genotyping By Sequencing (GBS) in the *Amaranthus* genus showed that there was no strong separation by species in the Hybridus complex, similarly to what was found in our results [64], reflecting strong gene flow and potential hybridization in this clade. Nevertheless, several species from the Hybridus clade were part of the blind samples provided to us and were correctly classified when compared to our database, which was enriched with multiple accessions per species. This shows that dendrograms can be used as a preliminary step for comparison of the protein profiles, but an enriched database with multiple accessions per species, is necessary to represent inherent variability between populations of a species, therefore increasing the accuracy of identification.

We then performed identification of blind samples using our protein spectra database (Additional file 2). On six blind samples where we did not achieve correct identification according to the phenotypic classification of samples provided, there was no apparent relationship to the Bruker matching score, meaning both high (> 2) and low (< 2) scores were present in mismatches. Bruker scores are calculated based on comparison of the number of matching peaks between test and database spectra and on the concordance of peak height and symmetry [52]. In our case, only one score (1.30) indicated low relatedness, on a mismatch between *A. spinosus* and *A. palmeri* (MIRL22-A.unknown-24 – Table 2). These two species, are nevertheless closely related [7, 64], as is the case for two other mismatches where our biotyping indicating *A. watsonii* had *A. palmeri* as phenotypic identification. In this latter case, previous research has shown that *A. watsonii* and *A. palmeri* are sister species according to morphological and molecular characterization [7, 65]. This is also suggested by comparison of full chloroplast genomes done in our lab (unpublished), and by a recently published plastome comparison analysis [66]. Additionally, comparative genomics has shown that *A. palmeri* and *A. watsonii* have similar genome sizes, transposon content, higher heterozigosity and a common origin of dioecy among dioecious species [65]. Work comparing their plastomes shows these two species have the closest relationship among all dioecious species studied [66], and even suggests that due to their morphological similarity and very small genetic distance, they could be a single polymorphic species instead of two different species. This would be in agreement with the inability of the protein biotyping to distinguish between the two alleged species.

Three samples that were identified as *A. hypochondriacus* by protein biotyping (MIRL-A.unknown 43, 50 and 60 – Table 2) did not match the seed phenotypic identification of *A. cruentus*, for the two first samples, and *A. caudatus* from the third sample. Our inability to identify *A. cruentus* seeds stems from the absence of this species in our database. However, *A. hypochondriacus, A. cruentus* and *A. caudatus* are all part of the hybridus clade from *Amaranthus* species, and closely related according to previous studies [7, 64–68]. The three species are grain domesticated amaranths originated from *A. hybridus* [7, 69]. Close evolutionary relationship potentially plays a role in common proteins showing up between these species in our biotyping analyses, but we also expect that continuing to enrich our *Amaranthus* protein spectra library with more accessions in each species, will result in better resolution.

## Conclusions

Protein biotyping is an efficient methodology to identify species in the genus *Amaranthus.* While some of the main problems that are present with DNA barcoding can arise when studying this complex genus, we determined that enrichment with a larger number of diverse populations in each species can increase accuracy in calling the correct species. As we gather more species and populations for our *Amaranthus* library we expect our accuracy and power to identify these species will increase.

While DNA barcoding and PCR tests derived from sequence analysis are still at the core of species identification, new alternate methods can provide complementary tools where difficulties exist with these more traditional methods and for specific situations. In our case protein biotyping has provided a quick method to identify species from seed protein, aiding seed analysts when seed plasticity makes it difficult to do a phenotypic characterization. This tool becomes important in the regulatory context of trade as a last step of classification when other tools or analysis are not sufficient. We also expect that the methodology can be easily transferred to other plants that are regulated at the federal level, but outside the regulatory context the protocols shown here can be applied to different plant genera for identification purposes.

## Methods

### Plant material

*Amaranthus* spp. seeds for our protein database were obtained from collaborators at Agriculture and Agri-Food Canada, Harrow, ON, and from the Germplasm Resource Information Centre (GRIN-Global) which is part of the U.S. National Plant Germplasm System. Blind *Amaranthus* spp. seed samples were provided by Dr. Marie-Josée Simard (Agriculture Agri-Food Canada, Saint-Jean-sur-Richelieu, QC), Dr. Robert Nurse (Agriculture and Agri-Food Canada, Harrow, ON), and Dr. Ruojing Wang (Canadian Food Inspection Agency, Saskatoon, SK).

### MALDI-TOF-MS sample preparation

#### Preliminary test

Individual seeds from four Brassicaceae species were manually ground in 2 mL rounded bottom microfuge tubes. Seed protein extraction was modified from the original protocol [52] to place the ground seeds in 50 µL of a 1:1 Acetonitrile (for UHPCL, Sigma-Aldrich, Oakville, Canada)-Formic Acid (ACS reagent, ≥ 96%, Sigma-Aldrich, Oakville, Canada). This was followed by a centrifugation step at maximum speed (20,000 rcf) to separate the supernatant carrying the proteins from the seed debris. Since the expected amount of protein needed to create a clear spectra was initially unknown, we generated two testing dilutions (1:1 and 1:10), by mixing either 5 µL of supernatant with 5 µL of a 1:1 Acetonitrile-Formic Acid solution, or 1µL of supernatant with 9 µL of the Acetonitrile-Formic Acid solution. Then 0.5 µL of each dilution per sample was added to the 96-well MBT biotarget plate (Bruker, Billerica, MA, US) followed by addition of 0.5 µL of HCCA dissolved matrix solution (Bruker, Billerica, MA, US). This mix was left to dry at room temperature (without exceeding one hour), and then overlaid with one additional 1 µL of HCCA matrix solution which was left to dry. One microliter of BTS (Bruker Bacterial Test Standard - Bruker, Billerica, MA, US) was spotted in duplicate on the MALDI target plate and allowed to dry at room temperature. The entire spot was overlaid with 1 µL of HCCA solution and allowed to dry at room temperature.

#### Final protocol

Single seeds were placed in 2 mL rounded bottom microfuge tubes and a 5 mm steel bead was added per tube. Samples were loaded into a tissue lyzer (Tissue Lyser II, Qiagen, Germantown, MD, US) and fully ground for 1 min at 25 Hz. Tubes were centrifuged at 12,000 rcf for 1 min and then 50 µL of a 1:1 solution of Formic Acid (ACS reagent, ≥ 96%, Sigma-Aldrich, Oakville, Canada) and Acetonitrile (for UHPCL, Sigma-Aldrich, Oakville, Canada) was added, and the tube was briefly vortexed. Samples were then centrifuged at 12,000 rcf for 1 min. Using a new tube 2 µL of the supernatant were mixed with 4 µL of HCCA dissolved matrix solution (Bruker, Billerica, MA, US) and 2 µL of 1:1 Formic acid – Acetonitrile solution. One microliter of this solution was pipetted onto the 96-well MBT biotarget plate (Bruker, Billerica, MA, US) and dried at room temperature (without exceeding one hour). The sample was then overlaid with 1 µL of HCCA matrix solution and allowed to dry at room temperature. One microliter of BTS was spotted in duplicate on the MALDI target plate and allowed to dry at room temperature. The entire spot was overlaid with 1 µL of HCCA solution and allowed to dry at room temperature.

#### MALDI-TOF-MS

Mass spectrometry was carried out over a range of 2 kDa to 20 kDa using a Bruker MALDI Biotyper Sirius instrument (Bruker, Billerica, MA, US), together with the MBT software suite including FlexControl Sirius (version 3.4) and MBT Compass (version 4.1) for the acquisition of spectra. The Smartbeam MBT laser was used with a frequency of 200 Hz, initial output power of 30% and maximum output of 40%, with a 2000 μm spot size and 240 laser shots per sample. The laser’s settings were Global Attenuator Range (0%), Attenuator Offset (48%) and Attenuator Range (20%). The ion source voltage was 19.77 kV and 18.01 kV for each source. The samples were spotted onto Bruker MBT Biotarget 96 target plates. The calibration followed manufacturer’s instructions using Bruker’s BTS control, comprised of *E. coli* ribonuclease A and myoglobin. Calibration peaks had masses at 3637.8; 5096.8; 5381.4; 6255.4; 7274.5; 10,300.2; 13,683.2, and 16,952.3 Da. Spectra were acquired using FlexControl Sirius (version 3.4) and MBT Compass (version 4.1) software under the manufacturer’s default settings. The generated spectra were used for principal-component analysis (PCA) and to create PCA dendrograms using the 70 main peaks of each spectra.

#### Database entries

To generate a database of protein spectra that can be used to test blind samples, database entries require 10 technical replicates per sample and 3 spectral readings per technical replicate, so a total of 30 spectra are compiled at the end per sample. This is necessary in order to obtain the minimum of 20 high quality spectra to be included in the database as a Main Spectra (MSP), which is calculated as an average of the 20–30 spectral readings. We performed this process for three seeds for each one of the accessions that were included in the generated database.

Database samples were plated on the MBT target plate (including at least 1 BTS spot and 1 Blank spot for quality control), and the target plate was placed into the equipment to acquire data using the FlexControl Software (version 3.4). BTS Calibration was performed through detection of the major eight spectral peaks in the expected range of the protein profile (Da range of +/-300ppm). The resulting BTS spectra was used as input for analysis with the FlexAnalysis software (version 3.4), where BTS spectra were smoothed, baseline was subtracted and BTS masses were identified. Sample spectra to create the database were also smoothed, baseline subtracted and outliers eliminated. A minimum of 20 spectra were selected to generate each one of the MSPs for the database.

The MSPs to be added to the database were generated in Compass Explorer software (version 4.1). The newly created MSPs were matched against all created database spectra to validate identity of the species. Spectra validated in this way are expected to match the same species with a Bruker score of 2.00 or higher. Each of the major 70 peaks used to create the MSP is expected to be at least 25% of the times in the 20–30 spectral sample reading compiled. MSPs are then added to a library of choice using the “Start Taxonomy Tree Editor”. We generated a library with a total of 16 species and 56 accessions from the *Amaranthus* genus. Our database was created based on preliminary information of target species with North American distribution and that can be confused with species of regulatory concern when performing morphological classification.

#### Blind sample testing

The first set of blind seed samples provided by Dr. Robert Nurse (AAFC, Harrow, ON) and Dr. Marie-Josée Simard (AAFC, Saint-Jean-sur-Richelieu, QC) had multiple seeds per accession and therefore three biological replicates (individual seeds in each accession) and three technical replicates per blind sample (coming from each seed) were tested. A second set of blind samples was obtained from Dr. Ruojing Wang from the CFIA Saskatoon Seed Science and Technology (SSST) Lab. These latter samples were chosen from regulated *Amaranthus* species, and visually similar seeds from species that occur in Canada and the United States. For this second set, mature intact seeds were selected from a single source, and samples were shuffled and numbered using a random number generator. Individual seeds were then sent in vials to our lab, with each species/sample having 2–4 replicates throughout the total number of randomized vials. Three technical replicates per seed were run in the Bruker protein biotyper. All samples from the two blind sets received were compared against the generated *Amaranthus* spp. MSPs database built in-house. Spectra from blind samples were compared against database entries, and were scored using Bruker Identification scores that establish relatedness between spectra where a score > 2.00 indicates high relatedness and a score below 1.70 indicates low relatedness. Bruker Scores are computed as described in Reeve & Pollard [52]. However, we considered matches and mismatches based on the top hits when comparing a blind sample to the database, regardless of the score. Validation of our identification was done by sending our results back to the three seed providers so they could match their phenotypic characterization with our biotyping identification. AAFC providers usually grew plants for characterization while the CFIA SSST performed expert analyst seed characterization.

### Electronic supplementary material

Below is the link to the electronic supplementary material.

Additional file 1 (.Pptx) (A) Clustering of protein spectra corresponding to four Brassica species. (B) c (Brassica carinata), B. r (Brassica rapa), B. j (Brassica juncea), B. n (Brassica napus), alt = Winter variety. B. Clustering of protein spectra corresponding to four Brassica species including two species with accessions from different years. Cluster 1: Brassica rapa, 2: Brassica juncea, 3: Brassica carinata, 4: Brassica napus. Winter B. napus (w). Color legends show the year the accession was collected.

Supplementary Material 2: Additional file 2 (.Xlsx) Protein biotyping database. Details of all accessions used.

Supplementary Material 3: Additional file 3 (.Pdf) MSP Validation Check 2023-05-10 Library. Bruker Daltonik MALDI Biotyper Classification Results.

Supplementary Material 4: Additional file 4. (.Xlsx) Blind sample testing for samples from AAFC in Table 1. For each sample, three seeds were tested, and for each seed three technical replicates were ran.

Supplementary Material 5: Additional file 5. (.Xlsx) Blind sample testing for samples from CFIA SSST in Table 2. Three technical replicates were run per seed.

## Data Availability

Data generated or analysed during this study are included in this published article [and its supplementary information files]. Protein spectra would be available upon request.
