# Supplementary material for: Seed protein biotyping in Amaranthus species: a tool for rapid identification of weedy amaranths of concern
Source: Plant Methods. 2023 Dec 11;19:143. doi: 10.1186/s13007-023-01116-9 (PMC10712156; doi:10.1186/s13007-023-01116-9)
Supplement: Supplementary file 1 — Additional file 1 (.Pptx) (A) Clustering of protein spectra corresponding to four Brassica species. (B) c (Brassica carinata), B. r (Brassica rapa), B. j (Brassica juncea), B. n (Brassica napus), alt = Winter variety. B. Clustering of protein spectra corresponding to four Brassica species including two species with accessions from different years. Cluster 1: Brassica rapa, 2: Brassica juncea, 3: Brassica carinata, 4: Brassica napus. Winter B. napus (w). Color legends show the year the accession was collected. [file 13007_2023_1116_MOESM1_ESM.pptx]

## Slide 1
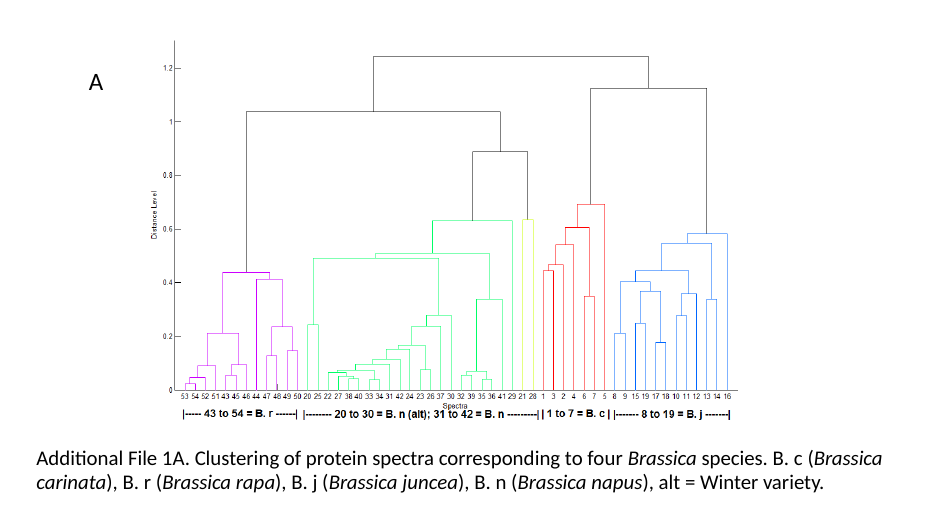

A
Additional File 1A. Clustering of protein spectra corresponding to four Brassica species. B. c (Brassica carinata), B. r (Brassica rapa), B. j (Brassica juncea), B. n (Brassica napus), alt = Winter variety.

## Slide 2
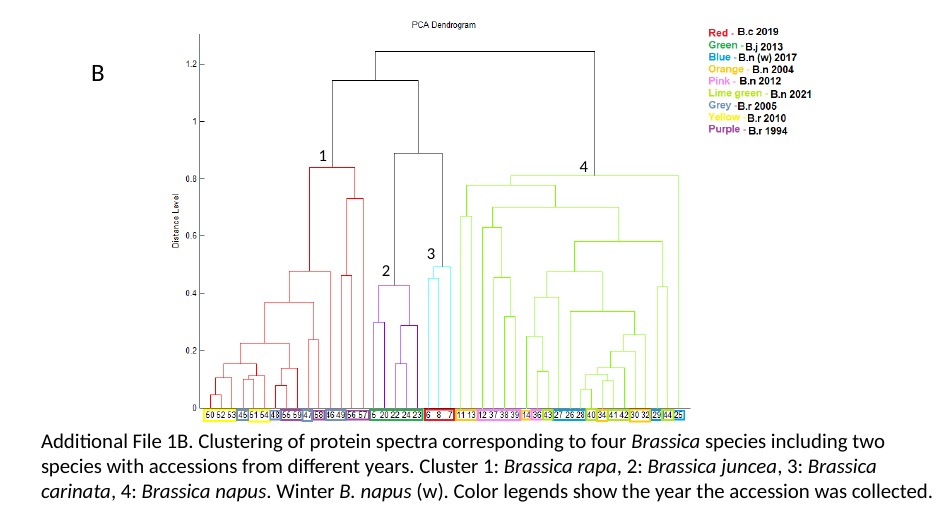

B
1
4
3
2
Additional File 1B. Clustering of protein spectra corresponding to four Brassica species including two species with accessions from different years. Cluster 1: Brassica rapa, 2: Brassica juncea, 3: Brassica carinata, 4: Brassica napus. Winter B. napus (w). Color legends show the year the accession was collected.
