# Supplementary material for: Seed protein biotyping in Amaranthus species: a tool for rapid identification of weedy amaranths of concern
Source: Plant Methods. 2023 Dec 11;19:143. doi: 10.1186/s13007-023-01116-9 (PMC10712156; doi:10.1186/s13007-023-01116-9)
Supplement: Supplementary file 3 — Supplementary Material 3: Additional file 3 (.Pdf) MSP Validation Check 2023-05-10 Library. Bruker Daltonik MALDI Biotyper Classification Results. [file 13007_2023_1116_MOESM3_ESM.pdf]

Bruker Daltonik MALDI Biotyper Classification Results

Project Info:

Project Name:	**MSP Validation Check 2023-05-10 Library**
Project Description:	Bruker Project Description
Project Owner:	Admin
Project Creation Date/Time:	2023-06-23 3:05:34 PM
Project Analyte Count:	142
Project Type:	Development
Validation:	not present
Validation Position:	

Result Overview

Analyte Name	Organism (best match)	Score Value	Organism (second best match)	Score Value
Amaranthus palmeri MALI PI549158-1 MIRL (+++) (B)	Amaranthus palmeri	3	Amaranthus palmeri	2.69
Amaranthus palmeri MALI PI549158-2 MIRL (+++) (B)	Amaranthus palmeri	3	Amaranthus palmeri	2.69
Amaranthus palmeri MALI PI549158-3 MIRL (+++) (B)	Amaranthus palmeri	3	Amaranthus palmeri	2.42
Amaranthus palmeri USA(AZ) PI632236-1 MIRL (+++) (A)	Amaranthus palmeri	3	Amaranthus palmeri	2.09
Amaranthus palmeri USA(AZ) PI632236-2 MIRL (+++) (B)	Amaranthus palmeri	3	Amaranthus palmeri	2.41

Amaranthus palmeri USA(AZ) PI632236-3 MIRL<br>**(+++) (A)**	Amaranthus palmeri	3	Amaranthus palmeri	2.26
Amaranthus tuberculatus IOWA PI 553086-1 MIRL<br>**(+++) (A)**	Amaranthus tuberculatus	3	Amaranthus tuberculatus	2.41
Amaranthus tuberculatus IOWA PI 553086-2 MIRL<br>**(+++) (A)**	Amaranthus tuberculatus	3	Amaranthus tuberculatus	2.39
Amaranthus spinosus PI 632248-1 MIRL<br>**(+++) (B)**	Amaranthus spinosus	3	Amaranthus spinosus	2.69
Amaranthus spinosus PI 632248-3 MIRL<br>**(+++) (B)**	Amaranthus spinosus	3	Amaranthus spinosus	2.69
Amaranthus spinosus PI 632248-2 MIRL<br>**(+++) (B)**	Amaranthus spinosus	3	Amaranthus spinosus	2.62
Amaranthus tuberculatus IOWA PI 553086-3 MIRL<br>**(+++) (A)**	Amaranthus tuberculatus	3	Amaranthus tuberculatus	2.32
Amaranthus tuberculatus KANSAS PI 60743-1 MIRL<br>**(+++) (B)**	Amaranthus tuberculatus	3	Amaranthus tuberculatus	2.23
Amaranthus tuberculatus KANSAS PI 60743-2 MIRL<br>**(+++) (A)**	Amaranthus tuberculatus	3	Amaranthus tuberculatus	2.14
Amaranthus tuberculatus KANSAS PI 60743-3 MIRL<br>**(+++) (B)**	Amaranthus tuberculatus	3	Amaranthus tuberculatus	2.21
Amaranthus arenicola PI 599670-1 MIRL<br>**(+++) (A)**	Amaranthus arenicola	3	Amaranthus arenicola	2.22
Amaranthus arenicola PI 599670-2 MIRL<br>**(+++) (A)**	Amaranthus arenicola	3	Amaranthus arenicola	2.12
Amaranthus arenicola PI 599670-3 MIRL<br>**(+++) (A)**	Amaranthus arenicola	3	Amaranthus arenicola	2.08
	Amaranthus watsonii	3	Amaranthus spinosus	2.21

Amaranthus watsonii PI 633593-1 MIRL<br>**(+++) (B)**				
Amaranthus watsonii PI 633593-2 MIRL<br>**(+++) (A)**	Amaranthus watsonii	3	Amaranthus watsonii	2.3
Amaranthus watsonii PI 633593-3 MIRL<br>**(+++) (A)**	Amaranthus watsonii	3	Amaranthus watsonii	2.31
Amaranthus palmeri MEXICO PUEBLA PI604557-1 MIRL<br>**(+++) (B)**	Amaranthus palmeri	3	Amaranthus palmeri	2.39
Amaranthus palmeri MEXICO PUEBLA PI604557-2 MIRL<br>**(+++) (B)**	Amaranthus palmeri	3	Amaranthus palmeri	2.52
Amaranthus palmeri MEXICO PUEBLA PI604557-3 MIRL<br>**(+++) (B)**	Amaranthus palmeri	3	Amaranthus palmeri	2.52
Amaranthus palmeri MEXICO VERACRUZ PI667167-1 MIRL<br>**(+++) (A)**	Amaranthus palmeri	3	Amaranthus palmeri	2.06
Amaranthus palmeri MEXICO VERACRUZ PI667167-2 MIRL<br>**(+++) (B)**	Amaranthus palmeri	3	Amaranthus spinosus	2.09
Amaranthus palmeri MEXICO VERACRUZ PI667167-3 MIRL<br>**(+++) (A)**	Amaranthus palmeri	3	Amaranthus palmeri	1.81
Amaranthus hypochondriacus PI658730-1 MIRL<br>**(+++) (B)**	Amaranthus hypochondriacus	3	Amaranthus hypochondriacus	2.8
Amaranthus hypochondriacus PI658730-2 MIRL<br>**(+++) (B)**	Amaranthus hypochondriacus	3	Amaranthus hypochondriacus	2.8
Amaranthus albus PI633580-1 MIRL<br>**(+++) (A)**	Amaranthus albus	3	Amaranthus albus	2.65
Amaranthus albus PI633580-3 MIRL<br>**(+++) (A)**	Amaranthus albus	3	Amaranthus albus	2.65
	Amaranthus hypochondriacus	3	Amaranthus hypochondriacus	2.79

Amaranthus hypochondriacus PI658730-3 MIRL **(+++) (B)**				
Amaranthus albus PI633580-2 MIRL **(+++) (A)**	Amaranthus albus	3	Amaranthus albus	2.23
Amaranthus palmeri DAKAR PI633587-1 MIRL **(+++) (B)**	Amaranthus palmeri	3	Amaranthus palmeri	2.63
Amaranthus palmeri DAKAR PI633587-2 MIRL **(+++) (A)**	Amaranthus palmeri	3	Amaranthus palmeri	2.63
Amaranthus palmeri DAKAR PI633587-3 MIRL **(+++) (B)**	Amaranthus palmeri	3	Amaranthus palmeri	2.59
Amaranthus tuberculatus IOWA PI674264-1 MIRL **(+++) (A)**	Amaranthus tuberculatus	3	Amaranthus tuberculatus	2.43
Amaranthus tuberculatus IOWA PI674264-2 MIRL **(+++) (B)**	Amaranthus tuberculatus	3	Amaranthus tuberculatus	2.43
Amaranthus tuberculatus IOWA PI674264-3 MIRL **(+++) (A)**	Amaranthus tuberculatus	3	Amaranthus tuberculatus	2.25
Amaranthus tuberculatus IOWA PI604247-1 MIRL **(+++) (A)**	Amaranthus tuberculatus	3	Amaranthus tuberculatus	2.74
Amaranthus tuberculatus IOWA PI604247-2 MIRL **(+++) (A)**	Amaranthus tuberculatus	3	Amaranthus tuberculatus	2.74
Amaranthus tuberculatus IOWA PI604247-3 MIRL **(+++) (A)**	Amaranthus tuberculatus	3	Amaranthus tuberculatus	2.63
Amaranthus palmeri ARIZONA PI686461-1 MIRL **(+++) (B)**	Amaranthus palmeri	3	Amaranthus palmeri	2.69
Amaranthus palmeri ARIZONA PI686461-2 MIRL **(+++) (B)**	Amaranthus palmeri	3	Amaranthus palmeri	2.49
	Amaranthus palmeri	3	Amaranthus palmeri	2.56

Amaranthus palmeri ARIZONA PI686461-3 MIRL **(+++) (B)**				
Amaranthus palmeri DAKAR PI633586-1 MIRL **(+++) (B)**	Amaranthus palmeri	3	Amaranthus palmeri	2.74
Amaranthus palmeri DAKAR PI633586-2 MIRL **(+++) (B)**	Amaranthus palmeri	3	Amaranthus palmeri	2.77
Amaranthus palmeri DAKAR PI633586-3 MIRL **(+++) (B)**	Amaranthus palmeri	3	Amaranthus palmeri	2.77
Amaranthus caudatus NJ AMES 5687-1 MIRL **(+++) (B)**	Amaranthus caudatus	3	Amaranthus caudatus	2.63
Amaranthus caudatus NJ AMES 5687-2 MIRL **(+++) (B)**	Amaranthus caudatus	3	Amaranthus caudatus	2.76
Amaranthus caudatus NJ AMES 5687-3 MIRL **(+++) (B)**	Amaranthus caudatus	3	Amaranthus caudatus	2.76
Amaranthus blitoides CANADA PI608663-1 MIRL **(+++) (A)**	Amaranthus blitoides	3	Amaranthus blitoides	2.78
Amaranthus blitoides CANADA PI608663-2 MIRL **(+++) (A)**	Amaranthus blitoides	3	Amaranthus blitoides	2.78
Amaranthus blitoides CANADA PI608663-3 MIRL **(+++) (A)**	Amaranthus blitoides	3	Amaranthus blitoides	2.44
Amaranthus viridis FLORIDA PI654388-1 MIRL **(+++) (A)**	Amaranthus viridis	3	Amaranthus viridis	2.88
Amaranthus viridis FLORIDA PI654388-2 MIRL **(+++) (A)**	Amaranthus viridis	3	Amaranthus viridis	2.93
Amaranthus viridis FLORIDA PI654388-3 MIRL **(+++) (A)**	Amaranthus viridis	3	Amaranthus viridis	2.93
Amaranthus blitum var. pseudogracilis NC PI632245-1	Amaranthus blitum	3	Amaranthus blitum	2.89

MIRL<br>(+++) (A)				
Amaranthus blitum var. pseudogracilis NC PI632245-2 MIRL<br>(+++) (A)	Amaranthus blitum	3	Amaranthus blitum	2.89
Amaranthus blitum var. pseudogracilis NC PI632245-3 MIRL<br>(+++) (A)	Amaranthus blitum	3	Amaranthus blitum	2.83
Amaranthus tricolor NJ AMES 5303-1 MIRL<br>(+++) (A)	Amaranthus tricolor	3	Amaranthus tricolor	2.78
Amaranthus tricolor NJ AMES 5303-2 MIRL<br>(+++) (A)	Amaranthus tricolor	3	Amaranthus tricolor	2.74
Amaranthus tricolor NJ AMES 5303-3 MIRL<br>(+++) (A)	Amaranthus tricolor	3	Amaranthus tricolor	2.78
Amaranthus hybridus HARROW 1 MIRL<br>(+++) (B)	Amaranthus hybridus	3	Amaranthus hybridus	2.59
Amaranthus hybridus HARROW 2 MIRL<br>(+++) (B)	Amaranthus hybridus	3	Amaranthus hybridus	2.84
Amaranthus hybridus HARROW 3 MIRL<br>(+++) (B)	Amaranthus hybridus	3	Amaranthus hybridus	2.84
Amaranthus powelli sub. powelli ME AMES 29205-1 MIRL<br>(+++) (B)	Amaranthus powelli	3	Amaranthus powelli	2.88
Amaranthus powelli sub. powelli ME AMES 29205-2 MIRL<br>(+++) (B)	Amaranthus powelli	3	Amaranthus powelli	2.88
Amaranthus powelli sub. powelli ME AMES 29205-3 MIRL<br>(+++) (B)	Amaranthus powelli	3	Amaranthus powelli	2.87
Amaranthus retroflexus CANADA AMES 5328-1 MIRL<br>(+++) (B)	Amaranthus retroflexus	3	Amaranthus retroflexus	2.85
		3		2.72

Amaranthus retroflexus CANADA AMES 5328-2 MIRL **(+++) (B)**	Amaranthus retroflexus		Amaranthus retroflexus	
Amaranthus retroflexus CANADA AMES 5328-3 MIRL **(+++) (B)**	Amaranthus retroflexus	3	Amaranthus retroflexus	2.85
Amaranthus retroflexus HARROW 1 MIRL **(+++) (B)**	Amaranthus retroflexus	3	Amaranthus retroflexus	2.9
Amaranthus retroflexus HARROW 2 MIRL **(+++) (B)**	Amaranthus retroflexus	3	Amaranthus retroflexus	2.76
Amaranthus retroflexus HARROW 3 MIRL **(+++) (B)**	Amaranthus retroflexus	3	Amaranthus retroflexus	2.9
Amaranthus powelli HARROW 1 MIRL **(+++) (B)**	Amaranthus powelli	3	Amaranthus powelli	2.75
Amaranthus powelli HARROW 2 MIRL **(+++) (B)**	Amaranthus powelli	3	Amaranthus powelli	2.76
Amaranthus powelli HARROW 3 MIRL **(+++) (B)**	Amaranthus powelli	3	Amaranthus powelli	2.75
Amaranthus rudis HARROW 1 MIRL **(+++) (B)**	Amaranthus rudis	3	Amaranthus rudis	2.45
Amaranthus rudis HARROW 2 MIRL **(+++) (B)**	Amaranthus rudis	3	Amaranthus rudis	2.45
Amaranthus rudis HARROW 3 MIRL **(+++) (B)**	Amaranthus rudis	3	Amaranthus rudis	2.3
Amaranthus albus HARROW 1 MIRL **(+++) (A)**	Amaranthus albus	3	Amaranthus albus	2.66
Amaranthus albus HARROW 2 MIRL **(+++) (A)**	Amaranthus albus	3	Amaranthus albus	2.66
	Amaranthus albus	3	Amaranthus albus	2.53

Amaranthus albus HARROW 3 MIRL<br>**(+++) (A)**				
Amaranthus blitoides HARROW 2 MIRL<br>**(+++) (A)**	Amaranthus blitoides	3	Amaranthus blitoides	2.71
Amaranthus blitoides HARROW 3 MIRL<br>**(+++) (A)**	Amaranthus blitoides	3	Amaranthus blitoides	2.71
Amaranthus spinosus HARROW 1 MIRL<br>**(+++) (B)**	Amaranthus spinosus	3	Amaranthus spinosus	2.67
Amaranthus spinosus HARROW 2 MIRL<br>**(+++) (B)**	Amaranthus spinosus	3	Amaranthus spinosus	2.67
Amaranthus spinosus HARROW 3 MIRL<br>**(+++) (B)**	Amaranthus spinosus	3	Amaranthus spinosus	2.62
Amaranthus spinosus NC PI632248-RE1 MIRL<br>**(+++) (B)**	Amaranthus spinosus	3	Amaranthus spinosus	2.74
Amaranthus spinosus NC PI632248-RE2 MIRL<br>**(+++) (B)**	Amaranthus spinosus	3	Amaranthus spinosus	2.72
Amaranthus spinosus NC PI632248-RE3 MIRL<br>**(+++) (B)**	Amaranthus spinosus	3	Amaranthus spinosus	2.75
Amaranthus palmeri MEXICO VERACRUZ PI667167-RE1 MIRL<br>**(+++) (B)**	Amaranthus palmeri	3	Amaranthus palmeri	2.32
Amaranthus palmeri MEXICO VERACRUZ PI667167-RE2 MIRL<br>**(+++) (A)**	Amaranthus palmeri	3	Amaranthus palmeri	2.36
Amaranthus palmeri MEXICO VERACRUZ PI667167-RE3 MIRL<br>**(+++) (B)**	Amaranthus palmeri	3	Amaranthus palmeri	2.36
Amaranthus watsonii MEXICO COLIMA PI633593-RE1 MIRL<br>**(+++) (B)**	Amaranthus watsonii	3	Amaranthus watsonii	2.35
	Amaranthus watsonii	3	Amaranthus palmeri	2.44

Amaranthus watsonii MEXICO COLIMA PI633593-RE2 MIRL (+++) (B)				
Amaranthus watsonii MEXICO COLIMA PI633593-RE3 MIRL (+++) (B)	Amaranthus watsonii	3	Amaranthus watsonii	2.28
Amaranthus powellii sub. Powellii TEXAS PI632241-1 MIRL (+++) (B)	Amaranthus powellii	3	Amaranthus powellii	2.65
Amaranthus powellii sub. Powellii TEXAS PI632241-2 MIRL (+++) (B)	Amaranthus powellii	3	Amaranthus powellii	2.57
Amaranthus powellii sub. Powellii TEXAS PI632241-3 MIRL (+++) (B)	Amaranthus powellii	3	Amaranthus powellii	2.66
Amaranthus powellii sub. Powellii NM PI649309-1 MIRL (+++) (B)	Amaranthus powellii	3	Amaranthus powellii	2.78
Amaranthus powellii sub. Powellii NM PI649309-2 MIRL (+++) (B)	Amaranthus powellii	3	Amaranthus powellii	2.51
Amaranthus powellii sub. Powellii NM PI649309-3 MIRL (+++) (B)	Amaranthus powellii	3	Amaranthus powellii	2.78
Amaranthus hybridus PUERTO RICO AMES 5152-1 MIRL (+++) (B)	Amaranthus hybridus	3	Amaranthus hybridus	2.73
Amaranthus hybridus PUERTO RICO AMES 5152-2 MIRL (+++) (B)	Amaranthus hybridus	3	Amaranthus hybridus	2.73
Amaranthus hybridus PUERTO RICO AMES 5152-3 MIRL (+++) (B)	Amaranthus hybridus	3	Amaranthus hybridus	2.69
Amaranthus hybridus INDIANA PI603895-1 MIRL (+++) (B)	Amaranthus hybridus	3	Amaranthus hybridus	2.58
Amaranthus hybridus INDIANA PI603895-2 MIRL (+++) (B)	Amaranthus hybridus	3	Amaranthus hybridus	2.43
	Amaranthus hybridus	3	Amaranthus hybridus	2.58

Amaranthus hybridus INDIANA PI603895-3 MIRL<br>**(+++) (B)**				
Amaranthus retroflexus ONTARIO AMES 35199-1 MIRL<br>**(+++) (A)**	Amaranthus retroflexus	3	Amaranthus retroflexus	2.68
Amaranthus retroflexus ONTARIO AMES 35199-2 MIRL<br>**(+++) (A)**	Amaranthus retroflexus	3	Amaranthus retroflexus	2.67
Amaranthus retroflexus ONTARIO AMES 35199-3 MIRL<br>**(+++) (A)**	Amaranthus retroflexus	3	Amaranthus retroflexus	2.64
Amaranthus retroflexus UTAH PI612857-1 MIRL<br>**(+++) (A)**	Amaranthus retroflexus	3	Amaranthus retroflexus	2.88
Amaranthus retroflexus UTAH PI612857-2 MIRL<br>**(+++) (A)**	Amaranthus retroflexus	3	Amaranthus retroflexus	2.88
Amaranthus retroflexus UTAH PI612857-3 MIRL<br>**(+++) (A)**	Amaranthus retroflexus	3	Amaranthus retroflexus	2.44
Amaranthus caudatus NJ PI553073-1 MIRL<br>**(+++) (B)**	Amaranthus caudatus	3	Amaranthus hybridus	2.06
Amaranthus caudatus NJ PI553073-2 MIRL<br>**(+++) (B)**	Amaranthus caudatus	3	Amaranthus caudatus	2.72
Amaranthus caudatus NJ PI553073-3 MIRL<br>**(+++) (B)**	Amaranthus caudatus	3	Amaranthus caudatus	2.72
Amaranthus caudatus CALIFORNIA PI690570-1 MIRL<br>**(+++) (B)**	Amaranthus caudatus	3	Amaranthus caudatus	2.76
Amaranthus caudatus CALIFORNIA PI690570-2 MIRL<br>**(+++) (B)**	Amaranthus caudatus	3	Amaranthus caudatus	2.76
Amaranthus caudatus CALIFORNIA PI690570-3 MIRL<br>**(+++) (B)**	Amaranthus caudatus	3	Amaranthus caudatus	2.76
	Amaranthus hypochondriacus	3	Amaranthus hypochondriacus	2.79

Amaranthus hypochondriacus MISSOURI PI698341-1 MIRL (+++) (B)				
Amaranthus hypochondriacus MISSOURI PI698341-2 MIRL (+++) (B)	Amaranthus hypochondriacus	3	Amaranthus hypochondriacus	2.89
Amaranthus hypochondriacus MISSOURI PI698341-3 MIRL (+++) (B)	Amaranthus hypochondriacus	3	Amaranthus hypochondriacus	2.89
Amaranthus hypochondriacus MEXICO SONORA PI599682-1 MIRL (+++) (B)	Amaranthus hypochondriacus	3	Amaranthus hypochondriacus	2.67
Amaranthus hypochondriacus MEXICO SONORA PI599682-2 MIRL (+++) (B)	Amaranthus hypochondriacus	3	Amaranthus hypochondriacus	2.72
Amaranthus hypochondriacus MEXICO SONORA PI599682-3 MIRL (+++) (B)	Amaranthus hypochondriacus	3	Amaranthus hypochondriacus	2.71
Amaranthus albus WASHINGTON PI654389-1 MIRL (+++) (A)	Amaranthus albus	3	Amaranthus albus	2.67
Amaranthus albus WASHINGTON PI654389-2 MIRL (+++) (A)	Amaranthus albus	3	Amaranthus albus	2.6
Amaranthus albus WASHINGTON PI654389-3 MIRL (+++) (A)	Amaranthus albus	3	Amaranthus albus	2.67
Amaranthus albus NC PI632244-1 MIRL (+++) (A)	Amaranthus albus	3	Amaranthus albus	2.66
Amaranthus albus NC PI632244-2 MIRL (+++) (A)	Amaranthus albus	3	Amaranthus albus	2.66
Amaranthus albus NC PI632244-3 MIRL (+++) (A)	Amaranthus albus	3	Amaranthus albus	2.61
Amaranthus arenicola KANSAS PI599671-1 MIRL (+++) (A)	Amaranthus arenicola	3	Amaranthus arenicola	1.86

Amaranthus arenicola KANSAS PI599671-2 MIRL **(+++) (A)**	Amaranthus arenicola	3	Amaranthus arenicola	2.28
Amaranthus arenicola KANSAS PI599671-3 MIRL **(+++) (A)**	Amaranthus arenicola	3	Amaranthus arenicola	2.27
Amaranthus arenicola TEXAS PI667168-1 MIRL **(+++) (B)**	Amaranthus arenicola	3	Amaranthus arenicola	2.25
Amaranthus arenicola TEXAS PI667168-2 MIRL **(+++) (B)**	Amaranthus arenicola	3	Amaranthus arenicola	2.48
Amaranthus arenicola TEXAS PI667168-3 MIRL **(+++) (B)**	Amaranthus arenicola	3	Amaranthus arenicola	2.49
Amaranthus californicus CALIFORNIA PI595319-1 MIRL **(+++) (A)**	Amaranthus californicus	3	Amaranthus californicus	2.54
Amaranthus californicus CALIFORNIA PI595319-2 MIRL **(+++) (A)**	Amaranthus californicus	3	Amaranthus californicus	2.54

Meaning of Score Values

Range	Description	Symbols	Color
**2.300 ... 3.000**	**highly probable species identification**	**(+++)**	green
**2.000 ... 2.299**	**secure genus identification, probable species identification**	**(++)**	green
**1.700 ... 1.999**	**probable genus identification**	**(+)**	yellow
**0.000 ... 1.699**	**not reliable identification**	**(-)**	red

Meaning of Consistency Categories (A - C)

Category	Description
**A**	**Species Consistency:** The best match was classified as 'green' (see above). Further 'green' matches are of the same species as the first one. Further 'yellow' matches are at least of the same genus as the first one.
**B**	**Genus Consistency:** The best match was classified as 'green' or 'yellow' (see above). Further 'green' or 'yellow' matches have at least the same genus as the first one. The conditions of species consistency are not fulfilled.
**C**	**No Consistency:** Neither species nor genus consistency (Please check for synonyms of names or microbial mixture).

Analyte1

Analyte Name:	Amaranthus palmeri MALI PI549158-1 MIRL
Analyte Description:	MSP
Analyte ID:	4403d2f9-9710-4f56-8807-3c16118d13e4
Analyte Creation Date/Time:	2022-05-10 10:35:16 AM
Applied MSP Library(ies):	
Applied Taxonomy Tree:	Bruker Taxonomy

	Matched Pattern		

Rank (Quality)		Score Value	NCBI Identifier
1 (+++)	Amaranthus palmeri MALI PI549158-1 MIRL	3	147656862
2 (+++)	Amaranthus palmeri MALI PI549158-2 MIRL	2.69	147656862
3 (+++)	Amaranthus palmeri MALI PI549158-3 MIRL	2.43	147656862
4 (+++)	Amaranthus palmeri ARIZONA PI686461-1 MIRL	2.38	147656862
5 (+++)	Amaranthus palmeri DAKAR PI633587-1 MIRL	2.36	147656862
6 (+++)	Amaranthus palmeri ARIZONA PI686461-2 MIRL	2.36	147656862
7 (+++)	Amaranthus palmeri DAKAR PI633586-1 MIRL	2.35	147656862
8 (+++)	Amaranthus palmeri MEXICO PUEBLA PI604557-3 MIRL	2.35	147656862
9 (+++)	Amaranthus palmeri MEXICO PUEBLA PI604557-2 MIRL	2.33	147656862
10 (+++)	Amaranthus palmeri DAKAR PI633586-3 MIRL	2.31	147656862
11 (+++)	Amaranthus watsonii MEXICO COLIMA PI633593-RE2 MIRL	2.3	147656862
12 (++)	Amaranthus palmeri ARIZONA PI686461-3 MIRL	2.28	147656862
13 (++)	Amaranthus palmeri DAKAR PI633586-2 MIRL	2.27	147656862
14 (++)	Amaranthus palmeri DAKAR PI633587-2 MIRL	2.27	147656862
15 (++)	Amaranthus palmeri DAKAR PI633587-3 MIRL	2.25	147656862
16 (++)	Amaranthus palmeri USA(AZ) PI632236-3 MIRL	2.16	147656862
17 (++)	Amaranthus palmeri USA(AZ) PI632236-2 MIRL	2.1	147656862
18 (++)	Amaranthus palmeri MEXICO PUEBLA PI604557-1 MIRL	2.01	147656862

19<br>(+)	Amaranthus watsonii MEXICO COLIMA PI633593-RE3 MIRL	1.92	147656862
20<br>(+)	Amaranthus palmeri MEXICO VERACRUZ PI667167-RE1 MIRL	1.88	147656862

Analyte2

Analyte Name:	Amaranthus palmeri MALI PI549158-2 MIRL
Analyte Description:	MSP
Analyte ID:	6aa67cba-caa9-4dab-b93f-b4b7a9c4f07f
Analyte Creation Date/Time:	2022-05-10 11:03:17 AM
Applied MSP Library(ies):	
Applied Taxonomy Tree:	Bruker Taxonomy

Rank (Quality)	Matched Pattern	Score Value	NCBI Identifier
1 (+++)	Amaranthus palmeri MALI PI549158-2 MIRL	3	147656862
2 (+++)	Amaranthus palmeri MALI PI549158-1 MIRL	2.69	147656862
3 (+++)	Amaranthus palmeri MALI PI549158-3 MIRL	2.41	147656862
4 (+++)	Amaranthus palmeri ARIZONA PI686461-2 MIRL	2.39	147656862
5 (+++)	Amaranthus palmeri DAKAR PI633586-3 MIRL	2.37	147656862
6 (+++)	Amaranthus palmeri DAKAR PI633586-1 MIRL	2.36	147656862
7 (+++)	Amaranthus watsonii MEXICO COLIMA PI633593-RE2 MIRL	2.36	147656862
8 (+++)	Amaranthus palmeri USA(AZ) PI632236-2 MIRL	2.34	147656862
9 (+++)	Amaranthus palmeri MEXICO PUEBLA PI604557-2 MIRL	2.33	147656862
10 (+++)	Amaranthus palmeri ARIZONA PI686461-1 MIRL	2.33	147656862
11 (+++)	Amaranthus palmeri DAKAR PI633587-1 MIRL	2.31	147656862

12<br>(++)	Amaranthus palmeri MEXICO PUEBLA PI604557-3 MIRL	2.28	147656862
13<br>(++)	Amaranthus palmeri DAKAR PI633587-3 MIRL	2.25	147656862
14<br>(++)	Amaranthus palmeri DAKAR PI633586-2 MIRL	2.25	147656862
15<br>(++)	Amaranthus palmeri ARIZONA PI686461-3 MIRL	2.18	147656862
16<br>(++)	Amaranthus palmeri USA(AZ) PI632236-3 MIRL	2.16	147656862
17<br>(++)	Amaranthus palmeri DAKAR PI633587-2 MIRL	2.1	147656862
18<br>(+)	Amaranthus watsonii MEXICO COLIMA PI633593-RE3 MIRL	1.99	147656862
19<br>(+)	Amaranthus spinosus NC PI632248-RE3 MIRL	1.98	147656862
20<br>(+)	Amaranthus watsonii MEXICO COLIMA PI633593-RE1 MIRL	1.98	147656862

Analyte3

Analyte Name:	Amaranthus palmeri MALI PI549158-3 MIRL
Analyte Description:	MSP
Analyte ID:	288c281b-118b-4e38-8d17-067fdd18035e
Analyte Creation Date/Time:	2022-05-10 11:20:11 AM
Applied MSP Library(ies):	
Applied Taxonomy Tree:	Bruker Taxonomy

Rank (Quality)	Matched Pattern	Score Value	NCBI Identifier
1 (+++)	Amaranthus palmeri MALI PI549158-3 MIRL	3	147656862
2 (+++)	Amaranthus palmeri MALI PI549158-1 MIRL	2.42	147656862
3 (+++)	Amaranthus palmeri MALI PI549158-2 MIRL	2.41	147656862
4 (+++)	Amaranthus palmeri DAKAR PI633586-1 MIRL	2.39	147656862
5 (+++)	Amaranthus palmeri ARIZONA PI686461-1 MIRL	2.33	147656862
6 (+++)	Amaranthus palmeri ARIZONA PI686461-2 MIRL	2.32	147656862
7 (++)	Amaranthus palmeri DAKAR PI633586-2 MIRL	2.29	147656862
8 (++)	Amaranthus palmeri DAKAR PI633586-3 MIRL	2.19	147656862
9 (++)	Amaranthus palmeri ARIZONA PI686461-3 MIRL	2.18	147656862
10 (++)	Amaranthus watsonii MEXICO COLIMA PI633593-RE2 MIRL	2.14	147656862
11 (++)	Amaranthus palmeri USA(AZ) PI632236-2 MIRL	2.14	147656862

12<br>(++)	Amaranthus palmeri MEXICO PUEBLA PI604557-3 MIRL	2.14	147656862
13<br>(++)	Amaranthus palmeri MEXICO PUEBLA PI604557-2 MIRL	2.13	147656862
14<br>(++)	Amaranthus palmeri DAKAR PI633587-3 MIRL	2.12	147656862
15<br>(++)	Amaranthus palmeri DAKAR PI633587-1 MIRL	2.1	147656862
16<br>(++)	Amaranthus palmeri USA(AZ) PI632236-3 MIRL	2.04	147656862
17<br>(++)	Amaranthus palmeri DAKAR PI633587-2 MIRL	2	147656862
18<br>(+)	Amaranthus palmeri MEXICO VERACRUZ PI667167-RE3 MIRL	1.86	147656862
19<br>(+)	Amaranthus palmeri MEXICO PUEBLA PI604557-1 MIRL	1.84	147656862
20<br>(+)	Amaranthus palmeri USA(AZ) PI632236-1 MIRL	1.83	147656862

Analyte4

Analyte Name:	Amaranthus palmeri USA(AZ) PI632236-1 MIRL
Analyte Description:	MSP
Analyte ID:	67e3d2ed-75f8-4100-a0a5-77d094e391e7
Analyte Creation Date/Time:	2022-05-11 10:39:48 AM
Applied MSP Library(ies):	
Applied Taxonomy Tree:	Bruker Taxonomy

Rank (Quality)	Matched Pattern	Score Value	NCBI Identifier
1 (+++)	Amaranthus palmeri USA(AZ) PI632236-1 MIRL	3	147656862
2 (++)	Amaranthus palmeri USA(AZ) PI632236-2 MIRL	2.09	147656862
3 (++)	Amaranthus palmeri USA(AZ) PI632236-3 MIRL	2.04	147656862
4 (++)	Amaranthus palmeri DAKAR PI633586-2 MIRL	2.02	147656862
5 (++)	Amaranthus palmeri DAKAR PI633586-1 MIRL	2	147656862
6 (+)	Amaranthus palmeri ARIZONA PI686461-3 MIRL	1.94	147656862
7 (+)	Amaranthus palmeri ARIZONA PI686461-2 MIRL	1.93	147656862
8 (+)	Amaranthus palmeri MEXICO PUEBLA PI604557-3 MIRL	1.85	147656862
9 (+)	Amaranthus palmeri MALI PI549158-1 MIRL	1.84	147656862
10 (+)	Amaranthus palmeri DAKAR PI633587-3 MIRL	1.83	147656862
11 (+)	Amaranthus palmeri MALI PI549158-3 MIRL	1.82	147656862

12<br>(+)	Amaranthus watsonii MEXICO COLIMA PI633593-RE2 MIRL	1.81	147656862
13<br>(+)	Amaranthus palmeri DAKAR PI633586-3 MIRL	1.8	147656862
14<br>(+)	Amaranthus palmeri ARIZONA PI686461-1 MIRL	1.78	147656862
15<br>(+)	Amaranthus palmeri MEXICO PUEBLA PI604557-2 MIRL	1.74	147656862
16<br>(+)	Amaranthus watsonii MEXICO COLIMA PI633593-RE3 MIRL	1.73	147656862
17<br>(+)	Amaranthus palmeri MALI PI549158-2 MIRL	1.71	147656862
18<br>(+)	Amaranthus palmeri MEXICO VERACRUZ PI667167-RE1 MIRL	1.71	147656862
19<br>(-)	Amaranthus palmeri DAKAR PI633587-1 MIRL	1.68	147656862
20<br>(-)	Amaranthus palmeri DAKAR PI633587-2 MIRL	1.65	147656862

Analyte5

Analyte Name:	Amaranthus palmeri USA(AZ) PI632236-2 MIRL
Analyte Description:	MSP
Analyte ID:	39ba6ff5-12e3-4ddd-8f6e-c331c4a2bf0b
Analyte Creation Date/Time:	2022-05-11 11:13:43 AM
Applied MSP Library(ies):	
Applied Taxonomy Tree:	Bruker Taxonomy

Rank (Quality)	Matched Pattern	Score Value	NCBI Identifier
1 (+++)	Amaranthus palmeri USA(AZ) PI632236-2 MIRL	3	147656862
2 (+++)	Amaranthus palmeri ARIZONA PI686461-2 MIRL	2.41	147656862
3 (+++)	Amaranthus palmeri DAKAR PI633586-2 MIRL	2.36	147656862
4 (+++)	Amaranthus palmeri ARIZONA PI686461-1 MIRL	2.34	147656862
5 (+++)	Amaranthus palmeri MALI PI549158-2 MIRL	2.33	147656862
6 (+++)	Amaranthus palmeri DAKAR PI633587-3 MIRL	2.33	147656862
7 (+++)	Amaranthus palmeri DAKAR PI633587-1 MIRL	2.3	147656862
8 (++)	Amaranthus palmeri DAKAR PI633586-3 MIRL	2.25	147656862
9 (++)	Amaranthus palmeri USA(AZ) PI632236-3 MIRL	2.24	147656862
10 (++)	Amaranthus palmeri DAKAR PI633587-2 MIRL	2.23	147656862
11 (++)	Amaranthus palmeri DAKAR PI633586-1 MIRL	2.22	147656862

12<br>(++)	Amaranthus palmeri ARIZONA PI686461-3 MIRL	2.22	147656862
13<br>(++)	Amaranthus palmeri MEXICO PUEBLA PI604557-3 MIRL	2.15	147656862
14<br>(++)	Amaranthus palmeri MALI PI549158-3 MIRL	2.14	147656862
15<br>(++)	Amaranthus palmeri MEXICO PUEBLA PI604557-2 MIRL	2.13	147656862
16<br>(++)	Amaranthus palmeri MALI PI549158-1 MIRL	2.1	147656862
17<br>(++)	Amaranthus palmeri USA(AZ) PI632236-1 MIRL	2.1	147656862
18<br>(++)	Amaranthus watsonii MEXICO COLIMA PI633593-RE2 MIRL	2.1	147656862
19<br>(+)	Amaranthus palmeri MEXICO VERACRUZ PI667167-RE1 MIRL	1.97	147656862
20<br>(+)	Amaranthus watsonii MEXICO COLIMA PI633593-RE3 MIRL	1.92	147656862

Analyte6

Analyte Name:	Amaranthus palmeri USA(AZ) PI632236-3 MIRL
Analyte Description:	MSP
Analyte ID:	2374e216-26a8-45ba-8fd3-c3fdf4e5ce8f
Analyte Creation Date/Time:	2022-06-22 4:19:17 PM
Applied MSP Library(ies):	
Applied Taxonomy Tree:	Bruker Taxonomy

Rank (Quality)	Matched Pattern	Score Value	NCBI Identifier
1 (+++)	Amaranthus palmeri USA(AZ) PI632236-3 MIRL	3	147656862
2 (++)	Amaranthus palmeri ARIZONA PI686461-2 MIRL	2.26	147656862
3 (++)	Amaranthus palmeri USA(AZ) PI632236-2 MIRL	2.24	147656862
4 (++)	Amaranthus palmeri DAKAR PI633586-2 MIRL	2.21	147656862
5 (++)	Amaranthus palmeri DAKAR PI633586-3 MIRL	2.19	147656862
6 (++)	Amaranthus palmeri MALI PI549158-2 MIRL	2.16	147656862
7 (++)	Amaranthus palmeri MALI PI549158-1 MIRL	2.16	147656862
8 (++)	Amaranthus palmeri ARIZONA PI686461-1 MIRL	2.14	147656862
9 (++)	Amaranthus palmeri DAKAR PI633587-2 MIRL	2.11	147656862
10 (++)	Amaranthus palmeri MEXICO PUEBLA PI604557-2 MIRL	2.08	147656862
11 (++)	Amaranthus palmeri DAKAR PI633586-1 MIRL	2.08	147656862

12<br>(++)	Amaranthus palmeri DAKAR PI633587-1 MIRL	2.07	147656862
13<br>(++)	Amaranthus palmeri ARIZONA PI686461-3 MIRL	2.05	147656862
14<br>(++)	Amaranthus palmeri USA(AZ) PI632236-1 MIRL	2.04	147656862
15<br>(++)	Amaranthus palmeri MALI PI549158-3 MIRL	2.04	147656862
16<br>(++)	Amaranthus palmeri DAKAR PI633587-3 MIRL	2.03	147656862
17<br>(++)	Amaranthus palmeri MEXICO PUEBLA PI604557-3 MIRL	2.02	147656862
18<br>(+)	Amaranthus watsonii MEXICO COLIMA PI633593-RE2 MIRL	1.94	147656862
19<br>(+)	Amaranthus palmeri MEXICO PUEBLA PI604557-1 MIRL	1.92	147656862
20<br>(+)	Amaranthus palmeri MEXICO VERACRUZ PI667167-RE1 MIRL	1.92	147656862

Analyte7

Analyte Name:	Amaranthus tuberculatus IOWA PI 553086-1 MIRL
Analyte Description:	MSP
Analyte ID:	8d15f2e6-1eb5-43a2-bc4f-679e913a17fd
Analyte Creation Date/Time:	2022-06-22 4:52:13 PM
Applied MSP Library(ies):	
Applied Taxonomy Tree:	Bruker Taxonomy

Rank (Quality)	Matched Pattern	Score Value	NCBI Identifier
1 (+++)	Amaranthus tuberculatus IOWA PI 553086-1 MIRL	3	147656862
2 (+++)	Amaranthus tuberculatus IOWA PI 553086-2 MIRL	2.41	147656862
3 (+++)	Amaranthus tuberculatus IOWA PI 553086-3 MIRL	2.3	147656862
4 (++)	Amaranthus tuberculatus KANSAS PI 60743-3 MIRL	2.07	147656862
5 (+)	Amaranthus arenicola TEXAS PI667168-1 MIRL	1.9	147656862
6 (+)	Amaranthus rudis HARROW 1 MIRL	1.83	147656862
7 (+)	Amaranthus rudis HARROW 3 MIRL	1.81	147656862
8 (+)	Amaranthus tuberculatus IOWA PI674264-2 MIRL	1.79	147656862
9 (+)	Amaranthus tuberculatus KANSAS PI 60743-1 MIRL	1.78	147656862
10 (+)	Amaranthus rudis HARROW 2 MIRL	1.76	147656862
11 (+)	Amaranthus arenicola TEXAS PI667168-3 MIRL	1.72	147656862

12<br>**(-)**	Amaranthus tuberculatus IOWA PI604247-2 MIRL	1.65	147656862
13<br>**(-)**	Amaranthus tuberculatus IOWA PI674264-1 MIRL	1.63	147656862
14<br>**(-)**	Amaranthus arenicola TEXAS PI667168-2 MIRL	1.55	147656862
15<br>**(-)**	Amaranthus tuberculatus KANSAS PI 60743-2 MIRL	1.54	147656862
16<br>**(-)**	Amaranthus tuberculatus IOWA PI604247-3 MIRL	1.39	147656862
17<br>**(-)**	Amaranthus arenicola PI 599670-1 MIRL	1.15	147656862
18<br>**(-)**	Amaranthus tuberculatus IOWA PI674264-3 MIRL	1.08	147656862
19<br>**(-)**	Amaranthus blitoides CANADA PI608663-2 MIRL	1.03	147656862
20<br>**(-)**	Amaranthus spinosus PI 632248-1 MIRL	1.02	147656862

Analyte8

Analyte Name:	Amaranthus tuberculatus IOWA PI 553086-2 MIRL
Analyte Description:	MSP
Analyte ID:	a80dca87-da7f-4c35-aa44-3fb69f0a1f06
Analyte Creation Date/Time:	2022-06-22 5:12:37 PM
Applied MSP Library(ies):	
Applied Taxonomy Tree:	Bruker Taxonomy

Rank (Quality)	Matched Pattern	Score Value	NCBI Identifier
1 (+++)	Amaranthus tuberculatus IOWA PI 553086-2 MIRL	3	147656862
2 (+++)	Amaranthus tuberculatus IOWA PI 553086-1 MIRL	2.39	147656862
3 (+++)	Amaranthus tuberculatus IOWA PI 553086-3 MIRL	2.32	147656862
4 (++)	Amaranthus tuberculatus KANSAS PI 60743-3 MIRL	2.08	147656862
5 (+)	Amaranthus tuberculatus KANSAS PI 60743-2 MIRL	1.81	147656862
6 (+)	Amaranthus tuberculatus KANSAS PI 60743-1 MIRL	1.73	147656862
7 (-)	Amaranthus tuberculatus IOWA PI674264-2 MIRL	1.67	147656862
8 (-)	Amaranthus tuberculatus IOWA PI674264-1 MIRL	1.66	147656862
9 (-)	Amaranthus arenicola TEXAS PI667168-1 MIRL	1.62	147656862
10 (-)	Amaranthus rudis HARROW 3 MIRL	1.62	147656862
11 (-)	Amaranthus arenicola TEXAS PI667168-3 MIRL	1.55	147656862

12<br>**(-)**	Amaranthus rudis HARROW 1 MIRL	1.51	147656862
13<br>**(-)**	Amaranthus arenicola TEXAS PI667168-2 MIRL	1.5	147656862
14<br>**(-)**	Amaranthus spinosus PI 632248-3 MIRL	1.36	147656862
15<br>**(-)**	Amaranthus rudis HARROW 2 MIRL	1.35	147656862
16<br>**(-)**	Amaranthus tuberculatus IOWA PI604247-2 MIRL	1.26	147656862
17<br>**(-)**	Amaranthus spinosus PI 632248-1 MIRL	1.17	147656862
18<br>**(-)**	Amaranthus tuberculatus IOWA PI604247-3 MIRL	1.16	147656862
19<br>**(-)**	Amaranthus spinosus PI 632248-2 MIRL	1.12	147656862
20<br>**(-)**	Amaranthus albus NC PI632244-1 MIRL	1.11	147656862

Analyte9

Analyte Name:	Amaranthus spinosus PI 632248-1 MIRL
Analyte Description:	MSP
Analyte ID:	fcecd553-f8f3-48f8-bd36-7dec89a8a8f2
Analyte Creation Date/Time:	2022-06-27 4:07:31 PM
Applied MSP Library(ies):	
Applied Taxonomy Tree:	Bruker Taxonomy

Rank (Quality)	Matched Pattern	Score Value	NCBI Identifier
1 (+++)	Amaranthus spinosus PI 632248-1 MIRL	3	147656862
2 (+++)	Amaranthus spinosus PI 632248-3 MIRL	2.69	147656862
3 (+++)	Amaranthus spinosus PI 632248-2 MIRL	2.62	147656862
4 (++)	Amaranthus palmeri MEXICO VERACRUZ PI667167-2 MIRL	2.08	147656862
5 (++)	Amaranthus watsonii PI 633593-1 MIRL	2.08	147656862
6 (+)	Amaranthus watsonii PI 633593-2 MIRL	1.81	147656862
7 (-)	Amaranthus watsonii PI 633593-3 MIRL	1.68	147656862
8 (-)	Amaranthus palmeri MEXICO VERACRUZ PI667167-3 MIRL	1.57	147656862
9 (-)	Amaranthus tuberculatus IOWA PI 553086-3 MIRL	1.39	147656862
10 (-)	Amaranthus tricolor NJ AMES 5303-2 MIRL	1.33	147656862
11 (-)	Amaranthus retroflexus UTAH PI612857-2 MIRL	1.31	147656862

12<br>(-)	Amaranthus tuberculatus IOWA PI 553086-2 MIRL	1.18	147656862
13<br>(-)	Amaranthus tricolor NJ AMES 5303-1 MIRL	1.15	147656862
14<br>(-)	Amaranthus tricolor NJ AMES 5303-3 MIRL	1.15	147656862
15<br>(-)	Amaranthus retroflexus UTAH PI612857-1 MIRL	1.14	147656862
16<br>(-)	Amaranthus palmeri MEXICO VERACRUZ PI667167-RE2 MIRL	1.1	147656862
17<br>(-)	Amaranthus tuberculatus IOWA PI674264-1 MIRL	1.09	147656862
18<br>(-)	Amaranthus spinosus HARROW 3 MIRL	1.07	147656862
19<br>(-)	Amaranthus spinosus HARROW 2 MIRL	1.05	147656862
20<br>(-)	Amaranthus spinosus HARROW 1 MIRL	1.02	147656862

Analyte10

Analyte Name:	Amaranthus spinosus PI 632248-3 MIRL
Analyte Description:	MSP
Analyte ID:	eabc3ea4-8d3d-4333-b6f2-a9323efaa7f2
Analyte Creation Date/Time:	2022-06-27 4:47:05 PM
Applied MSP Library(ies):	
Applied Taxonomy Tree:	Bruker Taxonomy

Rank (Quality)	Matched Pattern	Score Value	NCBI Identifier
1 (+++)	Amaranthus spinosus PI 632248-3 MIRL	3	147656862
2 (+++)	Amaranthus spinosus PI 632248-1 MIRL	2.69	147656862
3 (+++)	Amaranthus spinosus PI 632248-2 MIRL	2.57	147656862
4 (++)	Amaranthus watsonii PI 633593-1 MIRL	2.17	147656862
5 (+)	Amaranthus palmeri MEXICO VERACRUZ PI667167-2 MIRL	1.94	147656862
6 (-)	Amaranthus palmeri MEXICO VERACRUZ PI667167-3 MIRL	1.64	147656862
7 (-)	Amaranthus watsonii PI 633593-3 MIRL	1.63	147656862
8 (-)	Amaranthus watsonii PI 633593-2 MIRL	1.56	147656862
9 (-)	Amaranthus spinosus HARROW 2 MIRL	1.51	147656862
10 (-)	Amaranthus spinosus HARROW 1 MIRL	1.45	147656862
11 (-)	Amaranthus spinosus HARROW 3 MIRL	1.44	147656862

12<br>(-)	Amaranthus tuberculatus IOWA PI 553086-2 MIRL	1.4	147656862
13<br>(-)	Amaranthus tuberculatus IOWA PI 553086-3 MIRL	1.38	147656862
14<br>(-)	Amaranthus retroflexus UTAH PI612857-2 MIRL	1.21	147656862
15<br>(-)	Amaranthus spinosus NC PI632248-RE1 MIRL	1.2	147656862
16<br>(-)	Amaranthus palmeri ARIZONA PI686461-3 MIRL	1.16	147656862
17<br>(-)	Amaranthus spinosus NC PI632248-RE3 MIRL	1.12	147656862
18<br>(-)	Amaranthus tricolor NJ AMES 5303-2 MIRL	1.12	147656862
19<br>(-)	Amaranthus retroflexus UTAH PI612857-1 MIRL	1.08	147656862
20<br>(-)	Amaranthus arenicola PI 599670-2 MIRL	1.06	147656862

Analyte11

Analyte Name: Amaranthus spinosus PI 632248-2 MIRL
Analyte Description: MSP
Analyte ID: 9a183b18-b53c-4ab6-bf8a-76fbb48b538f
Analyte Creation Date/Time: 2022-06-27 5:07:18 PM
Applied MSP Library(ies):
Applied Taxonomy Tree: Bruker Taxonomy

Rank (Quality)	Matched Pattern	Score Value	NCBI Identifier
1 (+++)	Amaranthus spinosus PI 632248-2 MIRL	3	147656862
2 (+++)	Amaranthus spinosus PI 632248-1 MIRL	2.62	147656862
3 (+++)	Amaranthus spinosus PI 632248-3 MIRL	2.58	147656862
4 (++)	Amaranthus watsonii PI 633593-1 MIRL	2.22	147656862
5 (++)	Amaranthus palmeri MEXICO VERACRUZ PI667167-2 MIRL	2.07	147656862
6 (-)	Amaranthus watsonii PI 633593-3 MIRL	1.62	147656862
7 (-)	Amaranthus watsonii PI 633593-2 MIRL	1.59	147656862
8 (-)	Amaranthus palmeri MEXICO VERACRUZ PI667167-3 MIRL	1.47	147656862
9 (-)	Amaranthus tuberculatus IOWA PI 553086-3 MIRL	1.32	147656862
10 (-)	Amaranthus spinosus HARROW 2 MIRL	1.27	147656862
11 (-)	Amaranthus spinosus HARROW 1 MIRL	1.21	147656862

12<br>(-)	Amaranthus spinosus HARROW 3 MIRL	1.15	147656862
13<br>(-)	Amaranthus tuberculatus IOWA PI 553086-2 MIRL	1.13	147656862
14<br>(-)	Amaranthus retroflexus UTAH PI612857-2 MIRL	1.11	147656862
15<br>(-)	Amaranthus retroflexus UTAH PI612857-1 MIRL	1.07	147656862
16<br>(-)	Amaranthus tuberculatus IOWA PI674264-1 MIRL	1.05	147656862
17<br>(-)	Amaranthus spinosus NC PI632248-RE1 MIRL	0.97	147656862
18<br>(-)	Amaranthus arenicola PI 599670-3 MIRL	0.96	147656862
19<br>(-)	Amaranthus palmeri MEXICO PUEBLA PI604557-3 MIRL	0.96	147656862
20<br>(-)	Amaranthus tuberculatus IOWA PI674264-2 MIRL	0.94	147656862

Analyte12

Analyte Name:	Amaranthus tuberculatus IOWA PI 553086-3 MIRL
Analyte Description:	MSP
Analyte ID:	62dd7e50-ea3f-40e2-af39-4e18223cbeba
Analyte Creation Date/Time:	2022-06-28 12:49:43 PM
Applied MSP Library(ies):	
Applied Taxonomy Tree:	Bruker Taxonomy

Rank (Quality)	Matched Pattern	Score Value	NCBI Identifier
1 (+++)	Amaranthus tuberculatus IOWA PI 553086-3 MIRL	3	147656862
2 (+++)	Amaranthus tuberculatus IOWA PI 553086-2 MIRL	2.32	147656862
3 (+++)	Amaranthus tuberculatus IOWA PI 553086-1 MIRL	2.31	147656862
4 (++)	Amaranthus tuberculatus KANSAS PI 60743-3 MIRL	2.17	147656862
5 (+)	Amaranthus arenicola TEXAS PI667168-1 MIRL	1.96	147656862
6 (+)	Amaranthus arenicola TEXAS PI667168-3 MIRL	1.92	147656862
7 (+)	Amaranthus arenicola TEXAS PI667168-2 MIRL	1.82	147656862
8 (+)	Amaranthus tuberculatus KANSAS PI 60743-1 MIRL	1.78	147656862
9 (+)	Amaranthus tuberculatus IOWA PI604247-3 MIRL	1.77	147656862
10 (+)	Amaranthus tuberculatus IOWA PI674264-1 MIRL	1.71	147656862
11 (-)	Amaranthus rudis HARROW 3 MIRL	1.68	147656862

12<br>**(-)**	Amaranthus tuberculatus KANSAS PI 60743-2 MIRL	1.68	147656862
13<br>**(-)**	Amaranthus rudis HARROW 1 MIRL	1.58	147656862
14<br>**(-)**	Amaranthus tuberculatus IOWA PI674264-2 MIRL	1.56	147656862
15<br>**(-)**	Amaranthus tuberculatus IOWA PI604247-2 MIRL	1.52	147656862
16<br>**(-)**	Amaranthus rudis HARROW 2 MIRL	1.51	147656862
17<br>**(-)**	Amaranthus spinosus PI 632248-3 MIRL	1.38	147656862
18<br>**(-)**	Amaranthus tuberculatus IOWA PI674264-3 MIRL	1.37	147656862
19<br>**(-)**	Amaranthus tuberculatus IOWA PI604247-1 MIRL	1.37	147656862
20<br>**(-)**	Amaranthus spinosus PI 632248-1 MIRL	1.34	147656862

Analyte13

Analyte Name:	Amaranthus tuberculatus KANSAS PI 60743-1 MIRL
Analyte Description:	MSP
Analyte ID:	9c8cf440-a269-43d0-9f7a-6d1ec0de2c7a
Analyte Creation Date/Time:	2022-06-28 1:17:37 PM
Applied MSP Library(ies):	
Applied Taxonomy Tree:	Bruker Taxonomy

Rank (Quality)	Matched Pattern	Score Value	NCBI Identifier
1 (+++)	Amaranthus tuberculatus KANSAS PI 60743-1 MIRL	3	147656862
2 (++)	Amaranthus tuberculatus KANSAS PI 60743-3 MIRL	2.23	147656862
3 (++)	Amaranthus tuberculatus KANSAS PI 60743-2 MIRL	2.12	147656862
4 (++)	Amaranthus arenicola TEXAS PI667168-2 MIRL	2	147656862
5 (+)	Amaranthus arenicola TEXAS PI667168-1 MIRL	1.89	147656862
6 (+)	Amaranthus rudis HARROW 2 MIRL	1.84	147656862
7 (+)	Amaranthus tuberculatus IOWA PI 553086-3 MIRL	1.79	147656862
8 (+)	Amaranthus tuberculatus IOWA PI604247-2 MIRL	1.76	147656862
9 (+)	Amaranthus tuberculatus IOWA PI 553086-1 MIRL	1.76	147656862
10 (+)	Amaranthus rudis HARROW 3 MIRL	1.74	147656862
11 (+)	Amaranthus tuberculatus IOWA PI 553086-2 MIRL	1.71	147656862

12<br>**(-)**	Amaranthus arenicola TEXAS PI667168-3 MIRL	1.69	147656862
13<br>**(-)**	Amaranthus tuberculatus IOWA PI604247-1 MIRL	1.63	147656862
14<br>**(-)**	Amaranthus rudis HARROW 1 MIRL	1.53	147656862
15<br>**(-)**	Amaranthus tuberculatus IOWA PI674264-1 MIRL	1.49	147656862
16<br>**(-)**	Amaranthus tuberculatus IOWA PI604247-3 MIRL	1.49	147656862
17<br>**(-)**	Amaranthus tuberculatus IOWA PI674264-2 MIRL	1.4	147656862
18<br>**(-)**	Amaranthus arenicola PI 599670-2 MIRL	1.22	147656862
19<br>**(-)**	Amaranthus tuberculatus IOWA PI674264-3 MIRL	1.21	147656862
20<br>**(-)**	Amaranthus albus WASHINGTON PI654389-3 MIRL	1.1	147656862

Analyte14

Analyte Name:	Amaranthus tuberculatus KANSAS PI 60743-2 MIRL
Analyte Description:	MSP
Analyte ID:	9e4b23f8-56b0-4bd9-a610-b82bc73a34f9
Analyte Creation Date/Time:	2022-06-28 2:00:40 PM
Applied MSP Library(ies):	
Applied Taxonomy Tree:	Bruker Taxonomy

Rank (Quality)	Matched Pattern	Score Value	NCBI Identifier
1 (+++)	Amaranthus tuberculatus KANSAS PI 60743-2 MIRL	3	147656862
2 (++)	Amaranthus tuberculatus KANSAS PI 60743-1 MIRL	2.14	147656862
3 (++)	Amaranthus tuberculatus KANSAS PI 60743-3 MIRL	2.01	147656862
4 (+)	Amaranthus arenicola TEXAS PI667168-1 MIRL	1.97	147656862
5 (+)	Amaranthus arenicola TEXAS PI667168-3 MIRL	1.94	147656862
6 (+)	Amaranthus arenicola TEXAS PI667168-2 MIRL	1.9	147656862
7 (+)	Amaranthus rudis HARROW 3 MIRL	1.82	147656862
8 (+)	Amaranthus tuberculatus IOWA PI 553086-2 MIRL	1.81	147656862
9 (+)	Amaranthus rudis HARROW 2 MIRL	1.81	147656862
10 (+)	Amaranthus tuberculatus IOWA PI674264-1 MIRL	1.75	147656862
11 (-)	Amaranthus tuberculatus IOWA PI604247-2 MIRL	1.68	147656862

12<br>**(-)**	Amaranthus tuberculatus IOWA PI674264-2 MIRL	1.65	147656862
13<br>**(-)**	Amaranthus tuberculatus IOWA PI 553086-3 MIRL	1.64	147656862
14<br>**(-)**	Amaranthus tuberculatus IOWA PI 553086-1 MIRL	1.58	147656862
15<br>**(-)**	Amaranthus tuberculatus IOWA PI604247-1 MIRL	1.52	147656862
16<br>**(-)**	Amaranthus rudis HARROW 1 MIRL	1.49	147656862
17<br>**(-)**	Amaranthus tuberculatus IOWA PI674264-3 MIRL	1.43	147656862
18<br>**(-)**	Amaranthus albus NC PI632244-2 MIRL	1.39	147656862
19<br>**(-)**	Amaranthus tuberculatus IOWA PI604247-3 MIRL	1.38	147656862
20<br>**(-)**	Amaranthus albus NC PI632244-1 MIRL	1.25	147656862

Analyte15

Analyte Name:	Amaranthus tuberculatus KANSAS PI 60743-3 MIRL
Analyte Description:	MSP
Analyte ID:	c2e394e7-7ede-480b-9dff-8e946e0bd7d1
Analyte Creation Date/Time:	2022-06-28 2:20:15 PM
Applied MSP Library(ies):	
Applied Taxonomy Tree:	Bruker Taxonomy

Rank (Quality)	Matched Pattern	Score Value	NCBI Identifier
1 (+++)	Amaranthus tuberculatus KANSAS PI 60743-3 MIRL	3	147656862
2 (++)	Amaranthus tuberculatus KANSAS PI 60743-1 MIRL	2.21	147656862
3 (++)	Amaranthus tuberculatus IOWA PI 553086-3 MIRL	2.14	147656862
4 (++)	Amaranthus arenicola TEXAS PI667168-3 MIRL	2.12	147656862
5 (++)	Amaranthus tuberculatus IOWA PI 553086-1 MIRL	2.07	147656862
6 (++)	Amaranthus tuberculatus IOWA PI 553086-2 MIRL	2.07	147656862
7 (++)	Amaranthus arenicola TEXAS PI667168-1 MIRL	2.05	147656862
8 (++)	Amaranthus arenicola TEXAS PI667168-2 MIRL	2.04	147656862
9 (+)	Amaranthus tuberculatus KANSAS PI 60743-2 MIRL	1.97	147656862
10 (+)	Amaranthus rudis HARROW 1 MIRL	1.9	147656862
11 (+)	Amaranthus tuberculatus IOWA PI604247-2 MIRL	1.82	147656862

12<br>(+)	Amaranthus rudis HARROW 2 MIRL	1.81	147656862
13<br>(+)	Amaranthus tuberculatus IOWA PI674264-1 MIRL	1.71	147656862
14<br>(-)	Amaranthus tuberculatus IOWA PI604247-3 MIRL	1.64	147656862
15<br>(-)	Amaranthus rudis HARROW 3 MIRL	1.62	147656862
16<br>(-)	Amaranthus tuberculatus IOWA PI674264-2 MIRL	1.61	147656862
17<br>(-)	Amaranthus tuberculatus IOWA PI674264-3 MIRL	1.59	147656862
18<br>(-)	Amaranthus tuberculatus IOWA PI604247-1 MIRL	1.47	147656862
19<br>(-)	Amaranthus arenicola KANSAS PI599671-1 MIRL	1.17	147656862
20<br>(-)	Amaranthus arenicola PI 599670-1 MIRL	1.07	147656862

Analyte16

Analyte Name:	Amaranthus arenicola PI 599670-1 MIRL
Analyte Description:	MSP
Analyte ID:	5ec23b2d-9192-4013-9c90-a6faeeffa309
Analyte Creation Date/Time:	2022-06-28 3:24:41 PM
Applied MSP Library(ies):	
Applied Taxonomy Tree:	Bruker Taxonomy

Rank (Quality)	Matched Pattern	Score Value	NCBI Identifier
1 (+++)	Amaranthus arenicola PI 599670-1 MIRL	3	147656862
2 (++)	Amaranthus arenicola KANSAS PI599671-3 MIRL	2.22	147656862
3 (++)	Amaranthus arenicola PI 599670-2 MIRL	2.14	147656862
4 (++)	Amaranthus arenicola PI 599670-3 MIRL	2.1	147656862
5 (+)	Amaranthus arenicola KANSAS PI599671-2 MIRL	1.9	147656862
6 (-)	Amaranthus arenicola KANSAS PI599671-1 MIRL	1.64	147656862
7 (-)	Amaranthus watsonii PI 633593-3 MIRL	1.31	147656862
8 (-)	Amaranthus watsonii PI 633593-2 MIRL	1.29	147656862
9 (-)	Amaranthus tuberculatus IOWA PI 553086-3 MIRL	1.28	147656862
10 (-)	Amaranthus tuberculatus IOWA PI 553086-1 MIRL	1.14	147656862
11 (-)	Amaranthus watsonii PI 633593-1 MIRL	1.11	147656862

12<br>(-)	Amaranthus tuberculatus KANSAS PI 60743-3 MIRL	1.11	147656862
13<br>(-)	Amaranthus retroflexus HARROW 1 MIRL	1.05	147656862
14<br>(-)	Amaranthus retroflexus CANADA AMES 5328-3 MIRL	1.04	147656862
15<br>(-)	Amaranthus palmeri MEXICO PUEBLA PI604557-1 MIRL	1.02	147656862
16<br>(-)	Amaranthus spinosus PI 632248-3 MIRL	0.98	147656862
17<br>(-)	Amaranthus californicus CALIFORNIA PI595319-1 MIRL	0.98	147656862
18<br>(-)	Amaranthus palmeri MEXICO VERACRUZ PI667167-1 MIRL	0.97	147656862
19<br>(-)	Amaranthus spinosus HARROW 1 MIRL	0.97	147656862
20<br>(-)	Amaranthus rudis HARROW 1 MIRL	0.91	147656862

Analyte17

Analyte Name:	Amaranthus arenicola PI 599670-2 MIRL
Analyte Description:	MSP
Analyte ID:	ccf8d85d-093f-4c09-ba50-dc4c7dbbd343
Analyte Creation Date/Time:	2022-06-28 3:52:51 PM
Applied MSP Library(ies):	
Applied Taxonomy Tree:	Bruker Taxonomy

Rank (Quality)	Matched Pattern	Score Value	NCBI Identifier
1 (+++)	Amaranthus arenicola PI 599670-2 MIRL	3	147656862
2 (++)	Amaranthus arenicola PI 599670-1 MIRL	2.12	147656862
3 (+)	Amaranthus arenicola PI 599670-3 MIRL	1.99	147656862
4 (-)	Amaranthus arenicola KANSAS PI599671-3 MIRL	1.67	147656862
5 (-)	Amaranthus arenicola KANSAS PI599671-2 MIRL	1.56	147656862
6 (-)	Amaranthus watsonii PI 633593-2 MIRL	1.47	147656862
7 (-)	Amaranthus palmeri MEXICO VERACRUZ PI667167-3 MIRL	1.27	147656862
8 (-)	Amaranthus tuberculatus KANSAS PI 60743-2 MIRL	1.21	147656862
9 (-)	Amaranthus palmeri MEXICO VERACRUZ PI667167-1 MIRL	1.18	147656862
10 (-)	Amaranthus watsonii PI 633593-3 MIRL	1.18	147656862
11 (-)	Amaranthus tuberculatus KANSAS PI 60743-1 MIRL	1.13	147656862

12<br>(-)	Amaranthus arenicola KANSAS PI599671-1 MIRL	1.09	147656862
13<br>(-)	Amaranthus rudis HARROW 3 MIRL	1.06	147656862
14<br>(-)	Amaranthus spinosus PI 632248-3 MIRL	1.05	147656862
15<br>(-)	Amaranthus spinosus HARROW 1 MIRL	1.03	147656862
16<br>(-)	Amaranthus tuberculatus IOWA PI 553086-1 MIRL	1	147656862
17<br>(-)	Amaranthus tuberculatus KANSAS PI 60743-3 MIRL	1	147656862
18<br>(-)	Amaranthus tuberculatus IOWA PI 553086-3 MIRL	0.99	147656862
19<br>(-)	Amaranthus palmeri MEXICO PUEBLA PI604557-1 MIRL	0.95	147656862
20<br>(-)	Amaranthus powellii sub. Powellii TEXAS PI632241-3 MIRL	0.95	147656862

Analyte18

Analyte Name:	Amaranthus arenicola PI 599670-3 MIRL
Analyte Description:	MSP
Analyte ID:	633c6250-5c2a-47b3-8f11-0d3055d566a8
Analyte Creation Date/Time:	2022-06-28 4:18:15 PM
Applied MSP Library(ies):	
Applied Taxonomy Tree:	Bruker Taxonomy

Rank (Quality)	Matched Pattern	Score Value	NCBI Identifier
1 (+++)	Amaranthus arenicola PI 599670-3 MIRL	3	147656862
2 (++)	Amaranthus arenicola PI 599670-1 MIRL	2.08	147656862
3 (+)	Amaranthus arenicola PI 599670-2 MIRL	1.98	147656862
4 (+)	Amaranthus arenicola KANSAS PI599671-3 MIRL	1.76	147656862
5 (-)	Amaranthus arenicola KANSAS PI599671-2 MIRL	1.6	147656862
6 (-)	Amaranthus arenicola KANSAS PI599671-1 MIRL	1.49	147656862
7 (-)	Amaranthus watsonii PI 633593-1 MIRL	1.17	147656862
8 (-)	Amaranthus palmeri DAKAR PI633587-1 MIRL	1.15	147656862
9 (-)	Amaranthus tuberculatus IOWA PI 553086-3 MIRL	1.05	147656862
10 (-)	Amaranthus palmeri ARIZONA PI686461-1 MIRL	1.05	147656862
11 (-)	Amaranthus watsonii PI 633593-2 MIRL	1.01	147656862

12<br>(-)	Amaranthus palmeri DAKAR PI633586-3 MIRL	0.96	147656862
13<br>(-)	Amaranthus palmeri MEXICO VERACRUZ PI667167-1 MIRL	0.96	147656862
14<br>(-)	Amaranthus spinosus PI 632248-2 MIRL	0.96	147656862
15<br>(-)	Amaranthus arenicola TEXAS PI667168-3 MIRL	0.95	147656862
16<br>(-)	Amaranthus spinosus PI 632248-3 MIRL	0.95	147656862
17<br>(-)	Amaranthus palmeri DAKAR PI633587-3 MIRL	0.94	147656862
18<br>(-)	Amaranthus blitoides HARROW 3 MIRL	0.94	147656862
19<br>(-)	Amaranthus watsonii MEXICO COLIMA PI633593-RE1 MIRL	0.93	147656862
20<br>(-)	Amaranthus palmeri MEXICO VERACRUZ PI667167-3 MIRL	0.92	147656862

Analyte19

Analyte Name:	Amaranthus watsonii PI 633593-1 MIRL
Analyte Description:	MSP
Analyte ID:	7d01c253-1692-467d-a6ea-49717471af4d
Analyte Creation Date/Time:	2022-06-30 11:02:15 AM
Applied MSP Library(ies):	
Applied Taxonomy Tree:	Bruker Taxonomy

Rank (Quality)	Matched Pattern	Score Value	NCBI Identifier
1 (+++)	Amaranthus watsonii PI 633593-1 MIRL	3	147656862
2 (++)	Amaranthus spinosus PI 632248-2 MIRL	2.21	147656862
3 (++)	Amaranthus spinosus PI 632248-3 MIRL	2.17	147656862
4 (++)	Amaranthus watsonii PI 633593-2 MIRL	2.12	147656862
5 (++)	Amaranthus spinosus PI 632248-1 MIRL	2.09	147656862
6 (++)	Amaranthus watsonii PI 633593-3 MIRL	2.04	147656862
7 (+)	Amaranthus palmeri MEXICO VERACRUZ PI667167-2 MIRL	1.94	147656862
8 (-)	Amaranthus spinosus HARROW 2 MIRL	1.32	147656862
9 (-)	Amaranthus arenicola PI 599670-3 MIRL	1.26	147656862
10 (-)	Amaranthus palmeri MEXICO VERACRUZ PI667167-3 MIRL	1.24	147656862
11 (-)	Amaranthus spinosus HARROW 3 MIRL	1.15	147656862

12<br>(-)	Amaranthus arenicola PI 599670-1 MIRL	1.14	147656862
13<br>(-)	Amaranthus caudatus NJ PI553073-2 MIRL	1.08	147656862
14<br>(-)	Amaranthus retroflexus ONTARIO AMES 35199-2 MIRL	1.03	147656862
15<br>(-)	Amaranthus retroflexus UTAH PI612857-1 MIRL	1.02	147656862
16<br>(-)	Amaranthus spinosus HARROW 1 MIRL	1.01	147656862
17<br>(-)	Amaranthus spinosus NC PI632248-RE1 MIRL	1	147656862
18<br>(-)	Amaranthus tuberculatus IOWA PI674264-1 MIRL	0.99	147656862
19<br>(-)	Amaranthus palmeri MEXICO VERACRUZ PI667167-RE2 MIRL	0.96	147656862
20<br>(-)	Amaranthus caudatus CALIFORNIA PI690570-1 MIRL	0.96	147656862

Analyte20

Analyte Name:	Amaranthus watsonii PI 633593-2 MIRL
Analyte Description:	MSP
Analyte ID:	fcace11e-7f50-49ba-971d-00621e011d88
Analyte Creation Date/Time:	2022-06-30 11:12:39 AM
Applied MSP Library(ies):	
Applied Taxonomy Tree:	Bruker Taxonomy

Rank (Quality)	Matched Pattern	Score Value	NCBI Identifier
1 (+++)	Amaranthus watsonii PI 633593-2 MIRL	3	147656862
2 (+++)	Amaranthus watsonii PI 633593-3 MIRL	2.3	147656862
3 (++)	Amaranthus watsonii PI 633593-1 MIRL	2.1	147656862
4 (+)	Amaranthus spinosus PI 632248-1 MIRL	1.78	147656862
5 (-)	Amaranthus spinosus PI 632248-2 MIRL	1.58	147656862
6 (-)	Amaranthus palmeri MEXICO VERACRUZ PI667167-2 MIRL	1.55	147656862
7 (-)	Amaranthus spinosus PI 632248-3 MIRL	1.54	147656862
8 (-)	Amaranthus palmeri MEXICO VERACRUZ PI667167-3 MIRL	1.52	147656862
9 (-)	Amaranthus arenicola PI 599670-2 MIRL	1.47	147656862
10 (-)	Amaranthus arenicola PI 599670-1 MIRL	1.35	147656862
11 (-)	Amaranthus arenicola KANSAS PI599671-1 MIRL	1.28	147656862

12<br>(-)	Amaranthus watsonii MEXICO COLIMA PI633593-RE2 MIRL	1.24	147656862
13<br>(-)	Amaranthus palmeri MEXICO PUEBLA PI604557-3 MIRL	1.24	147656862
14<br>(-)	Amaranthus spinosus HARROW 3 MIRL	1.23	147656862
15<br>(-)	Amaranthus spinosus HARROW 2 MIRL	1.19	147656862
16<br>(-)	Amaranthus spinosus HARROW 1 MIRL	1.17	147656862
17<br>(-)	Amaranthus palmeri MEXICO VERACRUZ PI667167-RE2 MIRL	1.17	147656862
18<br>(-)	Amaranthus palmeri MEXICO VERACRUZ PI667167-RE1 MIRL	1.16	147656862
19<br>(-)	Amaranthus hybridus INDIANA PI603895-2 MIRL	1.16	147656862
20<br>(-)	Amaranthus spinosus NC PI632248-RE1 MIRL	1.15	147656862

Analyte21

Analyte Name:	Amaranthus watsonii PI 633593-3 MIRL
Analyte Description:	MSP
Analyte ID:	fa02ec01-2f7a-40be-a578-6129cf26c7b9
Analyte Creation Date/Time:	2022-06-30 11:20:37 AM
Applied MSP Library(ies):	
Applied Taxonomy Tree:	Bruker Taxonomy

Rank (Quality)	Matched Pattern	Score Value	NCBI Identifier
1 (+++)	Amaranthus watsonii PI 633593-3 MIRL	3	147656862
2 (+++)	Amaranthus watsonii PI 633593-2 MIRL	2.31	147656862
3 (++)	Amaranthus watsonii PI 633593-1 MIRL	2.03	147656862
4 (+)	Amaranthus palmeri MEXICO VERACRUZ PI667167-2 MIRL	1.81	147656862
5 (+)	Amaranthus spinosus PI 632248-1 MIRL	1.7	147656862
6 (-)	Amaranthus spinosus PI 632248-2 MIRL	1.69	147656862
7 (-)	Amaranthus spinosus PI 632248-3 MIRL	1.65	147656862
8 (-)	Amaranthus palmeri MEXICO VERACRUZ PI667167-3 MIRL	1.59	147656862
9 (-)	Amaranthus arenicola PI 599670-1 MIRL	1.32	147656862
10 (-)	Amaranthus arenicola PI 599670-2 MIRL	1.17	147656862
11 (-)	Amaranthus palmeri MEXICO PUEBLA PI604557-1 MIRL	1.14	147656862

12<br>(-)	Amaranthus watsonii MEXICO COLIMA PI633593-RE2 MIRL	1.09	147656862
13<br>(-)	Amaranthus watsonii MEXICO COLIMA PI633593-RE1 MIRL	1.08	147656862
14<br>(-)	Amaranthus tuberculatus IOWA PI674264-1 MIRL	1.05	147656862
15<br>(-)	Amaranthus watsonii MEXICO COLIMA PI633593-RE3 MIRL	1.04	147656862
16<br>(-)	Amaranthus palmeri DAKAR PI633587-1 MIRL	1.03	147656862
17<br>(-)	Amaranthus arenicola KANSAS PI599671-1 MIRL	1.02	147656862
18<br>(-)	Amaranthus palmeri MEXICO PUEBLA PI604557-2 MIRL	0.97	147656862
19<br>(-)	Amaranthus palmeri MEXICO VERACRUZ PI667167-1 MIRL	0.97	147656862
20<br>(-)	Amaranthus palmeri MEXICO PUEBLA PI604557-3 MIRL	0.97	147656862

Analyte22

Analyte Name:	Amaranthus palmeri MEXICO PUEBLA PI604557-1 MIRL
Analyte Description:	MSP
Analyte ID:	3c51dc68-b80e-43c4-9103-277dc3ebf352
Analyte Creation Date/Time:	2022-07-11 2:32:42 PM
Applied MSP Library(ies):	
Applied Taxonomy Tree:	Bruker Taxonomy

Rank (Quality)	Matched Pattern	Score Value	NCBI Identifier
1 (+++)	Amaranthus palmeri MEXICO PUEBLA PI604557-1 MIRL	3	147656862
2 (+++)	Amaranthus palmeri MEXICO PUEBLA PI604557-2 MIRL	2.39	147656862
3 (++)	Amaranthus palmeri MEXICO PUEBLA PI604557-3 MIRL	2.26	147656862
4 (++)	Amaranthus watsonii MEXICO COLIMA PI633593-RE2 MIRL	2.23	147656862
5 (++)	Amaranthus palmeri ARIZONA PI686461-2 MIRL	2.12	147656862
6 (++)	Amaranthus watsonii MEXICO COLIMA PI633593-RE3 MIRL	2.09	147656862
7 (+)	Amaranthus spinosus NC PI632248-RE3 MIRL	1.99	147656862
8 (+)	Amaranthus palmeri MALI PI549158-1 MIRL	1.99	147656862
9 (+)	Amaranthus palmeri MALI PI549158-2 MIRL	1.97	147656862
10 (+)	Amaranthus watsonii MEXICO COLIMA PI633593-RE1 MIRL	1.97	147656862
11 (+)	Amaranthus palmeri DAKAR PI633586-2 MIRL	1.95	147656862

12<br>(+)	Amaranthus palmeri ARIZONA PI686461-1 MIRL	1.94	147656862
13<br>(+)	Amaranthus palmeri USA(AZ) PI632236-3 MIRL	1.9	147656862
14<br>(+)	Amaranthus palmeri MEXICO VERACRUZ PI667167-RE3 MIRL	1.9	147656862
15<br>(+)	Amaranthus spinosus HARROW 3 MIRL	1.9	147656862
16<br>(+)	Amaranthus palmeri DAKAR PI633587-1 MIRL	1.89	147656862
17<br>(+)	Amaranthus palmeri USA(AZ) PI632236-2 MIRL	1.88	147656862
18<br>(+)	Amaranthus palmeri DAKAR PI633586-1 MIRL	1.87	147656862
19<br>(+)	Amaranthus palmeri ARIZONA PI686461-3 MIRL	1.85	147656862
20<br>(+)	Amaranthus palmeri MALI PI549158-3 MIRL	1.82	147656862

Analyte23

Analyte Name:	Amaranthus palmeri MEXICO PUEBLA PI604557-2 MIRL
Analyte Description:	MSP
Analyte ID:	31c635fe-2df8-457f-acb6-5e117fdfdfc0
Analyte Creation Date/Time:	2022-07-11 3:16:22 PM
Applied MSP Library(ies):	
Applied Taxonomy Tree:	Bruker Taxonomy

Rank (Quality)	Matched Pattern	Score Value	NCBI Identifier
1 (+++)	Amaranthus palmeri MEXICO PUEBLA PI604557-2 MIRL	3	147656862
2 (+++)	Amaranthus palmeri MEXICO PUEBLA PI604557-3 MIRL	2.52	147656862
3 (+++)	Amaranthus watsonii MEXICO COLIMA PI633593-RE2 MIRL	2.44	147656862
4 (+++)	Amaranthus palmeri MEXICO PUEBLA PI604557-1 MIRL	2.4	147656862
5 (+++)	Amaranthus palmeri MALI PI549158-1 MIRL	2.32	147656862
6 (+++)	Amaranthus palmeri MALI PI549158-2 MIRL	2.32	147656862
7 (++)	Amaranthus palmeri DAKAR PI633586-1 MIRL	2.21	147656862
8 (++)	Amaranthus palmeri DAKAR PI633587-1 MIRL	2.21	147656862
9 (++)	Amaranthus palmeri ARIZONA PI686461-1 MIRL	2.2	147656862
10 (++)	Amaranthus palmeri DAKAR PI633587-3 MIRL	2.16	147656862
11 (++)	Amaranthus palmeri ARIZONA PI686461-2 MIRL	2.15	147656862

12<br>(++)	Amaranthus palmeri MALI PI549158-3 MIRL	2.13	147656862
13<br>(++)	Amaranthus palmeri USA(AZ) PI632236-2 MIRL	2.13	147656862
14<br>(++)	Amaranthus palmeri ARIZONA PI686461-3 MIRL	2.1	147656862
15<br>(++)	Amaranthus palmeri USA(AZ) PI632236-3 MIRL	2.08	147656862
16<br>(++)	Amaranthus palmeri DAKAR PI633586-2 MIRL	2.07	147656862
17<br>(++)	Amaranthus palmeri DAKAR PI633586-3 MIRL	2.05	147656862
18<br>(++)	Amaranthus palmeri DAKAR PI633587-2 MIRL	2	147656862
19<br>(+)	Amaranthus watsonii MEXICO COLIMA PI633593-RE1 MIRL	1.96	147656862
20<br>(+)	Amaranthus watsonii MEXICO COLIMA PI633593-RE3 MIRL	1.95	147656862

Analyte24

Analyte Name:	Amaranthus palmeri MEXICO PUEBLA PI604557-3 MIRL
Analyte Description:	MSP
Analyte ID:	939d1007-fc59-4e10-80bf-607d1df7fbd9
Analyte Creation Date/Time:	2022-07-11 3:38:32 PM
Applied MSP Library(ies):	
Applied Taxonomy Tree:	Bruker Taxonomy

Rank (Quality)	Matched Pattern	Score Value	NCBI Identifier
1 (+++)	Amaranthus palmeri MEXICO PUEBLA PI604557-3 MIRL	3	147656862
2 (+++)	Amaranthus palmeri MEXICO PUEBLA PI604557-2 MIRL	2.52	147656862
3 (+++)	Amaranthus palmeri MALI PI549158-1 MIRL	2.35	147656862
4 (+++)	Amaranthus watsonii MEXICO COLIMA PI633593-RE2 MIRL	2.33	147656862
5 (++)	Amaranthus palmeri MEXICO PUEBLA PI604557-1 MIRL	2.28	147656862
6 (++)	Amaranthus palmeri MALI PI549158-2 MIRL	2.27	147656862
7 (++)	Amaranthus palmeri ARIZONA PI686461-2 MIRL	2.25	147656862
8 (++)	Amaranthus watsonii MEXICO COLIMA PI633593-RE3 MIRL	2.18	147656862
9 (++)	Amaranthus palmeri DAKAR PI633586-3 MIRL	2.17	147656862
10 (++)	Amaranthus palmeri DAKAR PI633586-2 MIRL	2.16	147656862
11 (++)	Amaranthus palmeri DAKAR PI633586-1 MIRL	2.15	147656862

12<br>(++)	Amaranthus palmeri USA(AZ) PI632236-2 MIRL	2.14	147656862
13<br>(++)	Amaranthus palmeri MALI PI549158-3 MIRL	2.13	147656862
14<br>(++)	Amaranthus palmeri ARIZONA PI686461-1 MIRL	2.1	147656862
15<br>(++)	Amaranthus palmeri ARIZONA PI686461-3 MIRL	2.09	147656862
16<br>(++)	Amaranthus palmeri MEXICO VERACRUZ PI667167-RE1 MIRL	2.08	147656862
17<br>(++)	Amaranthus palmeri DAKAR PI633587-3 MIRL	2.08	147656862
18<br>(++)	Amaranthus watsonii MEXICO COLIMA PI633593-RE1 MIRL	2.06	147656862
19<br>(++)	Amaranthus spinosus NC PI632248-RE3 MIRL	2.06	147656862
20<br>(++)	Amaranthus palmeri USA(AZ) PI632236-3 MIRL	2.02	147656862

Analyte25

Analyte Name:	Amaranthus palmeri MEXICO VERACRUZ PI667167-1 MIRL
Analyte Description:	MSP
Analyte ID:	87fdcaba-c6e7-4e0d-adf8-a8dce8cc4365
Analyte Creation Date/Time:	2022-07-11 3:51:01 PM
Applied MSP Library(ies):	
Applied Taxonomy Tree:	Bruker Taxonomy

Rank (Quality)	Matched Pattern	Score Value	NCBI Identifier
1 (+++)	Amaranthus palmeri MEXICO VERACRUZ PI667167-1 MIRL	3	147656862
2 (++)	Amaranthus palmeri MEXICO VERACRUZ PI667167-RE2 MIRL	2.06	147656862
3 (+)	Amaranthus palmeri MEXICO PUEBLA PI604557-3 MIRL	1.85	147656862
4 (+)	Amaranthus palmeri MEXICO VERACRUZ PI667167-RE1 MIRL	1.81	147656862
5 (+)	Amaranthus watsonii MEXICO COLIMA PI633593-RE1 MIRL	1.8	147656862
6 (+)	Amaranthus palmeri DAKAR PI633586-3 MIRL	1.76	147656862
7 (+)	Amaranthus palmeri MEXICO VERACRUZ PI667167-RE3 MIRL	1.76	147656862
8 (+)	Amaranthus palmeri MALI PI549158-3 MIRL	1.75	147656862
9 (+)	Amaranthus palmeri MEXICO VERACRUZ PI667167-3 MIRL	1.74	147656862
10 (+)	Amaranthus spinosus HARROW 2 MIRL	1.72	147656862
11 (+)	Amaranthus palmeri DAKAR PI633586-2 MIRL	1.72	147656862

12<br>(+)	Amaranthus palmeri USA(AZ) PI632236-2 MIRL	1.71	147656862
13<br>(+)	Amaranthus palmeri MEXICO PUEBLA PI604557-2 MIRL	1.71	147656862
14<br>(-)	Amaranthus watsonii MEXICO COLIMA PI633593-RE3 MIRL	1.69	147656862
15<br>(-)	Amaranthus palmeri DAKAR PI633586-1 MIRL	1.68	147656862
16<br>(-)	Amaranthus watsonii MEXICO COLIMA PI633593-RE2 MIRL	1.63	147656862
17<br>(-)	Amaranthus spinosus HARROW 1 MIRL	1.59	147656862
18<br>(-)	Amaranthus palmeri MEXICO PUEBLA PI604557-1 MIRL	1.58	147656862
19<br>(-)	Amaranthus spinosus NC PI632248-RE3 MIRL	1.58	147656862
20<br>(-)	Amaranthus palmeri ARIZONA PI686461-3 MIRL	1.58	147656862

Analyte26

Analyte Name:	Amaranthus palmeri MEXICO VERACRUZ PI667167-2 MIRL
Analyte Description:	MSP
Analyte ID:	5e4e0f47-ddbc-4309-93b8-ee9737c874e9
Analyte Creation Date/Time:	2022-07-11 4:07:45 PM
Applied MSP Library(ies):	
Applied Taxonomy Tree:	Bruker Taxonomy

Rank (Quality)	Matched Pattern	Score Value	NCBI Identifier
1 (+++)	Amaranthus palmeri MEXICO VERACRUZ PI667167-2 MIRL	3	147656862
2 (++)	Amaranthus spinosus PI 632248-1 MIRL	2.09	147656862
3 (++)	Amaranthus spinosus PI 632248-2 MIRL	2.06	147656862
4 (+)	Amaranthus spinosus PI 632248-3 MIRL	1.93	147656862
5 (+)	Amaranthus watsonii PI 633593-1 MIRL	1.91	147656862
6 (+)	Amaranthus watsonii PI 633593-3 MIRL	1.8	147656862
7 (-)	Amaranthus watsonii PI 633593-2 MIRL	1.56	147656862
8 (-)	Amaranthus palmeri MEXICO VERACRUZ PI667167-3 MIRL	1.38	147656862
9 (-)	Amaranthus spinosus HARROW 2 MIRL	1.19	147656862
10 (-)	Amaranthus spinosus HARROW 1 MIRL	1.18	147656862
11 (-)	Amaranthus palmeri ARIZONA PI686461-1 MIRL	1.02	147656862

12<br>(-)	Amaranthus hybridus INDIANA PI603895-3 MIRL	1	147656862
13<br>(-)	Amaranthus hybridus PUERTO RICO AMES 5152-2 MIRL	0.99	147656862
14<br>(-)	Amaranthus retroflexus UTAH PI612857-1 MIRL	0.97	147656862
15<br>(-)	Amaranthus hypochondriacus MISSOURI PI698341-2 MIRL	0.86	147656862
16<br>(-)	Amaranthus palmeri DAKAR PI633587-1 MIRL	0.86	147656862
17<br>(-)	Amaranthus arenicola TEXAS PI667168-1 MIRL	0.84	147656862
18<br>(-)	Amaranthus tuberculatus IOWA PI604247-3 MIRL	0.84	147656862
19<br>(-)	Amaranthus tuberculatus IOWA PI674264-1 MIRL	0.83	147656862
20<br>(-)	Amaranthus retroflexus UTAH PI612857-2 MIRL	0.83	147656862

Analyte27

Analyte Name:	Amaranthus palmeri MEXICO VERACRUZ PI667167-3 MIRL
Analyte Description:	MSP
Analyte ID:	716561d3-ff07-430f-a84f-3089880016af
Analyte Creation Date/Time:	2022-07-11 4:28:37 PM
Applied MSP Library(ies):	
Applied Taxonomy Tree:	Bruker Taxonomy

Rank (Quality)	Matched Pattern	Score Value	NCBI Identifier
1 (+++)	Amaranthus palmeri MEXICO VERACRUZ PI667167-3 MIRL	3	147656862
2 (+)	Amaranthus palmeri MEXICO VERACRUZ PI667167-RE3 MIRL	1.81	147656862
3 (+)	Amaranthus spinosus HARROW 2 MIRL	1.8	147656862
4 (+)	Amaranthus palmeri MEXICO VERACRUZ PI667167-RE1 MIRL	1.79	147656862
5 (+)	Amaranthus spinosus HARROW 3 MIRL	1.79	147656862
6 (+)	Amaranthus palmeri MEXICO VERACRUZ PI667167-RE2 MIRL	1.76	147656862
7 (+)	Amaranthus watsonii MEXICO COLIMA PI633593-RE1 MIRL	1.75	147656862
8 (+)	Amaranthus palmeri MEXICO VERACRUZ PI667167-1 MIRL	1.73	147656862
9 (+)	Amaranthus spinosus HARROW 1 MIRL	1.72	147656862
10 (-)	Amaranthus spinosus PI 632248-3 MIRL	1.68	147656862
11 (-)	Amaranthus watsonii MEXICO COLIMA PI633593-RE3 MIRL	1.64	147656862

12<br>(-)	Amaranthus palmeri MEXICO PUEBLA PI604557-3 MIRL	1.62	147656862
13<br>(-)	Amaranthus spinosus PI 632248-1 MIRL	1.62	147656862
14<br>(-)	Amaranthus watsonii PI 633593-3 MIRL	1.59	147656862
15<br>(-)	Amaranthus spinosus NC PI632248-RE3 MIRL	1.58	147656862
16<br>(-)	Amaranthus watsonii PI 633593-2 MIRL	1.56	147656862
17<br>(-)	Amaranthus spinosus PI 632248-2 MIRL	1.53	147656862
18<br>(-)	Amaranthus palmeri MALI PI549158-3 MIRL	1.5	147656862
19<br>(-)	Amaranthus watsonii MEXICO COLIMA PI633593-RE2 MIRL	1.48	147656862
20<br>(-)	Amaranthus spinosus NC PI632248-RE1 MIRL	1.47	147656862

Analyte28

Analyte Name:	Amaranthus hypochondriacus PI658730-1 MIRL
Analyte Description:	MSP
Analyte ID:	86aa2565-4e17-4bf9-9139-ca5d3f3b4da0
Analyte Creation Date/Time:	2022-10-25 2:40:28 PM
Applied MSP Library(ies):	
Applied Taxonomy Tree:	Bruker Taxonomy

Rank (Quality)	Matched Pattern	Score Value	NCBI Identifier
1 (+++)	Amaranthus hypochondriacus PI658730-1 MIRL	3	147656862
2 (+++)	Amaranthus hypochondriacus PI658730-2 MIRL	2.8	147656862
3 (+++)	Amaranthus hypochondriacus PI658730-3 MIRL	2.76	147656862
4 (+++)	Amaranthus hypochondriacus MISSOURI PI698341-1 MIRL	2.62	147656862
5 (+++)	Amaranthus hypochondriacus MISSOURI PI698341-3 MIRL	2.55	147656862
6 (+++)	Amaranthus caudatus NJ AMES 5687-1 MIRL	2.5	147656862
7 (+++)	Amaranthus hypochondriacus MISSOURI PI698341-2 MIRL	2.49	147656862
8 (+++)	Amaranthus hybridus PUERTO RICO AMES 5152-3 MIRL	2.48	147656862
9 (+++)	Amaranthus hybridus PUERTO RICO AMES 5152-1 MIRL	2.44	147656862
10 (+++)	Amaranthus hybridus HARROW 3 MIRL	2.42	147656862
11 (+++)	Amaranthus hybridus PUERTO RICO AMES 5152-2 MIRL	2.42	147656862

12<br>(+++)	Amaranthus caudatus NJ AMES 5687-2 MIRL	2.41	147656862
13<br>(+++)	Amaranthus hybridus HARROW 2 MIRL	2.36	147656862
14<br>(+++)	Amaranthus hypochondriacus MEXICO SONORA PI599682-1 MIRL	2.35	147656862
15<br>(++)	Amaranthus caudatus NJ AMES 5687-3 MIRL	2.27	147656862
16<br>(++)	Amaranthus hybridus HARROW 1 MIRL	2.12	147656862
17<br>(++)	Amaranthus hypochondriacus MEXICO SONORA PI599682-2 MIRL	2.07	147656862
18<br>(++)	Amaranthus hypochondriacus MEXICO SONORA PI599682-3 MIRL	2.04	147656862
19<br>(+)	Amaranthus hybridus INDIANA PI603895-2 MIRL	1.85	147656862
20<br>(+)	Amaranthus powellii sub. Powellii TEXAS PI632241-1 MIRL	1.72	147656862

Analyte29

Analyte Name: Amaranthus hypochondriacus PI658730-2 MIRL
Analyte Description: MSP
Analyte ID: 16c31e49-8f73-45a0-a18f-e9690525f8b0
Analyte Creation Date/Time: 2022-10-25 2:52:46 PM
Applied MSP Library(ies):
Applied Taxonomy Tree: Bruker Taxonomy

Rank (Quality)	Matched Pattern	Score Value	NCBI Identifier
1 (+++)	Amaranthus hypochondriacus PI658730-2 MIRL	3	147656862
2 (+++)	Amaranthus hypochondriacus PI658730-1 MIRL	2.8	147656862
3 (+++)	Amaranthus hypochondriacus PI658730-3 MIRL	2.78	147656862
4 (+++)	Amaranthus hypochondriacus MISSOURI PI698341-1 MIRL	2.73	147656862
5 (+++)	Amaranthus hypochondriacus MISSOURI PI698341-3 MIRL	2.57	147656862
6 (+++)	Amaranthus hypochondriacus MISSOURI PI698341-2 MIRL	2.54	147656862
7 (+++)	Amaranthus caudatus NJ AMES 5687-1 MIRL	2.49	147656862
8 (+++)	Amaranthus hybridus HARROW 3 MIRL	2.48	147656862
9 (+++)	Amaranthus hybridus PUERTO RICO AMES 5152-3 MIRL	2.46	147656862
10 (+++)	Amaranthus caudatus NJ AMES 5687-2 MIRL	2.42	147656862
11 (+++)	Amaranthus hybridus PUERTO RICO AMES 5152-2 MIRL	2.39	147656862

12 (+++)	Amaranthus hybridus HARROW 2 MIRL	2.37	147656862
13 (+++)	Amaranthus hypochondriacus MEXICO SONORA PI599682-1 MIRL	2.36	147656862
14 (+++)	Amaranthus caudatus NJ AMES 5687-3 MIRL	2.33	147656862
15 (++)	Amaranthus hybridus PUERTO RICO AMES 5152-1 MIRL	2.27	147656862
16 (++)	Amaranthus hypochondriacus MEXICO SONORA PI599682-2 MIRL	2.26	147656862
17 (++)	Amaranthus hybridus HARROW 1 MIRL	2.24	147656862
18 (++)	Amaranthus hypochondriacus MEXICO SONORA PI599682-3 MIRL	2.18	147656862
19 (+)	Amaranthus powellii sub. Powellii TEXAS PI632241-1 MIRL	1.91	147656862
20 (+)	Amaranthus hybridus INDIANA PI603895-2 MIRL	1.84	147656862

Analyte30

Analyte Name:	Amaranthus albus PI633580-1 MIRL
Analyte Description:	MSP
Analyte ID:	3130997f-4948-431f-b63e-a3284abcbfb5
Analyte Creation Date/Time:	2022-10-25 3:11:11 PM
Applied MSP Library(ies):	
Applied Taxonomy Tree:	Bruker Taxonomy

Rank (Quality)	Matched Pattern	Score Value	NCBI Identifier
1 (+++)	Amaranthus albus PI633580-1 MIRL	3	147656862
2 (+++)	Amaranthus albus PI633580-3 MIRL	2.65	147656862
3 (++)	Amaranthus albus NC PI632244-1 MIRL	2.23	147656862
4 (++)	Amaranthus albus NC PI632244-2 MIRL	2.16	147656862
5 (+)	Amaranthus albus NC PI632244-3 MIRL	1.92	147656862
6 (+)	Amaranthus albus WASHINGTON PI654389-3 MIRL	1.88	147656862
7 (+)	Amaranthus albus HARROW 1 MIRL	1.87	147656862
8 (+)	Amaranthus albus PI633580-2 MIRL	1.84	147656862
9 (+)	Amaranthus albus WASHINGTON PI654389-1 MIRL	1.75	147656862
10 (-)	Amaranthus albus HARROW 2 MIRL	1.61	147656862
11 (-)	Amaranthus albus HARROW 3 MIRL	1.47	147656862

12<br>**(-)**	Amaranthus blitoides HARROW 2 MIRL	1.43	147656862
13<br>**(-)**	Amaranthus blitoides CANADA PI608663-1 MIRL	1.42	147656862
14<br>**(-)**	Amaranthus blitoides CANADA PI608663-3 MIRL	1.39	147656862
15<br>**(-)**	Amaranthus caudatus NJ AMES 5687-2 MIRL	1.38	147656862
16<br>**(-)**	Amaranthus caudatus NJ AMES 5687-1 MIRL	1.37	147656862
17<br>**(-)**	Amaranthus hypochondriacus PI658730-1 MIRL	1.35	147656862
18<br>**(-)**	Amaranthus albus WASHINGTON PI654389-2 MIRL	1.31	147656862
19<br>**(-)**	Amaranthus blitoides HARROW 3 MIRL	1.31	147656862
20<br>**(-)**	Amaranthus blitoides CANADA PI608663-2 MIRL	1.26	147656862

Analyte31

Analyte Name: Amaranthus albus PI633580-3 MIRL
Analyte Description: MSP
Analyte ID: 67b326de-a817-47bb-a892-85b48d3beab4
Analyte Creation Date/Time: 2022-10-25 3:32:50 PM
Applied MSP Library(ies):
Applied Taxonomy Tree: Bruker Taxonomy

Rank (Quality)	Matched Pattern	Score Value	NCBI Identifier
1 (+++)	Amaranthus albus PI633580-3 MIRL	3	147656862
2 (+++)	Amaranthus albus PI633580-1 MIRL	2.65	147656862
3 (+++)	Amaranthus albus NC PI632244-1 MIRL	2.42	147656862
4 (+++)	Amaranthus albus NC PI632244-2 MIRL	2.3	147656862
5 (++)	Amaranthus albus WASHINGTON PI654389-3 MIRL	2.09	147656862
6 (++)	Amaranthus albus NC PI632244-3 MIRL	2.08	147656862
7 (++)	Amaranthus albus WASHINGTON PI654389-1 MIRL	2	147656862
8 (+)	Amaranthus albus PI633580-2 MIRL	1.88	147656862
9 (+)	Amaranthus albus HARROW 1 MIRL	1.86	147656862
10 (+)	Amaranthus albus HARROW 2 MIRL	1.85	147656862
11 (+)	Amaranthus albus HARROW 3 MIRL	1.74	147656862

12<br>(+)	Amaranthus albus WASHINGTON PI654389-2 MIRL	1.71	147656862
13<br>(-)	Amaranthus blitoides HARROW 3 MIRL	1.56	147656862
14<br>(-)	Amaranthus blitoides CANADA PI608663-3 MIRL	1.45	147656862
15<br>(-)	Amaranthus blitoides CANADA PI608663-1 MIRL	1.43	147656862
16<br>(-)	Amaranthus blitoides CANADA PI608663-2 MIRL	1.33	147656862
17<br>(-)	Amaranthus californicus CALIFORNIA PI595319-2 MIRL	1.31	147656862
18<br>(-)	Amaranthus blitoides HARROW 2 MIRL	1.28	147656862
19<br>(-)	Amaranthus caudatus NJ AMES 5687-1 MIRL	1.24	147656862
20<br>(-)	Amaranthus hybridus INDIANA PI603895-2 MIRL	1.21	147656862

Analyte32

Analyte Name:	Amaranthus hypochondriacus PI658730-3 MIRL
Analyte Description:	MSP
Analyte ID:	5497927f-35e1-4ad6-8d94-c087c11910ef
Analyte Creation Date/Time:	2022-10-25 3:40:45 PM
Applied MSP Library(ies):	
Applied Taxonomy Tree:	Bruker Taxonomy

Rank (Quality)	Matched Pattern	Score Value	NCBI Identifier
1 (+++)	Amaranthus hypochondriacus PI658730-3 MIRL	3	147656862
2 (+++)	Amaranthus hypochondriacus PI658730-2 MIRL	2.79	147656862
3 (+++)	Amaranthus hypochondriacus PI658730-1 MIRL	2.76	147656862
4 (+++)	Amaranthus hypochondriacus MISSOURI PI698341-1 MIRL	2.63	147656862
5 (+++)	Amaranthus caudatus NJ AMES 5687-1 MIRL	2.49	147656862
6 (+++)	Amaranthus hypochondriacus MISSOURI PI698341-3 MIRL	2.43	147656862
7 (+++)	Amaranthus hypochondriacus MISSOURI PI698341-2 MIRL	2.38	147656862
8 (+++)	Amaranthus hybridus PUERTO RICO AMES 5152-3 MIRL	2.34	147656862
9 (+++)	Amaranthus hybridus PUERTO RICO AMES 5152-2 MIRL	2.32	147656862
10 (+++)	Amaranthus hybridus HARROW 3 MIRL	2.32	147656862
11 (++)	Amaranthus caudatus NJ AMES 5687-2 MIRL	2.29	147656862

12 (++)	Amaranthus hybridus PUERTO RICO AMES 5152-1 MIRL	2.23	147656862
13 (++)	Amaranthus hybridus HARROW 2 MIRL	2.22	147656862
14 (++)	Amaranthus caudatus NJ AMES 5687-3 MIRL	2.2	147656862
15 (++)	Amaranthus hypochondriacus MEXICO SONORA PI599682-1 MIRL	2.19	147656862
16 (++)	Amaranthus hybridus HARROW 1 MIRL	2.03	147656862
17 (++)	Amaranthus hypochondriacus MEXICO SONORA PI599682-2 MIRL	2	147656862
18 (+)	Amaranthus hypochondriacus MEXICO SONORA PI599682-3 MIRL	1.89	147656862
19 (+)	Amaranthus powellii sub. Powellii TEXAS PI632241-1 MIRL	1.72	147656862
20 (-)	Amaranthus powelli sub. powelli ME AMES 29205-2 MIRL	1.69	147656862

Analyte33

Analyte Name:	Amaranthus albus PI633580-2 MIRL
Analyte Description:	MSP
Analyte ID:	31078ff9-cfea-4822-8b1f-42894be3004b
Analyte Creation Date/Time:	2022-10-25 3:47:45 PM
Applied MSP Library(ies):	
Applied Taxonomy Tree:	Bruker Taxonomy

Rank (Quality)	Matched Pattern	Score Value	NCBI Identifier
1 (+++)	Amaranthus albus PI633580-2 MIRL	3	147656862
2 (++)	Amaranthus albus WASHINGTON PI654389-2 MIRL	2.23	147656862
3 (++)	Amaranthus albus HARROW 3 MIRL	2.08	147656862
4 (+)	Amaranthus albus WASHINGTON PI654389-3 MIRL	1.98	147656862
5 (+)	Amaranthus albus WASHINGTON PI654389-1 MIRL	1.89	147656862
6 (+)	Amaranthus albus PI633580-1 MIRL	1.84	147656862
7 (+)	Amaranthus albus NC PI632244-2 MIRL	1.83	147656862
8 (+)	Amaranthus albus PI633580-3 MIRL	1.79	147656862
9 (+)	Amaranthus albus HARROW 2 MIRL	1.73	147656862
10 (-)	Amaranthus albus NC PI632244-1 MIRL	1.69	147656862
11 (-)	Amaranthus albus HARROW 1 MIRL	1.6	147656862

12<br>**(-)**	Amaranthus albus NC PI632244-3 MIRL	1.45	147656862
13<br>**(-)**	Amaranthus californicus CALIFORNIA PI595319-1 MIRL	1.31	147656862
14<br>**(-)**	Amaranthus arenicola TEXAS PI667168-1 MIRL	1.1	147656862
15<br>**(-)**	Amaranthus caudatus NJ AMES 5687-2 MIRL	1.08	147656862
16<br>**(-)**	Amaranthus caudatus NJ PI553073-1 MIRL	1.05	147656862
17<br>**(-)**	Amaranthus caudatus NJ AMES 5687-1 MIRL	1.03	147656862
18<br>**(-)**	Amaranthus hypochondriacus PI658730-1 MIRL	0.99	147656862
19<br>**(-)**	Amaranthus tuberculatus IOWA PI 553086-2 MIRL	0.99	147656862
20<br>**(-)**	Amaranthus tuberculatus IOWA PI604247-1 MIRL	0.98	147656862

Analyte34

Analyte Name:	Amaranthus palmeri DAKAR PI633587-1 MIRL
Analyte Description:	MSP
Analyte ID:	3c4e53ca-5464-412d-aa94-e1196d027ed3
Analyte Creation Date/Time:	2022-10-27 12:32:24 PM
Applied MSP Library(ies):	
Applied Taxonomy Tree:	Bruker Taxonomy

Rank (Quality)	Matched Pattern	Score Value	NCBI Identifier
1 (+++)	Amaranthus palmeri DAKAR PI633587-1 MIRL	3	147656862
2 (+++)	Amaranthus palmeri DAKAR PI633587-2 MIRL	2.63	147656862
3 (+++)	Amaranthus palmeri ARIZONA PI686461-1 MIRL	2.62	147656862
4 (+++)	Amaranthus palmeri DAKAR PI633587-3 MIRL	2.59	147656862
5 (+++)	Amaranthus palmeri DAKAR PI633586-1 MIRL	2.54	147656862
6 (+++)	Amaranthus palmeri ARIZONA PI686461-3 MIRL	2.52	147656862
7 (+++)	Amaranthus palmeri DAKAR PI633586-3 MIRL	2.51	147656862
8 (+++)	Amaranthus palmeri DAKAR PI633586-2 MIRL	2.41	147656862
9 (+++)	Amaranthus palmeri ARIZONA PI686461-2 MIRL	2.38	147656862
10 (+++)	Amaranthus palmeri MALI PI549158-1 MIRL	2.36	147656862
11 (+++)	Amaranthus palmeri MALI PI549158-2 MIRL	2.31	147656862

12 (+++)	Amaranthus palmeri USA(AZ) PI632236-2 MIRL	2.3	147656862
13 (++)	Amaranthus watsonii MEXICO COLIMA PI633593-RE2 MIRL	2.23	147656862
14 (++)	Amaranthus palmeri MEXICO PUEBLA PI604557-2 MIRL	2.22	147656862
15 (++)	Amaranthus palmeri MALI PI549158-3 MIRL	2.1	147656862
16 (++)	Amaranthus palmeri USA(AZ) PI632236-3 MIRL	2.07	147656862
17 (+)	Amaranthus palmeri MEXICO VERACRUZ PI667167-RE1 MIRL	1.93	147656862
18 (+)	Amaranthus palmeri MEXICO PUEBLA PI604557-1 MIRL	1.92	147656862
19 (+)	Amaranthus palmeri MEXICO PUEBLA PI604557-3 MIRL	1.89	147656862
20 (+)	Amaranthus spinosus NC PI632248-RE3 MIRL	1.83	147656862

Analyte35

Analyte Name:	Amaranthus palmeri DAKAR PI633587-2 MIRL
Analyte Description:	MSP
Analyte ID:	fa94ff1c-4288-4d51-a583-6e96cdf6ccd0
Analyte Creation Date/Time:	2022-10-27 12:42:12 PM
Applied MSP Library(ies):	
Applied Taxonomy Tree:	Bruker Taxonomy

Rank (Quality)	Matched Pattern	Score Value	NCBI Identifier
1 (+++)	Amaranthus palmeri DAKAR PI633587-2 MIRL	3	147656862
2 (+++)	Amaranthus palmeri DAKAR PI633587-1 MIRL	2.63	147656862
3 (+++)	Amaranthus palmeri ARIZONA PI686461-1 MIRL	2.54	147656862
4 (+++)	Amaranthus palmeri DAKAR PI633587-3 MIRL	2.52	147656862
5 (+++)	Amaranthus palmeri ARIZONA PI686461-3 MIRL	2.5	147656862
6 (+++)	Amaranthus palmeri DAKAR PI633586-1 MIRL	2.46	147656862
7 (+++)	Amaranthus palmeri DAKAR PI633586-3 MIRL	2.3	147656862
8 (+++)	Amaranthus palmeri DAKAR PI633586-2 MIRL	2.3	147656862
9 (++)	Amaranthus palmeri ARIZONA PI686461-2 MIRL	2.28	147656862
10 (++)	Amaranthus palmeri MALI PI549158-1 MIRL	2.27	147656862
11 (++)	Amaranthus palmeri USA(AZ) PI632236-2 MIRL	2.23	147656862

12<br>(++)	Amaranthus palmeri USA(AZ) PI632236-3 MIRL	2.11	147656862
13<br>(++)	Amaranthus palmeri MALI PI549158-2 MIRL	2.1	147656862
14<br>(++)	Amaranthus palmeri MALI PI549158-3 MIRL	2.01	147656862
15<br>(++)	Amaranthus palmeri MEXICO PUEBLA PI604557-3 MIRL	2.01	147656862
16<br>(++)	Amaranthus palmeri MEXICO PUEBLA PI604557-2 MIRL	2	147656862
17<br>(+)	Amaranthus watsonii MEXICO COLIMA PI633593-RE2 MIRL	1.98	147656862
18<br>(+)	Amaranthus spinosus NC PI632248-RE3 MIRL	1.78	147656862
19<br>(+)	Amaranthus palmeri MEXICO VERACRUZ PI667167-RE3 MIRL	1.77	147656862
20<br>(+)	Amaranthus watsonii MEXICO COLIMA PI633593-RE3 MIRL	1.77	147656862

Analyte36

Analyte Name:	Amaranthus palmeri DAKAR PI633587-3 MIRL
Analyte Description:	MSP
Analyte ID:	9a8baaf1-1c1d-4aae-a8c9-f232da53f663
Analyte Creation Date/Time:	2022-10-27 12:48:48 PM
Applied MSP Library(ies):	
Applied Taxonomy Tree:	Bruker Taxonomy

Rank (Quality)	Matched Pattern	Score Value	NCBI Identifier
1 (+++)	Amaranthus palmeri DAKAR PI633587-3 MIRL	3	147656862
2 (+++)	Amaranthus palmeri DAKAR PI633587-1 MIRL	2.59	147656862
3 (+++)	Amaranthus palmeri ARIZONA PI686461-3 MIRL	2.54	147656862
4 (+++)	Amaranthus palmeri ARIZONA PI686461-1 MIRL	2.53	147656862
5 (+++)	Amaranthus palmeri DAKAR PI633586-1 MIRL	2.53	147656862
6 (+++)	Amaranthus palmeri DAKAR PI633587-2 MIRL	2.53	147656862
7 (+++)	Amaranthus palmeri DAKAR PI633586-3 MIRL	2.52	147656862
8 (+++)	Amaranthus palmeri ARIZONA PI686461-2 MIRL	2.42	147656862
9 (+++)	Amaranthus palmeri DAKAR PI633586-2 MIRL	2.39	147656862
10 (+++)	Amaranthus palmeri USA(AZ) PI632236-2 MIRL	2.34	147656862
11 (++)	Amaranthus palmeri MALI PI549158-1 MIRL	2.25	147656862

12<br>(++)	Amaranthus palmeri MALI PI549158-2 MIRL	2.25	147656862
13<br>(++)	Amaranthus palmeri MEXICO PUEBLA PI604557-2 MIRL	2.17	147656862
14<br>(++)	Amaranthus palmeri MALI PI549158-3 MIRL	2.13	147656862
15<br>(++)	Amaranthus palmeri MEXICO PUEBLA PI604557-3 MIRL	2.09	147656862
16<br>(++)	Amaranthus palmeri USA(AZ) PI632236-3 MIRL	2.04	147656862
17<br>(++)	Amaranthus watsonii MEXICO COLIMA PI633593-RE2 MIRL	2.01	147656862
18<br>(++)	Amaranthus watsonii MEXICO COLIMA PI633593-RE3 MIRL	2	147656862
19<br>(+)	Amaranthus palmeri USA(AZ) PI632236-1 MIRL	1.84	147656862
20<br>(+)	Amaranthus palmeri MEXICO VERACRUZ PI667167-RE1 MIRL	1.84	147656862

Analyte37

Analyte Name:	Amaranthus tuberculatus IOWA PI674264-1 MIRL
Analyte Description:	MSP
Analyte ID:	a48a9897-a0ea-40ac-a6b4-afb63fdc48e6
Analyte Creation Date/Time:	2022-10-27 12:56:50 PM
Applied MSP Library(ies):	
Applied Taxonomy Tree:	Bruker Taxonomy

Rank (Quality)	Matched Pattern	Score Value	NCBI Identifier
1 (+++)	Amaranthus tuberculatus IOWA PI674264-1 MIRL	3	147656862
2 (+++)	Amaranthus tuberculatus IOWA PI674264-2 MIRL	2.43	147656862
3 (++)	Amaranthus tuberculatus IOWA PI604247-2 MIRL	2.26	147656862
4 (++)	Amaranthus tuberculatus IOWA PI674264-3 MIRL	2.25	147656862
5 (++)	Amaranthus tuberculatus IOWA PI604247-3 MIRL	2.04	147656862
6 (++)	Amaranthus tuberculatus IOWA PI604247-1 MIRL	2.03	147656862
7 (+)	Amaranthus rudis HARROW 2 MIRL	1.79	147656862
8 (+)	Amaranthus rudis HARROW 1 MIRL	1.78	147656862
9 (+)	Amaranthus tuberculatus KANSAS PI 60743-2 MIRL	1.77	147656862
10 (+)	Amaranthus tuberculatus KANSAS PI 60743-3 MIRL	1.77	147656862
11 (+)	Amaranthus tuberculatus IOWA PI 553086-3 MIRL	1.75	147656862

12<br>(+)	Amaranthus tuberculatus IOWA PI 553086-2 MIRL	1.72	147656862
13<br>(+)	Amaranthus arenicola TEXAS PI667168-1 MIRL	1.71	147656862
14<br>(-)	Amaranthus tuberculatus IOWA PI 553086-1 MIRL	1.67	147656862
15<br>(-)	Amaranthus rudis HARROW 3 MIRL	1.57	147656862
16<br>(-)	Amaranthus tuberculatus KANSAS PI 60743-1 MIRL	1.53	147656862
17<br>(-)	Amaranthus arenicola TEXAS PI667168-3 MIRL	1.52	147656862
18<br>(-)	Amaranthus arenicola KANSAS PI599671-1 MIRL	1.4	147656862
19<br>(-)	Amaranthus arenicola TEXAS PI667168-2 MIRL	1.32	147656862
20<br>(-)	Amaranthus palmeri USA(AZ) PI632236-3 MIRL	1.17	147656862

Analyte38

Analyte Name:	Amaranthus tuberculatus IOWA PI674264-2 MIRL
Analyte Description:	MSP
Analyte ID:	2bf6955e-571f-4f82-a7f7-c24d9cb43aec
Analyte Creation Date/Time:	2022-10-27 2:23:57 PM
Applied MSP Library(ies):	
Applied Taxonomy Tree:	Bruker Taxonomy

Rank (Quality)	Matched Pattern	Score Value	NCBI Identifier
1 (+++)	Amaranthus tuberculatus IOWA PI674264-2 MIRL	3	147656862
2 (+++)	Amaranthus tuberculatus IOWA PI674264-1 MIRL	2.43	147656862
3 (+++)	Amaranthus tuberculatus IOWA PI604247-2 MIRL	2.37	147656862
4 (++)	Amaranthus tuberculatus IOWA PI604247-3 MIRL	2.28	147656862
5 (++)	Amaranthus tuberculatus IOWA PI604247-1 MIRL	2.25	147656862
6 (++)	Amaranthus tuberculatus IOWA PI674264-3 MIRL	2.22	147656862
7 (++)	Amaranthus rudis HARROW 1 MIRL	2.02	147656862
8 (+)	Amaranthus rudis HARROW 2 MIRL	1.93	147656862
9 (+)	Amaranthus tuberculatus IOWA PI 553086-1 MIRL	1.82	147656862
10 (+)	Amaranthus tuberculatus KANSAS PI 60743-3 MIRL	1.79	147656862
11 (+)	Amaranthus rudis HARROW 3 MIRL	1.73	147656862

12<br>(+)	Amaranthus tuberculatus IOWA PI 553086-2 MIRL	1.72	147656862
13<br>(+)	Amaranthus arenicola TEXAS PI667168-3 MIRL	1.71	147656862
14<br>(-)	Amaranthus tuberculatus KANSAS PI 60743-2 MIRL	1.66	147656862
15<br>(-)	Amaranthus arenicola TEXAS PI667168-1 MIRL	1.61	147656862
16<br>(-)	Amaranthus tuberculatus IOWA PI 553086-3 MIRL	1.61	147656862
17<br>(-)	Amaranthus tuberculatus KANSAS PI 60743-1 MIRL	1.44	147656862
18<br>(-)	Amaranthus arenicola KANSAS PI599671-1 MIRL	1.31	147656862
19<br>(-)	Amaranthus arenicola TEXAS PI667168-2 MIRL	1.25	147656862
20<br>(-)	Amaranthus blitum var. pseudogracilis NC PI632245-2 MIRL	1.19	147656862

Analyte39

Analyte Name:	Amaranthus tuberculatus IOWA PI674264-3 MIRL
Analyte Description:	MSP
Analyte ID:	24be68a9-fd13-4a9b-99e0-aef734f1d6d7
Analyte Creation Date/Time:	2022-10-27 2:30:52 PM
Applied MSP Library(ies):	
Applied Taxonomy Tree:	Bruker Taxonomy

Rank (Quality)	Matched Pattern	Score Value	NCBI Identifier
1 (+++)	Amaranthus tuberculatus IOWA PI674264-3 MIRL	3	147656862
2 (++)	Amaranthus tuberculatus IOWA PI674264-1 MIRL	2.25	147656862
3 (++)	Amaranthus tuberculatus IOWA PI674264-2 MIRL	2.22	147656862
4 (++)	Amaranthus tuberculatus IOWA PI604247-2 MIRL	2.18	147656862
5 (++)	Amaranthus tuberculatus IOWA PI604247-3 MIRL	2.18	147656862
6 (+)	Amaranthus tuberculatus IOWA PI604247-1 MIRL	1.97	147656862
7 (+)	Amaranthus rudis HARROW 2 MIRL	1.93	147656862
8 (+)	Amaranthus arenicola TEXAS PI667168-3 MIRL	1.85	147656862
9 (+)	Amaranthus rudis HARROW 1 MIRL	1.84	147656862
10 (+)	Amaranthus tuberculatus KANSAS PI 60743-3 MIRL	1.75	147656862
11 (+)	Amaranthus arenicola TEXAS PI667168-2 MIRL	1.74	147656862

12<br>(+)	Amaranthus rudis HARROW 3 MIRL	1.7	147656862
13<br>(-)	Amaranthus arenicola TEXAS PI667168-1 MIRL	1.63	147656862
14<br>(-)	Amaranthus arenicola KANSAS PI599671-1 MIRL	1.48	147656862
15<br>(-)	Amaranthus tuberculatus KANSAS PI 60743-2 MIRL	1.43	147656862
16<br>(-)	Amaranthus tuberculatus IOWA PI 553086-3 MIRL	1.43	147656862
17<br>(-)	Amaranthus caudatus NJ AMES 5687-3 MIRL	1.28	147656862
18<br>(-)	Amaranthus tuberculatus KANSAS PI 60743-1 MIRL	1.25	147656862
19<br>(-)	Amaranthus blitum var. pseudogracilis NC PI632245-1 MIRL	1.19	147656862
20<br>(-)	Amaranthus hypochondriacus MEXICO SONORA PI599682-2 MIRL	1.15	147656862

Analyte40

Analyte Name:	Amaranthus tuberculatus IOWA PI604247-1 MIRL
Analyte Description:	MSP
Analyte ID:	821a14d1-d970-4336-8b11-e8b9f76c20f4
Analyte Creation Date/Time:	2022-10-27 2:37:40 PM
Applied MSP Library(ies):	
Applied Taxonomy Tree:	Bruker Taxonomy

Rank (Quality)	Matched Pattern	Score Value	NCBI Identifier
1 (+++)	Amaranthus tuberculatus IOWA PI604247-1 MIRL	3	147656862
2 (+++)	Amaranthus tuberculatus IOWA PI604247-2 MIRL	2.74	147656862
3 (+++)	Amaranthus tuberculatus IOWA PI604247-3 MIRL	2.53	147656862
4 (++)	Amaranthus tuberculatus IOWA PI674264-2 MIRL	2.24	147656862
5 (++)	Amaranthus tuberculatus IOWA PI674264-1 MIRL	2.03	147656862
6 (+)	Amaranthus rudis HARROW 1 MIRL	1.97	147656862
7 (+)	Amaranthus tuberculatus IOWA PI674264-3 MIRL	1.97	147656862
8 (-)	Amaranthus tuberculatus KANSAS PI 60743-1 MIRL	1.65	147656862
9 (-)	Amaranthus rudis HARROW 2 MIRL	1.64	147656862
10 (-)	Amaranthus tuberculatus KANSAS PI 60743-2 MIRL	1.54	147656862
11 (-)	Amaranthus tuberculatus KANSAS PI 60743-3 MIRL	1.54	147656862

12<br>(-)	Amaranthus rudis HARROW 3 MIRL	1.51	147656862
13<br>(-)	Amaranthus arenicola TEXAS PI667168-1 MIRL	1.51	147656862
14<br>(-)	Amaranthus tuberculatus IOWA PI 553086-3 MIRL	1.41	147656862
15<br>(-)	Amaranthus arenicola TEXAS PI667168-3 MIRL	1.39	147656862
16<br>(-)	Amaranthus arenicola TEXAS PI667168-2 MIRL	1.34	147656862
17<br>(-)	Amaranthus tuberculatus IOWA PI 553086-2 MIRL	1.14	147656862
18<br>(-)	Amaranthus blitum var. pseudogracilis NC PI632245-3 MIRL	1.14	147656862
19<br>(-)	Amaranthus albus NC PI632244-2 MIRL	1.12	147656862
20<br>(-)	Amaranthus watsonii MEXICO COLIMA PI633593-RE3 MIRL	1.11	147656862

Analyte41

Analyte Name:	Amaranthus tuberculatus IOWA PI604247-2 MIRL
Analyte Description:	MSP
Analyte ID:	181a027f-5d96-4e00-aaa1-f5b0aefad99b
Analyte Creation Date/Time:	2022-10-27 2:43:49 PM
Applied MSP Library(ies):	
Applied Taxonomy Tree:	Bruker Taxonomy

Rank (Quality)	Matched Pattern	Score Value	NCBI Identifier
1 (+++)	Amaranthus tuberculatus IOWA PI604247-2 MIRL	3	147656862
2 (+++)	Amaranthus tuberculatus IOWA PI604247-1 MIRL	2.74	147656862
3 (+++)	Amaranthus tuberculatus IOWA PI604247-3 MIRL	2.63	147656862
4 (+++)	Amaranthus tuberculatus IOWA PI674264-2 MIRL	2.37	147656862
5 (++)	Amaranthus tuberculatus IOWA PI674264-1 MIRL	2.26	147656862
6 (++)	Amaranthus tuberculatus IOWA PI674264-3 MIRL	2.18	147656862
7 (+)	Amaranthus rudis HARROW 1 MIRL	1.96	147656862
8 (+)	Amaranthus tuberculatus KANSAS PI 60743-3 MIRL	1.89	147656862
9 (+)	Amaranthus arenicola TEXAS PI667168-1 MIRL	1.77	147656862
10 (+)	Amaranthus tuberculatus KANSAS PI 60743-1 MIRL	1.7	147656862
11 (-)	Amaranthus tuberculatus KANSAS PI 60743-2 MIRL	1.68	147656862

12<br>**(-)**	Amaranthus rudis HARROW 2 MIRL	1.64	147656862
13<br>**(-)**	Amaranthus tuberculatus IOWA PI 553086-3 MIRL	1.6	147656862
14<br>**(-)**	Amaranthus tuberculatus IOWA PI 553086-1 MIRL	1.6	147656862
15<br>**(-)**	Amaranthus arenicola TEXAS PI667168-3 MIRL	1.56	147656862
16<br>**(-)**	Amaranthus rudis HARROW 3 MIRL	1.5	147656862
17<br>**(-)**	Amaranthus arenicola TEXAS PI667168-2 MIRL	1.48	147656862
18<br>**(-)**	Amaranthus tuberculatus IOWA PI 553086-2 MIRL	1.31	147656862
19<br>**(-)**	Amaranthus spinosus NC PI632248-RE3 MIRL	1.14	147656862
20<br>**(-)**	Amaranthus spinosus NC PI632248-RE2 MIRL	1.1	147656862

Analyte42

Analyte Name:	Amaranthus tuberculatus IOWA PI604247-3 MIRL
Analyte Description:	MSP
Analyte ID:	dbc1cb70-d00c-435e-afcb-1508bb3ec3eb
Analyte Creation Date/Time:	2022-10-27 2:52:21 PM
Applied MSP Library(ies):	
Applied Taxonomy Tree:	Bruker Taxonomy

Rank (Quality)	Matched Pattern	Score Value	NCBI Identifier
1 (+++)	Amaranthus tuberculatus IOWA PI604247-3 MIRL	3	147656862
2 (+++)	Amaranthus tuberculatus IOWA PI604247-2 MIRL	2.63	147656862
3 (+++)	Amaranthus tuberculatus IOWA PI604247-1 MIRL	2.53	147656862
4 (++)	Amaranthus tuberculatus IOWA PI674264-2 MIRL	2.28	147656862
5 (++)	Amaranthus tuberculatus IOWA PI674264-3 MIRL	2.18	147656862
6 (++)	Amaranthus tuberculatus IOWA PI674264-1 MIRL	2.04	147656862
7 (+)	Amaranthus tuberculatus KANSAS PI 60743-3 MIRL	1.82	147656862
8 (+)	Amaranthus rudis HARROW 1 MIRL	1.82	147656862
9 (+)	Amaranthus rudis HARROW 2 MIRL	1.8	147656862
10 (+)	Amaranthus tuberculatus IOWA PI 553086-3 MIRL	1.8	147656862
11 (+)	Amaranthus arenicola TEXAS PI667168-3 MIRL	1.79	147656862

12<br>(+)	Amaranthus arenicola TEXAS PI667168-1 MIRL	1.76	147656862
13<br>(+)	Amaranthus arenicola TEXAS PI667168-2 MIRL	1.72	147656862
14<br>(-)	Amaranthus tuberculatus KANSAS PI 60743-1 MIRL	1.54	147656862
15<br>(-)	Amaranthus rudis HARROW 3 MIRL	1.54	147656862
16<br>(-)	Amaranthus tuberculatus IOWA PI 553086-1 MIRL	1.43	147656862
17<br>(-)	Amaranthus tuberculatus KANSAS PI 60743-2 MIRL	1.37	147656862
18<br>(-)	Amaranthus tuberculatus IOWA PI 553086-2 MIRL	1.23	147656862
19<br>(-)	Amaranthus palmeri USA(AZ) PI632236-1 MIRL	1.17	147656862
20<br>(-)	Amaranthus arenicola KANSAS PI599671-1 MIRL	1.11	147656862

Analyte43

Analyte Name:	Amaranthus palmeri ARIZONA PI686461-1 MIRL
Analyte Description:	MSP
Analyte ID:	131892d6-87a7-4539-a29e-c87cd0831dcc
Analyte Creation Date/Time:	2022-11-09 1:30:47 PM
Applied MSP Library(ies):	
Applied Taxonomy Tree:	Bruker Taxonomy

Rank (Quality)	Matched Pattern	Score Value	NCBI Identifier
1 (+++)	Amaranthus palmeri ARIZONA PI686461-1 MIRL	3	147656862
2 (+++)	Amaranthus palmeri DAKAR PI633586-1 MIRL	2.69	147656862
3 (+++)	Amaranthus palmeri DAKAR PI633587-1 MIRL	2.62	147656862
4 (+++)	Amaranthus palmeri DAKAR PI633586-3 MIRL	2.58	147656862
5 (+++)	Amaranthus palmeri ARIZONA PI686461-3 MIRL	2.56	147656862
6 (+++)	Amaranthus palmeri DAKAR PI633587-2 MIRL	2.55	147656862
7 (+++)	Amaranthus palmeri DAKAR PI633587-3 MIRL	2.52	147656862
8 (+++)	Amaranthus palmeri DAKAR PI633586-2 MIRL	2.5	147656862
9 (+++)	Amaranthus palmeri ARIZONA PI686461-2 MIRL	2.42	147656862
10 (+++)	Amaranthus palmeri MALI PI549158-1 MIRL	2.37	147656862
11 (+++)	Amaranthus palmeri USA(AZ) PI632236-2 MIRL	2.33	147656862

12<br>(+++)	Amaranthus palmeri MALI PI549158-3 MIRL	2.33	147656862
13<br>(+++)	Amaranthus palmeri MALI PI549158-2 MIRL	2.33	147656862
14<br>(++)	Amaranthus palmeri MEXICO PUEBLA PI604557-2 MIRL	2.2	147656862
15<br>(++)	Amaranthus palmeri USA(AZ) PI632236-3 MIRL	2.13	147656862
16<br>(++)	Amaranthus palmeri MEXICO PUEBLA PI604557-3 MIRL	2.11	147656862
17<br>(++)	Amaranthus watsonii MEXICO COLIMA PI633593-RE2 MIRL	2.03	147656862
18<br>(+)	Amaranthus palmeri MEXICO PUEBLA PI604557-1 MIRL	1.97	147656862
19<br>(+)	Amaranthus watsonii MEXICO COLIMA PI633593-RE3 MIRL	1.84	147656862
20<br>(+)	Amaranthus palmeri MEXICO VERACRUZ PI667167-RE1 MIRL	1.83	147656862

Analyte44

Analyte Name:	Amaranthus palmeri ARIZONA PI686461-2 MIRL
Analyte Description:	MSP
Analyte ID:	11feaf47-6c6e-4cff-ad05-eae25114c817
Analyte Creation Date/Time:	2022-11-09 1:39:17 PM
Applied MSP Library(ies):	
Applied Taxonomy Tree:	Bruker Taxonomy

Rank (Quality)	Matched Pattern	Score Value	NCBI Identifier
1 (+++)	Amaranthus palmeri ARIZONA PI686461-2 MIRL	3	147656862
2 (+++)	Amaranthus palmeri DAKAR PI633586-2 MIRL	2.49	147656862
3 (+++)	Amaranthus palmeri DAKAR PI633586-1 MIRL	2.47	147656862
4 (+++)	Amaranthus palmeri ARIZONA PI686461-1 MIRL	2.42	147656862
5 (+++)	Amaranthus palmeri DAKAR PI633586-3 MIRL	2.42	147656862
6 (+++)	Amaranthus palmeri DAKAR PI633587-3 MIRL	2.42	147656862
7 (+++)	Amaranthus palmeri USA(AZ) PI632236-2 MIRL	2.41	147656862
8 (+++)	Amaranthus palmeri MALI PI549158-2 MIRL	2.39	147656862
9 (+++)	Amaranthus palmeri MALI PI549158-1 MIRL	2.36	147656862
10 (+++)	Amaranthus palmeri DAKAR PI633587-1 MIRL	2.33	147656862
11 (+++)	Amaranthus palmeri MALI PI549158-3 MIRL	2.32	147656862

12<br>(++)	Amaranthus palmeri DAKAR PI633587-2 MIRL	2.28	147656862
13<br>(++)	Amaranthus palmeri ARIZONA PI686461-3 MIRL	2.27	147656862
14<br>(++)	Amaranthus palmeri USA(AZ) PI632236-3 MIRL	2.26	147656862
15<br>(++)	Amaranthus watsonii MEXICO COLIMA PI633593-RE2 MIRL	2.25	147656862
16<br>(++)	Amaranthus palmeri MEXICO PUEBLA PI604557-3 MIRL	2.25	147656862
17<br>(++)	Amaranthus palmeri MEXICO PUEBLA PI604557-2 MIRL	2.16	147656862
18<br>(++)	Amaranthus palmeri MEXICO PUEBLA PI604557-1 MIRL	2.14	147656862
19<br>(++)	Amaranthus watsonii MEXICO COLIMA PI633593-RE3 MIRL	2.12	147656862
20<br>(++)	Amaranthus spinosus NC PI632248-RE3 MIRL	2.09	147656862

Analyte45

Analyte Name:	Amaranthus palmeri ARIZONA PI686461-3 MIRL
Analyte Description:	MSP
Analyte ID:	d5756841-e8d5-4c9e-9e4c-a1f7ef497ade
Analyte Creation Date/Time:	2022-11-09 1:45:52 PM
Applied MSP Library(ies):	
Applied Taxonomy Tree:	Bruker Taxonomy

Rank (Quality)	Matched Pattern	Score Value	NCBI Identifier
1 (+++)	Amaranthus palmeri ARIZONA PI686461-3 MIRL	3	147656862
2 (+++)	Amaranthus palmeri ARIZONA PI686461-1 MIRL	2.56	147656862
3 (+++)	Amaranthus palmeri DAKAR PI633587-3 MIRL	2.53	147656862
4 (+++)	Amaranthus palmeri DAKAR PI633587-1 MIRL	2.52	147656862
5 (+++)	Amaranthus palmeri DAKAR PI633586-1 MIRL	2.5	147656862
6 (+++)	Amaranthus palmeri DAKAR PI633587-2 MIRL	2.5	147656862
7 (+++)	Amaranthus palmeri DAKAR PI633586-3 MIRL	2.43	147656862
8 (+++)	Amaranthus palmeri DAKAR PI633586-2 MIRL	2.4	147656862
9 (++)	Amaranthus palmeri MALI PI549158-1 MIRL	2.28	147656862
10 (++)	Amaranthus palmeri ARIZONA PI686461-2 MIRL	2.27	147656862
11 (++)	Amaranthus palmeri USA(AZ) PI632236-2 MIRL	2.21	147656862

12<br>(++)	Amaranthus palmeri MALI PI549158-3 MIRL	2.18	147656862
13<br>(++)	Amaranthus palmeri MALI PI549158-2 MIRL	2.18	147656862
14<br>(++)	Amaranthus watsonii MEXICO COLIMA PI633593-RE2 MIRL	2.17	147656862
15<br>(++)	Amaranthus palmeri MEXICO PUEBLA PI604557-2 MIRL	2.11	147656862
16<br>(++)	Amaranthus palmeri MEXICO PUEBLA PI604557-3 MIRL	2.09	147656862
17<br>(++)	Amaranthus watsonii MEXICO COLIMA PI633593-RE3 MIRL	2.05	147656862
18<br>(++)	Amaranthus palmeri USA(AZ) PI632236-3 MIRL	2.05	147656862
19<br>(+)	Amaranthus palmeri USA(AZ) PI632236-1 MIRL	1.94	147656862
20<br>(+)	Amaranthus watsonii MEXICO COLIMA PI633593-RE1 MIRL	1.89	147656862

Analyte46

Analyte Name:	Amaranthus palmeri DAKAR PI633586-1 MIRL
Analyte Description:	MSP
Analyte ID:	bbab4a23-903f-4047-9841-871a7afbe03c
Analyte Creation Date/Time:	2022-11-09 1:53:48 PM
Applied MSP Library(ies):	
Applied Taxonomy Tree:	Bruker Taxonomy

Rank (Quality)	Matched Pattern	Score Value	NCBI Identifier
1 (+++)	Amaranthus palmeri DAKAR PI633586-1 MIRL	3	147656862
2 (+++)	Amaranthus palmeri DAKAR PI633586-3 MIRL	2.74	147656862
3 (+++)	Amaranthus palmeri ARIZONA PI686461-1 MIRL	2.69	147656862
4 (+++)	Amaranthus palmeri DAKAR PI633586-2 MIRL	2.62	147656862
5 (+++)	Amaranthus palmeri DAKAR PI633587-1 MIRL	2.54	147656862
6 (+++)	Amaranthus palmeri DAKAR PI633587-3 MIRL	2.53	147656862
7 (+++)	Amaranthus palmeri ARIZONA PI686461-3 MIRL	2.5	147656862
8 (+++)	Amaranthus palmeri ARIZONA PI686461-2 MIRL	2.47	147656862
9 (+++)	Amaranthus palmeri DAKAR PI633587-2 MIRL	2.46	147656862
10 (+++)	Amaranthus palmeri MALI PI549158-3 MIRL	2.4	147656862
11 (+++)	Amaranthus palmeri MALI PI549158-2 MIRL	2.37	147656862

12<br>(+++)	Amaranthus palmeri MALI PI549158-1 MIRL	2.35	147656862
13<br>(++)	Amaranthus watsonii MEXICO COLIMA PI633593-RE2 MIRL	2.22	147656862
14<br>(++)	Amaranthus palmeri USA(AZ) PI632236-2 MIRL	2.22	147656862
15<br>(++)	Amaranthus palmeri MEXICO PUEBLA PI604557-2 MIRL	2.22	147656862
16<br>(++)	Amaranthus palmeri MEXICO PUEBLA PI604557-3 MIRL	2.16	147656862
17<br>(++)	Amaranthus palmeri USA(AZ) PI632236-3 MIRL	2.08	147656862
18<br>(++)	Amaranthus palmeri USA(AZ) PI632236-1 MIRL	2	147656862
19<br>(+)	Amaranthus palmeri MEXICO VERACRUZ PI667167-RE3 MIRL	1.95	147656862
20<br>(+)	Amaranthus watsonii MEXICO COLIMA PI633593-RE1 MIRL	1.93	147656862

Analyte47

Analyte Name:	Amaranthus palmeri DAKAR PI633586-2 MIRL
Analyte Description:	MSP
Analyte ID:	1d98f949-8c2b-4989-aef5-aad717b3c2ba
Analyte Creation Date/Time:	2022-11-09 2:00:10 PM
Applied MSP Library(ies):	
Applied Taxonomy Tree:	Bruker Taxonomy

Rank (Quality)	Matched Pattern	Score Value	NCBI Identifier
1 (+++)	Amaranthus palmeri DAKAR PI633586-2 MIRL	3	147656862
2 (+++)	Amaranthus palmeri DAKAR PI633586-3 MIRL	2.77	147656862
3 (+++)	Amaranthus palmeri DAKAR PI633586-1 MIRL	2.63	147656862
4 (+++)	Amaranthus palmeri ARIZONA PI686461-1 MIRL	2.5	147656862
5 (+++)	Amaranthus palmeri ARIZONA PI686461-2 MIRL	2.5	147656862
6 (+++)	Amaranthus palmeri DAKAR PI633587-1 MIRL	2.42	147656862
7 (+++)	Amaranthus palmeri ARIZONA PI686461-3 MIRL	2.4	147656862
8 (+++)	Amaranthus palmeri DAKAR PI633587-3 MIRL	2.39	147656862
9 (+++)	Amaranthus palmeri USA(AZ) PI632236-2 MIRL	2.36	147656862
10 (+++)	Amaranthus palmeri DAKAR PI633587-2 MIRL	2.3	147656862
11 (++)	Amaranthus watsonii MEXICO COLIMA PI633593-RE2 MIRL	2.29	147656862

12<br>(++)	Amaranthus palmeri MALI PI549158-3 MIRL	2.29	147656862
13<br>(++)	Amaranthus palmeri MALI PI549158-1 MIRL	2.28	147656862
14<br>(++)	Amaranthus palmeri MALI PI549158-2 MIRL	2.25	147656862
15<br>(++)	Amaranthus palmeri USA(AZ) PI632236-3 MIRL	2.23	147656862
16<br>(++)	Amaranthus palmeri MEXICO PUEBLA PI604557-3 MIRL	2.16	147656862
17<br>(++)	Amaranthus palmeri MEXICO VERACRUZ PI667167-RE1 MIRL	2.12	147656862
18<br>(++)	Amaranthus palmeri MEXICO PUEBLA PI604557-2 MIRL	2.07	147656862
19<br>(++)	Amaranthus palmeri USA(AZ) PI632236-1 MIRL	2.02	147656862
20<br>(++)	Amaranthus watsonii MEXICO COLIMA PI633593-RE1 MIRL	2.01	147656862

Analyte48

Analyte Name:	Amaranthus palmeri DAKAR PI633586-3 MIRL
Analyte Description:	MSP
Analyte ID:	1933c5c3-140c-4bc8-a220-62a38148331a
Analyte Creation Date/Time:	2022-11-09 2:05:55 PM
Applied MSP Library(ies):	
Applied Taxonomy Tree:	Bruker Taxonomy

Rank (Quality)	Matched Pattern	Score Value	NCBI Identifier
1 (+++)	Amaranthus palmeri DAKAR PI633586-3 MIRL	3	147656862
2 (+++)	Amaranthus palmeri DAKAR PI633586-2 MIRL	2.77	147656862
3 (+++)	Amaranthus palmeri DAKAR PI633586-1 MIRL	2.74	147656862
4 (+++)	Amaranthus palmeri ARIZONA PI686461-1 MIRL	2.59	147656862
5 (+++)	Amaranthus palmeri DAKAR PI633587-3 MIRL	2.52	147656862
6 (+++)	Amaranthus palmeri DAKAR PI633587-1 MIRL	2.51	147656862
7 (+++)	Amaranthus palmeri ARIZONA PI686461-3 MIRL	2.44	147656862
8 (+++)	Amaranthus palmeri ARIZONA PI686461-2 MIRL	2.42	147656862
9 (+++)	Amaranthus palmeri MALI PI549158-2 MIRL	2.37	147656862
10 (+++)	Amaranthus palmeri MALI PI549158-1 MIRL	2.32	147656862
11 (+++)	Amaranthus palmeri DAKAR PI633587-2 MIRL	2.3	147656862

12<br>(++)	Amaranthus palmeri USA(AZ) PI632236-2 MIRL	2.25	147656862
13<br>(++)	Amaranthus watsonii MEXICO COLIMA PI633593-RE2 MIRL	2.21	147656862
14<br>(++)	Amaranthus palmeri USA(AZ) PI632236-3 MIRL	2.2	147656862
15<br>(++)	Amaranthus palmeri MALI PI549158-3 MIRL	2.2	147656862
16<br>(++)	Amaranthus palmeri MEXICO PUEBLA PI604557-3 MIRL	2.17	147656862
17<br>(++)	Amaranthus palmeri MEXICO PUEBLA PI604557-2 MIRL	2.15	147656862
18<br>(++)	Amaranthus palmeri MEXICO VERACRUZ PI667167-RE1 MIRL	2.07	147656862
19<br>(+)	Amaranthus watsonii MEXICO COLIMA PI633593-RE1 MIRL	1.93	147656862
20<br>(+)	Amaranthus watsonii MEXICO COLIMA PI633593-RE3 MIRL	1.86	147656862

Analyte49

Analyte Name:	Amaranthus caudatus NJ AMES 5687-1 MIRL
Analyte Description:	MSP
Analyte ID:	324643be-d5f3-4091-8407-7817c6f06d9d
Analyte Creation Date/Time:	2022-11-09 2:14:57 PM
Applied MSP Library(ies):	
Applied Taxonomy Tree:	Bruker Taxonomy

Rank (Quality)	Matched Pattern	Score Value	NCBI Identifier
1 (+++)	Amaranthus caudatus NJ AMES 5687-1 MIRL	3	147656862
2 (+++)	Amaranthus caudatus NJ AMES 5687-2 MIRL	2.63	147656862
3 (+++)	Amaranthus caudatus NJ AMES 5687-3 MIRL	2.56	147656862
4 (+++)	Amaranthus hypochondriacus PI658730-1 MIRL	2.5	147656862
5 (+++)	Amaranthus hypochondriacus PI658730-2 MIRL	2.49	147656862
6 (+++)	Amaranthus hypochondriacus PI658730-3 MIRL	2.49	147656862
7 (+++)	Amaranthus hybridus PUERTO RICO AMES 5152-3 MIRL	2.48	147656862
8 (+++)	Amaranthus hybridus PUERTO RICO AMES 5152-1 MIRL	2.4	147656862
9 (+++)	Amaranthus hypochondriacus MEXICO SONORA PI599682-1 MIRL	2.37	147656862
10 (+++)	Amaranthus hybridus HARROW 3 MIRL	2.36	147656862
11 (+++)	Amaranthus hybridus PUERTO RICO AMES 5152-2 MIRL	2.34	147656862

12<br>(+++)	Amaranthus hypochondriacus MISSOURI PI698341-1 MIRL	2.3	147656862
13<br>(++)	Amaranthus hybridus HARROW 2 MIRL	2.29	147656862
14<br>(++)	Amaranthus hypochondriacus MEXICO SONORA PI599682-2 MIRL	2.16	147656862
15<br>(++)	Amaranthus hypochondriacus MISSOURI PI698341-3 MIRL	2.14	147656862
16<br>(++)	Amaranthus hypochondriacus MISSOURI PI698341-2 MIRL	2.09	147656862
17<br>(++)	Amaranthus hypochondriacus MEXICO SONORA PI599682-3 MIRL	2.04	147656862
18<br>(++)	Amaranthus hybridus HARROW 1 MIRL	2	147656862
19<br>(+)	Amaranthus hybridus INDIANA PI603895-2 MIRL	1.74	147656862
20<br>(+)	Amaranthus caudatus NJ PI553073-3 MIRL	1.72	147656862

Analyte50

Analyte Name: Amaranthus caudatus NJ AMES 5687-2 MIRL
Analyte Description: MSP
Analyte ID: 6a88c238-6b2c-4a86-93ad-49d5fa0126a6
Analyte Creation Date/Time: 2022-11-09 2:21:10 PM
Applied MSP Library(ies):
Applied Taxonomy Tree: Bruker Taxonomy

Rank (Quality)	Matched Pattern	Score Value	NCBI Identifier
1 (+++)	Amaranthus caudatus NJ AMES 5687-2 MIRL	3	147656862
2 (+++)	Amaranthus caudatus NJ AMES 5687-3 MIRL	2.76	147656862
3 (+++)	Amaranthus caudatus NJ AMES 5687-1 MIRL	2.63	147656862
4 (+++)	Amaranthus hypochondriacus PI658730-2 MIRL	2.42	147656862
5 (+++)	Amaranthus hypochondriacus PI658730-1 MIRL	2.41	147656862
6 (+++)	Amaranthus hybridus HARROW 3 MIRL	2.37	147656862
7 (+++)	Amaranthus hybridus HARROW 2 MIRL	2.35	147656862
8 (+++)	Amaranthus hybridus PUERTO RICO AMES 5152-3 MIRL	2.34	147656862
9 (++)	Amaranthus hypochondriacus PI658730-3 MIRL	2.29	147656862
10 (++)	Amaranthus hypochondriacus MEXICO SONORA PI599682-1 MIRL	2.29	147656862
11 (++)	Amaranthus hybridus HARROW 1 MIRL	2.27	147656862

12<br>(++)	Amaranthus hybridus PUERTO RICO AMES 5152-2 MIRL	2.25	147656862
13<br>(++)	Amaranthus hybridus PUERTO RICO AMES 5152-1 MIRL	2.24	147656862
14<br>(++)	Amaranthus hypochondriacus MISSOURI PI698341-1 MIRL	2.22	147656862
15<br>(++)	Amaranthus hypochondriacus MEXICO SONORA PI599682-2 MIRL	2.21	147656862
16<br>(++)	Amaranthus hypochondriacus MISSOURI PI698341-3 MIRL	2.06	147656862
17<br>(++)	Amaranthus caudatus CALIFORNIA PI690570-1 MIRL	2.04	147656862
18<br>(+)	Amaranthus hypochondriacus MISSOURI PI698341-2 MIRL	1.99	147656862
19<br>(+)	Amaranthus caudatus CALIFORNIA PI690570-2 MIRL	1.99	147656862
20<br>(+)	Amaranthus caudatus CALIFORNIA PI690570-3 MIRL	1.94	147656862

Analyte51

Analyte Name:	Amaranthus caudatus NJ AMES 5687-3 MIRL
Analyte Description:	MSP
Analyte ID:	88dd1468-e257-4506-80b7-00d6481c20eb
Analyte Creation Date/Time:	2022-11-09 2:27:04 PM
Applied MSP Library(ies):	
Applied Taxonomy Tree:	Bruker Taxonomy

Rank (Quality)	Matched Pattern	Score Value	NCBI Identifier
1 (+++)	Amaranthus caudatus NJ AMES 5687-3 MIRL	3	147656862
2 (+++)	Amaranthus caudatus NJ AMES 5687-2 MIRL	2.76	147656862
3 (+++)	Amaranthus caudatus NJ AMES 5687-1 MIRL	2.56	147656862
4 (+++)	Amaranthus hypochondriacus PI658730-2 MIRL	2.33	147656862
5 (+++)	Amaranthus hybridus PUERTO RICO AMES 5152-3 MIRL	2.32	147656862
6 (++)	Amaranthus hybridus HARROW 3 MIRL	2.28	147656862
7 (++)	Amaranthus hypochondriacus PI658730-1 MIRL	2.27	147656862
8 (++)	Amaranthus hybridus HARROW 2 MIRL	2.26	147656862
9 (++)	Amaranthus hypochondriacus MEXICO SONORA PI599682-2 MIRL	2.21	147656862
10 (++)	Amaranthus hypochondriacus MEXICO SONORA PI599682-1 MIRL	2.21	147656862
11 (++)	Amaranthus hypochondriacus PI658730-3 MIRL	2.2	147656862

12<br>(++)	Amaranthus caudatus CALIFORNIA PI690570-1 MIRL	2.17	147656862
13<br>(++)	Amaranthus hybridus PUERTO RICO AMES 5152-2 MIRL	2.16	147656862
14<br>(++)	Amaranthus hypochondriacus MISSOURI PI698341-1 MIRL	2.14	147656862
15<br>(++)	Amaranthus caudatus CALIFORNIA PI690570-3 MIRL	2.14	147656862
16<br>(++)	Amaranthus hybridus HARROW 1 MIRL	2.14	147656862
17<br>(++)	Amaranthus hybridus PUERTO RICO AMES 5152-1 MIRL	2.13	147656862
18<br>(++)	Amaranthus caudatus CALIFORNIA PI690570-2 MIRL	2.1	147656862
19<br>(+)	Amaranthus hypochondriacus MISSOURI PI698341-3 MIRL	1.99	147656862
20<br>(+)	Amaranthus hypochondriacus MISSOURI PI698341-2 MIRL	1.93	147656862

Analyte52

Analyte Name:	Amaranthus blitoides CANADA PI608663-1 MIRL
Analyte Description:	MSP
Analyte ID:	2e797bae-7a47-4158-951f-777ca549c4fc
Analyte Creation Date/Time:	2022-11-22 1:33:03 PM
Applied MSP Library(ies):	
Applied Taxonomy Tree:	Bruker Taxonomy

Rank (Quality)	Matched Pattern	Score Value	NCBI Identifier
1 (+++)	Amaranthus blitoides CANADA PI608663-1 MIRL	3	147656862
2 (+++)	Amaranthus blitoides CANADA PI608663-2 MIRL	2.78	147656862
3 (+++)	Amaranthus blitoides HARROW 2 MIRL	2.48	147656862
4 (+++)	Amaranthus blitoides HARROW 3 MIRL	2.4	147656862
5 (++)	Amaranthus blitoides CANADA PI608663-3 MIRL	2.24	147656862
6 (-)	Amaranthus albus NC PI632244-1 MIRL	1.5	147656862
7 (-)	Amaranthus tricolor NJ AMES 5303-2 MIRL	1.48	147656862
8 (-)	Amaranthus albus HARROW 2 MIRL	1.47	147656862
9 (-)	Amaranthus tricolor NJ AMES 5303-3 MIRL	1.47	147656862
10 (-)	Amaranthus albus PI633580-1 MIRL	1.43	147656862
11 (-)	Amaranthus tricolor NJ AMES 5303-1 MIRL	1.43	147656862

12<br>(-)	Amaranthus albus HARROW 1 MIRL	1.41	147656862
13<br>(-)	Amaranthus hypochondriacus MEXICO SONORA PI599682-2 MIRL	1.37	147656862
14<br>(-)	Amaranthus albus WASHINGTON PI654389-1 MIRL	1.37	147656862
15<br>(-)	Amaranthus albus NC PI632244-3 MIRL	1.32	147656862
16<br>(-)	Amaranthus californicus CALIFORNIA PI595319-2 MIRL	1.28	147656862
17<br>(-)	Amaranthus hypochondriacus MEXICO SONORA PI599682-1 MIRL	1.25	147656862
18<br>(-)	Amaranthus albus WASHINGTON PI654389-3 MIRL	1.22	147656862
19<br>(-)	Amaranthus hypochondriacus MISSOURI PI698341-3 MIRL	1.21	147656862
20<br>(-)	Amaranthus albus HARROW 3 MIRL	1.2	147656862

Analyte53

Analyte Name:	Amaranthus blitoides CANADA PI608663-2 MIRL
Analyte Description:	MSP
Analyte ID:	51977c1d-d685-4e39-abda-3e0553d856a8
Analyte Creation Date/Time:	2022-11-22 1:40:40 PM
Applied MSP Library(ies):	
Applied Taxonomy Tree:	Bruker Taxonomy

Rank (Quality)	Matched Pattern	Score Value	NCBI Identifier
1 (+++)	Amaranthus blitoides CANADA PI608663-2 MIRL	3	147656862
2 (+++)	Amaranthus blitoides CANADA PI608663-1 MIRL	2.78	147656862
3 (+++)	Amaranthus blitoides HARROW 2 MIRL	2.6	147656862
4 (+++)	Amaranthus blitoides HARROW 3 MIRL	2.57	147656862
5 (+++)	Amaranthus blitoides CANADA PI608663-3 MIRL	2.33	147656862
6 (-)	Amaranthus albus HARROW 2 MIRL	1.48	147656862
7 (-)	Amaranthus albus HARROW 1 MIRL	1.48	147656862
8 (-)	Amaranthus californicus CALIFORNIA PI595319-2 MIRL	1.43	147656862
9 (-)	Amaranthus albus NC PI632244-1 MIRL	1.39	147656862
10 (-)	Amaranthus albus WASHINGTON PI654389-3 MIRL	1.36	147656862
11 (-)	Amaranthus tricolor NJ AMES 5303-3 MIRL	1.34	147656862

12<br>(-)	Amaranthus albus PI633580-3 MIRL	1.34	147656862
13<br>(-)	Amaranthus albus WASHINGTON PI654389-1 MIRL	1.32	147656862
14<br>(-)	Amaranthus hypochondriacus MEXICO SONORA PI599682-2 MIRL	1.32	147656862
15<br>(-)	Amaranthus hypochondriacus MEXICO SONORA PI599682-1 MIRL	1.31	147656862
16<br>(-)	Amaranthus albus NC PI632244-3 MIRL	1.31	147656862
17<br>(-)	Amaranthus albus HARROW 3 MIRL	1.3	147656862
18<br>(-)	Amaranthus tricolor NJ AMES 5303-1 MIRL	1.3	147656862
19<br>(-)	Amaranthus albus NC PI632244-2 MIRL	1.29	147656862
20<br>(-)	Amaranthus hybridus INDIANA PI603895-2 MIRL	1.26	147656862

Analyte54

Analyte Name: Amaranthus blitoides CANADA PI608663-3 MIRL
Analyte Description: MSP
Analyte ID: 2ab930f1-006e-4872-b476-5bb04088a55f
Analyte Creation Date/Time: 2022-11-22 1:46:27 PM
Applied MSP Library(ies):
Applied Taxonomy Tree: Bruker Taxonomy

Rank (Quality)	Matched Pattern	Score Value	NCBI Identifier
1 (+++)	Amaranthus blitoides CANADA PI608663-3 MIRL	3	147656862
2 (+++)	Amaranthus blitoides HARROW 3 MIRL	2.44	147656862
3 (+++)	Amaranthus blitoides CANADA PI608663-2 MIRL	2.33	147656862
4 (+++)	Amaranthus blitoides HARROW 2 MIRL	2.32	147656862
5 (++)	Amaranthus blitoides CANADA PI608663-1 MIRL	2.24	147656862
6 (-)	Amaranthus albus NC PI632244-1 MIRL	1.65	147656862
7 (-)	Amaranthus albus NC PI632244-2 MIRL	1.56	147656862
8 (-)	Amaranthus albus HARROW 3 MIRL	1.52	147656862
9 (-)	Amaranthus albus WASHINGTON PI654389-1 MIRL	1.5	147656862
10 (-)	Amaranthus albus WASHINGTON PI654389-3 MIRL	1.49	147656862
11 (-)	Amaranthus albus HARROW 2 MIRL	1.48	147656862

12<br>**(-)**	Amaranthus albus HARROW 1 MIRL	1.47	147656862
13<br>**(-)**	Amaranthus albus NC PI632244-3 MIRL	1.46	147656862
14<br>**(-)**	Amaranthus albus WASHINGTON PI654389-2 MIRL	1.45	147656862
15<br>**(-)**	Amaranthus albus PI633580-3 MIRL	1.45	147656862
16<br>**(-)**	Amaranthus californicus CALIFORNIA PI595319-2 MIRL	1.4	147656862
17<br>**(-)**	Amaranthus albus PI633580-1 MIRL	1.4	147656862
18<br>**(-)**	Amaranthus tricolor NJ AMES 5303-3 MIRL	1.28	147656862
19<br>**(-)**	Amaranthus tricolor NJ AMES 5303-2 MIRL	1.18	147656862
20<br>**(-)**	Amaranthus palmeri MALI PI549158-3 MIRL	1.16	147656862

Analyte55

Analyte Name:	Amaranthus viridis FLORIDA PI654388-1 MIRL
Analyte Description:	MSP
Analyte ID:	f5da62c0-f74d-47d5-9143-8334b3c2761f
Analyte Creation Date/Time:	2022-11-22 1:53:17 PM
Applied MSP Library(ies):	
Applied Taxonomy Tree:	Bruker Taxonomy

Rank (Quality)	Matched Pattern	Score Value	NCBI Identifier
1 (+++)	Amaranthus viridis FLORIDA PI654388-1 MIRL	3	147656862
2 (+++)	Amaranthus viridis FLORIDA PI654388-2 MIRL	2.88	147656862
3 (+++)	Amaranthus viridis FLORIDA PI654388-3 MIRL	2.87	147656862
4 (-)	Amaranthus blitum var. pseudogracilis NC PI632245-3 MIRL	1.22	147656862
5 (-)	Amaranthus blitum var. pseudogracilis NC PI632245-2 MIRL	1.16	147656862
6 (-)	Amaranthus blitum var. pseudogracilis NC PI632245-1 MIRL	1.15	147656862
7 (-)	Amaranthus rudis HARROW 2 MIRL	1.1	147656862
8 (-)	Amaranthus palmeri MEXICO VERACRUZ PI667167-1 MIRL	0.97	147656862
9 (-)	Amaranthus palmeri DAKAR PI633586-3 MIRL	0.97	147656862
10 (-)	Amaranthus palmeri MEXICO VERACRUZ PI667167-RE1 MIRL	0.96	147656862
11 (-)	Amaranthus albus NC PI632244-2 MIRL	0.93	147656862

12<br>(-)	Amaranthus retroflexus ONTARIO AMES 35199-1 MIRL	0.88	147656862
13<br>(-)	Amaranthus palmeri DAKAR PI633586-2 MIRL	0.87	147656862
14<br>(-)	Amaranthus spinosus NC PI632248-RE2 MIRL	0.86	147656862
15<br>(-)	Amaranthus palmeri DAKAR PI633586-1 MIRL	0.85	147656862
16<br>(-)	Amaranthus powellii sub. Powellii NM PI649309-1 MIRL	0.85	147656862
17<br>(-)	Amaranthus albus HARROW 2 MIRL	0.84	147656862
18<br>(-)	Amaranthus albus HARROW 3 MIRL	0.81	147656862
19<br>(-)	Amaranthus retroflexus UTAH PI612857-3 MIRL	0.8	147656862
20<br>(-)	Amaranthus tuberculatus IOWA PI604247-1 MIRL	0.76	147656862

Analyte56

Analyte Name:	Amaranthus viridis FLORIDA PI654388-2 MIRL
Analyte Description:	MSP
Analyte ID:	d3981f60-6a15-4a7a-991e-767a3c214019
Analyte Creation Date/Time:	2022-11-22 2:03:42 PM
Applied MSP Library(ies):	
Applied Taxonomy Tree:	Bruker Taxonomy

Rank (Quality)	Matched Pattern	Score Value	NCBI Identifier
1 (+++)	Amaranthus viridis FLORIDA PI654388-2 MIRL	3	147656862
2 (+++)	Amaranthus viridis FLORIDA PI654388-3 MIRL	2.93	147656862
3 (+++)	Amaranthus viridis FLORIDA PI654388-1 MIRL	2.88	147656862
4 (-)	Amaranthus blitum var. pseudogracilis NC PI632245-3 MIRL	1.26	147656862
5 (-)	Amaranthus blitum var. pseudogracilis NC PI632245-1 MIRL	1.25	147656862
6 (-)	Amaranthus blitum var. pseudogracilis NC PI632245-2 MIRL	1.21	147656862
7 (-)	Amaranthus hypochondriacus MEXICO SONORA PI599682-1 MIRL	0.96	147656862
8 (-)	Amaranthus hybridus PUERTO RICO AMES 5152-3 MIRL	0.94	147656862
9 (-)	Amaranthus hybridus PUERTO RICO AMES 5152-1 MIRL	0.9	147656862
10 (-)	Amaranthus tuberculatus IOWA PI674264-3 MIRL	0.9	147656862
11 (-)	Amaranthus retroflexus ONTARIO AMES 35199-1 MIRL	0.88	147656862

12<br>(-)	Amaranthus albus PI633580-1 MIRL	0.87	147656862
13<br>(-)	Amaranthus palmeri DAKAR PI633586-1 MIRL	0.86	147656862
14<br>(-)	Amaranthus tuberculatus IOWA PI 553086-1 MIRL	0.86	147656862
15<br>(-)	Amaranthus retroflexus UTAH PI612857-1 MIRL	0.84	147656862
16<br>(-)	Amaranthus rudis HARROW 2 MIRL	0.84	147656862
17<br>(-)	Amaranthus powellii sub. Powellii NM PI649309-1 MIRL	0.78	147656862
18<br>(-)	Amaranthus albus PI633580-3 MIRL	0.74	147656862
19<br>(-)	Amaranthus albus HARROW 3 MIRL	0.73	147656862
20<br>(-)	Amaranthus spinosus PI 632248-1 MIRL	0.72	147656862

Analyte57

Analyte Name:	Amaranthus viridis FLORIDA PI654388-3 MIRL
Analyte Description:	MSP
Analyte ID:	68a76006-dbe0-4395-8b91-b205a8f5f36b
Analyte Creation Date/Time:	2022-11-22 2:10:13 PM
Applied MSP Library(ies):	
Applied Taxonomy Tree:	Bruker Taxonomy

Rank (Quality)	Matched Pattern	Score Value	NCBI Identifier
1 (+++)	Amaranthus viridis FLORIDA PI654388-3 MIRL	3	147656862
2 (+++)	Amaranthus viridis FLORIDA PI654388-2 MIRL	2.93	147656862
3 (+++)	Amaranthus viridis FLORIDA PI654388-1 MIRL	2.87	147656862
4 (-)	Amaranthus blitum var. pseudogracilis NC PI632245-3 MIRL	1.25	147656862
5 (-)	Amaranthus blitum var. pseudogracilis NC PI632245-1 MIRL	1.24	147656862
6 (-)	Amaranthus blitum var. pseudogracilis NC PI632245-2 MIRL	1.23	147656862
7 (-)	Amaranthus albus PI633580-1 MIRL	1.18	147656862
8 (-)	Amaranthus albus WASHINGTON PI654389-2 MIRL	1.05	147656862
9 (-)	Amaranthus hybridus PUERTO RICO AMES 5152-3 MIRL	0.98	147656862
10 (-)	Amaranthus rudis HARROW 2 MIRL	0.96	147656862
11 (-)	Amaranthus albus PI633580-3 MIRL	0.94	147656862

12<br>(-)	Amaranthus hybridus PUERTO RICO AMES 5152-1 MIRL	0.92	147656862
13<br>(-)	Amaranthus retroflexus UTAH PI612857-2 MIRL	0.83	147656862
14<br>(-)	Amaranthus hypochondriacus PI658730-1 MIRL	0.83	147656862
15<br>(-)	Amaranthus retroflexus ONTARIO AMES 35199-1 MIRL	0.81	147656862
16<br>(-)	Amaranthus albus HARROW 3 MIRL	0.8	147656862
17<br>(-)	Amaranthus tuberculatus IOWA PI674264-3 MIRL	0.79	147656862
18<br>(-)	Amaranthus tricolor NJ AMES 5303-3 MIRL	0.78	147656862
19<br>(-)	Amaranthus hypochondriacus PI658730-2 MIRL	0.78	147656862
20<br>(-)	Amaranthus albus WASHINGTON PI654389-1 MIRL	0.75	147656862

Analyte58

Analyte Name: Amaranthus blitum var. pseudogracilis NC PI632245-1 MIRL
Analyte Description: MSP
Analyte ID: 1e68c50e-4323-4426-b2c2-29ac6316eaf9
Analyte Creation Date/Time: 2022-11-22 2:21:08 PM
Applied MSP Library(ies):
Applied Taxonomy Tree: Bruker Taxonomy

Rank (Quality)	Matched Pattern	Score Value	NCBI Identifier
1 (+++)	Amaranthus blitum var. pseudogracilis NC PI632245-1 MIRL	3	147656862
2 (+++)	Amaranthus blitum var. pseudogracilis NC PI632245-2 MIRL	2.89	147656862
3 (+++)	Amaranthus blitum var. pseudogracilis NC PI632245-3 MIRL	2.82	147656862
4 (-)	Amaranthus viridis FLORIDA PI654388-2 MIRL	1.25	147656862
5 (-)	Amaranthus viridis FLORIDA PI654388-3 MIRL	1.24	147656862
6 (-)	Amaranthus tuberculatus IOWA PI674264-3 MIRL	1.21	147656862
7 (-)	Amaranthus viridis FLORIDA PI654388-1 MIRL	1.15	147656862
8 (-)	Amaranthus caudatus NJ AMES 5687-2 MIRL	1.1	147656862
9 (-)	Amaranthus hypochondriacus PI658730-1 MIRL	1.09	147656862
10 (-)	Amaranthus hypochondriacus MISSOURI PI698341-1 MIRL	1.08	147656862
11 (-)	Amaranthus tuberculatus IOWA PI674264-2 MIRL	1.06	147656862

12<br>(-)	Amaranthus tuberculatus IOWA PI604247-1 MIRL	1.04	147656862
13<br>(-)	Amaranthus albus PI633580-3 MIRL	1.01	147656862
14<br>(-)	Amaranthus caudatus NJ AMES 5687-3 MIRL	0.99	147656862
15<br>(-)	Amaranthus hypochondriacus PI658730-2 MIRL	0.98	147656862
16<br>(-)	Amaranthus spinosus NC PI632248-RE3 MIRL	0.96	147656862
17<br>(-)	Amaranthus albus PI633580-1 MIRL	0.93	147656862
18<br>(-)	Amaranthus hybridus PUERTO RICO AMES 5152-2 MIRL	0.91	147656862
19<br>(-)	Amaranthus californicus CALIFORNIA PI595319-1 MIRL	0.91	147656862
20<br>(-)	Amaranthus hypochondriacus MISSOURI PI698341-2 MIRL	0.9	147656862

Analyte59

Analyte Name:	Amaranthus blitum var. pseudogracilis NC PI632245-2 MIRL
Analyte Description:	MSP
Analyte ID:	05817581-f36e-46eb-b1f2-d9c2ab89f5e0
Analyte Creation Date/Time:	2022-11-22 2:26:33 PM
Applied MSP Library(ies):	
Applied Taxonomy Tree:	Bruker Taxonomy

Rank (Quality)	Matched Pattern	Score Value	NCBI Identifier
1 (+++)	Amaranthus blitum var. pseudogracilis NC PI632245-2 MIRL	3	147656862
2 (+++)	Amaranthus blitum var. pseudogracilis NC PI632245-1 MIRL	2.89	147656862
3 (+++)	Amaranthus blitum var. pseudogracilis NC PI632245-3 MIRL	2.83	147656862
4 (-)	Amaranthus viridis FLORIDA PI654388-3 MIRL	1.23	147656862
5 (-)	Amaranthus viridis FLORIDA PI654388-2 MIRL	1.21	147656862
6 (-)	Amaranthus tuberculatus IOWA PI674264-2 MIRL	1.19	147656862
7 (-)	Amaranthus viridis FLORIDA PI654388-1 MIRL	1.16	147656862
8 (-)	Amaranthus tuberculatus IOWA PI604247-1 MIRL	1.09	147656862
9 (-)	Amaranthus hypochondriacus PI658730-1 MIRL	1.05	147656862
10 (-)	Amaranthus albus PI633580-3 MIRL	1.04	147656862
11 (-)	Amaranthus hypochondriacus PI658730-2 MIRL	1.02	147656862

12<br>(-)	Amaranthus albus PI633580-1 MIRL	0.99	147656862
13<br>(-)	Amaranthus hypochondriacus MISSOURI PI698341-1 MIRL	0.97	147656862
14<br>(-)	Amaranthus tuberculatus IOWA PI674264-3 MIRL	0.95	147656862
15<br>(-)	Amaranthus hypochondriacus MISSOURI PI698341-2 MIRL	0.93	147656862
16<br>(-)	Amaranthus hybridus PUERTO RICO AMES 5152-2 MIRL	0.92	147656862
17<br>(-)	Amaranthus albus NC PI632244-2 MIRL	0.91	147656862
18<br>(-)	Amaranthus tuberculatus IOWA PI674264-1 MIRL	0.91	147656862
19<br>(-)	Amaranthus hybridus HARROW 2 MIRL	0.9	147656862
20<br>(-)	Amaranthus tuberculatus KANSAS PI 60743-3 MIRL	0.88	147656862

Analyte60

Analyte Name:	Amaranthus blitum var. pseudogracilis NC PI632245-3 MIRL
Analyte Description:	MSP
Analyte ID:	cf5c577c-b934-4896-8f77-c44d88e5af5d
Analyte Creation Date/Time:	2022-11-22 2:33:13 PM
Applied MSP Library(ies):	
Applied Taxonomy Tree:	Bruker Taxonomy

Rank (Quality)	Matched Pattern	Score Value	NCBI Identifier
1 (+++)	Amaranthus blitum var. pseudogracilis NC PI632245-3 MIRL	3	147656862
2 (+++)	Amaranthus blitum var. pseudogracilis NC PI632245-2 MIRL	2.83	147656862
3 (+++)	Amaranthus blitum var. pseudogracilis NC PI632245-1 MIRL	2.82	147656862
4 (-)	Amaranthus viridis FLORIDA PI654388-2 MIRL	1.26	147656862
5 (-)	Amaranthus viridis FLORIDA PI654388-3 MIRL	1.26	147656862
6 (-)	Amaranthus viridis FLORIDA PI654388-1 MIRL	1.25	147656862
7 (-)	Amaranthus albus NC PI632244-1 MIRL	1.14	147656862
8 (-)	Amaranthus tuberculatus IOWA PI604247-1 MIRL	1.14	147656862
9 (-)	Amaranthus spinosus NC PI632248-RE2 MIRL	1.11	147656862
10 (-)	Amaranthus tuberculatus IOWA PI674264-2 MIRL	1.11	147656862
11 (-)	Amaranthus albus NC PI632244-2 MIRL	1.09	147656862

12<br>**(-)**	Amaranthus caudatus NJ AMES 5687-2 MIRL	1.09	147656862
13<br>**(-)**	Amaranthus tuberculatus KANSAS PI 60743-3 MIRL	1.03	147656862
14<br>**(-)**	Amaranthus tuberculatus IOWA PI674264-3 MIRL	1.03	147656862
15<br>**(-)**	Amaranthus retroflexus UTAH PI612857-3 MIRL	1	147656862
16<br>**(-)**	Amaranthus caudatus NJ AMES 5687-3 MIRL	0.99	147656862
17<br>**(-)**	Amaranthus palmeri DAKAR PI633587-1 MIRL	0.99	147656862
18<br>**(-)**	Amaranthus hypochondriacus MISSOURI PI698341-2 MIRL	0.98	147656862
19<br>**(-)**	Amaranthus californicus CALIFORNIA PI595319-1 MIRL	0.97	147656862
20<br>**(-)**	Amaranthus hypochondriacus MISSOURI PI698341-1 MIRL	0.93	147656862

Analyte61

Analyte Name:	Amaranthus tricolor NJ AMES 5303-1 MIRL
Analyte Description:	MSP
Analyte ID:	df1738de-01db-480e-8d68-eb12358c1f52
Analyte Creation Date/Time:	2022-11-24 2:59:28 PM
Applied MSP Library(ies):	
Applied Taxonomy Tree:	Bruker Taxonomy

Rank (Quality)	Matched Pattern	Score Value	NCBI Identifier
1 (+++)	Amaranthus tricolor NJ AMES 5303-1 MIRL	3	147656862
2 (+++)	Amaranthus tricolor NJ AMES 5303-3 MIRL	2.78	147656862
3 (+++)	Amaranthus tricolor NJ AMES 5303-2 MIRL	2.73	147656862
4 (-)	Amaranthus blitoides CANADA PI608663-1 MIRL	1.43	147656862
5 (-)	Amaranthus blitoides HARROW 2 MIRL	1.34	147656862
6 (-)	Amaranthus blitoides HARROW 3 MIRL	1.33	147656862
7 (-)	Amaranthus blitoides CANADA PI608663-2 MIRL	1.3	147656862
8 (-)	Amaranthus albus PI633580-1 MIRL	1.23	147656862
9 (-)	Amaranthus retroflexus CANADA AMES 5328-2 MIRL	1.21	147656862
10 (-)	Amaranthus retroflexus HARROW 1 MIRL	1.18	147656862
11 (-)	Amaranthus retroflexus CANADA AMES 5328-3 MIRL	1.17	147656862

12<br>(-)	Amaranthus palmeri MEXICO VERACRUZ PI667167-3 MIRL	1.17	147656862
13<br>(-)	Amaranthus spinosus PI 632248-1 MIRL	1.15	147656862
14<br>(-)	Amaranthus albus NC PI632244-2 MIRL	1.15	147656862
15<br>(-)	Amaranthus hypochondriacus MEXICO SONORA PI599682-3 MIRL	1.15	147656862
16<br>(-)	Amaranthus powelli HARROW 1 MIRL	1.14	147656862
17<br>(-)	Amaranthus blitoides CANADA PI608663-3 MIRL	1.12	147656862
18<br>(-)	Amaranthus powelli sub. powelli ME AMES 29205-2 MIRL	1.12	147656862
19<br>(-)	Amaranthus powelli sub. powelli ME AMES 29205-3 MIRL	1.11	147656862
20<br>(-)	Amaranthus retroflexus UTAH PI612857-3 MIRL	1.09	147656862

Analyte62

Analyte Name:	Amaranthus tricolor NJ AMES 5303-2 MIRL
Analyte Description:	MSP
Analyte ID:	366acdca-9280-4f36-a722-8ec250703d11
Analyte Creation Date/Time:	2022-11-24 3:05:21 PM
Applied MSP Library(ies):	
Applied Taxonomy Tree:	Bruker Taxonomy

Rank (Quality)	Matched Pattern	Score Value	NCBI Identifier
1 (+++)	Amaranthus tricolor NJ AMES 5303-2 MIRL	3	147656862
2 (+++)	Amaranthus tricolor NJ AMES 5303-3 MIRL	2.74	147656862
3 (+++)	Amaranthus tricolor NJ AMES 5303-1 MIRL	2.73	147656862
4 (-)	Amaranthus blitoides CANADA PI608663-1 MIRL	1.48	147656862
5 (-)	Amaranthus blitoides HARROW 2 MIRL	1.37	147656862
6 (-)	Amaranthus spinosus PI 632248-1 MIRL	1.33	147656862
7 (-)	Amaranthus blitoides CANADA PI608663-2 MIRL	1.2	147656862
8 (-)	Amaranthus blitoides CANADA PI608663-3 MIRL	1.18	147656862
9 (-)	Amaranthus spinosus PI 632248-3 MIRL	1.14	147656862
10 (-)	Amaranthus blitoides HARROW 3 MIRL	1.14	147656862
11 (-)	Amaranthus hypochondriacus MEXICO SONORA PI599682-2 MIRL	1.13	147656862

12<br>(-)	Amaranthus hypochondriacus MEXICO SONORA PI599682-3 MIRL	1.11	147656862
13<br>(-)	Amaranthus albus WASHINGTON PI654389-1 MIRL	1.04	147656862
14<br>(-)	Amaranthus albus NC PI632244-1 MIRL	1.03	147656862
15<br>(-)	Amaranthus albus NC PI632244-2 MIRL	1.03	147656862
16<br>(-)	Amaranthus hypochondriacus MEXICO SONORA PI599682-1 MIRL	1	147656862
17<br>(-)	Amaranthus retroflexus HARROW 3 MIRL	0.98	147656862
18<br>(-)	Amaranthus retroflexus ONTARIO AMES 35199-3 MIRL	0.96	147656862
19<br>(-)	Amaranthus retroflexus CANADA AMES 5328-2 MIRL	0.95	147656862
20<br>(-)	Amaranthus retroflexus CANADA AMES 5328-3 MIRL	0.94	147656862

Analyte63

Analyte Name:	Amaranthus tricolor NJ AMES 5303-3 MIRL
Analyte Description:	MSP
Analyte ID:	c3298f74-f899-4b5a-a321-7e114eb4006d
Analyte Creation Date/Time:	2022-11-24 3:10:06 PM
Applied MSP Library(ies):	
Applied Taxonomy Tree:	Bruker Taxonomy

Rank (Quality)	Matched Pattern	Score Value	NCBI Identifier
1 (+++)	Amaranthus tricolor NJ AMES 5303-3 MIRL	3	147656862
2 (+++)	Amaranthus tricolor NJ AMES 5303-1 MIRL	2.78	147656862
3 (+++)	Amaranthus tricolor NJ AMES 5303-2 MIRL	2.74	147656862
4 (-)	Amaranthus blitoides HARROW 2 MIRL	1.51	147656862
5 (-)	Amaranthus blitoides CANADA PI608663-1 MIRL	1.47	147656862
6 (-)	Amaranthus blitoides CANADA PI608663-2 MIRL	1.34	147656862
7 (-)	Amaranthus blitoides CANADA PI608663-3 MIRL	1.28	147656862
8 (-)	Amaranthus blitoides HARROW 3 MIRL	1.23	147656862
9 (-)	Amaranthus spinosus PI 632248-1 MIRL	1.16	147656862
10 (-)	Amaranthus retroflexus HARROW 1 MIRL	1.07	147656862
11 (-)	Amaranthus palmeri MEXICO VERACRUZ PI667167-3 MIRL	1.06	147656862

12<br>(-)	Amaranthus hypochondriacus MEXICO SONORA PI599682-3 MIRL	1.05	147656862
13<br>(-)	Amaranthus spinosus HARROW 3 MIRL	1.03	147656862
14<br>(-)	Amaranthus albus PI633580-1 MIRL	1.02	147656862
15<br>(-)	Amaranthus hypochondriacus MISSOURI PI698341-3 MIRL	1	147656862
16<br>(-)	Amaranthus retroflexus HARROW 3 MIRL	0.99	147656862
17<br>(-)	Amaranthus powellii sub. Powellii TEXAS PI632241-2 MIRL	0.98	147656862
18<br>(-)	Amaranthus tuberculatus IOWA PI 553086-1 MIRL	0.96	147656862
19<br>(-)	Amaranthus hypochondriacus MEXICO SONORA PI599682-2 MIRL	0.95	147656862
20<br>(-)	Amaranthus caudatus NJ AMES 5687-1 MIRL	0.94	147656862

Analyte64

Analyte Name:	Amaranthus hybridus HARROW 1 MIRL
Analyte Description:	MSP
Analyte ID:	fa9c5b11-92d7-4c5c-958b-4e03260f5c14
Analyte Creation Date/Time:	2022-11-24 3:15:21 PM
Applied MSP Library(ies):	
Applied Taxonomy Tree:	Bruker Taxonomy

Rank (Quality)	Matched Pattern	Score Value	NCBI Identifier
1 (+++)	Amaranthus hybridus HARROW 1 MIRL	3	147656862
2 (+++)	Amaranthus hybridus HARROW 3 MIRL	2.59	147656862
3 (+++)	Amaranthus hybridus HARROW 2 MIRL	2.52	147656862
4 (++)	Amaranthus caudatus NJ AMES 5687-2 MIRL	2.27	147656862
5 (++)	Amaranthus hypochondriacus PI658730-2 MIRL	2.24	147656862
6 (++)	Amaranthus hypochondriacus MEXICO SONORA PI599682-1 MIRL	2.18	147656862
7 (++)	Amaranthus hypochondriacus MEXICO SONORA PI599682-2 MIRL	2.16	147656862
8 (++)	Amaranthus hybridus INDIANA PI603895-2 MIRL	2.16	147656862
9 (++)	Amaranthus hypochondriacus MISSOURI PI698341-1 MIRL	2.14	147656862
10 (++)	Amaranthus caudatus NJ AMES 5687-3 MIRL	2.14	147656862
11 (++)	Amaranthus hypochondriacus PI658730-1 MIRL	2.12	147656862

12<br>(++)	Amaranthus hypochondriacus MISSOURI PI698341-2 MIRL	2.1	147656862
13<br>(++)	Amaranthus hybridus PUERTO RICO AMES 5152-3 MIRL	2.08	147656862
14<br>(++)	Amaranthus hypochondriacus MEXICO SONORA PI599682-3 MIRL	2.07	147656862
15<br>(++)	Amaranthus hypochondriacus MISSOURI PI698341-3 MIRL	2.06	147656862
16<br>(++)	Amaranthus hypochondriacus PI658730-3 MIRL	2.03	147656862
17<br>(++)	Amaranthus caudatus NJ AMES 5687-1 MIRL	2	147656862
18<br>(++)	Amaranthus hybridus PUERTO RICO AMES 5152-1 MIRL	2	147656862
19<br>(+)	Amaranthus hybridus INDIANA PI603895-1 MIRL	1.99	147656862
20<br>(+)	Amaranthus hybridus INDIANA PI603895-3 MIRL	1.97	147656862

Analyte65

Analyte Name:	Amaranthus hybridus HARROW 2 MIRL
Analyte Description:	MSP
Analyte ID:	cb05b308-1859-4202-a8b4-c64dd81825f5
Analyte Creation Date/Time:	2022-11-24 3:20:42 PM
Applied MSP Library(ies):	
Applied Taxonomy Tree:	Bruker Taxonomy

Rank (Quality)	Matched Pattern	Score Value	NCBI Identifier
1 (+++)	Amaranthus hybridus HARROW 2 MIRL	3	147656862
2 (+++)	Amaranthus hybridus HARROW 3 MIRL	2.84	147656862
3 (+++)	Amaranthus hybridus HARROW 1 MIRL	2.52	147656862
4 (+++)	Amaranthus hypochondriacus PI658730-2 MIRL	2.37	147656862
5 (+++)	Amaranthus hypochondriacus PI658730-1 MIRL	2.36	147656862
6 (+++)	Amaranthus caudatus NJ AMES 5687-2 MIRL	2.35	147656862
7 (+++)	Amaranthus hybridus PUERTO RICO AMES 5152-3 MIRL	2.34	147656862
8 (+++)	Amaranthus hybridus PUERTO RICO AMES 5152-2 MIRL	2.31	147656862
9 (+++)	Amaranthus hypochondriacus MISSOURI PI698341-1 MIRL	2.31	147656862
10 (++)	Amaranthus caudatus NJ AMES 5687-1 MIRL	2.29	147656862
11 (++)	Amaranthus hybridus PUERTO RICO AMES 5152-1 MIRL	2.28	147656862

12<br>(++)	Amaranthus hypochondriacus MEXICO SONORA PI599682-1 MIRL	2.26	147656862
13<br>(++)	Amaranthus caudatus NJ AMES 5687-3 MIRL	2.26	147656862
14<br>(++)	Amaranthus hypochondriacus PI658730-3 MIRL	2.22	147656862
15<br>(++)	Amaranthus hypochondriacus MEXICO SONORA PI599682-2 MIRL	2.22	147656862
16<br>(++)	Amaranthus hypochondriacus MISSOURI PI698341-3 MIRL	2.19	147656862
17<br>(++)	Amaranthus hybridus INDIANA PI603895-2 MIRL	2.13	147656862
18<br>(++)	Amaranthus hypochondriacus MEXICO SONORA PI599682-3 MIRL	2.12	147656862
19<br>(++)	Amaranthus hypochondriacus MISSOURI PI698341-2 MIRL	2.07	147656862
20<br>(+)	Amaranthus hybridus INDIANA PI603895-3 MIRL	1.9	147656862

Analyte66

Analyte Name:	Amaranthus hybridus HARROW 3 MIRL
Analyte Description:	MSP
Analyte ID:	9764e7f7-aa06-4cc7-99d8-232f4c158056
Analyte Creation Date/Time:	2022-11-24 3:26:26 PM
Applied MSP Library(ies):	
Applied Taxonomy Tree:	Bruker Taxonomy

Rank (Quality)	Matched Pattern	Score Value	NCBI Identifier
1 (+++)	Amaranthus hybridus HARROW 3 MIRL	3	147656862
2 (+++)	Amaranthus hybridus HARROW 2 MIRL	2.84	147656862
3 (+++)	Amaranthus hybridus HARROW 1 MIRL	2.59	147656862
4 (+++)	Amaranthus hypochondriacus PI658730-2 MIRL	2.48	147656862
5 (+++)	Amaranthus hypochondriacus PI658730-1 MIRL	2.42	147656862
6 (+++)	Amaranthus caudatus NJ AMES 5687-2 MIRL	2.37	147656862
7 (+++)	Amaranthus hybridus PUERTO RICO AMES 5152-3 MIRL	2.37	147656862
8 (+++)	Amaranthus caudatus NJ AMES 5687-1 MIRL	2.35	147656862
9 (+++)	Amaranthus hypochondriacus MISSOURI PI698341-1 MIRL	2.33	147656862
10 (+++)	Amaranthus hypochondriacus PI658730-3 MIRL	2.31	147656862
11 (+++)	Amaranthus hypochondriacus MEXICO SONORA PI599682-1 MIRL	2.3	147656862

12<br>(++)	Amaranthus caudatus NJ AMES 5687-3 MIRL	2.28	147656862
13<br>(++)	Amaranthus hybridus PUERTO RICO AMES 5152-2 MIRL	2.26	147656862
14<br>(++)	Amaranthus hypochondriacus MEXICO SONORA PI599682-2 MIRL	2.24	147656862
15<br>(++)	Amaranthus hybridus PUERTO RICO AMES 5152-1 MIRL	2.22	147656862
16<br>(++)	Amaranthus hypochondriacus MISSOURI PI698341-3 MIRL	2.16	147656862
17<br>(++)	Amaranthus hybridus INDIANA PI603895-2 MIRL	2.16	147656862
18<br>(++)	Amaranthus hypochondriacus MISSOURI PI698341-2 MIRL	2.13	147656862
19<br>(++)	Amaranthus hypochondriacus MEXICO SONORA PI599682-3 MIRL	2.12	147656862
20<br>(+)	Amaranthus hybridus INDIANA PI603895-3 MIRL	1.92	147656862

Analyte67

Analyte Name:	Amaranthus powelli sub. powelli ME AMES 29205-1 MIRL
Analyte Description:	MSP
Analyte ID:	394c4785-49b6-42e5-8172-72728511759a
Analyte Creation Date/Time:	2022-11-24 3:32:07 PM
Applied MSP Library(ies):	
Applied Taxonomy Tree:	Bruker Taxonomy

Rank (Quality)	Matched Pattern	Score Value	NCBI Identifier
1 (+++)	Amaranthus powelli sub. powelli ME AMES 29205-1 MIRL	3	147656862
2 (+++)	Amaranthus powelli sub. powelli ME AMES 29205-2 MIRL	2.88	147656862
3 (+++)	Amaranthus powelli sub. powelli ME AMES 29205-3 MIRL	2.81	147656862
4 (+++)	Amaranthus powelli HARROW 3 MIRL	2.75	147656862
5 (+++)	Amaranthus powelli HARROW 2 MIRL	2.65	147656862
6 (+++)	Amaranthus powellii sub. Powellii TEXAS PI632241-2 MIRL	2.56	147656862
7 (+++)	Amaranthus powelli HARROW 1 MIRL	2.48	147656862
8 (+++)	Amaranthus powellii sub. Powellii NM PI649309-2 MIRL	2.48	147656862
9 (+++)	Amaranthus powellii sub. Powellii NM PI649309-3 MIRL	2.33	147656862
10 (+++)	Amaranthus powellii sub. Powellii NM PI649309-1 MIRL	2.33	147656862
11 (++)	Amaranthus powellii sub. Powellii TEXAS PI632241-1 MIRL	2.29	147656862

12<br>(++)	Amaranthus retroflexus CANADA AMES 5328-3 MIRL	2.28	147656862
13<br>(++)	Amaranthus retroflexus HARROW 1 MIRL	2.26	147656862
14<br>(++)	Amaranthus retroflexus HARROW 3 MIRL	2.24	147656862
15<br>(++)	Amaranthus retroflexus CANADA AMES 5328-2 MIRL	2.21	147656862
16<br>(++)	Amaranthus powellii sub. Powellii TEXAS PI632241-3 MIRL	2.19	147656862
17<br>(++)	Amaranthus retroflexus CANADA AMES 5328-1 MIRL	2.15	147656862
18<br>(++)	Amaranthus retroflexus HARROW 2 MIRL	2.01	147656862
19<br>(+)	Amaranthus retroflexus ONTARIO AMES 35199-3 MIRL	1.95	147656862
20<br>(+)	Amaranthus hybridus HARROW 2 MIRL	1.81	147656862

Analyte68

Analyte Name:	Amaranthus powelli sub. powelli ME AMES 29205-2 MIRL
Analyte Description:	MSP
Analyte ID:	a743c1c3-415a-4bc1-888f-6ff6c89cef2e
Analyte Creation Date/Time:	2022-11-24 3:39:54 PM
Applied MSP Library(ies):	
Applied Taxonomy Tree:	Bruker Taxonomy

Rank (Quality)	Matched Pattern	Score Value	NCBI Identifier
1 (+++)	Amaranthus powelli sub. powelli ME AMES 29205-2 MIRL	3	147656862
2 (+++)	Amaranthus powelli sub. powelli ME AMES 29205-1 MIRL	2.88	147656862
3 (+++)	Amaranthus powelli sub. powelli ME AMES 29205-3 MIRL	2.87	147656862
4 (+++)	Amaranthus powelli HARROW 3 MIRL	2.68	147656862
5 (+++)	Amaranthus powelli HARROW 2 MIRL	2.59	147656862
6 (+++)	Amaranthus powellii sub. Powellii TEXAS PI632241-2 MIRL	2.55	147656862
7 (+++)	Amaranthus powellii sub. Powellii NM PI649309-2 MIRL	2.5	147656862
8 (+++)	Amaranthus powelli HARROW 1 MIRL	2.46	147656862
9 (+++)	Amaranthus powellii sub. Powellii NM PI649309-3 MIRL	2.3	147656862
10 (++)	Amaranthus retroflexus CANADA AMES 5328-2 MIRL	2.26	147656862
11 (++)	Amaranthus retroflexus CANADA AMES 5328-3 MIRL	2.25	147656862

12<br>(++)	Amaranthus powellii sub. Powellii NM PI649309-1 MIRL	2.25	147656862
13<br>(++)	Amaranthus powellii sub. Powellii TEXAS PI632241-1 MIRL	2.23	147656862
14<br>(++)	Amaranthus powellii sub. Powellii TEXAS PI632241-3 MIRL	2.16	147656862
15<br>(++)	Amaranthus retroflexus CANADA AMES 5328-1 MIRL	2.14	147656862
16<br>(++)	Amaranthus retroflexus HARROW 3 MIRL	2.13	147656862
17<br>(++)	Amaranthus retroflexus HARROW 1 MIRL	2.09	147656862
18<br>(+)	Amaranthus retroflexus ONTARIO AMES 35199-3 MIRL	1.93	147656862
19<br>(+)	Amaranthus retroflexus HARROW 2 MIRL	1.91	147656862
20<br>(+)	Amaranthus hybridus HARROW 2 MIRL	1.88	147656862

Analyte69

Analyte Name:	Amaranthus powelli sub. powelli ME AMES 29205-3 MIRL
Analyte Description:	MSP
Analyte ID:	1f887e90-50ae-49b5-bf90-5d0c80f50652
Analyte Creation Date/Time:	2022-11-24 3:46:13 PM
Applied MSP Library(ies):	
Applied Taxonomy Tree:	Bruker Taxonomy

Rank (Quality)	Matched Pattern	Score Value	NCBI Identifier
1 (+++)	Amaranthus powelli sub. powelli ME AMES 29205-3 MIRL	3	147656862
2 (+++)	Amaranthus powelli sub. powelli ME AMES 29205-2 MIRL	2.87	147656862
3 (+++)	Amaranthus powelli sub. powelli ME AMES 29205-1 MIRL	2.81	147656862
4 (+++)	Amaranthus powelli HARROW 3 MIRL	2.65	147656862
5 (+++)	Amaranthus powelli HARROW 2 MIRL	2.53	147656862
6 (+++)	Amaranthus powellii sub. Powellii TEXAS PI632241-2 MIRL	2.51	147656862
7 (+++)	Amaranthus powellii sub. Powellii NM PI649309-2 MIRL	2.45	147656862
8 (+++)	Amaranthus powelli HARROW 1 MIRL	2.44	147656862
9 (++)	Amaranthus powellii sub. Powellii NM PI649309-1 MIRL	2.25	147656862
10 (++)	Amaranthus powellii sub. Powellii TEXAS PI632241-1 MIRL	2.22	147656862
11 (++)	Amaranthus retroflexus CANADA AMES 5328-2 MIRL	2.22	147656862

12<br>(++)	Amaranthus powellii sub. Powellii NM PI649309-3 MIRL	2.22	147656862
13<br>(++)	Amaranthus retroflexus CANADA AMES 5328-3 MIRL	2.22	147656862
14<br>(++)	Amaranthus powellii sub. Powellii TEXAS PI632241-3 MIRL	2.16	147656862
15<br>(++)	Amaranthus retroflexus HARROW 1 MIRL	2.15	147656862
16<br>(++)	Amaranthus retroflexus HARROW 3 MIRL	2.09	147656862
17<br>(++)	Amaranthus retroflexus CANADA AMES 5328-1 MIRL	2.08	147656862
18<br>(+)	Amaranthus retroflexus HARROW 2 MIRL	1.91	147656862
19<br>(+)	Amaranthus hybridus HARROW 2 MIRL	1.79	147656862
20<br>(+)	Amaranthus retroflexus ONTARIO AMES 35199-3 MIRL	1.75	147656862

Analyte70

Analyte Name:	Amaranthus retroflexus CANADA AMES 5328-1 MIRL
Analyte Description:	MSP
Analyte ID:	a783e8d1-ee86-4f8f-99b1-614325b38271
Analyte Creation Date/Time:	2022-12-06 3:03:22 PM
Applied MSP Library(ies):	
Applied Taxonomy Tree:	Bruker Taxonomy

Rank (Quality)	Matched Pattern	Score Value	NCBI Identifier
1 (+++)	Amaranthus retroflexus CANADA AMES 5328-1 MIRL	3	147656862
2 (+++)	Amaranthus retroflexus CANADA AMES 5328-3 MIRL	2.85	147656862
3 (+++)	Amaranthus retroflexus CANADA AMES 5328-2 MIRL	2.72	147656862
4 (+++)	Amaranthus retroflexus HARROW 1 MIRL	2.67	147656862
5 (+++)	Amaranthus retroflexus HARROW 3 MIRL	2.65	147656862
6 (+++)	Amaranthus retroflexus HARROW 2 MIRL	2.45	147656862
7 (+++)	Amaranthus powellii sub. Powellii TEXAS PI632241-1 MIRL	2.3	147656862
8 (++)	Amaranthus powellii sub. Powellii TEXAS PI632241-2 MIRL	2.21	147656862
9 (++)	Amaranthus powelli HARROW 3 MIRL	2.2	147656862
10 (++)	Amaranthus powelli sub. powelli ME AMES 29205-1 MIRL	2.16	147656862
11 (++)	Amaranthus powelli HARROW 2 MIRL	2.16	147656862

12<br>(++)	Amaranthus powelli sub. powelli ME AMES 29205-2 MIRL	2.14	147656862
13<br>(++)	Amaranthus powelli sub. powelli ME AMES 29205-3 MIRL	2.08	147656862
14<br>(++)	Amaranthus powellii sub. Powellii TEXAS PI632241-3 MIRL	2.08	147656862
15<br>(++)	Amaranthus retroflexus ONTARIO AMES 35199-3 MIRL	2.04	147656862
16<br>(++)	Amaranthus powellii sub. Powellii NM PI649309-2 MIRL	2.02	147656862
17<br>(++)	Amaranthus powelli HARROW 1 MIRL	2.01	147656862
18<br>(+)	Amaranthus retroflexus UTAH PI612857-3 MIRL	1.9	147656862
19<br>(+)	Amaranthus hybridus HARROW 2 MIRL	1.88	147656862
20<br>(+)	Amaranthus powellii sub. Powellii NM PI649309-3 MIRL	1.86	147656862

Analyte71

Analyte Name:	Amaranthus retroflexus CANADA AMES 5328-2 MIRL
Analyte Description:	MSP
Analyte ID:	f5503fcb-f5a6-438b-825f-8107d9d476d8
Analyte Creation Date/Time:	2022-12-06 3:11:44 PM
Applied MSP Library(ies):	
Applied Taxonomy Tree:	Bruker Taxonomy

Rank (Quality)	Matched Pattern	Score Value	NCBI Identifier
1 (+++)	Amaranthus retroflexus CANADA AMES 5328-2 MIRL	3	147656862
2 (+++)	Amaranthus retroflexus CANADA AMES 5328-3 MIRL	2.72	147656862
3 (+++)	Amaranthus retroflexus CANADA AMES 5328-1 MIRL	2.72	147656862
4 (+++)	Amaranthus retroflexus HARROW 1 MIRL	2.61	147656862
5 (+++)	Amaranthus retroflexus HARROW 3 MIRL	2.59	147656862
6 (+++)	Amaranthus retroflexus HARROW 2 MIRL	2.42	147656862
7 (++)	Amaranthus powelli sub. powelli ME AMES 29205-2 MIRL	2.25	147656862
8 (++)	Amaranthus powelli sub. powelli ME AMES 29205-3 MIRL	2.22	147656862
9 (++)	Amaranthus powelli sub. powelli ME AMES 29205-1 MIRL	2.2	147656862
10 (++)	Amaranthus powellii sub. Powellii TEXAS PI632241-1 MIRL	2.14	147656862
11 (++)	Amaranthus powelli HARROW 3 MIRL	2.13	147656862

12<br>(++)	Amaranthus powelli HARROW 2 MIRL	2.12	147656862
13<br>(++)	Amaranthus powelli HARROW 1 MIRL	2.04	147656862
14<br>(++)	Amaranthus powellii sub. Powellii NM PI649309-2 MIRL	2.03	147656862
15<br>(++)	Amaranthus powellii sub. Powellii TEXAS PI632241-2 MIRL	2.02	147656862
16<br>(++)	Amaranthus powellii sub. Powellii TEXAS PI632241-3 MIRL	2.01	147656862
17<br>(+)	Amaranthus retroflexus ONTARIO AMES 35199-3 MIRL	1.99	147656862
18<br>(+)	Amaranthus retroflexus UTAH PI612857-3 MIRL	1.95	147656862
19<br>(+)	Amaranthus powellii sub. Powellii NM PI649309-1 MIRL	1.83	147656862
20<br>(+)	Amaranthus palmeri MALI PI549158-1 MIRL	1.8	147656862

Analyte72

Analyte Name:	Amaranthus retroflexus CANADA AMES 5328-3 MIRL
Analyte Description:	MSP
Analyte ID:	2eb86ff1-f54d-4113-b1f0-5d737c05e29b
Analyte Creation Date/Time:	2022-12-06 3:19:36 PM
Applied MSP Library(ies):	
Applied Taxonomy Tree:	Bruker Taxonomy

Rank (Quality)	Matched Pattern	Score Value	NCBI Identifier
1 (+++)	Amaranthus retroflexus CANADA AMES 5328-3 MIRL	3	147656862
2 (+++)	Amaranthus retroflexus CANADA AMES 5328-1 MIRL	2.85	147656862
3 (+++)	Amaranthus retroflexus HARROW 1 MIRL	2.76	147656862
4 (+++)	Amaranthus retroflexus CANADA AMES 5328-2 MIRL	2.72	147656862
5 (+++)	Amaranthus retroflexus HARROW 3 MIRL	2.72	147656862
6 (+++)	Amaranthus retroflexus HARROW 2 MIRL	2.57	147656862
7 (+++)	Amaranthus powelli HARROW 2 MIRL	2.32	147656862
8 (++)	Amaranthus powelli sub. powelli ME AMES 29205-1 MIRL	2.28	147656862
9 (++)	Amaranthus powellii sub. Powellii TEXAS PI632241-1 MIRL	2.26	147656862
10 (++)	Amaranthus powelli sub. powelli ME AMES 29205-2 MIRL	2.25	147656862
11 (++)	Amaranthus powelli HARROW 3 MIRL	2.24	147656862

12<br>(++)	Amaranthus powellii sub. Powellii TEXAS PI632241-2 MIRL	2.22	147656862
13<br>(++)	Amaranthus powelli sub. powelli ME AMES 29205-3 MIRL	2.22	147656862
14<br>(++)	Amaranthus powellii sub. Powellii TEXAS PI632241-3 MIRL	2.2	147656862
15<br>(++)	Amaranthus retroflexus ONTARIO AMES 35199-3 MIRL	2.19	147656862
16<br>(++)	Amaranthus powellii sub. Powellii NM PI649309-2 MIRL	2.19	147656862
17<br>(++)	Amaranthus powelli HARROW 1 MIRL	2.17	147656862
18<br>(++)	Amaranthus retroflexus UTAH PI612857-3 MIRL	2.09	147656862
19<br>(++)	Amaranthus powellii sub. Powellii NM PI649309-1 MIRL	2.05	147656862
20<br>(+)	Amaranthus retroflexus ONTARIO AMES 35199-2 MIRL	1.97	147656862

Analyte73

Analyte Name:	Amaranthus retroflexus HARROW 1 MIRL
Analyte Description:	MSP
Analyte ID:	73ab03ee-f614-4c77-bf49-abbff5b2f88b
Analyte Creation Date/Time:	2022-12-06 3:28:19 PM
Applied MSP Library(ies):	
Applied Taxonomy Tree:	Bruker Taxonomy

Rank (Quality)	Matched Pattern	Score Value	NCBI Identifier
1 (+++)	Amaranthus retroflexus HARROW 1 MIRL	3	147656862
2 (+++)	Amaranthus retroflexus HARROW 3 MIRL	2.9	147656862
3 (+++)	Amaranthus retroflexus CANADA AMES 5328-3 MIRL	2.76	147656862
4 (+++)	Amaranthus retroflexus HARROW 2 MIRL	2.76	147656862
5 (+++)	Amaranthus retroflexus CANADA AMES 5328-1 MIRL	2.67	147656862
6 (+++)	Amaranthus retroflexus CANADA AMES 5328-2 MIRL	2.61	147656862
7 (+++)	Amaranthus powelli HARROW 2 MIRL	2.35	147656862
8 (+++)	Amaranthus retroflexus ONTARIO AMES 35199-3 MIRL	2.3	147656862
9 (++)	Amaranthus powelli sub. powelli ME AMES 29205-1 MIRL	2.26	147656862
10 (++)	Amaranthus powelli HARROW 3 MIRL	2.24	147656862
11 (++)	Amaranthus powelli HARROW 1 MIRL	2.2	147656862

12 (++)	Amaranthus retroflexus ONTARIO AMES 35199-2 MIRL	2.15	147656862
13 (++)	Amaranthus powelli sub. powelli ME AMES 29205-3 MIRL	2.15	147656862
14 (++)	Amaranthus powellii sub. Powellii TEXAS PI632241-2 MIRL	2.11	147656862
15 (++)	Amaranthus powellii sub. Powellii NM PI649309-1 MIRL	2.11	147656862
16 (++)	Amaranthus retroflexus UTAH PI612857-3 MIRL	2.11	147656862
17 (++)	Amaranthus powelli sub. powelli ME AMES 29205-2 MIRL	2.09	147656862
18 (++)	Amaranthus powellii sub. Powellii NM PI649309-2 MIRL	2.08	147656862
19 (++)	Amaranthus powellii sub. Powellii TEXAS PI632241-1 MIRL	2.08	147656862
20 (++)	Amaranthus powellii sub. Powellii TEXAS PI632241-3 MIRL	2.05	147656862

Analyte74

Analyte Name:	Amaranthus retroflexus HARROW 2 MIRL
Analyte Description:	MSP
Analyte ID:	ed810d0f-0788-48d9-a5c3-62130f0bf741
Analyte Creation Date/Time:	2022-12-06 3:33:11 PM
Applied MSP Library(ies):	
Applied Taxonomy Tree:	Bruker Taxonomy

Rank (Quality)	Matched Pattern	Score Value	NCBI Identifier
1 (+++)	Amaranthus retroflexus HARROW 2 MIRL	3	147656862
2 (+++)	Amaranthus retroflexus HARROW 1 MIRL	2.76	147656862
3 (+++)	Amaranthus retroflexus HARROW 3 MIRL	2.73	147656862
4 (+++)	Amaranthus retroflexus CANADA AMES 5328-3 MIRL	2.57	147656862
5 (+++)	Amaranthus retroflexus CANADA AMES 5328-1 MIRL	2.45	147656862
6 (+++)	Amaranthus retroflexus CANADA AMES 5328-2 MIRL	2.42	147656862
7 (+++)	Amaranthus retroflexus ONTARIO AMES 35199-3 MIRL	2.36	147656862
8 (++)	Amaranthus powelli HARROW 1 MIRL	2.26	147656862
9 (++)	Amaranthus powelli HARROW 2 MIRL	2.26	147656862
10 (++)	Amaranthus retroflexus ONTARIO AMES 35199-2 MIRL	2.24	147656862
11 (++)	Amaranthus retroflexus UTAH PI612857-3 MIRL	2.2	147656862

12<br>(++)	Amaranthus retroflexus ONTARIO AMES 35199-1 MIRL	2.13	147656862
13<br>(++)	Amaranthus powellii sub. Powellii TEXAS PI632241-3 MIRL	2.11	147656862
14<br>(++)	Amaranthus powelli HARROW 3 MIRL	2.09	147656862
15<br>(++)	Amaranthus powelli sub. powelli ME AMES 29205-1 MIRL	2.01	147656862
16<br>(+)	Amaranthus powellii sub. Powellii NM PI649309-1 MIRL	1.97	147656862
17<br>(+)	Amaranthus powellii sub. Powellii TEXAS PI632241-2 MIRL	1.96	147656862
18<br>(+)	Amaranthus powellii sub. Powellii TEXAS PI632241-1 MIRL	1.92	147656862
19<br>(+)	Amaranthus powelli sub. powelli ME AMES 29205-2 MIRL	1.91	147656862
20<br>(+)	Amaranthus powelli sub. powelli ME AMES 29205-3 MIRL	1.91	147656862

Analyte75

Analyte Name:	Amaranthus retroflexus HARROW 3 MIRL
Analyte Description:	MSP
Analyte ID:	b5e91afb-a2ca-4933-b2dc-20283c670056
Analyte Creation Date/Time:	2022-12-06 3:38:31 PM
Applied MSP Library(ies):	
Applied Taxonomy Tree:	Bruker Taxonomy

Rank (Quality)	Matched Pattern	Score Value	NCBI Identifier
1 (+++)	Amaranthus retroflexus HARROW 3 MIRL	3	147656862
2 (+++)	Amaranthus retroflexus HARROW 1 MIRL	2.9	147656862
3 (+++)	Amaranthus retroflexus HARROW 2 MIRL	2.73	147656862
4 (+++)	Amaranthus retroflexus CANADA AMES 5328-3 MIRL	2.72	147656862
5 (+++)	Amaranthus retroflexus CANADA AMES 5328-1 MIRL	2.66	147656862
6 (+++)	Amaranthus retroflexus CANADA AMES 5328-2 MIRL	2.59	147656862
7 (++)	Amaranthus powelli HARROW 3 MIRL	2.27	147656862
8 (++)	Amaranthus powelli HARROW 2 MIRL	2.26	147656862
9 (++)	Amaranthus retroflexus ONTARIO AMES 35199-3 MIRL	2.25	147656862
10 (++)	Amaranthus powelli sub. powelli ME AMES 29205-1 MIRL	2.24	147656862
11 (++)	Amaranthus powelli sub. powelli ME AMES 29205-2 MIRL	2.13	147656862

12<br>(++)	Amaranthus powellii sub. Powellii TEXAS PI632241-2 MIRL	2.11	147656862
13<br>(++)	Amaranthus retroflexus UTAH PI612857-3 MIRL	2.1	147656862
14<br>(++)	Amaranthus powelli HARROW 1 MIRL	2.1	147656862
15<br>(++)	Amaranthus powelli sub. powelli ME AMES 29205-3 MIRL	2.09	147656862
16<br>(++)	Amaranthus powellii sub. Powellii TEXAS PI632241-1 MIRL	2.04	147656862
17<br>(++)	Amaranthus retroflexus ONTARIO AMES 35199-2 MIRL	2.03	147656862
18<br>(++)	Amaranthus powellii sub. Powellii TEXAS PI632241-3 MIRL	2	147656862
19<br>(++)	Amaranthus powellii sub. Powellii NM PI649309-2 MIRL	2	147656862
20<br>(+)	Amaranthus powellii sub. Powellii NM PI649309-1 MIRL	1.98	147656862

Analyte76

Analyte Name:	Amaranthus powelli HARROW 1 MIRL
Analyte Description:	MSP
Analyte ID:	435d601b-9311-416a-9722-b726224122f8
Analyte Creation Date/Time:	2022-12-06 3:42:40 PM
Applied MSP Library(ies):	
Applied Taxonomy Tree:	Bruker Taxonomy

Rank (Quality)	Matched Pattern	Score Value	NCBI Identifier
1 (+++)	Amaranthus powelli HARROW 1 MIRL	3	147656862
2 (+++)	Amaranthus powelli HARROW 2 MIRL	2.75	147656862
3 (+++)	Amaranthus powelli HARROW 3 MIRL	2.64	147656862
4 (+++)	Amaranthus powelli sub. powelli ME AMES 29205-1 MIRL	2.48	147656862
5 (+++)	Amaranthus powelli sub. powelli ME AMES 29205-2 MIRL	2.45	147656862
6 (+++)	Amaranthus powelli sub. powelli ME AMES 29205-3 MIRL	2.44	147656862
7 (+++)	Amaranthus powellii sub. Powellii NM PI649309-1 MIRL	2.43	147656862
8 (+++)	Amaranthus powellii sub. Powellii TEXAS PI632241-2 MIRL	2.41	147656862
9 (+++)	Amaranthus powellii sub. Powellii NM PI649309-3 MIRL	2.37	147656862
10 (++)	Amaranthus powellii sub. Powellii TEXAS PI632241-3 MIRL	2.28	147656862
11 (++)	Amaranthus retroflexus HARROW 2 MIRL	2.26	147656862

12<br>(++)	Amaranthus retroflexus HARROW 1 MIRL	2.2	147656862
13<br>(++)	Amaranthus retroflexus CANADA AMES 5328-3 MIRL	2.17	147656862
14<br>(++)	Amaranthus powellii sub. Powellii NM PI649309-2 MIRL	2.12	147656862
15<br>(++)	Amaranthus retroflexus HARROW 3 MIRL	2.1	147656862
16<br>(++)	Amaranthus powellii sub. Powellii TEXAS PI632241-1 MIRL	2.09	147656862
17<br>(++)	Amaranthus retroflexus CANADA AMES 5328-2 MIRL	2.04	147656862
18<br>(++)	Amaranthus retroflexus CANADA AMES 5328-1 MIRL	2.01	147656862
19<br>(+)	Amaranthus retroflexus ONTARIO AMES 35199-3 MIRL	1.97	147656862
20<br>(+)	Amaranthus retroflexus UTAH PI612857-3 MIRL	1.92	147656862

Analyte77

Analyte Name: Amaranthus powelli HARROW 2 MIRL
Analyte Description: MSP
Analyte ID: ba2760df-69e6-4b7f-a27f-af861b7abcc2
Analyte Creation Date/Time: 2022-12-06 3:46:43 PM
Applied MSP Library(ies):
Applied Taxonomy Tree: Bruker Taxonomy

Rank (Quality)	Matched Pattern	Score Value	NCBI Identifier
1 (+++)	Amaranthus powelli HARROW 2 MIRL	3	147656862
2 (+++)	Amaranthus powelli HARROW 1 MIRL	2.76	147656862
3 (+++)	Amaranthus powelli HARROW 3 MIRL	2.75	147656862
4 (+++)	Amaranthus powelli sub. powelli ME AMES 29205-1 MIRL	2.65	147656862
5 (+++)	Amaranthus powelli sub. powelli ME AMES 29205-2 MIRL	2.59	147656862
6 (+++)	Amaranthus powellii sub. Powellii TEXAS PI632241-2 MIRL	2.57	147656862
7 (+++)	Amaranthus powelli sub. powelli ME AMES 29205-3 MIRL	2.52	147656862
8 (+++)	Amaranthus powellii sub. Powellii NM PI649309-1 MIRL	2.43	147656862
9 (+++)	Amaranthus powellii sub. Powellii NM PI649309-3 MIRL	2.39	147656862
10 (+++)	Amaranthus retroflexus HARROW 1 MIRL	2.35	147656862
11 (+++)	Amaranthus powellii sub. Powellii TEXAS PI632241-3 MIRL	2.35	147656862

12<br>(+++)	Amaranthus retroflexus CANADA AMES 5328-3 MIRL	2.32	147656862
13<br>(++)	Amaranthus powellii sub. Powellii NM PI649309-2 MIRL	2.27	147656862
14<br>(++)	Amaranthus retroflexus HARROW 2 MIRL	2.26	147656862
15<br>(++)	Amaranthus retroflexus HARROW 3 MIRL	2.26	147656862
16<br>(++)	Amaranthus powellii sub. Powellii TEXAS PI632241-1 MIRL	2.17	147656862
17<br>(++)	Amaranthus retroflexus CANADA AMES 5328-1 MIRL	2.16	147656862
18<br>(++)	Amaranthus retroflexus CANADA AMES 5328-2 MIRL	2.12	147656862
19<br>(+)	Amaranthus retroflexus ONTARIO AMES 35199-3 MIRL	1.94	147656862
20<br>(+)	Amaranthus retroflexus UTAH PI612857-3 MIRL	1.91	147656862

Analyte78

Analyte Name:	Amaranthus powelli HARROW 3 MIRL
Analyte Description:	MSP
Analyte ID:	6a69de55-8700-4f87-bc11-bb60733176f6
Analyte Creation Date/Time:	2022-12-06 3:51:45 PM
Applied MSP Library(ies):	
Applied Taxonomy Tree:	Bruker Taxonomy

Rank (Quality)	Matched Pattern	Score Value	NCBI Identifier
1 (+++)	Amaranthus powelli HARROW 3 MIRL	3	147656862
2 (+++)	Amaranthus powelli sub. powelli ME AMES 29205-1 MIRL	2.75	147656862
3 (+++)	Amaranthus powelli HARROW 2 MIRL	2.75	147656862
4 (+++)	Amaranthus powelli sub. powelli ME AMES 29205-2 MIRL	2.69	147656862
5 (+++)	Amaranthus powelli sub. powelli ME AMES 29205-3 MIRL	2.65	147656862
6 (+++)	Amaranthus powelli HARROW 1 MIRL	2.64	147656862
7 (+++)	Amaranthus powellii sub. Powellii TEXAS PI632241-2 MIRL	2.53	147656862
8 (+++)	Amaranthus powellii sub. Powellii TEXAS PI632241-3 MIRL	2.32	147656862
9 (++)	Amaranthus powellii sub. Powellii NM PI649309-1 MIRL	2.29	147656862
10 (++)	Amaranthus retroflexus CANADA AMES 5328-3 MIRL	2.28	147656862
11 (++)	Amaranthus powellii sub. Powellii NM PI649309-3 MIRL	2.28	147656862

12<br>(++)	Amaranthus powellii sub. Powellii NM PI649309-2 MIRL	2.27	147656862
13<br>(++)	Amaranthus retroflexus HARROW 3 MIRL	2.27	147656862
14<br>(++)	Amaranthus retroflexus HARROW 1 MIRL	2.24	147656862
15<br>(++)	Amaranthus retroflexus CANADA AMES 5328-1 MIRL	2.2	147656862
16<br>(++)	Amaranthus powellii sub. Powellii TEXAS PI632241-1 MIRL	2.18	147656862
17<br>(++)	Amaranthus retroflexus CANADA AMES 5328-2 MIRL	2.13	147656862
18<br>(++)	Amaranthus retroflexus HARROW 2 MIRL	2.09	147656862
19<br>(+)	Amaranthus retroflexus ONTARIO AMES 35199-3 MIRL	1.84	147656862
20<br>(+)	Amaranthus retroflexus UTAH PI612857-3 MIRL	1.83	147656862

Analyte79

Analyte Name:	Amaranthus rudis HARROW 1 MIRL
Analyte Description:	MSP
Analyte ID:	b6b441b4-3341-4ed3-bffd-47cd8c5152e1
Analyte Creation Date/Time:	2022-12-19 10:31:28 AM
Applied MSP Library(ies):	
Applied Taxonomy Tree:	Bruker Taxonomy

Rank (Quality)	Matched Pattern	Score Value	NCBI Identifier
1 (+++)	Amaranthus rudis HARROW 1 MIRL	3	147656862
2 (+++)	Amaranthus rudis HARROW 2 MIRL	2.45	147656862
3 (++)	Amaranthus tuberculatus IOWA PI674264-2 MIRL	2.01	147656862
4 (+)	Amaranthus tuberculatus IOWA PI604247-1 MIRL	1.97	147656862
5 (+)	Amaranthus tuberculatus IOWA PI604247-2 MIRL	1.96	147656862
6 (+)	Amaranthus tuberculatus KANSAS PI 60743-3 MIRL	1.93	147656862
7 (+)	Amaranthus arenicola TEXAS PI667168-1 MIRL	1.89	147656862
8 (+)	Amaranthus tuberculatus IOWA PI 553086-1 MIRL	1.85	147656862
9 (+)	Amaranthus arenicola TEXAS PI667168-3 MIRL	1.84	147656862
10 (+)	Amaranthus rudis HARROW 3 MIRL	1.81	147656862
11 (+)	Amaranthus tuberculatus IOWA PI604247-3 MIRL	1.79	147656862

12<br>(+)	Amaranthus tuberculatus IOWA PI674264-1 MIRL	1.78	147656862
13<br>(+)	Amaranthus tuberculatus IOWA PI674264-3 MIRL	1.76	147656862
14<br>(+)	Amaranthus arenicola TEXAS PI667168-2 MIRL	1.7	147656862
15<br>(-)	Amaranthus tuberculatus IOWA PI 553086-2 MIRL	1.62	147656862
16<br>(-)	Amaranthus tuberculatus IOWA PI 553086-3 MIRL	1.57	147656862
17<br>(-)	Amaranthus tuberculatus KANSAS PI 60743-1 MIRL	1.57	147656862
18<br>(-)	Amaranthus tuberculatus KANSAS PI 60743-2 MIRL	1.51	147656862
19<br>(-)	Amaranthus arenicola KANSAS PI599671-1 MIRL	1.21	147656862
20<br>(-)	Amaranthus hypochondriacus PI658730-1 MIRL	1.06	147656862

Analyte80

Analyte Name: Amaranthus rudis HARROW 2 MIRL
Analyte Description: MSP
Analyte ID: 957570ed-65be-4bb7-8563-4d7e7ac8c84b
Analyte Creation Date/Time: 2022-12-19 10:40:20 AM
Applied MSP Library(ies):
Applied Taxonomy Tree: Bruker Taxonomy

Rank (Quality)	Matched Pattern	Score Value	NCBI Identifier
1 (+++)	Amaranthus rudis HARROW 2 MIRL	3	147656862
2 (+++)	Amaranthus rudis HARROW 1 MIRL	2.45	147656862
3 (+++)	Amaranthus rudis HARROW 3 MIRL	2.3	147656862
4 (++)	Amaranthus arenicola TEXAS PI667168-1 MIRL	2.12	147656862
5 (++)	Amaranthus arenicola TEXAS PI667168-3 MIRL	2.02	147656862
6 (+)	Amaranthus tuberculatus IOWA PI674264-3 MIRL	1.93	147656862
7 (+)	Amaranthus tuberculatus IOWA PI674264-2 MIRL	1.93	147656862
8 (+)	Amaranthus tuberculatus KANSAS PI 60743-1 MIRL	1.87	147656862
9 (+)	Amaranthus tuberculatus KANSAS PI 60743-3 MIRL	1.87	147656862
10 (+)	Amaranthus tuberculatus KANSAS PI 60743-2 MIRL	1.81	147656862
11 (+)	Amaranthus arenicola TEXAS PI667168-2 MIRL	1.8	147656862

12<br>(+)	Amaranthus tuberculatus IOWA PI604247-3 MIRL	1.8	147656862
13<br>(+)	Amaranthus tuberculatus IOWA PI674264-1 MIRL	1.78	147656862
14<br>(+)	Amaranthus tuberculatus IOWA PI 553086-1 MIRL	1.77	147656862
15<br>(-)	Amaranthus tuberculatus IOWA PI604247-1 MIRL	1.64	147656862
16<br>(-)	Amaranthus tuberculatus IOWA PI604247-2 MIRL	1.64	147656862
17<br>(-)	Amaranthus tuberculatus IOWA PI 553086-3 MIRL	1.51	147656862
18<br>(-)	Amaranthus albus WASHINGTON PI654389-1 MIRL	1.39	147656862
19<br>(-)	Amaranthus tuberculatus IOWA PI 553086-2 MIRL	1.38	147656862
20<br>(-)	Amaranthus albus HARROW 3 MIRL	1.33	147656862

Analyte81

Analyte Name:	Amaranthus rudis HARROW 3 MIRL
Analyte Description:	MSP
Analyte ID:	d11b2f73-3c5b-40d0-a511-41673abaf843
Analyte Creation Date/Time:	2022-12-19 10:46:02 AM
Applied MSP Library(ies):	
Applied Taxonomy Tree:	Bruker Taxonomy

Rank (Quality)	Matched Pattern	Score Value	NCBI Identifier
1 (+++)	Amaranthus rudis HARROW 3 MIRL	3	147656862
2 (+++)	Amaranthus rudis HARROW 2 MIRL	2.3	147656862
3 (++)	Amaranthus arenicola TEXAS PI667168-1 MIRL	2.07	147656862
4 (+)	Amaranthus arenicola TEXAS PI667168-3 MIRL	1.97	147656862
5 (+)	Amaranthus arenicola TEXAS PI667168-2 MIRL	1.84	147656862
6 (+)	Amaranthus tuberculatus IOWA PI 553086-1 MIRL	1.81	147656862
7 (+)	Amaranthus tuberculatus KANSAS PI 60743-2 MIRL	1.81	147656862
8 (+)	Amaranthus rudis HARROW 1 MIRL	1.78	147656862
9 (+)	Amaranthus tuberculatus KANSAS PI 60743-1 MIRL	1.77	147656862
10 (+)	Amaranthus tuberculatus IOWA PI674264-2 MIRL	1.72	147656862
11 (+)	Amaranthus tuberculatus IOWA PI 553086-3 MIRL	1.7	147656862

12<br>**(-)**	Amaranthus tuberculatus IOWA PI674264-3 MIRL	1.69	147656862
13<br>**(-)**	Amaranthus tuberculatus KANSAS PI 60743-3 MIRL	1.66	147656862
14<br>**(-)**	Amaranthus tuberculatus IOWA PI 553086-2 MIRL	1.63	147656862
15<br>**(-)**	Amaranthus tuberculatus IOWA PI674264-1 MIRL	1.56	147656862
16<br>**(-)**	Amaranthus tuberculatus IOWA PI604247-3 MIRL	1.54	147656862
17<br>**(-)**	Amaranthus tuberculatus IOWA PI604247-1 MIRL	1.51	147656862
18<br>**(-)**	Amaranthus tuberculatus IOWA PI604247-2 MIRL	1.49	147656862
19<br>**(-)**	Amaranthus arenicola KANSAS PI599671-1 MIRL	1.24	147656862
20<br>**(-)**	Amaranthus arenicola KANSAS PI599671-3 MIRL	1.19	147656862

Analyte82

Analyte Name:	Amaranthus albus HARROW 1 MIRL
Analyte Description:	MSP
Analyte ID:	8326c0ee-e518-4e9b-9a78-dea3019ba8ac
Analyte Creation Date/Time:	2022-12-19 10:55:53 AM
Applied MSP Library(ies):	
Applied Taxonomy Tree:	Bruker Taxonomy

Rank (Quality)	Matched Pattern	Score Value	NCBI Identifier
1 (+++)	Amaranthus albus HARROW 1 MIRL	3	147656862
2 (+++)	Amaranthus albus HARROW 2 MIRL	2.66	147656862
3 (+++)	Amaranthus albus HARROW 3 MIRL	2.53	147656862
4 (+++)	Amaranthus albus WASHINGTON PI654389-3 MIRL	2.45	147656862
5 (+++)	Amaranthus albus NC PI632244-1 MIRL	2.42	147656862
6 (+++)	Amaranthus albus NC PI632244-3 MIRL	2.35	147656862
7 (+++)	Amaranthus albus WASHINGTON PI654389-1 MIRL	2.34	147656862
8 (++)	Amaranthus albus NC PI632244-2 MIRL	2.27	147656862
9 (++)	Amaranthus albus WASHINGTON PI654389-2 MIRL	2.18	147656862
10 (+)	Amaranthus albus PI633580-3 MIRL	1.87	147656862
11 (+)	Amaranthus albus PI633580-1 MIRL	1.87	147656862

12<br>**(-)**	Amaranthus albus PI633580-2 MIRL	1.63	147656862
13<br>**(-)**	Amaranthus blitoides HARROW 2 MIRL	1.59	147656862
14<br>**(-)**	Amaranthus californicus CALIFORNIA PI595319-1 MIRL	1.53	147656862
15<br>**(-)**	Amaranthus blitoides CANADA PI608663-3 MIRL	1.47	147656862
16<br>**(-)**	Amaranthus blitoides CANADA PI608663-2 MIRL	1.46	147656862
17<br>**(-)**	Amaranthus blitoides CANADA PI608663-1 MIRL	1.4	147656862
18<br>**(-)**	Amaranthus blitoides HARROW 3 MIRL	1.37	147656862
19<br>**(-)**	Amaranthus californicus CALIFORNIA PI595319-2 MIRL	1.34	147656862
20<br>**(-)**	Amaranthus caudatus NJ PI553073-1 MIRL	1.27	147656862

Analyte83

Analyte Name:	Amaranthus albus HARROW 2 MIRL
Analyte Description:	MSP
Analyte ID:	f1de7837-bc29-4982-8627-f5cc2a1eec77
Analyte Creation Date/Time:	2022-12-19 11:00:59 AM
Applied MSP Library(ies):	
Applied Taxonomy Tree:	Bruker Taxonomy

Rank (Quality)	Matched Pattern	Score Value	NCBI Identifier
1 (+++)	Amaranthus albus HARROW 2 MIRL	3	147656862
2 (+++)	Amaranthus albus HARROW 1 MIRL	2.66	147656862
3 (+++)	Amaranthus albus HARROW 3 MIRL	2.4	147656862
4 (+++)	Amaranthus albus WASHINGTON PI654389-1 MIRL	2.37	147656862
5 (+++)	Amaranthus albus WASHINGTON PI654389-3 MIRL	2.33	147656862
6 (++)	Amaranthus albus NC PI632244-3 MIRL	2.28	147656862
7 (++)	Amaranthus albus NC PI632244-1 MIRL	2.28	147656862
8 (++)	Amaranthus albus NC PI632244-2 MIRL	2.2	147656862
9 (++)	Amaranthus albus WASHINGTON PI654389-2 MIRL	2.12	147656862
10 (+)	Amaranthus albus PI633580-3 MIRL	1.88	147656862
11 (+)	Amaranthus blitoides HARROW 3 MIRL	1.8	147656862

12<br>(+)	Amaranthus albus PI633580-2 MIRL	1.73	147656862
13<br>(-)	Amaranthus albus PI633580-1 MIRL	1.63	147656862
14<br>(-)	Amaranthus blitoides HARROW 2 MIRL	1.6	147656862
15<br>(-)	Amaranthus blitoides CANADA PI608663-2 MIRL	1.49	147656862
16<br>(-)	Amaranthus blitoides CANADA PI608663-3 MIRL	1.47	147656862
17<br>(-)	Amaranthus blitoides CANADA PI608663-1 MIRL	1.45	147656862
18<br>(-)	Amaranthus californicus CALIFORNIA PI595319-1 MIRL	1.38	147656862
19<br>(-)	Amaranthus hybridus INDIANA PI603895-1 MIRL	1.22	147656862
20<br>(-)	Amaranthus palmeri MALI PI549158-3 MIRL	1.19	147656862

Analyte84

Analyte Name:	Amaranthus albus HARROW 3 MIRL
Analyte Description:	MSP
Analyte ID:	83a22922-463d-49b0-8c90-dbf9a916d86d
Analyte Creation Date/Time:	2022-12-19 11:13:00 AM
Applied MSP Library(ies):	
Applied Taxonomy Tree:	Bruker Taxonomy

Rank (Quality)	Matched Pattern	Score Value	NCBI Identifier
1 (+++)	Amaranthus albus HARROW 3 MIRL	3	147656862
2 (+++)	Amaranthus albus HARROW 1 MIRL	2.53	147656862
3 (+++)	Amaranthus albus WASHINGTON PI654389-3 MIRL	2.47	147656862
4 (+++)	Amaranthus albus WASHINGTON PI654389-2 MIRL	2.45	147656862
5 (+++)	Amaranthus albus HARROW 2 MIRL	2.4	147656862
6 (++)	Amaranthus albus WASHINGTON PI654389-1 MIRL	2.21	147656862
7 (++)	Amaranthus albus NC PI632244-1 MIRL	2.2	147656862
8 (++)	Amaranthus albus NC PI632244-2 MIRL	2.15	147656862
9 (++)	Amaranthus albus PI633580-2 MIRL	2.08	147656862
10 (+)	Amaranthus albus NC PI632244-3 MIRL	1.92	147656862
11 (+)	Amaranthus albus PI633580-3 MIRL	1.75	147656862

12<br>(-)	Amaranthus blitoides HARROW 3 MIRL	1.56	147656862
13<br>(-)	Amaranthus californicus CALIFORNIA PI595319-1 MIRL	1.54	147656862
14<br>(-)	Amaranthus blitoides CANADA PI608663-3 MIRL	1.51	147656862
15<br>(-)	Amaranthus blitoides HARROW 2 MIRL	1.48	147656862
16<br>(-)	Amaranthus albus PI633580-1 MIRL	1.48	147656862
17<br>(-)	Amaranthus hybridus INDIANA PI603895-1 MIRL	1.39	147656862
18<br>(-)	Amaranthus caudatus NJ PI553073-1 MIRL	1.36	147656862
19<br>(-)	Amaranthus hybridus INDIANA PI603895-2 MIRL	1.35	147656862
20<br>(-)	Amaranthus rudis HARROW 2 MIRL	1.33	147656862

Analyte85

Analyte Name:	Amaranthus blitoides HARROW 2 MIRL
Analyte Description:	MSP
Analyte ID:	323358d7-b0f7-4246-9b84-1765eb4538fd
Analyte Creation Date/Time:	2022-12-19 11:23:44 AM
Applied MSP Library(ies):	
Applied Taxonomy Tree:	Bruker Taxonomy

Rank (Quality)	Matched Pattern	Score Value	NCBI Identifier
1 (+++)	Amaranthus blitoides HARROW 2 MIRL	3	147656862
2 (+++)	Amaranthus blitoides HARROW 3 MIRL	2.71	147656862
3 (+++)	Amaranthus blitoides CANADA PI608663-2 MIRL	2.6	147656862
4 (+++)	Amaranthus blitoides CANADA PI608663-1 MIRL	2.48	147656862
5 (+++)	Amaranthus blitoides CANADA PI608663-3 MIRL	2.32	147656862
6 (-)	Amaranthus albus HARROW 2 MIRL	1.61	147656862
7 (-)	Amaranthus albus HARROW 1 MIRL	1.59	147656862
8 (-)	Amaranthus albus WASHINGTON PI654389-3 MIRL	1.58	147656862
9 (-)	Amaranthus albus WASHINGTON PI654389-1 MIRL	1.55	147656862
10 (-)	Amaranthus albus NC PI632244-1 MIRL	1.54	147656862
11 (-)	Amaranthus tricolor NJ AMES 5303-3 MIRL	1.5	147656862

12<br>**(-)**	Amaranthus albus HARROW 3 MIRL	1.48	147656862
13<br>**(-)**	Amaranthus albus NC PI632244-2 MIRL	1.46	147656862
14<br>**(-)**	Amaranthus palmeri MALI PI549158-3 MIRL	1.45	147656862
15<br>**(-)**	Amaranthus albus PI633580-1 MIRL	1.43	147656862
16<br>**(-)**	Amaranthus albus NC PI632244-3 MIRL	1.43	147656862
17<br>**(-)**	Amaranthus tricolor NJ AMES 5303-2 MIRL	1.4	147656862
18<br>**(-)**	Amaranthus hybridus INDIANA PI603895-2 MIRL	1.37	147656862
19<br>**(-)**	Amaranthus tricolor NJ AMES 5303-1 MIRL	1.37	147656862
20<br>**(-)**	Amaranthus albus WASHINGTON PI654389-2 MIRL	1.34	147656862

Analyte86

Analyte Name:	Amaranthus blitoides HARROW 3 MIRL
Analyte Description:	MSP
Analyte ID:	ac6aed70-41ae-4ba4-a536-df54443ec94c
Analyte Creation Date/Time:	2022-12-19 11:30:54 AM
Applied MSP Library(ies):	
Applied Taxonomy Tree:	Bruker Taxonomy

Rank (Quality)	Matched Pattern	Score Value	NCBI Identifier
1 (+++)	Amaranthus blitoides HARROW 3 MIRL	3	147656862
2 (+++)	Amaranthus blitoides HARROW 2 MIRL	2.71	147656862
3 (+++)	Amaranthus blitoides CANADA PI608663-2 MIRL	2.57	147656862
4 (+++)	Amaranthus blitoides CANADA PI608663-3 MIRL	2.44	147656862
5 (+++)	Amaranthus blitoides CANADA PI608663-1 MIRL	2.41	147656862
6 (+)	Amaranthus albus HARROW 2 MIRL	1.81	147656862
7 (-)	Amaranthus albus WASHINGTON PI654389-3 MIRL	1.68	147656862
8 (-)	Amaranthus albus HARROW 3 MIRL	1.58	147656862
9 (-)	Amaranthus albus PI633580-3 MIRL	1.57	147656862
10 (-)	Amaranthus albus NC PI632244-1 MIRL	1.54	147656862
11 (-)	Amaranthus albus NC PI632244-3 MIRL	1.54	147656862

12<br>**(-)**	Amaranthus albus WASHINGTON PI654389-1 MIRL	1.5	147656862
13<br>**(-)**	Amaranthus albus NC PI632244-2 MIRL	1.48	147656862
14<br>**(-)**	Amaranthus hybridus INDIANA PI603895-2 MIRL	1.41	147656862
15<br>**(-)**	Amaranthus albus HARROW 1 MIRL	1.37	147656862
16<br>**(-)**	Amaranthus albus WASHINGTON PI654389-2 MIRL	1.34	147656862
17<br>**(-)**	Amaranthus tricolor NJ AMES 5303-1 MIRL	1.33	147656862
18<br>**(-)**	Amaranthus californicus CALIFORNIA PI595319-2 MIRL	1.32	147656862
19<br>**(-)**	Amaranthus albus PI633580-1 MIRL	1.31	147656862
20<br>**(-)**	Amaranthus hybridus HARROW 1 MIRL	1.26	147656862

Analyte87

Analyte Name: Amaranthus spinosus HARROW 1 MIRL
Analyte Description: MSP
Analyte ID: 6d856760-bf79-4627-8545-1ab26ff8cbb3
Analyte Creation Date/Time: 2022-12-19 11:42:04 AM
Applied MSP Library(ies):
Applied Taxonomy Tree: Bruker Taxonomy

Rank (Quality)	Matched Pattern	Score Value	NCBI Identifier
1 (+++)	Amaranthus spinosus HARROW 1 MIRL	3	147656862
2 (+++)	Amaranthus spinosus HARROW 2 MIRL	2.67	147656862
3 (+++)	Amaranthus spinosus HARROW 3 MIRL	2.62	147656862
4 (++)	Amaranthus spinosus NC PI632248-RE1 MIRL	2.21	147656862
5 (++)	Amaranthus spinosus NC PI632248-RE3 MIRL	2.16	147656862
6 (++)	Amaranthus spinosus NC PI632248-RE2 MIRL	2.16	147656862
7 (++)	Amaranthus watsonii MEXICO COLIMA PI633593-RE1 MIRL	2	147656862
8 (+)	Amaranthus palmeri MEXICO VERACRUZ PI667167-RE1 MIRL	1.84	147656862
9 (+)	Amaranthus palmeri MEXICO VERACRUZ PI667167-RE3 MIRL	1.81	147656862
10 (+)	Amaranthus watsonii MEXICO COLIMA PI633593-RE3 MIRL	1.81	147656862
11 (+)	Amaranthus palmeri MEXICO VERACRUZ PI667167-3 MIRL	1.73	147656862

12<br>(+)	Amaranthus palmeri MEXICO PUEBLA PI604557-1 MIRL	1.73	147656862
13<br>(-)	Amaranthus palmeri ARIZONA PI686461-2 MIRL	1.67	147656862
14<br>(-)	Amaranthus palmeri MEXICO PUEBLA PI604557-3 MIRL	1.65	147656862
15<br>(-)	Amaranthus watsonii MEXICO COLIMA PI633593-RE2 MIRL	1.65	147656862
16<br>(-)	Amaranthus palmeri ARIZONA PI686461-3 MIRL	1.65	147656862
17<br>(-)	Amaranthus palmeri DAKAR PI633587-1 MIRL	1.6	147656862
18<br>(-)	Amaranthus palmeri MEXICO PUEBLA PI604557-2 MIRL	1.6	147656862
19<br>(-)	Amaranthus palmeri MEXICO VERACRUZ PI667167-1 MIRL	1.58	147656862
20<br>(-)	Amaranthus palmeri MALI PI549158-2 MIRL	1.49	147656862

Analyte88

Analyte Name:	Amaranthus spinosus HARROW 2 MIRL
Analyte Description:	MSP
Analyte ID:	f59e98cb-a7ff-495d-baf3-4c14fb2ab5e9
Analyte Creation Date/Time:	2022-12-19 11:47:14 AM
Applied MSP Library(ies):	
Applied Taxonomy Tree:	Bruker Taxonomy

Rank (Quality)	Matched Pattern	Score Value	NCBI Identifier
1 (+++)	Amaranthus spinosus HARROW 2 MIRL	3	147656862
2 (+++)	Amaranthus spinosus HARROW 1 MIRL	2.67	147656862
3 (+++)	Amaranthus spinosus HARROW 3 MIRL	2.54	147656862
4 (++)	Amaranthus spinosus NC PI632248-RE1 MIRL	2.22	147656862
5 (++)	Amaranthus spinosus NC PI632248-RE2 MIRL	2.11	147656862
6 (++)	Amaranthus spinosus NC PI632248-RE3 MIRL	2.09	147656862
7 (++)	Amaranthus watsonii MEXICO COLIMA PI633593-RE1 MIRL	2.04	147656862
8 (+)	Amaranthus palmeri MEXICO VERACRUZ PI667167-RE3 MIRL	1.93	147656862
9 (+)	Amaranthus palmeri MEXICO VERACRUZ PI667167-RE1 MIRL	1.85	147656862
10 (+)	Amaranthus watsonii MEXICO COLIMA PI633593-RE3 MIRL	1.81	147656862
11 (+)	Amaranthus palmeri MEXICO VERACRUZ PI667167-3 MIRL	1.78	147656862

12<br>(+)	Amaranthus palmeri MEXICO PUEBLA PI604557-3 MIRL	1.75	147656862
13<br>(+)	Amaranthus palmeri MEXICO VERACRUZ PI667167-1 MIRL	1.72	147656862
14<br>(+)	Amaranthus watsonii MEXICO COLIMA PI633593-RE2 MIRL	1.72	147656862
15<br>(-)	Amaranthus palmeri ARIZONA PI686461-2 MIRL	1.69	147656862
16<br>(-)	Amaranthus palmeri MEXICO PUEBLA PI604557-1 MIRL	1.67	147656862
17<br>(-)	Amaranthus spinosus PI 632248-3 MIRL	1.53	147656862
18<br>(-)	Amaranthus palmeri MEXICO VERACRUZ PI667167-RE2 MIRL	1.53	147656862
19<br>(-)	Amaranthus palmeri MALI PI549158-2 MIRL	1.52	147656862
20<br>(-)	Amaranthus powellii sub. Powellii TEXAS PI632241-3 MIRL	1.5	147656862

Analyte89

Analyte Name:	Amaranthus spinosus HARROW 3 MIRL
Analyte Description:	MSP
Analyte ID:	461b42a3-a95d-4589-a668-75a9cbe1d49d
Analyte Creation Date/Time:	2022-12-19 11:55:02 AM
Applied MSP Library(ies):	
Applied Taxonomy Tree:	Bruker Taxonomy

Rank (Quality)	Matched Pattern	Score Value	NCBI Identifier
1 (+++)	Amaranthus spinosus HARROW 3 MIRL	3	147656862
2 (+++)	Amaranthus spinosus HARROW 1 MIRL	2.62	147656862
3 (+++)	Amaranthus spinosus HARROW 2 MIRL	2.55	147656862
4 (+++)	Amaranthus spinosus NC PI632248-RE1 MIRL	2.52	147656862
5 (+++)	Amaranthus spinosus NC PI632248-RE3 MIRL	2.51	147656862
6 (+++)	Amaranthus spinosus NC PI632248-RE2 MIRL	2.46	147656862
7 (++)	Amaranthus watsonii MEXICO COLIMA PI633593-RE1 MIRL	2.23	147656862
8 (++)	Amaranthus palmeri MEXICO VERACRUZ PI667167-RE3 MIRL	2.19	147656862
9 (++)	Amaranthus palmeri MEXICO VERACRUZ PI667167-RE1 MIRL	2.1	147656862
10 (++)	Amaranthus watsonii MEXICO COLIMA PI633593-RE3 MIRL	2	147656862
11 (+)	Amaranthus palmeri ARIZONA PI686461-2 MIRL	1.96	147656862

12<br>(+)	Amaranthus palmeri MEXICO PUEBLA PI604557-1 MIRL	1.9	147656862
13<br>(+)	Amaranthus palmeri MALI PI549158-2 MIRL	1.88	147656862
14<br>(+)	Amaranthus watsonii MEXICO COLIMA PI633593-RE2 MIRL	1.87	147656862
15<br>(+)	Amaranthus palmeri MEXICO PUEBLA PI604557-3 MIRL	1.85	147656862
16<br>(+)	Amaranthus palmeri MEXICO PUEBLA PI604557-2 MIRL	1.81	147656862
17<br>(+)	Amaranthus palmeri MEXICO VERACRUZ PI667167-3 MIRL	1.8	147656862
18<br>(+)	Amaranthus palmeri MALI PI549158-3 MIRL	1.78	147656862
19<br>(+)	Amaranthus palmeri MALI PI549158-1 MIRL	1.78	147656862
20<br>(+)	Amaranthus palmeri ARIZONA PI686461-3 MIRL	1.77	147656862

Analyte90

Analyte Name:	Amaranthus spinosus NC PI632248-RE1 MIRL
Analyte Description:	MSP
Analyte ID:	6e42046f-8088-4721-95d5-796d2f437c33
Analyte Creation Date/Time:	2023-01-05 11:40:46 AM
Applied MSP Library(ies):	
Applied Taxonomy Tree:	Bruker Taxonomy

Rank (Quality)	Matched Pattern	Score Value	NCBI Identifier
1 (+++)	Amaranthus spinosus NC PI632248-RE1 MIRL	3	147656862
2 (+++)	Amaranthus spinosus NC PI632248-RE3 MIRL	2.74	147656862
3 (+++)	Amaranthus spinosus NC PI632248-RE2 MIRL	2.68	147656862
4 (+++)	Amaranthus spinosus HARROW 3 MIRL	2.51	147656862
5 (++)	Amaranthus spinosus HARROW 2 MIRL	2.22	147656862
6 (++)	Amaranthus spinosus HARROW 1 MIRL	2.2	147656862
7 (++)	Amaranthus palmeri MEXICO VERACRUZ PI667167-RE3 MIRL	2.2	147656862
8 (++)	Amaranthus palmeri MEXICO VERACRUZ PI667167-RE1 MIRL	2.12	147656862
9 (++)	Amaranthus watsonii MEXICO COLIMA PI633593-RE1 MIRL	2.07	147656862
10 (++)	Amaranthus watsonii MEXICO COLIMA PI633593-RE2 MIRL	2.01	147656862
11 (+)	Amaranthus palmeri ARIZONA PI686461-2 MIRL	1.91	147656862

12<br>(+)	Amaranthus watsonii MEXICO COLIMA PI633593-RE3 MIRL	1.86	147656862
13<br>(+)	Amaranthus palmeri MEXICO PUEBLA PI604557-3 MIRL	1.83	147656862
14<br>(+)	Amaranthus palmeri DAKAR PI633586-2 MIRL	1.75	147656862
15<br>(+)	Amaranthus retroflexus HARROW 2 MIRL	1.74	147656862
16<br>(+)	Amaranthus palmeri MEXICO PUEBLA PI604557-1 MIRL	1.72	147656862
17<br>(-)	Amaranthus retroflexus HARROW 3 MIRL	1.69	147656862
18<br>(-)	Amaranthus palmeri MEXICO VERACRUZ PI667167-RE2 MIRL	1.66	147656862
19<br>(-)	Amaranthus retroflexus CANADA AMES 5328-3 MIRL	1.66	147656862
20<br>(-)	Amaranthus palmeri MALI PI549158-3 MIRL	1.64	147656862

Analyte91

Analyte Name:	Amaranthus spinosus NC PI632248-RE2 MIRL
Analyte Description:	MSP
Analyte ID:	4f16493b-8249-40ed-8fee-548be836b5b5
Analyte Creation Date/Time:	2023-01-05 11:49:28 AM
Applied MSP Library(ies):	
Applied Taxonomy Tree:	Bruker Taxonomy

Rank (Quality)	Matched Pattern	Score Value	NCBI Identifier
1 (+++)	Amaranthus spinosus NC PI632248-RE2 MIRL	3	147656862
2 (+++)	Amaranthus spinosus NC PI632248-RE3 MIRL	2.72	147656862
3 (+++)	Amaranthus spinosus NC PI632248-RE1 MIRL	2.68	147656862
4 (+++)	Amaranthus spinosus HARROW 3 MIRL	2.46	147656862
5 (++)	Amaranthus palmeri MEXICO VERACRUZ PI667167-RE3 MIRL	2.17	147656862
6 (++)	Amaranthus spinosus HARROW 1 MIRL	2.17	147656862
7 (++)	Amaranthus palmeri MEXICO VERACRUZ PI667167-RE1 MIRL	2.14	147656862
8 (++)	Amaranthus spinosus HARROW 2 MIRL	2.14	147656862
9 (++)	Amaranthus watsonii MEXICO COLIMA PI633593-RE1 MIRL	2.03	147656862
10 (+)	Amaranthus watsonii MEXICO COLIMA PI633593-RE2 MIRL	1.99	147656862
11 (+)	Amaranthus palmeri MEXICO PUEBLA PI604557-3 MIRL	1.94	147656862

12 (+)	Amaranthus watsonii MEXICO COLIMA PI633593-RE3 MIRL	1.93	147656862
13 (+)	Amaranthus palmeri MALI PI549158-2 MIRL	1.9	147656862
14 (+)	Amaranthus palmeri ARIZONA PI686461-2 MIRL	1.82	147656862
15 (+)	Amaranthus palmeri MEXICO PUEBLA PI604557-1 MIRL	1.81	147656862
16 (+)	Amaranthus retroflexus CANADA AMES 5328-3 MIRL	1.79	147656862
17 (+)	Amaranthus powelli HARROW 1 MIRL	1.77	147656862
18 (+)	Amaranthus palmeri DAKAR PI633586-2 MIRL	1.76	147656862
19 (+)	Amaranthus palmeri ARIZONA PI686461-3 MIRL	1.75	147656862
20 (+)	Amaranthus palmeri MEXICO VERACRUZ PI667167-RE2 MIRL	1.74	147656862

Analyte92

Analyte Name:	Amaranthus spinosus NC PI632248-RE3 MIRL
Analyte Description:	MSP
Analyte ID:	ec48af4a-d80f-4e3f-b158-c08558c7920e
Analyte Creation Date/Time:	2023-01-05 11:54:39 AM
Applied MSP Library(ies):	
Applied Taxonomy Tree:	Bruker Taxonomy

Rank (Quality)	Matched Pattern	Score Value	NCBI Identifier
1 (+++)	Amaranthus spinosus NC PI632248-RE3 MIRL	3	147656862
2 (+++)	Amaranthus spinosus NC PI632248-RE1 MIRL	2.75	147656862
3 (+++)	Amaranthus spinosus NC PI632248-RE2 MIRL	2.73	147656862
4 (+++)	Amaranthus spinosus HARROW 3 MIRL	2.51	147656862
5 (++)	Amaranthus watsonii MEXICO COLIMA PI633593-RE1 MIRL	2.25	147656862
6 (++)	Amaranthus spinosus HARROW 1 MIRL	2.18	147656862
7 (++)	Amaranthus palmeri MEXICO VERACRUZ PI667167-RE3 MIRL	2.16	147656862
8 (++)	Amaranthus palmeri MEXICO VERACRUZ PI667167-RE1 MIRL	2.16	147656862
9 (++)	Amaranthus watsonii MEXICO COLIMA PI633593-RE2 MIRL	2.16	147656862
10 (++)	Amaranthus spinosus HARROW 2 MIRL	2.12	147656862
11 (++)	Amaranthus palmeri ARIZONA PI686461-2 MIRL	2.09	147656862

12<br>(++)	Amaranthus palmeri MEXICO PUEBLA PI604557-3 MIRL	2.06	147656862
13<br>(++)	Amaranthus palmeri MEXICO PUEBLA PI604557-1 MIRL	2.01	147656862
14<br>(+)	Amaranthus palmeri MALI PI549158-2 MIRL	1.98	147656862
15<br>(+)	Amaranthus palmeri DAKAR PI633586-2 MIRL	1.93	147656862
16<br>(+)	Amaranthus watsonii MEXICO COLIMA PI633593-RE3 MIRL	1.93	147656862
17<br>(+)	Amaranthus palmeri MALI PI549158-1 MIRL	1.87	147656862
18<br>(+)	Amaranthus palmeri USA(AZ) PI632236-2 MIRL	1.86	147656862
19<br>(+)	Amaranthus palmeri DAKAR PI633586-1 MIRL	1.86	147656862
20<br>(+)	Amaranthus palmeri MEXICO PUEBLA PI604557-2 MIRL	1.84	147656862

Analyte93

Analyte Name:	Amaranthus palmeri MEXICO VERACRUZ PI667167-RE1 MIRL
Analyte Description:	MSP
Analyte ID:	4cbd9d2f-b2b4-4995-b053-05f5ee7b3f7d
Analyte Creation Date/Time:	2023-01-05 1:26:00 PM
Applied MSP Library(ies):	
Applied Taxonomy Tree:	Bruker Taxonomy

Rank (Quality)	Matched Pattern	Score Value	NCBI Identifier
1 (+++)	Amaranthus palmeri MEXICO VERACRUZ PI667167-RE1 MIRL	3	147656862
2 (+++)	Amaranthus palmeri MEXICO VERACRUZ PI667167-RE3 MIRL	2.32	147656862
3 (++)	Amaranthus watsonii MEXICO COLIMA PI633593-RE1 MIRL	2.21	147656862
4 (++)	Amaranthus spinosus NC PI632248-RE3 MIRL	2.15	147656862
5 (++)	Amaranthus watsonii MEXICO COLIMA PI633593-RE3 MIRL	2.14	147656862
6 (++)	Amaranthus spinosus NC PI632248-RE2 MIRL	2.14	147656862
7 (++)	Amaranthus spinosus NC PI632248-RE1 MIRL	2.12	147656862
8 (++)	Amaranthus palmeri DAKAR PI633586-2 MIRL	2.12	147656862
9 (++)	Amaranthus spinosus HARROW 3 MIRL	2.11	147656862
10 (++)	Amaranthus palmeri MEXICO VERACRUZ PI667167-RE2 MIRL	2.07	147656862
	Amaranthus palmeri MEXICO PUEBLA PI604557-3 MIRL	2.07	147656862

11<br>(++)			
12<br>(++)	Amaranthus palmeri DAKAR PI633586-3 MIRL	2.06	147656862
13<br>(++)	Amaranthus watsonii MEXICO COLIMA PI633593-RE2 MIRL	2.06	147656862
14<br>(+)	Amaranthus palmeri USA(AZ) PI632236-2 MIRL	1.97	147656862
15<br>(+)	Amaranthus palmeri ARIZONA PI686461-2 MIRL	1.94	147656862
16<br>(+)	Amaranthus palmeri MALI PI549158-2 MIRL	1.93	147656862
17<br>(+)	Amaranthus palmeri DAKAR PI633586-1 MIRL	1.92	147656862
18<br>(+)	Amaranthus palmeri DAKAR PI633587-1 MIRL	1.91	147656862
19<br>(+)	Amaranthus palmeri USA(AZ) PI632236-3 MIRL	1.91	147656862
20<br>(+)	Amaranthus spinosus HARROW 2 MIRL	1.87	147656862

Analyte94

Analyte Name:	Amaranthus palmeri MEXICO VERACRUZ PI667167-RE2 MIRL
Analyte Description:	MSP
Analyte ID:	61a7f58f-952e-42ba-8f2e-e3e27704e885
Analyte Creation Date/Time:	2023-01-05 1:31:24 PM
Applied MSP Library(ies):	
Applied Taxonomy Tree:	Bruker Taxonomy

Rank (Quality)	Matched Pattern	Score Value	NCBI Identifier
1 (+++)	Amaranthus palmeri MEXICO VERACRUZ PI667167-RE2 MIRL	3	147656862
2 (+++)	Amaranthus palmeri MEXICO VERACRUZ PI667167-RE3 MIRL	2.36	147656862
3 (++)	Amaranthus palmeri MEXICO VERACRUZ PI667167-1 MIRL	2.06	147656862
4 (++)	Amaranthus palmeri MEXICO VERACRUZ PI667167-RE1 MIRL	2.05	147656862
5 (+)	Amaranthus palmeri USA(AZ) PI632236-3 MIRL	1.85	147656862
6 (+)	Amaranthus watsonii MEXICO COLIMA PI633593-RE1 MIRL	1.83	147656862
7 (+)	Amaranthus palmeri DAKAR PI633586-2 MIRL	1.83	147656862
8 (+)	Amaranthus palmeri DAKAR PI633586-3 MIRL	1.81	147656862
9 (+)	Amaranthus watsonii MEXICO COLIMA PI633593-RE3 MIRL	1.8	147656862
10 (+)	Amaranthus palmeri DAKAR PI633586-1 MIRL	1.78	147656862
	Amaranthus palmeri MEXICO VERACRUZ PI667167-3 MIRL	1.77	147656862

11<br>(+)			
12<br>(+)	Amaranthus spinosus NC PI632248-RE3 MIRL	1.75	147656862
13<br>(+)	Amaranthus spinosus NC PI632248-RE2 MIRL	1.74	147656862
14<br>(-)	Amaranthus palmeri ARIZONA PI686461-3 MIRL	1.69	147656862
15<br>(-)	Amaranthus palmeri DAKAR PI633587-2 MIRL	1.69	147656862
16<br>(-)	Amaranthus palmeri MALI PI549158-2 MIRL	1.68	147656862
17<br>(-)	Amaranthus palmeri MALI PI549158-3 MIRL	1.67	147656862
18<br>(-)	Amaranthus spinosus NC PI632248-RE1 MIRL	1.66	147656862
19<br>(-)	Amaranthus palmeri MEXICO PUEBLA PI604557-3 MIRL	1.65	147656862
20<br>(-)	Amaranthus palmeri MEXICO PUEBLA PI604557-1 MIRL	1.64	147656862

Analyte95

Analyte Name:	Amaranthus palmeri MEXICO VERACRUZ PI667167-RE3 MIRL
Analyte Description:	MSP
Analyte ID:	cb444ee8-223a-46b8-97c0-8d31374d4f3d
Analyte Creation Date/Time:	2023-01-05 1:42:04 PM
Applied MSP Library(ies):	
Applied Taxonomy Tree:	Bruker Taxonomy

Rank (Quality)	Matched Pattern	Score Value	NCBI Identifier
1 (+++)	Amaranthus palmeri MEXICO VERACRUZ PI667167-RE3 MIRL	3	147656862
2 (+++)	Amaranthus palmeri MEXICO VERACRUZ PI667167-RE2 MIRL	2.36	147656862
3 (+++)	Amaranthus palmeri MEXICO VERACRUZ PI667167-RE1 MIRL	2.31	147656862
4 (++)	Amaranthus spinosus NC PI632248-RE1 MIRL	2.21	147656862
5 (++)	Amaranthus spinosus HARROW 3 MIRL	2.19	147656862
6 (++)	Amaranthus watsonii MEXICO COLIMA PI633593-RE1 MIRL	2.18	147656862
7 (++)	Amaranthus spinosus NC PI632248-RE2 MIRL	2.17	147656862
8 (++)	Amaranthus spinosus NC PI632248-RE3 MIRL	2.16	147656862
9 (++)	Amaranthus watsonii MEXICO COLIMA PI633593-RE3 MIRL	2.08	147656862
10 (++)	Amaranthus palmeri MEXICO PUEBLA PI604557-3 MIRL	2.01	147656862
	Amaranthus palmeri ARIZONA PI686461-2 MIRL	1.96	147656862

11<br>(+)			
12<br>(+)	Amaranthus spinosus HARROW 2 MIRL	1.94	147656862
13<br>(+)	Amaranthus palmeri DAKAR PI633586-1 MIRL	1.94	147656862
14<br>(+)	Amaranthus watsonii MEXICO COLIMA PI633593-RE2 MIRL	1.93	147656862
15<br>(+)	Amaranthus palmeri MEXICO PUEBLA PI604557-1 MIRL	1.91	147656862
16<br>(+)	Amaranthus palmeri MALI PI549158-2 MIRL	1.87	147656862
17<br>(+)	Amaranthus palmeri MALI PI549158-3 MIRL	1.86	147656862
18<br>(+)	Amaranthus palmeri USA(AZ) PI632236-2 MIRL	1.86	147656862
19<br>(+)	Amaranthus palmeri MALI PI549158-1 MIRL	1.84	147656862
20<br>(+)	Amaranthus palmeri MEXICO VERACRUZ PI667167-3 MIRL	1.83	147656862

Analyte96

Analyte Name:	Amaranthus watsonii MEXICO COLIMA PI633593-RE1 MIRL
Analyte Description:	MSP
Analyte ID:	7ac40ea8-3150-494b-b529-bd7896383494
Analyte Creation Date/Time:	2023-01-05 1:48:50 PM
Applied MSP Library(ies):	
Applied Taxonomy Tree:	Bruker Taxonomy

Rank (Quality)	Matched Pattern	Score Value	NCBI Identifier
1 (+++)	Amaranthus watsonii MEXICO COLIMA PI633593-RE1 MIRL	3	147656862
2 (+++)	Amaranthus watsonii MEXICO COLIMA PI633593-RE2 MIRL	2.35	147656862
3 (++)	Amaranthus watsonii MEXICO COLIMA PI633593-RE3 MIRL	2.28	147656862
4 (++)	Amaranthus spinosus NC PI632248-RE3 MIRL	2.24	147656862
5 (++)	Amaranthus spinosus HARROW 3 MIRL	2.24	147656862
6 (++)	Amaranthus palmeri MEXICO VERACRUZ PI667167-RE1 MIRL	2.21	147656862
7 (++)	Amaranthus palmeri MEXICO VERACRUZ PI667167-RE3 MIRL	2.18	147656862
8 (++)	Amaranthus spinosus NC PI632248-RE1 MIRL	2.07	147656862
9 (++)	Amaranthus palmeri MEXICO PUEBLA PI604557-3 MIRL	2.06	147656862
10 (++)	Amaranthus spinosus HARROW 2 MIRL	2.06	147656862
11 (++)	Amaranthus spinosus NC PI632248-RE2 MIRL	2.03	147656862

12<br>(++)	Amaranthus spinosus HARROW 1 MIRL	2.01	147656862
13<br>(++)	Amaranthus palmeri DAKAR PI633586-2 MIRL	2	147656862
14<br>(+)	Amaranthus palmeri ARIZONA PI686461-2 MIRL	1.99	147656862
15<br>(+)	Amaranthus palmeri MEXICO PUEBLA PI604557-1 MIRL	1.98	147656862
16<br>(+)	Amaranthus palmeri MALI PI549158-2 MIRL	1.97	147656862
17<br>(+)	Amaranthus palmeri MEXICO PUEBLA PI604557-2 MIRL	1.96	147656862
18<br>(+)	Amaranthus palmeri DAKAR PI633586-3 MIRL	1.92	147656862
19<br>(+)	Amaranthus palmeri DAKAR PI633586-1 MIRL	1.91	147656862
20<br>(+)	Amaranthus palmeri USA(AZ) PI632236-2 MIRL	1.89	147656862

Analyte97

Analyte Name:	Amaranthus watsonii MEXICO COLIMA PI633593-RE2 MIRL
Analyte Description:	MSP
Analyte ID:	f7670fd4-5ae6-41b3-b45d-d2c93c5d3ffa
Analyte Creation Date/Time:	2023-01-05 1:57:15 PM
Applied MSP Library(ies):	
Applied Taxonomy Tree:	Bruker Taxonomy

Rank (Quality)	Matched Pattern	Score Value	NCBI Identifier
1 (+++)	Amaranthus watsonii MEXICO COLIMA PI633593-RE2 MIRL	3	147656862
2 (+++)	Amaranthus palmeri MEXICO PUEBLA PI604557-2 MIRL	2.44	147656862
3 (+++)	Amaranthus watsonii MEXICO COLIMA PI633593-RE1 MIRL	2.36	147656862
4 (+++)	Amaranthus palmeri MALI PI549158-2 MIRL	2.35	147656862
5 (+++)	Amaranthus palmeri MEXICO PUEBLA PI604557-3 MIRL	2.33	147656862
6 (++)	Amaranthus palmeri MALI PI549158-1 MIRL	2.29	147656862
7 (++)	Amaranthus palmeri DAKAR PI633586-2 MIRL	2.27	147656862
8 (++)	Amaranthus palmeri MEXICO PUEBLA PI604557-1 MIRL	2.25	147656862
9 (++)	Amaranthus palmeri ARIZONA PI686461-2 MIRL	2.25	147656862
10 (++)	Amaranthus palmeri DAKAR PI633587-1 MIRL	2.23	147656862
11 (++)	Amaranthus watsonii MEXICO COLIMA PI633593-RE3 MIRL	2.23	147656862

12<br>(++)	Amaranthus palmeri DAKAR PI633586-1 MIRL	2.22	147656862
13<br>(++)	Amaranthus palmeri ARIZONA PI686461-3 MIRL	2.17	147656862
14<br>(++)	Amaranthus palmeri DAKAR PI633586-3 MIRL	2.17	147656862
15<br>(++)	Amaranthus spinosus NC PI632248-RE3 MIRL	2.16	147656862
16<br>(++)	Amaranthus palmeri MALI PI549158-3 MIRL	2.13	147656862
17<br>(++)	Amaranthus palmeri USA(AZ) PI632236-2 MIRL	2.09	147656862
18<br>(++)	Amaranthus palmeri MEXICO VERACRUZ PI667167-RE1 MIRL	2.07	147656862
19<br>(++)	Amaranthus palmeri ARIZONA PI686461-1 MIRL	2.03	147656862
20<br>(++)	Amaranthus spinosus NC PI632248-RE1 MIRL	2.02	147656862

Analyte98

Analyte Name:	Amaranthus watsonii MEXICO COLIMA PI633593-RE3 MIRL
Analyte Description:	MSP
Analyte ID:	8cc03c80-165c-4a16-aa29-40063f2ebb2e
Analyte Creation Date/Time:	2023-01-05 2:03:01 PM
Applied MSP Library(ies):	
Applied Taxonomy Tree:	Bruker Taxonomy

Rank (Quality)	Matched Pattern	Score Value	NCBI Identifier
1 (+++)	Amaranthus watsonii MEXICO COLIMA PI633593-RE3 MIRL	3	147656862
2 (++)	Amaranthus watsonii MEXICO COLIMA PI633593-RE1 MIRL	2.28	147656862
3 (++)	Amaranthus watsonii MEXICO COLIMA PI633593-RE2 MIRL	2.23	147656862
4 (++)	Amaranthus palmeri MEXICO PUEBLA PI604557-3 MIRL	2.18	147656862
5 (++)	Amaranthus palmeri MEXICO VERACRUZ PI667167-RE1 MIRL	2.14	147656862
6 (++)	Amaranthus palmeri ARIZONA PI686461-2 MIRL	2.12	147656862
7 (++)	Amaranthus palmeri MEXICO PUEBLA PI604557-1 MIRL	2.11	147656862
8 (++)	Amaranthus palmeri MEXICO VERACRUZ PI667167-RE3 MIRL	2.08	147656862
9 (++)	Amaranthus palmeri ARIZONA PI686461-3 MIRL	2.05	147656862
10 (++)	Amaranthus spinosus HARROW 3 MIRL	2	147656862
11 (+)	Amaranthus palmeri DAKAR PI633587-3 MIRL	1.99	147656862

12<br>(+)	Amaranthus palmeri MALI PI549158-2 MIRL	1.98	147656862
13<br>(+)	Amaranthus palmeri DAKAR PI633586-2 MIRL	1.97	147656862
14<br>(+)	Amaranthus palmeri MEXICO PUEBLA PI604557-2 MIRL	1.95	147656862
15<br>(+)	Amaranthus spinosus NC PI632248-RE2 MIRL	1.93	147656862
16<br>(+)	Amaranthus spinosus NC PI632248-RE3 MIRL	1.92	147656862
17<br>(+)	Amaranthus palmeri USA(AZ) PI632236-2 MIRL	1.91	147656862
18<br>(+)	Amaranthus palmeri MALI PI549158-1 MIRL	1.91	147656862
19<br>(+)	Amaranthus spinosus NC PI632248-RE1 MIRL	1.87	147656862
20<br>(+)	Amaranthus palmeri DAKAR PI633586-3 MIRL	1.86	147656862

Analyte99

Analyte Name:	Amaranthus powellii sub. Powellii TEXAS PI632241-1 MIRL
Analyte Description:	MSP
Analyte ID:	ddf23ff4-0f2c-4f8a-a0be-498f685553d6
Analyte Creation Date/Time:	2023-03-31 2:09:15 PM
Applied MSP Library(ies):	
Applied Taxonomy Tree:	Bruker Taxonomy

Rank (Quality)	Matched Pattern	Score Value	NCBI Identifier
1 (+++)	Amaranthus powellii sub. Powellii TEXAS PI632241-1 MIRL	3	147656862
2 (+++)	Amaranthus powellii sub. Powellii TEXAS PI632241-3 MIRL	2.65	147656862
3 (+++)	Amaranthus powellii sub. Powellii TEXAS PI632241-2 MIRL	2.52	147656862
4 (+++)	Amaranthus retroflexus CANADA AMES 5328-1 MIRL	2.3	147656862
5 (++)	Amaranthus powelli sub. powelli ME AMES 29205-1 MIRL	2.29	147656862
6 (++)	Amaranthus retroflexus CANADA AMES 5328-3 MIRL	2.26	147656862
7 (++)	Amaranthus powelli sub. powelli ME AMES 29205-2 MIRL	2.23	147656862
8 (++)	Amaranthus powelli sub. powelli ME AMES 29205-3 MIRL	2.22	147656862
9 (++)	Amaranthus powelli HARROW 3 MIRL	2.18	147656862
10 (++)	Amaranthus powelli HARROW 2 MIRL	2.17	147656862
11 (++)	Amaranthus retroflexus CANADA AMES 5328-2 MIRL	2.15	147656862

12<br>(++)	Amaranthus powelli HARROW 1 MIRL	2.09	147656862
13<br>(++)	Amaranthus powellii sub. Powellii NM PI649309-2 MIRL	2.07	147656862
14<br>(++)	Amaranthus retroflexus HARROW 1 MIRL	2.07	147656862
15<br>(++)	Amaranthus powellii sub. Powellii NM PI649309-3 MIRL	2.06	147656862
16<br>(++)	Amaranthus retroflexus HARROW 3 MIRL	2.03	147656862
17<br>(++)	Amaranthus powellii sub. Powellii NM PI649309-1 MIRL	2	147656862
18<br>(+)	Amaranthus retroflexus HARROW 2 MIRL	1.92	147656862
19<br>(+)	Amaranthus caudatus NJ AMES 5687-3 MIRL	1.89	147656862
20<br>(+)	Amaranthus hybridus HARROW 1 MIRL	1.88	147656862

Analyte100

Analyte Name:	Amaranthus powellii sub. Powellii TEXAS PI632241-2 MIRL
Analyte Description:	MSP
Analyte ID:	7f62edd2-3689-48b0-bae9-a5b8414e54e2
Analyte Creation Date/Time:	2023-03-31 2:26:30 PM
Applied MSP Library(ies):	
Applied Taxonomy Tree:	Bruker Taxonomy

Rank (Quality)	Matched Pattern	Score Value	NCBI Identifier
1 (+++)	Amaranthus powellii sub. Powellii TEXAS PI632241-2 MIRL	3	147656862
2 (+++)	Amaranthus powellii sub. Powellii TEXAS PI632241-3 MIRL	2.57	147656862
3 (+++)	Amaranthus powelli HARROW 2 MIRL	2.57	147656862
4 (+++)	Amaranthus powelli sub. powelli ME AMES 29205-1 MIRL	2.56	147656862
5 (+++)	Amaranthus powelli sub. powelli ME AMES 29205-2 MIRL	2.55	147656862
6 (+++)	Amaranthus powelli HARROW 3 MIRL	2.53	147656862
7 (+++)	Amaranthus powellii sub. Powellii TEXAS PI632241-1 MIRL	2.52	147656862
8 (+++)	Amaranthus powelli sub. powelli ME AMES 29205-3 MIRL	2.51	147656862
9 (+++)	Amaranthus powelli HARROW 1 MIRL	2.41	147656862
10 (++)	Amaranthus powellii sub. Powellii NM PI649309-2 MIRL	2.25	147656862
11 (++)	Amaranthus retroflexus CANADA AMES 5328-3 MIRL	2.22	147656862

12<br>(++)	Amaranthus powellii sub. Powellii NM PI649309-3 MIRL	2.22	147656862
13<br>(++)	Amaranthus powellii sub. Powellii NM PI649309-1 MIRL	2.22	147656862
14<br>(++)	Amaranthus retroflexus CANADA AMES 5328-1 MIRL	2.21	147656862
15<br>(++)	Amaranthus retroflexus HARROW 1 MIRL	2.11	147656862
16<br>(++)	Amaranthus retroflexus HARROW 3 MIRL	2.11	147656862
17<br>(++)	Amaranthus retroflexus CANADA AMES 5328-2 MIRL	2.02	147656862
18<br>(+)	Amaranthus retroflexus HARROW 2 MIRL	1.97	147656862
19<br>(+)	Amaranthus hypochondriacus MISSOURI PI698341-2 MIRL	1.83	147656862
20<br>(+)	Amaranthus retroflexus ONTARIO AMES 35199-3 MIRL	1.83	147656862

Analyte101

Analyte Name:	Amaranthus powellii sub. Powellii TEXAS PI632241-3 MIRL
Analyte Description:	MSP
Analyte ID:	59bd757c-32d1-4fb6-a3f7-3d79768ccbe3
Analyte Creation Date/Time:	2023-03-31 2:56:38 PM
Applied MSP Library(ies):	
Applied Taxonomy Tree:	Bruker Taxonomy

Rank (Quality)	Matched Pattern	Score Value	NCBI Identifier
1 (+++)	Amaranthus powellii sub. Powellii TEXAS PI632241-3 MIRL	3	147656862
2 (+++)	Amaranthus powellii sub. Powellii TEXAS PI632241-1 MIRL	2.66	147656862
3 (+++)	Amaranthus powellii sub. Powellii TEXAS PI632241-2 MIRL	2.57	147656862
4 (+++)	Amaranthus powelli HARROW 2 MIRL	2.35	147656862
5 (+++)	Amaranthus powelli HARROW 3 MIRL	2.32	147656862
6 (++)	Amaranthus powelli HARROW 1 MIRL	2.28	147656862
7 (++)	Amaranthus powellii sub. Powellii NM PI649309-3 MIRL	2.26	147656862
8 (++)	Amaranthus retroflexus CANADA AMES 5328-3 MIRL	2.2	147656862
9 (++)	Amaranthus powellii sub. Powellii NM PI649309-1 MIRL	2.2	147656862
10 (++)	Amaranthus powelli sub. powelli ME AMES 29205-1 MIRL	2.19	147656862
11 (++)	Amaranthus powelli sub. powelli ME AMES 29205-2 MIRL	2.17	147656862

12<br>(++)	Amaranthus powelli sub. powelli ME AMES 29205-3 MIRL	2.16	147656862
13<br>(++)	Amaranthus retroflexus HARROW 2 MIRL	2.1	147656862
14<br>(++)	Amaranthus retroflexus CANADA AMES 5328-1 MIRL	2.08	147656862
15<br>(++)	Amaranthus retroflexus HARROW 1 MIRL	2.05	147656862
16<br>(++)	Amaranthus retroflexus CANADA AMES 5328-2 MIRL	2.02	147656862
17<br>(++)	Amaranthus retroflexus HARROW 3 MIRL	2	147656862
18<br>(+)	Amaranthus powellii sub. Powellii NM PI649309-2 MIRL	1.98	147656862
19<br>(+)	Amaranthus caudatus CALIFORNIA PI690570-3 MIRL	1.94	147656862
20<br>(+)	Amaranthus hypochondriacus MISSOURI PI698341-3 MIRL	1.93	147656862

Analyte102

Analyte Name:	Amaranthus powellii sub. Powellii NM PI649309-1 MIRL
Analyte Description:	MSP
Analyte ID:	0f234928-40a8-4dec-a3de-1c2b9725f0a5
Analyte Creation Date/Time:	2023-03-31 3:03:31 PM
Applied MSP Library(ies):	
Applied Taxonomy Tree:	Bruker Taxonomy

Rank (Quality)	Matched Pattern	Score Value	NCBI Identifier
1 (+++)	Amaranthus powellii sub. Powellii NM PI649309-1 MIRL	3	147656862
2 (+++)	Amaranthus powellii sub. Powellii NM PI649309-3 MIRL	2.78	147656862
3 (+++)	Amaranthus powellii sub. Powellii NM PI649309-2 MIRL	2.48	147656862
4 (+++)	Amaranthus powelli HARROW 1 MIRL	2.42	147656862
5 (+++)	Amaranthus powelli HARROW 2 MIRL	2.42	147656862
6 (+++)	Amaranthus powelli sub. powelli ME AMES 29205-1 MIRL	2.32	147656862
7 (++)	Amaranthus powelli HARROW 3 MIRL	2.28	147656862
8 (++)	Amaranthus powelli sub. powelli ME AMES 29205-2 MIRL	2.24	147656862
9 (++)	Amaranthus powelli sub. powelli ME AMES 29205-3 MIRL	2.23	147656862
10 (++)	Amaranthus powellii sub. Powellii TEXAS PI632241-2 MIRL	2.21	147656862
11 (++)	Amaranthus powellii sub. Powellii TEXAS PI632241-3 MIRL	2.2	147656862

12<br>(++)	Amaranthus retroflexus HARROW 1 MIRL	2.11	147656862
13<br>(++)	Amaranthus retroflexus CANADA AMES 5328-3 MIRL	2.05	147656862
14<br>(+)	Amaranthus powellii sub. Powellii TEXAS PI632241-1 MIRL	1.99	147656862
15<br>(+)	Amaranthus retroflexus HARROW 3 MIRL	1.98	147656862
16<br>(+)	Amaranthus retroflexus UTAH PI612857-3 MIRL	1.97	147656862
17<br>(+)	Amaranthus retroflexus HARROW 2 MIRL	1.97	147656862
18<br>(+)	Amaranthus retroflexus ONTARIO AMES 35199-3 MIRL	1.88	147656862
19<br>(+)	Amaranthus retroflexus CANADA AMES 5328-1 MIRL	1.83	147656862
20<br>(+)	Amaranthus retroflexus CANADA AMES 5328-2 MIRL	1.83	147656862

Analyte103

Analyte Name: Amaranthus powellii sub. Powellii NM PI649309-2 MIRL
Analyte Description: MSP
Analyte ID: 4f40817c-277e-45c6-9b8f-71911c5e527c
Analyte Creation Date/Time: 2023-03-31 3:12:51 PM
Applied MSP Library(ies):
Applied Taxonomy Tree: Bruker Taxonomy

Rank (Quality)	Matched Pattern	Score Value	NCBI Identifier
1 (+++)	Amaranthus powellii sub. Powellii NM PI649309-2 MIRL	3	147656862
2 (+++)	Amaranthus powellii sub. Powellii NM PI649309-3 MIRL	2.51	147656862
3 (+++)	Amaranthus powelli sub. powelli ME AMES 29205-2 MIRL	2.5	147656862
4 (+++)	Amaranthus powellii sub. Powellii NM PI649309-1 MIRL	2.48	147656862
5 (+++)	Amaranthus powelli sub. powelli ME AMES 29205-1 MIRL	2.48	147656862
6 (+++)	Amaranthus powelli sub. powelli ME AMES 29205-3 MIRL	2.45	147656862
7 (++)	Amaranthus powelli HARROW 3 MIRL	2.27	147656862
8 (++)	Amaranthus powelli HARROW 2 MIRL	2.27	147656862
9 (++)	Amaranthus powellii sub. Powellii TEXAS PI632241-2 MIRL	2.25	147656862
10 (++)	Amaranthus retroflexus CANADA AMES 5328-3 MIRL	2.19	147656862
11 (++)	Amaranthus powelli HARROW 1 MIRL	2.12	147656862

12 (++)	Amaranthus retroflexus HARROW 1 MIRL	2.09	147656862
13 (++)	Amaranthus powellii sub. Powellii TEXAS PI632241-1 MIRL	2.08	147656862
14 (++)	Amaranthus retroflexus CANADA AMES 5328-2 MIRL	2.03	147656862
15 (++)	Amaranthus retroflexus CANADA AMES 5328-1 MIRL	2.03	147656862
16 (++)	Amaranthus retroflexus HARROW 3 MIRL	2	147656862
17 (+)	Amaranthus powellii sub. Powellii TEXAS PI632241-3 MIRL	1.98	147656862
18 (+)	Amaranthus retroflexus ONTARIO AMES 35199-3 MIRL	1.92	147656862
19 (+)	Amaranthus retroflexus HARROW 2 MIRL	1.91	147656862
20 (+)	Amaranthus retroflexus ONTARIO AMES 35199-2 MIRL	1.82	147656862

Analyte104

Analyte Name:	Amaranthus powellii sub. Powellii NM PI649309-3 MIRL
Analyte Description:	MSP
Analyte ID:	89e1772e-57a5-4add-88a0-7fdefe9f9de8
Analyte Creation Date/Time:	2023-03-31 3:23:45 PM
Applied MSP Library(ies):	
Applied Taxonomy Tree:	Bruker Taxonomy

Rank (Quality)	Matched Pattern	Score Value	NCBI Identifier
1 (+++)	Amaranthus powellii sub. Powellii NM PI649309-3 MIRL	3	147656862
2 (+++)	Amaranthus powellii sub. Powellii NM PI649309-1 MIRL	2.78	147656862
3 (+++)	Amaranthus powellii sub. Powellii NM PI649309-2 MIRL	2.51	147656862
4 (+++)	Amaranthus powelli HARROW 2 MIRL	2.39	147656862
5 (+++)	Amaranthus powelli HARROW 1 MIRL	2.37	147656862
6 (+++)	Amaranthus powelli sub. powelli ME AMES 29205-1 MIRL	2.33	147656862
7 (+++)	Amaranthus powelli sub. powelli ME AMES 29205-2 MIRL	2.3	147656862
8 (++)	Amaranthus powelli HARROW 3 MIRL	2.28	147656862
9 (++)	Amaranthus powellii sub. Powellii TEXAS PI632241-3 MIRL	2.26	147656862
10 (++)	Amaranthus powellii sub. Powellii TEXAS PI632241-2 MIRL	2.22	147656862
11 (++)	Amaranthus powelli sub. powelli ME AMES 29205-3 MIRL	2.22	147656862

12<br>(++)	Amaranthus powellii sub. Powellii TEXAS PI632241-1 MIRL	2.06	147656862
13<br>(+)	Amaranthus retroflexus HARROW 1 MIRL	1.99	147656862
14<br>(+)	Amaranthus retroflexus ONTARIO AMES 35199-3 MIRL	1.92	147656862
15<br>(+)	Amaranthus retroflexus CANADA AMES 5328-3 MIRL	1.92	147656862
16<br>(+)	Amaranthus retroflexus HARROW 2 MIRL	1.91	147656862
17<br>(+)	Amaranthus retroflexus CANADA AMES 5328-1 MIRL	1.86	147656862
18<br>(+)	Amaranthus retroflexus HARROW 3 MIRL	1.83	147656862
19<br>(+)	Amaranthus retroflexus UTAH PI612857-3 MIRL	1.83	147656862
20<br>(+)	Amaranthus retroflexus ONTARIO AMES 35199-2 MIRL	1.78	147656862

Analyte105

Analyte Name:	Amaranthus hybridus PUERTO RICO AMES 5152-1 MIRL
Analyte Description:	MSP
Analyte ID:	1a6dc17b-55a6-41aa-9392-0396be606bff
Analyte Creation Date/Time:	2023-03-31 3:28:49 PM
Applied MSP Library(ies):	
Applied Taxonomy Tree:	Bruker Taxonomy

Rank (Quality)	Matched Pattern	Score Value	NCBI Identifier
1 (+++)	Amaranthus hybridus PUERTO RICO AMES 5152-1 MIRL	3	147656862
2 (+++)	Amaranthus hybridus PUERTO RICO AMES 5152-2 MIRL	2.73	147656862
3 (+++)	Amaranthus hybridus PUERTO RICO AMES 5152-3 MIRL	2.69	147656862
4 (+++)	Amaranthus hypochondriacus MEXICO SONORA PI599682-1 MIRL	2.61	147656862
5 (+++)	Amaranthus hypochondriacus MEXICO SONORA PI599682-3 MIRL	2.53	147656862
6 (+++)	Amaranthus hypochondriacus PI658730-1 MIRL	2.44	147656862
7 (+++)	Amaranthus hypochondriacus MEXICO SONORA PI599682-2 MIRL	2.42	147656862
8 (+++)	Amaranthus caudatus NJ AMES 5687-1 MIRL	2.4	147656862
9 (+++)	Amaranthus hypochondriacus MISSOURI PI698341-3 MIRL	2.37	147656862
10 (+++)	Amaranthus hypochondriacus MISSOURI PI698341-1 MIRL	2.34	147656862
11 (+++)	Amaranthus hypochondriacus MISSOURI PI698341-2 MIRL	2.32	147656862

12<br>(++)	Amaranthus hybridus HARROW 2 MIRL	2.28	147656862
13<br>(++)	Amaranthus hypochondriacus PI658730-2 MIRL	2.27	147656862
14<br>(++)	Amaranthus caudatus NJ AMES 5687-2 MIRL	2.24	147656862
15<br>(++)	Amaranthus hypochondriacus PI658730-3 MIRL	2.23	147656862
16<br>(++)	Amaranthus hybridus HARROW 3 MIRL	2.22	147656862
17<br>(++)	Amaranthus caudatus NJ AMES 5687-3 MIRL	2.13	147656862
18<br>(++)	Amaranthus hybridus INDIANA PI603895-2 MIRL	2.02	147656862
19<br>(++)	Amaranthus hybridus HARROW 1 MIRL	2	147656862
20<br>(+)	Amaranthus caudatus NJ PI553073-3 MIRL	1.88	147656862

Analyte106

Analyte Name:	Amaranthus hybridus PUERTO RICO AMES 5152-2 MIRL
Analyte Description:	MSP
Analyte ID:	1d70fa38-0a0a-41d0-b4ad-ebd1d00cba60
Analyte Creation Date/Time:	2023-03-31 3:34:52 PM
Applied MSP Library(ies):	
Applied Taxonomy Tree:	Bruker Taxonomy

Rank (Quality)	Matched Pattern	Score Value	NCBI Identifier
1 (+++)	Amaranthus hybridus PUERTO RICO AMES 5152-2 MIRL	3	147656862
2 (+++)	Amaranthus hybridus PUERTO RICO AMES 5152-1 MIRL	2.73	147656862
3 (+++)	Amaranthus hybridus PUERTO RICO AMES 5152-3 MIRL	2.64	147656862
4 (+++)	Amaranthus hypochondriacus MEXICO SONORA PI599682-1 MIRL	2.63	147656862
5 (+++)	Amaranthus hypochondriacus MEXICO SONORA PI599682-3 MIRL	2.46	147656862
6 (+++)	Amaranthus hypochondriacus MISSOURI PI698341-1 MIRL	2.43	147656862
7 (+++)	Amaranthus hypochondriacus MEXICO SONORA PI599682-2 MIRL	2.43	147656862
8 (+++)	Amaranthus hypochondriacus PI658730-1 MIRL	2.42	147656862
9 (+++)	Amaranthus hypochondriacus PI658730-2 MIRL	2.39	147656862
10 (+++)	Amaranthus caudatus NJ AMES 5687-1 MIRL	2.34	147656862
11 (+++)	Amaranthus hypochondriacus PI658730-3 MIRL	2.31	147656862

12<br>(+++)	Amaranthus hybridus HARROW 2 MIRL	2.31	147656862
13<br>(+++)	Amaranthus hypochondriacus MISSOURI PI698341-3 MIRL	2.3	147656862
14<br>(++)	Amaranthus caudatus NJ AMES 5687-2 MIRL	2.26	147656862
15<br>(++)	Amaranthus hybridus HARROW 3 MIRL	2.25	147656862
16<br>(++)	Amaranthus hypochondriacus MISSOURI PI698341-2 MIRL	2.25	147656862
17<br>(++)	Amaranthus caudatus NJ AMES 5687-3 MIRL	2.16	147656862
18<br>(+)	Amaranthus hybridus INDIANA PI603895-2 MIRL	1.98	147656862
19<br>(+)	Amaranthus hybridus HARROW 1 MIRL	1.9	147656862
20<br>(+)	Amaranthus caudatus NJ PI553073-3 MIRL	1.89	147656862

Analyte107

Analyte Name: Amaranthus hybridus PUERTO RICO AMES 5152-3 MIRL
Analyte Description: MSP
Analyte ID: 80b1d3b5-c47a-4f59-b520-e1d043e1373f
Analyte Creation Date/Time: 2023-03-31 3:38:46 PM
Applied MSP Library(ies):
Applied Taxonomy Tree: Bruker Taxonomy

Rank (Quality)	Matched Pattern	Score Value	NCBI Identifier
1 (+++)	Amaranthus hybridus PUERTO RICO AMES 5152-3 MIRL	3	147656862
2 (+++)	Amaranthus hybridus PUERTO RICO AMES 5152-1 MIRL	2.69	147656862
3 (+++)	Amaranthus hybridus PUERTO RICO AMES 5152-2 MIRL	2.64	147656862
4 (+++)	Amaranthus hypochondriacus MEXICO SONORA PI599682-1 MIRL	2.61	147656862
5 (+++)	Amaranthus caudatus NJ AMES 5687-1 MIRL	2.48	147656862
6 (+++)	Amaranthus hypochondriacus PI658730-1 MIRL	2.48	147656862
7 (+++)	Amaranthus hypochondriacus MEXICO SONORA PI599682-2 MIRL	2.46	147656862
8 (+++)	Amaranthus hypochondriacus PI658730-2 MIRL	2.46	147656862
9 (+++)	Amaranthus hypochondriacus MISSOURI PI698341-1 MIRL	2.39	147656862
10 (+++)	Amaranthus hybridus HARROW 3 MIRL	2.37	147656862
11 (+++)	Amaranthus hypochondriacus PI658730-3 MIRL	2.34	147656862

12<br>(+++)	Amaranthus hybridus HARROW 2 MIRL	2.34	147656862
13<br>(+++)	Amaranthus caudatus NJ AMES 5687-2 MIRL	2.33	147656862
14<br>(+++)	Amaranthus caudatus NJ AMES 5687-3 MIRL	2.32	147656862
15<br>(++)	Amaranthus hypochondriacus MEXICO SONORA PI599682-3 MIRL	2.28	147656862
16<br>(++)	Amaranthus hypochondriacus MISSOURI PI698341-3 MIRL	2.24	147656862
17<br>(++)	Amaranthus hypochondriacus MISSOURI PI698341-2 MIRL	2.21	147656862
18<br>(++)	Amaranthus hybridus HARROW 1 MIRL	2.08	147656862
19<br>(+)	Amaranthus hybridus INDIANA PI603895-2 MIRL	1.92	147656862
20<br>(+)	Amaranthus caudatus NJ PI553073-2 MIRL	1.81	147656862

Analyte108

Analyte Name:	Amaranthus hybridus INDIANA PI603895-1 MIRL
Analyte Description:	MSP
Analyte ID:	d6e138d9-d49f-4a36-bacf-a691e21d8d52
Analyte Creation Date/Time:	2023-04-12 3:18:15 PM
Applied MSP Library(ies):	
Applied Taxonomy Tree:	Bruker Taxonomy

Rank (Quality)	Matched Pattern	Score Value	NCBI Identifier
1 (+++)	Amaranthus hybridus INDIANA PI603895-1 MIRL	3	147656862
2 (+++)	Amaranthus hybridus INDIANA PI603895-3 MIRL	2.58	147656862
3 (+++)	Amaranthus hybridus INDIANA PI603895-2 MIRL	2.35	147656862
4 (++)	Amaranthus caudatus CALIFORNIA PI690570-1 MIRL	2.12	147656862
5 (++)	Amaranthus caudatus CALIFORNIA PI690570-2 MIRL	2.08	147656862
6 (++)	Amaranthus caudatus NJ PI553073-1 MIRL	2.06	147656862
7 (++)	Amaranthus caudatus NJ PI553073-3 MIRL	2.06	147656862
8 (++)	Amaranthus caudatus NJ PI553073-2 MIRL	2	147656862
9 (+)	Amaranthus hybridus HARROW 1 MIRL	1.99	147656862
10 (+)	Amaranthus caudatus CALIFORNIA PI690570-3 MIRL	1.99	147656862
11 (+)	Amaranthus hypochondriacus MEXICO SONORA PI599682-3 MIRL	1.82	147656862

12<br>(+)	Amaranthus hybridus HARROW 3 MIRL	1.81	147656862
13<br>(+)	Amaranthus hypochondriacus MEXICO SONORA PI599682-2 MIRL	1.81	147656862
14<br>(+)	Amaranthus hypochondriacus MISSOURI PI698341-2 MIRL	1.71	147656862
15<br>(+)	Amaranthus hybridus HARROW 2 MIRL	1.7	147656862
16<br>(-)	Amaranthus hypochondriacus MISSOURI PI698341-1 MIRL	1.69	147656862
17<br>(-)	Amaranthus hypochondriacus MISSOURI PI698341-3 MIRL	1.68	147656862
18<br>(-)	Amaranthus hypochondriacus MEXICO SONORA PI599682-1 MIRL	1.6	147656862
19<br>(-)	Amaranthus hypochondriacus PI658730-2 MIRL	1.57	147656862
20<br>(-)	Amaranthus caudatus NJ AMES 5687-3 MIRL	1.56	147656862

Analyte109

Analyte Name: Amaranthus hybridus INDIANA PI603895-2 MIRL
Analyte Description: MSP
Analyte ID: 0a06f2a5-9c79-4163-afd7-67b9a59ae05e
Analyte Creation Date/Time: 2023-04-12 3:30:21 PM
Applied MSP Library(ies):
Applied Taxonomy Tree: Bruker Taxonomy

Rank (Quality)	Matched Pattern	Score Value	NCBI Identifier
1 (+++)	Amaranthus hybridus INDIANA PI603895-2 MIRL	3	147656862
2 (+++)	Amaranthus hybridus INDIANA PI603895-3 MIRL	2.43	147656862
3 (+++)	Amaranthus hybridus INDIANA PI603895-1 MIRL	2.35	147656862
4 (+++)	Amaranthus caudatus NJ PI553073-3 MIRL	2.31	147656862
5 (+++)	Amaranthus caudatus NJ PI553073-2 MIRL	2.3	147656862
6 (++)	Amaranthus hypochondriacus MEXICO SONORA PI599682-3 MIRL	2.29	147656862
7 (++)	Amaranthus caudatus CALIFORNIA PI690570-3 MIRL	2.29	147656862
8 (++)	Amaranthus hypochondriacus MEXICO SONORA PI599682-2 MIRL	2.23	147656862
9 (++)	Amaranthus hypochondriacus MISSOURI PI698341-2 MIRL	2.23	147656862
10 (++)	Amaranthus caudatus CALIFORNIA PI690570-1 MIRL	2.2	147656862
11 (++)	Amaranthus caudatus CALIFORNIA PI690570-2 MIRL	2.2	147656862

12<br>(++)	Amaranthus hybridus HARROW 1 MIRL	2.16	147656862
13<br>(++)	Amaranthus hybridus HARROW 3 MIRL	2.16	147656862
14<br>(++)	Amaranthus hypochondriacus MISSOURI PI698341-3 MIRL	2.15	147656862
15<br>(++)	Amaranthus hypochondriacus MISSOURI PI698341-1 MIRL	2.13	147656862
16<br>(++)	Amaranthus hybridus HARROW 2 MIRL	2.13	147656862
17<br>(++)	Amaranthus hypochondriacus MEXICO SONORA PI599682-1 MIRL	2.11	147656862
18<br>(++)	Amaranthus hybridus PUERTO RICO AMES 5152-1 MIRL	2.02	147656862
19<br>(+)	Amaranthus hybridus PUERTO RICO AMES 5152-2 MIRL	1.98	147656862
20<br>(+)	Amaranthus hybridus PUERTO RICO AMES 5152-3 MIRL	1.92	147656862

Analyte110

Analyte Name:	Amaranthus hybridus INDIANA PI603895-3 MIRL
Analyte Description:	MSP
Analyte ID:	7637ccf5-3187-4be1-a321-91547be47289
Analyte Creation Date/Time:	2023-04-12 3:37:28 PM
Applied MSP Library(ies):	
Applied Taxonomy Tree:	Bruker Taxonomy

Rank (Quality)	Matched Pattern	Score Value	NCBI Identifier
1 (+++)	Amaranthus hybridus INDIANA PI603895-3 MIRL	3	147656862
2 (+++)	Amaranthus hybridus INDIANA PI603895-1 MIRL	2.58	147656862
3 (+++)	Amaranthus hybridus INDIANA PI603895-2 MIRL	2.43	147656862
4 (++)	Amaranthus caudatus NJ PI553073-3 MIRL	2.19	147656862
5 (++)	Amaranthus caudatus NJ PI553073-2 MIRL	2.17	147656862
6 (++)	Amaranthus caudatus CALIFORNIA PI690570-1 MIRL	2.15	147656862
7 (++)	Amaranthus caudatus CALIFORNIA PI690570-2 MIRL	2.13	147656862
8 (++)	Amaranthus caudatus CALIFORNIA PI690570-3 MIRL	2.06	147656862
9 (+)	Amaranthus hybridus HARROW 1 MIRL	1.97	147656862
10 (+)	Amaranthus hypochondriacus MEXICO SONORA PI599682-3 MIRL	1.93	147656862
11 (+)	Amaranthus hybridus HARROW 3 MIRL	1.92	147656862

12<br>(+)	Amaranthus hybridus HARROW 2 MIRL	1.89	147656862
13<br>(+)	Amaranthus hypochondriacus MISSOURI PI698341-2 MIRL	1.88	147656862
14<br>(+)	Amaranthus hypochondriacus MEXICO SONORA PI599682-2 MIRL	1.8	147656862
15<br>(+)	Amaranthus hypochondriacus MISSOURI PI698341-3 MIRL	1.79	147656862
16<br>(+)	Amaranthus hypochondriacus PI658730-2 MIRL	1.78	147656862
17<br>(+)	Amaranthus hypochondriacus MISSOURI PI698341-1 MIRL	1.75	147656862
18<br>(+)	Amaranthus hypochondriacus MEXICO SONORA PI599682-1 MIRL	1.74	147656862
19<br>(-)	Amaranthus hypochondriacus PI658730-1 MIRL	1.67	147656862
20<br>(-)	Amaranthus caudatus NJ PI553073-1 MIRL	1.64	147656862

Analyte111

Analyte Name:	Amaranthus retroflexus ONTARIO AMES 35199-1 MIRL
Analyte Description:	MSP
Analyte ID:	aeebe366-dc8d-4b6c-8fe4-f8a0a935baff
Analyte Creation Date/Time:	2023-04-12 3:45:11 PM
Applied MSP Library(ies):	
Applied Taxonomy Tree:	Bruker Taxonomy

Rank (Quality)	Matched Pattern	Score Value	NCBI Identifier
1 (+++)	Amaranthus retroflexus ONTARIO AMES 35199-1 MIRL	3	147656862
2 (+++)	Amaranthus retroflexus ONTARIO AMES 35199-2 MIRL	2.68	147656862
3 (+++)	Amaranthus retroflexus UTAH PI612857-2 MIRL	2.6	147656862
4 (+++)	Amaranthus retroflexus UTAH PI612857-1 MIRL	2.59	147656862
5 (++)	Amaranthus retroflexus ONTARIO AMES 35199-3 MIRL	2.28	147656862
6 (++)	Amaranthus retroflexus UTAH PI612857-3 MIRL	2.17	147656862
7 (++)	Amaranthus retroflexus HARROW 2 MIRL	2.13	147656862
8 (+)	Amaranthus retroflexus HARROW 3 MIRL	1.94	147656862
9 (+)	Amaranthus retroflexus HARROW 1 MIRL	1.94	147656862
10 (+)	Amaranthus retroflexus CANADA AMES 5328-3 MIRL	1.9	147656862
11 (+)	Amaranthus powellii sub. Powellii NM PI649309-1 MIRL	1.74	147656862

12<br>(-)	Amaranthus powellii sub. Powellii NM PI649309-2 MIRL	1.65	147656862
13<br>(-)	Amaranthus powelli HARROW 1 MIRL	1.65	147656862
14<br>(-)	Amaranthus retroflexus CANADA AMES 5328-1 MIRL	1.63	147656862
15<br>(-)	Amaranthus powelli sub. powelli ME AMES 29205-1 MIRL	1.62	147656862
16<br>(-)	Amaranthus powellii sub. Powellii NM PI649309-3 MIRL	1.61	147656862
17<br>(-)	Amaranthus powelli HARROW 2 MIRL	1.57	147656862
18<br>(-)	Amaranthus powelli sub. powelli ME AMES 29205-2 MIRL	1.55	147656862
19<br>(-)	Amaranthus powelli HARROW 3 MIRL	1.52	147656862
20<br>(-)	Amaranthus caudatus CALIFORNIA PI690570-3 MIRL	1.49	147656862

Analyte112

Analyte Name:	Amaranthus retroflexus ONTARIO AMES 35199-2 MIRL
Analyte Description:	MSP
Analyte ID:	376d2d9b-7c37-47b5-a013-b3720163382c
Analyte Creation Date/Time:	2023-04-12 3:51:29 PM
Applied MSP Library(ies):	
Applied Taxonomy Tree:	Bruker Taxonomy

Rank (Quality)	Matched Pattern	Score Value	NCBI Identifier
1 (+++)	Amaranthus retroflexus ONTARIO AMES 35199-2 MIRL	3	147656862
2 (+++)	Amaranthus retroflexus ONTARIO AMES 35199-1 MIRL	2.67	147656862
3 (+++)	Amaranthus retroflexus ONTARIO AMES 35199-3 MIRL	2.63	147656862
4 (+++)	Amaranthus retroflexus UTAH PI612857-1 MIRL	2.37	147656862
5 (+++)	Amaranthus retroflexus UTAH PI612857-3 MIRL	2.32	147656862
6 (+++)	Amaranthus retroflexus UTAH PI612857-2 MIRL	2.3	147656862
7 (++)	Amaranthus retroflexus HARROW 2 MIRL	2.24	147656862
8 (++)	Amaranthus retroflexus HARROW 1 MIRL	2.15	147656862
9 (++)	Amaranthus retroflexus HARROW 3 MIRL	2.03	147656862
10 (+)	Amaranthus retroflexus CANADA AMES 5328-3 MIRL	1.97	147656862
11 (+)	Amaranthus powellii sub. Powellii NM PI649309-2 MIRL	1.82	147656862

12<br>(+)	Amaranthus powellii sub. Powellii NM PI649309-1 MIRL	1.8	147656862
13<br>(+)	Amaranthus retroflexus CANADA AMES 5328-1 MIRL	1.78	147656862
14<br>(+)	Amaranthus powellii sub. Powellii NM PI649309-3 MIRL	1.78	147656862
15<br>(+)	Amaranthus powelli HARROW 2 MIRL	1.76	147656862
16<br>(+)	Amaranthus powelli HARROW 1 MIRL	1.76	147656862
17<br>(+)	Amaranthus powellii sub. Powellii TEXAS PI632241-2 MIRL	1.73	147656862
18<br>(+)	Amaranthus powelli sub. powelli ME AMES 29205-2 MIRL	1.72	147656862
19<br>(+)	Amaranthus powelli sub. powelli ME AMES 29205-1 MIRL	1.71	147656862
20<br>(-)	Amaranthus powelli HARROW 3 MIRL	1.67	147656862

Analyte113

Analyte Name:	Amaranthus retroflexus ONTARIO AMES 35199-3 MIRL
Analyte Description:	MSP
Analyte ID:	065ef2c3-632c-446d-88f8-fabbe81a9c97
Analyte Creation Date/Time:	2023-04-12 3:58:02 PM
Applied MSP Library(ies):	
Applied Taxonomy Tree:	Bruker Taxonomy

Rank (Quality)	Matched Pattern	Score Value	NCBI Identifier
1 (+++)	Amaranthus retroflexus ONTARIO AMES 35199-3 MIRL	3	147656862
2 (+++)	Amaranthus retroflexus ONTARIO AMES 35199-2 MIRL	2.64	147656862
3 (+++)	Amaranthus retroflexus UTAH PI612857-3 MIRL	2.44	147656862
4 (+++)	Amaranthus retroflexus HARROW 2 MIRL	2.36	147656862
5 (+++)	Amaranthus retroflexus HARROW 1 MIRL	2.3	147656862
6 (++)	Amaranthus retroflexus ONTARIO AMES 35199-1 MIRL	2.28	147656862
7 (++)	Amaranthus retroflexus HARROW 3 MIRL	2.24	147656862
8 (++)	Amaranthus retroflexus CANADA AMES 5328-3 MIRL	2.19	147656862
9 (++)	Amaranthus retroflexus UTAH PI612857-1 MIRL	2.14	147656862
10 (++)	Amaranthus retroflexus UTAH PI612857-2 MIRL	2.05	147656862
11 (++)	Amaranthus retroflexus CANADA AMES 5328-1 MIRL	2.03	147656862

12<br>(+)	Amaranthus retroflexus CANADA AMES 5328-2 MIRL	1.99	147656862
13<br>(+)	Amaranthus powelli HARROW 1 MIRL	1.97	147656862
14<br>(+)	Amaranthus powelli sub. powelli ME AMES 29205-1 MIRL	1.95	147656862
15<br>(+)	Amaranthus powelli HARROW 2 MIRL	1.94	147656862
16<br>(+)	Amaranthus powelli sub. powelli ME AMES 29205-2 MIRL	1.93	147656862
17<br>(+)	Amaranthus powellii sub. Powellii NM PI649309-3 MIRL	1.92	147656862
18<br>(+)	Amaranthus powellii sub. Powellii NM PI649309-2 MIRL	1.92	147656862
19<br>(+)	Amaranthus powellii sub. Powellii NM PI649309-1 MIRL	1.88	147656862
20<br>(+)	Amaranthus powelli HARROW 3 MIRL	1.83	147656862

Analyte114

Analyte Name:	Amaranthus retroflexus UTAH PI612857-1 MIRL
Analyte Description:	MSP
Analyte ID:	7c8ab489-77b7-4e67-b274-df3e60c6d2a7
Analyte Creation Date/Time:	2023-04-13 2:11:19 PM
Applied MSP Library(ies):	
Applied Taxonomy Tree:	Bruker Taxonomy

Rank (Quality)	Matched Pattern	Score Value	NCBI Identifier
1 (+++)	Amaranthus retroflexus UTAH PI612857-1 MIRL	3	147656862
2 (+++)	Amaranthus retroflexus UTAH PI612857-2 MIRL	2.88	147656862
3 (+++)	Amaranthus retroflexus ONTARIO AMES 35199-1 MIRL	2.59	147656862
4 (+++)	Amaranthus retroflexus ONTARIO AMES 35199-2 MIRL	2.37	147656862
5 (++)	Amaranthus retroflexus ONTARIO AMES 35199-3 MIRL	2.13	147656862
6 (++)	Amaranthus retroflexus UTAH PI612857-3 MIRL	2.03	147656862
7 (+)	Amaranthus retroflexus HARROW 1 MIRL	1.76	147656862
8 (+)	Amaranthus powelli HARROW 1 MIRL	1.72	147656862
9 (+)	Amaranthus powelli HARROW 2 MIRL	1.7	147656862
10 (-)	Amaranthus powellii sub. Powellii NM PI649309-1 MIRL	1.68	147656862
11 (-)	Amaranthus retroflexus HARROW 2 MIRL	1.65	147656862

12<br>(-)	Amaranthus powellii sub. Powellii NM PI649309-3 MIRL	1.6	147656862
13<br>(-)	Amaranthus retroflexus CANADA AMES 5328-3 MIRL	1.59	147656862
14<br>(-)	Amaranthus retroflexus HARROW 3 MIRL	1.58	147656862
15<br>(-)	Amaranthus powelli HARROW 3 MIRL	1.58	147656862
16<br>(-)	Amaranthus powellii sub. Powellii TEXAS PI632241-2 MIRL	1.57	147656862
17<br>(-)	Amaranthus powellii sub. Powellii NM PI649309-2 MIRL	1.55	147656862
18<br>(-)	Amaranthus powelli sub. powelli ME AMES 29205-3 MIRL	1.52	147656862
19<br>(-)	Amaranthus powelli sub. powelli ME AMES 29205-2 MIRL	1.5	147656862
20<br>(-)	Amaranthus powelli sub. powelli ME AMES 29205-1 MIRL	1.49	147656862

Analyte115

Analyte Name:	Amaranthus retroflexus UTAH PI612857-2 MIRL
Analyte Description:	MSP
Analyte ID:	5452e1bf-31a0-48dd-a87d-359d18f59942
Analyte Creation Date/Time:	2023-04-13 2:19:20 PM
Applied MSP Library(ies):	
Applied Taxonomy Tree:	Bruker Taxonomy

Rank (Quality)	Matched Pattern	Score Value	NCBI Identifier
1 (+++)	Amaranthus retroflexus UTAH PI612857-2 MIRL	3	147656862
2 (+++)	Amaranthus retroflexus UTAH PI612857-1 MIRL	2.88	147656862
3 (+++)	Amaranthus retroflexus ONTARIO AMES 35199-1 MIRL	2.6	147656862
4 (+++)	Amaranthus retroflexus ONTARIO AMES 35199-2 MIRL	2.3	147656862
5 (++)	Amaranthus retroflexus ONTARIO AMES 35199-3 MIRL	2.05	147656862
6 (++)	Amaranthus retroflexus UTAH PI612857-3 MIRL	2	147656862
7 (+)	Amaranthus retroflexus HARROW 2 MIRL	1.78	147656862
8 (+)	Amaranthus retroflexus HARROW 1 MIRL	1.76	147656862
9 (-)	Amaranthus powelli HARROW 1 MIRL	1.66	147656862
10 (-)	Amaranthus retroflexus HARROW 3 MIRL	1.62	147656862
11 (-)	Amaranthus powelli HARROW 2 MIRL	1.59	147656862

12<br>(-)	Amaranthus retroflexus CANADA AMES 5328-3 MIRL	1.56	147656862
13<br>(-)	Amaranthus powelli HARROW 3 MIRL	1.51	147656862
14<br>(-)	Amaranthus hybridus INDIANA PI603895-3 MIRL	1.45	147656862
15<br>(-)	Amaranthus powellii sub. Powellii NM PI649309-1 MIRL	1.42	147656862
16<br>(-)	Amaranthus caudatus CALIFORNIA PI690570-2 MIRL	1.4	147656862
17<br>(-)	Amaranthus retroflexus CANADA AMES 5328-1 MIRL	1.39	147656862
18<br>(-)	Amaranthus powelli sub. powelli ME AMES 29205-3 MIRL	1.38	147656862
19<br>(-)	Amaranthus powellii sub. Powellii TEXAS PI632241-3 MIRL	1.37	147656862
20<br>(-)	Amaranthus powellii sub. Powellii NM PI649309-2 MIRL	1.37	147656862

Analyte116

Analyte Name:	Amaranthus retroflexus UTAH PI612857-3 MIRL
Analyte Description:	MSP
Analyte ID:	0a5d361e-6c14-4d73-a361-29384641a2f0
Analyte Creation Date/Time:	2023-04-13 2:24:51 PM
Applied MSP Library(ies):	
Applied Taxonomy Tree:	Bruker Taxonomy

Rank (Quality)	Matched Pattern	Score Value	NCBI Identifier
1 (+++)	Amaranthus retroflexus UTAH PI612857-3 MIRL	3	147656862
2 (+++)	Amaranthus retroflexus ONTARIO AMES 35199-3 MIRL	2.44	147656862
3 (+++)	Amaranthus retroflexus ONTARIO AMES 35199-2 MIRL	2.31	147656862
4 (++)	Amaranthus retroflexus HARROW 2 MIRL	2.19	147656862
5 (++)	Amaranthus retroflexus ONTARIO AMES 35199-1 MIRL	2.15	147656862
6 (++)	Amaranthus retroflexus HARROW 1 MIRL	2.1	147656862
7 (++)	Amaranthus retroflexus HARROW 3 MIRL	2.1	147656862
8 (++)	Amaranthus retroflexus CANADA AMES 5328-3 MIRL	2.09	147656862
9 (++)	Amaranthus retroflexus UTAH PI612857-1 MIRL	2.02	147656862
10 (+)	Amaranthus retroflexus UTAH PI612857-2 MIRL	1.99	147656862
11 (+)	Amaranthus powellii sub. Powellii NM PI649309-1 MIRL	1.97	147656862

12<br>(+)	Amaranthus powellii sub. Powellii TEXAS PI632241-3 MIRL	1.97	147656862
13<br>(+)	Amaranthus retroflexus CANADA AMES 5328-2 MIRL	1.95	147656862
14<br>(+)	Amaranthus powelli HARROW 2 MIRL	1.92	147656862
15<br>(+)	Amaranthus powelli HARROW 1 MIRL	1.92	147656862
16<br>(+)	Amaranthus retroflexus CANADA AMES 5328-1 MIRL	1.89	147656862
17<br>(+)	Amaranthus powelli HARROW 3 MIRL	1.83	147656862
18<br>(+)	Amaranthus powellii sub. Powellii TEXAS PI632241-1 MIRL	1.83	147656862
19<br>(+)	Amaranthus powellii sub. Powellii NM PI649309-3 MIRL	1.81	147656862
20<br>(+)	Amaranthus powelli sub. powelli ME AMES 29205-2 MIRL	1.77	147656862

Analyte117

Analyte Name:	Amaranthus caudatus NJ PI553073-1 MIRL
Analyte Description:	MSP
Analyte ID:	a90cdf33-a31f-43a7-ac13-70f83e16b258
Analyte Creation Date/Time:	2023-04-25 1:25:15 PM
Applied MSP Library(ies):	
Applied Taxonomy Tree:	Bruker Taxonomy

Rank (Quality)	Matched Pattern	Score Value	NCBI Identifier
1 (+++)	Amaranthus caudatus NJ PI553073-1 MIRL	3	147656862
2 (++)	Amaranthus hybridus INDIANA PI603895-1 MIRL	2.06	147656862
3 (+)	Amaranthus caudatus CALIFORNIA PI690570-1 MIRL	1.98	147656862
4 (+)	Amaranthus caudatus CALIFORNIA PI690570-2 MIRL	1.94	147656862
5 (+)	Amaranthus caudatus CALIFORNIA PI690570-3 MIRL	1.94	147656862
6 (+)	Amaranthus caudatus NJ PI553073-2 MIRL	1.92	147656862
7 (+)	Amaranthus caudatus NJ PI553073-3 MIRL	1.83	147656862
8 (-)	Amaranthus hybridus INDIANA PI603895-3 MIRL	1.63	147656862
9 (-)	Amaranthus hybridus INDIANA PI603895-2 MIRL	1.63	147656862
10 (-)	Amaranthus hypochondriacus MEXICO SONORA PI599682-2 MIRL	1.54	147656862
11 (-)	Amaranthus albus WASHINGTON PI654389-2 MIRL	1.46	147656862

12<br>(-)	Amaranthus hypochondriacus MISSOURI PI698341-2 MIRL	1.45	147656862
13<br>(-)	Amaranthus powelli HARROW 2 MIRL	1.4	147656862
14<br>(-)	Amaranthus albus HARROW 3 MIRL	1.37	147656862
15<br>(-)	Amaranthus hypochondriacus MISSOURI PI698341-3 MIRL	1.37	147656862
16<br>(-)	Amaranthus albus WASHINGTON PI654389-1 MIRL	1.31	147656862
17<br>(-)	Amaranthus hypochondriacus MEXICO SONORA PI599682-3 MIRL	1.29	147656862
18<br>(-)	Amaranthus albus HARROW 1 MIRL	1.27	147656862
19<br>(-)	Amaranthus hypochondriacus MEXICO SONORA PI599682-1 MIRL	1.25	147656862
20<br>(-)	Amaranthus hybridus HARROW 1 MIRL	1.24	147656862

Analyte118

Analyte Name: Amaranthus caudatus NJ PI553073-2 MIRL
Analyte Description: MSP
Analyte ID: 06fbcc91-aa09-4a1f-9c39-510fa58a6613
Analyte Creation Date/Time: 2023-04-25 1:31:42 PM
Applied MSP Library(ies):
Applied Taxonomy Tree: Bruker Taxonomy

Rank (Quality)	Matched Pattern	Score Value	NCBI Identifier
1 (+++)	Amaranthus caudatus NJ PI553073-2 MIRL	3	147656862
2 (+++)	Amaranthus caudatus NJ PI553073-3 MIRL	2.72	147656862
3 (+++)	Amaranthus caudatus CALIFORNIA PI690570-3 MIRL	2.65	147656862
4 (+++)	Amaranthus caudatus CALIFORNIA PI690570-1 MIRL	2.58	147656862
5 (+++)	Amaranthus caudatus CALIFORNIA PI690570-2 MIRL	2.53	147656862
6 (++)	Amaranthus hybridus INDIANA PI603895-2 MIRL	2.29	147656862
7 (++)	Amaranthus hybridus INDIANA PI603895-3 MIRL	2.17	147656862
8 (++)	Amaranthus hypochondriacus MEXICO SONORA PI599682-2 MIRL	2.14	147656862
9 (++)	Amaranthus hybridus INDIANA PI603895-1 MIRL	2	147656862
10 (+)	Amaranthus hypochondriacus MISSOURI PI698341-3 MIRL	1.99	147656862
11 (+)	Amaranthus hypochondriacus MEXICO SONORA PI599682-1 MIRL	1.98	147656862

12 (+)	Amaranthus hypochondriacus MEXICO SONORA PI599682-3 MIRL	1.95	147656862
13 (+)	Amaranthus caudatus NJ PI553073-1 MIRL	1.92	147656862
14 (+)	Amaranthus hypochondriacus MISSOURI PI698341-2 MIRL	1.92	147656862
15 (+)	Amaranthus hybridus HARROW 3 MIRL	1.8	147656862
16 (+)	Amaranthus hybridus HARROW 2 MIRL	1.8	147656862
17 (+)	Amaranthus hybridus PUERTO RICO AMES 5152-3 MIRL	1.8	147656862
18 (+)	Amaranthus hypochondriacus MISSOURI PI698341-1 MIRL	1.79	147656862
19 (+)	Amaranthus hybridus HARROW 1 MIRL	1.74	147656862
20 (+)	Amaranthus caudatus NJ AMES 5687-3 MIRL	1.71	147656862

Analyte119

Analyte Name:	Amaranthus caudatus NJ PI553073-3 MIRL
Analyte Description:	MSP
Analyte ID:	920905d3-6f83-49a7-aed3-d5420c389618
Analyte Creation Date/Time:	2023-04-25 1:40:10 PM
Applied MSP Library(ies):	
Applied Taxonomy Tree:	Bruker Taxonomy

Rank (Quality)	Matched Pattern	Score Value	NCBI Identifier
1 (+++)	Amaranthus caudatus NJ PI553073-3 MIRL	3	147656862
2 (+++)	Amaranthus caudatus NJ PI553073-2 MIRL	2.72	147656862
3 (+++)	Amaranthus caudatus CALIFORNIA PI690570-3 MIRL	2.68	147656862
4 (+++)	Amaranthus caudatus CALIFORNIA PI690570-1 MIRL	2.65	147656862
5 (+++)	Amaranthus caudatus CALIFORNIA PI690570-2 MIRL	2.6	147656862
6 (+++)	Amaranthus hybridus INDIANA PI603895-2 MIRL	2.31	147656862
7 (++)	Amaranthus hybridus INDIANA PI603895-3 MIRL	2.19	147656862
8 (++)	Amaranthus hypochondriacus MEXICO SONORA PI599682-2 MIRL	2.18	147656862
9 (++)	Amaranthus hypochondriacus MISSOURI PI698341-3 MIRL	2.15	147656862
10 (++)	Amaranthus hypochondriacus MISSOURI PI698341-2 MIRL	2.13	147656862
11 (++)	Amaranthus hypochondriacus MEXICO SONORA PI599682-1 MIRL	2.09	147656862

12<br>(++)	Amaranthus hybridus INDIANA PI603895-1 MIRL	2.07	147656862
13<br>(++)	Amaranthus hypochondriacus MISSOURI PI698341-1 MIRL	2.05	147656862
14<br>(++)	Amaranthus hypochondriacus MEXICO SONORA PI599682-3 MIRL	2.01	147656862
15<br>(+)	Amaranthus hybridus PUERTO RICO AMES 5152-2 MIRL	1.88	147656862
16<br>(+)	Amaranthus hybridus PUERTO RICO AMES 5152-1 MIRL	1.87	147656862
17<br>(+)	Amaranthus hybridus HARROW 3 MIRL	1.86	147656862
18<br>(+)	Amaranthus hybridus HARROW 1 MIRL	1.84	147656862
19<br>(+)	Amaranthus caudatus NJ PI553073-1 MIRL	1.83	147656862
20<br>(+)	Amaranthus hybridus HARROW 2 MIRL	1.82	147656862

Analyte120

Analyte Name:	Amaranthus caudatus CALIFORNIA PI690570-1 MIRL
Analyte Description:	MSP
Analyte ID:	758af967-651e-4991-b6ce-923efb61bed3
Analyte Creation Date/Time:	2023-04-25 1:46:28 PM
Applied MSP Library(ies):	
Applied Taxonomy Tree:	Bruker Taxonomy

Rank (Quality)	Matched Pattern	Score Value	NCBI Identifier
1 (+++)	Amaranthus caudatus CALIFORNIA PI690570-1 MIRL	3	147656862
2 (+++)	Amaranthus caudatus CALIFORNIA PI690570-3 MIRL	2.76	147656862
3 (+++)	Amaranthus caudatus CALIFORNIA PI690570-2 MIRL	2.76	147656862
4 (+++)	Amaranthus caudatus NJ PI553073-3 MIRL	2.65	147656862
5 (+++)	Amaranthus caudatus NJ PI553073-2 MIRL	2.59	147656862
6 (++)	Amaranthus hybridus INDIANA PI603895-2 MIRL	2.2	147656862
7 (++)	Amaranthus caudatus NJ AMES 5687-3 MIRL	2.16	147656862
8 (++)	Amaranthus hybridus INDIANA PI603895-3 MIRL	2.15	147656862
9 (++)	Amaranthus hybridus INDIANA PI603895-1 MIRL	2.12	147656862
10 (++)	Amaranthus hypochondriacus MEXICO SONORA PI599682-2 MIRL	2.08	147656862
11 (++)	Amaranthus caudatus NJ AMES 5687-2 MIRL	2.03	147656862

12 (+)	Amaranthus caudatus NJ PI553073-1 MIRL	1.98	147656862
13 (+)	Amaranthus hypochondriacus MISSOURI PI698341-2 MIRL	1.95	147656862
14 (+)	Amaranthus hypochondriacus MISSOURI PI698341-3 MIRL	1.91	147656862
15 (+)	Amaranthus hybridus HARROW 1 MIRL	1.9	147656862
16 (+)	Amaranthus hypochondriacus MISSOURI PI698341-1 MIRL	1.84	147656862
17 (+)	Amaranthus hypochondriacus MEXICO SONORA PI599682-3 MIRL	1.82	147656862
18 (+)	Amaranthus hypochondriacus MEXICO SONORA PI599682-1 MIRL	1.8	147656862
19 (+)	Amaranthus powellii sub. Powellii TEXAS PI632241-3 MIRL	1.76	147656862
20 (+)	Amaranthus hybridus HARROW 3 MIRL	1.74	147656862

Analyte121

Analyte Name:	Amaranthus caudatus CALIFORNIA PI690570-2 MIRL
Analyte Description:	MSP
Analyte ID:	38dce031-dffa-4825-909f-7e6e896f1f7b
Analyte Creation Date/Time:	2023-04-25 1:59:37 PM
Applied MSP Library(ies):	
Applied Taxonomy Tree:	Bruker Taxonomy

Rank (Quality)	Matched Pattern	Score Value	NCBI Identifier
1 (+++)	Amaranthus caudatus CALIFORNIA PI690570-2 MIRL	3	147656862
2 (+++)	Amaranthus caudatus CALIFORNIA PI690570-1 MIRL	2.76	147656862
3 (+++)	Amaranthus caudatus CALIFORNIA PI690570-3 MIRL	2.69	147656862
4 (+++)	Amaranthus caudatus NJ PI553073-3 MIRL	2.6	147656862
5 (+++)	Amaranthus caudatus NJ PI553073-2 MIRL	2.53	147656862
6 (++)	Amaranthus hybridus INDIANA PI603895-2 MIRL	2.2	147656862
7 (++)	Amaranthus hybridus INDIANA PI603895-3 MIRL	2.13	147656862
8 (++)	Amaranthus caudatus NJ AMES 5687-3 MIRL	2.08	147656862
9 (++)	Amaranthus hybridus INDIANA PI603895-1 MIRL	2.07	147656862
10 (++)	Amaranthus hypochondriacus MEXICO SONORA PI599682-2 MIRL	2.02	147656862
11 (+)	Amaranthus caudatus NJ AMES 5687-2 MIRL	1.98	147656862

12 (+)	Amaranthus caudatus NJ PI553073-1 MIRL	1.93	147656862
13 (+)	Amaranthus hypochondriacus MISSOURI PI698341-2 MIRL	1.92	147656862
14 (+)	Amaranthus hypochondriacus MISSOURI PI698341-1 MIRL	1.88	147656862
15 (+)	Amaranthus hybridus HARROW 1 MIRL	1.86	147656862
16 (+)	Amaranthus hypochondriacus MISSOURI PI698341-3 MIRL	1.84	147656862
17 (+)	Amaranthus hypochondriacus MEXICO SONORA PI599682-3 MIRL	1.84	147656862
18 (+)	Amaranthus powellii sub. Powellii TEXAS PI632241-3 MIRL	1.83	147656862
19 (+)	Amaranthus hypochondriacus MEXICO SONORA PI599682-1 MIRL	1.81	147656862
20 (+)	Amaranthus hybridus PUERTO RICO AMES 5152-1 MIRL	1.77	147656862

Analyte122

Analyte Name:	Amaranthus caudatus CALIFORNIA PI690570-3 MIRL
Analyte Description:	MSP
Analyte ID:	42f04a4d-d1f2-48cb-98ad-a11099b0f9bf
Analyte Creation Date/Time:	2023-04-25 2:05:29 PM
Applied MSP Library(ies):	
Applied Taxonomy Tree:	Bruker Taxonomy

Rank (Quality)	Matched Pattern	Score Value	NCBI Identifier
1 (+++)	Amaranthus caudatus CALIFORNIA PI690570-3 MIRL	3	147656862
2 (+++)	Amaranthus caudatus CALIFORNIA PI690570-1 MIRL	2.76	147656862
3 (+++)	Amaranthus caudatus CALIFORNIA PI690570-2 MIRL	2.69	147656862
4 (+++)	Amaranthus caudatus NJ PI553073-3 MIRL	2.67	147656862
5 (+++)	Amaranthus caudatus NJ PI553073-2 MIRL	2.66	147656862
6 (++)	Amaranthus hybridus INDIANA PI603895-2 MIRL	2.28	147656862
7 (++)	Amaranthus hypochondriacus MEXICO SONORA PI599682-2 MIRL	2.11	147656862
8 (++)	Amaranthus hybridus INDIANA PI603895-3 MIRL	2.07	147656862
9 (++)	Amaranthus hypochondriacus MISSOURI PI698341-3 MIRL	2.04	147656862
10 (++)	Amaranthus hypochondriacus MISSOURI PI698341-2 MIRL	2.03	147656862
11 (++)	Amaranthus caudatus NJ AMES 5687-3 MIRL	2.01	147656862

12 (+)	Amaranthus hypochondriacus MEXICO SONORA PI599682-1 MIRL	1.99	147656862
13 (+)	Amaranthus hybridus INDIANA PI603895-1 MIRL	1.97	147656862
14 (+)	Amaranthus powellii sub. Powellii TEXAS PI632241-3 MIRL	1.95	147656862
15 (+)	Amaranthus hypochondriacus MISSOURI PI698341-1 MIRL	1.94	147656862
16 (+)	Amaranthus caudatus NJ PI553073-1 MIRL	1.93	147656862
17 (+)	Amaranthus hypochondriacus MEXICO SONORA PI599682-3 MIRL	1.93	147656862
18 (+)	Amaranthus caudatus NJ AMES 5687-2 MIRL	1.91	147656862
19 (+)	Amaranthus hybridus PUERTO RICO AMES 5152-1 MIRL	1.8	147656862
20 (+)	Amaranthus hybridus HARROW 1 MIRL	1.8	147656862

Analyte123

Analyte Name: Amaranthus hypochondriacus MISSOURI PI698341-1 MIRL
Analyte Description: MSP
Analyte ID: d1d96e8f-af05-4e9e-9dde-d06049165e9f
Analyte Creation Date/Time: 2023-04-25 2:23:46 PM
Applied MSP Library(ies):
Applied Taxonomy Tree: Bruker Taxonomy

Rank (Quality)	Matched Pattern	Score Value	NCBI Identifier
1 (+++)	Amaranthus hypochondriacus MISSOURI PI698341-1 MIRL	3	147656862
2 (+++)	Amaranthus hypochondriacus MISSOURI PI698341-3 MIRL	2.79	147656862
3 (+++)	Amaranthus hypochondriacus MISSOURI PI698341-2 MIRL	2.76	147656862
4 (+++)	Amaranthus hypochondriacus PI658730-2 MIRL	2.72	147656862
5 (+++)	Amaranthus hypochondriacus PI658730-3 MIRL	2.63	147656862
6 (+++)	Amaranthus hypochondriacus PI658730-1 MIRL	2.62	147656862
7 (+++)	Amaranthus hypochondriacus MEXICO SONORA PI599682-1 MIRL	2.4	147656862
8 (+++)	Amaranthus hybridus PUERTO RICO AMES 5152-2 MIRL	2.39	147656862
9 (+++)	Amaranthus hybridus PUERTO RICO AMES 5152-3 MIRL	2.39	147656862
10 (+++)	Amaranthus hybridus PUERTO RICO AMES 5152-1 MIRL	2.34	147656862
11 (+++)	Amaranthus hybridus HARROW 3 MIRL	2.33	147656862

12 (+++)	Amaranthus hypochondriacus MEXICO SONORA PI599682-2 MIRL	2.32	147656862
13 (+++)	Amaranthus hypochondriacus MEXICO SONORA PI599682-3 MIRL	2.31	147656862
14 (+++)	Amaranthus hybridus HARROW 2 MIRL	2.31	147656862
15 (+++)	Amaranthus caudatus NJ AMES 5687-1 MIRL	2.3	147656862
16 (++)	Amaranthus caudatus NJ AMES 5687-2 MIRL	2.22	147656862
17 (++)	Amaranthus caudatus NJ AMES 5687-3 MIRL	2.14	147656862
18 (++)	Amaranthus hybridus HARROW 1 MIRL	2.13	147656862
19 (++)	Amaranthus hybridus INDIANA PI603895-2 MIRL	2.13	147656862
20 (++)	Amaranthus caudatus NJ PI553073-3 MIRL	2.05	147656862

Analyte124

Analyte Name:	Amaranthus hypochondriacus MISSOURI PI698341-2 MIRL
Analyte Description:	MSP
Analyte ID:	9f3ed725-14b6-46db-9073-c7a8df3e5e85
Analyte Creation Date/Time:	2023-04-25 2:29:42 PM
Applied MSP Library(ies):	
Applied Taxonomy Tree:	Bruker Taxonomy

Rank (Quality)	Matched Pattern	Score Value	NCBI Identifier
1 (+++)	Amaranthus hypochondriacus MISSOURI PI698341-2 MIRL	3	147656862
2 (+++)	Amaranthus hypochondriacus MISSOURI PI698341-3 MIRL	2.89	147656862
3 (+++)	Amaranthus hypochondriacus MISSOURI PI698341-1 MIRL	2.76	147656862
4 (+++)	Amaranthus hypochondriacus PI658730-2 MIRL	2.53	147656862
5 (+++)	Amaranthus hypochondriacus PI658730-1 MIRL	2.49	147656862
6 (+++)	Amaranthus hypochondriacus PI658730-3 MIRL	2.37	147656862
7 (+++)	Amaranthus hybridus PUERTO RICO AMES 5152-1 MIRL	2.32	147656862
8 (++)	Amaranthus hypochondriacus MEXICO SONORA PI599682-3 MIRL	2.29	147656862
9 (++)	Amaranthus hypochondriacus MEXICO SONORA PI599682-2 MIRL	2.28	147656862
10 (++)	Amaranthus hybridus PUERTO RICO AMES 5152-2 MIRL	2.25	147656862
11 (++)	Amaranthus hybridus INDIANA PI603895-2 MIRL	2.23	147656862

12<br>(++)	Amaranthus hypochondriacus MEXICO SONORA PI599682-1 MIRL	2.21	147656862
13<br>(++)	Amaranthus hybridus PUERTO RICO AMES 5152-3 MIRL	2.21	147656862
14<br>(++)	Amaranthus caudatus NJ PI553073-3 MIRL	2.14	147656862
15<br>(++)	Amaranthus hybridus HARROW 3 MIRL	2.13	147656862
16<br>(++)	Amaranthus hybridus HARROW 1 MIRL	2.1	147656862
17<br>(++)	Amaranthus hybridus HARROW 2 MIRL	2.07	147656862
18<br>(++)	Amaranthus caudatus NJ AMES 5687-1 MIRL	2.06	147656862
19<br>(++)	Amaranthus caudatus CALIFORNIA PI690570-3 MIRL	2.03	147656862
20<br>(+)	Amaranthus caudatus NJ AMES 5687-2 MIRL	1.99	147656862

Analyte125

Analyte Name:	Amaranthus hypochondriacus MISSOURI PI698341-3 MIRL
Analyte Description:	MSP
Analyte ID:	0b2972ec-6fb8-4e49-a85d-73a89680c745
Analyte Creation Date/Time:	2023-04-25 2:34:59 PM
Applied MSP Library(ies):	
Applied Taxonomy Tree:	Bruker Taxonomy

Rank (Quality)	Matched Pattern	Score Value	NCBI Identifier
1 (+++)	Amaranthus hypochondriacus MISSOURI PI698341-3 MIRL	3	147656862
2 (+++)	Amaranthus hypochondriacus MISSOURI PI698341-2 MIRL	2.89	147656862
3 (+++)	Amaranthus hypochondriacus MISSOURI PI698341-1 MIRL	2.79	147656862
4 (+++)	Amaranthus hypochondriacus PI658730-2 MIRL	2.57	147656862
5 (+++)	Amaranthus hypochondriacus PI658730-1 MIRL	2.54	147656862
6 (+++)	Amaranthus hypochondriacus PI658730-3 MIRL	2.41	147656862
7 (+++)	Amaranthus hybridus PUERTO RICO AMES 5152-1 MIRL	2.37	147656862
8 (+++)	Amaranthus hybridus PUERTO RICO AMES 5152-2 MIRL	2.3	147656862
9 (++)	Amaranthus hypochondriacus MEXICO SONORA PI599682-2 MIRL	2.29	147656862
10 (++)	Amaranthus hypochondriacus MEXICO SONORA PI599682-3 MIRL	2.25	147656862
11 (++)	Amaranthus hybridus PUERTO RICO AMES 5152-3 MIRL	2.24	147656862

12<br>(++)	Amaranthus hypochondriacus MEXICO SONORA PI599682-1 MIRL	2.23	147656862
13<br>(++)	Amaranthus hybridus HARROW 2 MIRL	2.19	147656862
14<br>(++)	Amaranthus caudatus NJ PI553073-3 MIRL	2.16	147656862
15<br>(++)	Amaranthus hybridus HARROW 3 MIRL	2.16	147656862
16<br>(++)	Amaranthus hybridus INDIANA PI603895-2 MIRL	2.15	147656862
17<br>(++)	Amaranthus caudatus NJ AMES 5687-1 MIRL	2.14	147656862
18<br>(++)	Amaranthus caudatus NJ AMES 5687-2 MIRL	2.06	147656862
19<br>(++)	Amaranthus hybridus HARROW 1 MIRL	2.05	147656862
20<br>(++)	Amaranthus caudatus CALIFORNIA PI690570-3 MIRL	2.04	147656862

Analyte126

Analyte Name:	Amaranthus hypochondriacus MEXICO SONORA PI599682-1 MIRL
Analyte Description:	MSP
Analyte ID:	2ba5fe12-5638-46d7-a65b-bdeee2c7e265
Analyte Creation Date/Time:	2023-04-28 1:57:19 PM
Applied MSP Library(ies):	
Applied Taxonomy Tree:	Bruker Taxonomy

Rank (Quality)	Matched Pattern	Score Value	NCBI Identifier
1 (+++)	Amaranthus hypochondriacus MEXICO SONORA PI599682-1 MIRL	3	147656862
2 (+++)	Amaranthus hypochondriacus MEXICO SONORA PI599682-2 MIRL	2.67	147656862
3 (+++)	Amaranthus hypochondriacus MEXICO SONORA PI599682-3 MIRL	2.66	147656862
4 (+++)	Amaranthus hybridus PUERTO RICO AMES 5152-2 MIRL	2.63	147656862
5 (+++)	Amaranthus hybridus PUERTO RICO AMES 5152-1 MIRL	2.61	147656862
6 (+++)	Amaranthus hybridus PUERTO RICO AMES 5152-3 MIRL	2.61	147656862
7 (+++)	Amaranthus hypochondriacus MISSOURI PI698341-1 MIRL	2.4	147656862
8 (+++)	Amaranthus caudatus NJ AMES 5687-1 MIRL	2.37	147656862
9 (+++)	Amaranthus hypochondriacus PI658730-2 MIRL	2.36	147656862
10 (+++)	Amaranthus hypochondriacus PI658730-1 MIRL	2.34	147656862
	Amaranthus hybridus HARROW 3 MIRL	2.3	147656862

11<br>(+++)			
12<br>(++)	Amaranthus caudatus NJ AMES 5687-2 MIRL	2.28	147656862
13<br>(++)	Amaranthus hybridus HARROW 2 MIRL	2.26	147656862
14<br>(++)	Amaranthus hypochondriacus MISSOURI PI698341-3 MIRL	2.23	147656862
15<br>(++)	Amaranthus hypochondriacus MISSOURI PI698341-2 MIRL	2.2	147656862
16<br>(++)	Amaranthus caudatus NJ AMES 5687-3 MIRL	2.2	147656862
17<br>(++)	Amaranthus hypochondriacus PI658730-3 MIRL	2.19	147656862
18<br>(++)	Amaranthus hybridus HARROW 1 MIRL	2.18	147656862
19<br>(++)	Amaranthus hybridus INDIANA PI603895-2 MIRL	2.11	147656862
20<br>(++)	Amaranthus caudatus NJ PI553073-3 MIRL	2.09	147656862

Analyte127

Analyte Name:	Amaranthus hypochondriacus MEXICO SONORA PI599682-2 MIRL
Analyte Description:	MSP
Analyte ID:	d41e3403-2c82-48d4-a9b4-b4a7c8bde248
Analyte Creation Date/Time:	2023-04-28 2:04:33 PM
Applied MSP Library(ies):	
Applied Taxonomy Tree:	Bruker Taxonomy

Rank (Quality)	Matched Pattern	Score Value	NCBI Identifier
1 (+++)	Amaranthus hypochondriacus MEXICO SONORA PI599682-2 MIRL	3	147656862
2 (+++)	Amaranthus hypochondriacus MEXICO SONORA PI599682-3 MIRL	2.72	147656862
3 (+++)	Amaranthus hypochondriacus MEXICO SONORA PI599682-1 MIRL	2.67	147656862
4 (+++)	Amaranthus hybridus PUERTO RICO AMES 5152-3 MIRL	2.46	147656862
5 (+++)	Amaranthus hybridus PUERTO RICO AMES 5152-2 MIRL	2.43	147656862
6 (+++)	Amaranthus hybridus PUERTO RICO AMES 5152-1 MIRL	2.42	147656862
7 (+++)	Amaranthus hypochondriacus MISSOURI PI698341-1 MIRL	2.32	147656862
8 (++)	Amaranthus hypochondriacus MISSOURI PI698341-3 MIRL	2.29	147656862
9 (++)	Amaranthus hypochondriacus MISSOURI PI698341-2 MIRL	2.28	147656862
10 (++)	Amaranthus hypochondriacus PI658730-2 MIRL	2.26	147656862
	Amaranthus hybridus HARROW 3 MIRL	2.24	147656862

11<br>(++)			
12<br>(++)	Amaranthus hybridus INDIANA PI603895-2 MIRL	2.23	147656862
13<br>(++)	Amaranthus hybridus HARROW 2 MIRL	2.22	147656862
14<br>(++)	Amaranthus caudatus NJ AMES 5687-2 MIRL	2.21	147656862
15<br>(++)	Amaranthus caudatus NJ AMES 5687-3 MIRL	2.21	147656862
16<br>(++)	Amaranthus caudatus NJ PI553073-3 MIRL	2.18	147656862
17<br>(++)	Amaranthus hybridus HARROW 1 MIRL	2.16	147656862
18<br>(++)	Amaranthus caudatus NJ AMES 5687-1 MIRL	2.16	147656862
19<br>(++)	Amaranthus caudatus NJ PI553073-2 MIRL	2.15	147656862
20<br>(++)	Amaranthus caudatus CALIFORNIA PI690570-3 MIRL	2.11	147656862

Analyte128

Analyte Name:	Amaranthus hypochondriacus MEXICO SONORA PI599682-3 MIRL
Analyte Description:	MSP
Analyte ID:	cdf28beb-c102-4e47-a8e8-2065dd0c04ca
Analyte Creation Date/Time:	2023-04-28 2:11:27 PM
Applied MSP Library(ies):	
Applied Taxonomy Tree:	Bruker Taxonomy

Rank (Quality)	Matched Pattern	Score Value	NCBI Identifier
1 (+++)	Amaranthus hypochondriacus MEXICO SONORA PI599682-3 MIRL	3	147656862
2 (+++)	Amaranthus hypochondriacus MEXICO SONORA PI599682-2 MIRL	2.71	147656862
3 (+++)	Amaranthus hypochondriacus MEXICO SONORA PI599682-1 MIRL	2.66	147656862
4 (+++)	Amaranthus hybridus PUERTO RICO AMES 5152-1 MIRL	2.53	147656862
5 (+++)	Amaranthus hybridus PUERTO RICO AMES 5152-2 MIRL	2.46	147656862
6 (+++)	Amaranthus hypochondriacus MISSOURI PI698341-1 MIRL	2.32	147656862
7 (+++)	Amaranthus hybridus INDIANA PI603895-2 MIRL	2.3	147656862
8 (++)	Amaranthus hypochondriacus MISSOURI PI698341-2 MIRL	2.29	147656862
9 (++)	Amaranthus hybridus PUERTO RICO AMES 5152-3 MIRL	2.28	147656862
10 (++)	Amaranthus hypochondriacus MISSOURI PI698341-3 MIRL	2.26	147656862
	Amaranthus hypochondriacus PI658730-2 MIRL	2.18	147656862

11 (++)			
12 (++)	Amaranthus hybridus HARROW 2 MIRL	2.12	147656862
13 (++)	Amaranthus hybridus HARROW 3 MIRL	2.12	147656862
14 (++)	Amaranthus hybridus HARROW 1 MIRL	2.07	147656862
15 (++)	Amaranthus hypochondriacus PI658730-1 MIRL	2.04	147656862
16 (++)	Amaranthus caudatus NJ AMES 5687-1 MIRL	2.04	147656862
17 (++)	Amaranthus caudatus NJ PI553073-3 MIRL	2.02	147656862
18 (+)	Amaranthus caudatus NJ PI553073-2 MIRL	1.96	147656862
19 (+)	Amaranthus hybridus INDIANA PI603895-3 MIRL	1.94	147656862
20 (+)	Amaranthus caudatus CALIFORNIA PI690570-3 MIRL	1.93	147656862

Analyte129

Analyte Name: Amaranthus albus WASHINGTON PI654389-1 MIRL
Analyte Description: MSP
Analyte ID: f5a1ad24-7719-4e0f-bd1c-fa434f1cbf45
Analyte Creation Date/Time: 2023-04-28 2:22:19 PM
Applied MSP Library(ies):
Applied Taxonomy Tree: Bruker Taxonomy

Rank (Quality)	Matched Pattern	Score Value	NCBI Identifier
1 (+++)	Amaranthus albus WASHINGTON PI654389-1 MIRL	3	147656862
2 (+++)	Amaranthus albus WASHINGTON PI654389-3 MIRL	2.67	147656862
3 (+++)	Amaranthus albus HARROW 2 MIRL	2.37	147656862
4 (+++)	Amaranthus albus HARROW 1 MIRL	2.34	147656862
5 (++)	Amaranthus albus WASHINGTON PI654389-2 MIRL	2.28	147656862
6 (++)	Amaranthus albus NC PI632244-2 MIRL	2.28	147656862
7 (++)	Amaranthus albus HARROW 3 MIRL	2.21	147656862
8 (++)	Amaranthus albus NC PI632244-3 MIRL	2.2	147656862
9 (++)	Amaranthus albus NC PI632244-1 MIRL	2.18	147656862
10 (++)	Amaranthus albus PI633580-3 MIRL	2	147656862
11 (+)	Amaranthus albus PI633580-2 MIRL	1.89	147656862

12<br>(+)	Amaranthus albus PI633580-1 MIRL	1.75	147656862
13<br>(-)	Amaranthus blitoides HARROW 2 MIRL	1.55	147656862
14<br>(-)	Amaranthus blitoides CANADA PI608663-3 MIRL	1.5	147656862
15<br>(-)	Amaranthus hybridus INDIANA PI603895-1 MIRL	1.49	147656862
16<br>(-)	Amaranthus blitoides HARROW 3 MIRL	1.49	147656862
17<br>(-)	Amaranthus californicus CALIFORNIA PI595319-2 MIRL	1.49	147656862
18<br>(-)	Amaranthus rudis HARROW 2 MIRL	1.39	147656862
19<br>(-)	Amaranthus blitoides CANADA PI608663-1 MIRL	1.37	147656862
20<br>(-)	Amaranthus hybridus INDIANA PI603895-2 MIRL	1.36	147656862

Analyte130

Analyte Name:	Amaranthus albus WASHINGTON PI654389-2 MIRL
Analyte Description:	MSP
Analyte ID:	36ec8fb2-94d3-4e90-96a2-f19c1b17b4e7
Analyte Creation Date/Time:	2023-04-28 2:30:25 PM
Applied MSP Library(ies):	
Applied Taxonomy Tree:	Bruker Taxonomy

Rank (Quality)	Matched Pattern	Score Value	NCBI Identifier
1 (+++)	Amaranthus albus WASHINGTON PI654389-2 MIRL	3	147656862
2 (+++)	Amaranthus albus WASHINGTON PI654389-3 MIRL	2.6	147656862
3 (+++)	Amaranthus albus HARROW 3 MIRL	2.45	147656862
4 (++)	Amaranthus albus WASHINGTON PI654389-1 MIRL	2.28	147656862
5 (++)	Amaranthus albus PI633580-2 MIRL	2.23	147656862
6 (++)	Amaranthus albus HARROW 1 MIRL	2.18	147656862
7 (++)	Amaranthus albus HARROW 2 MIRL	2.12	147656862
8 (++)	Amaranthus albus NC PI632244-3 MIRL	2.06	147656862
9 (++)	Amaranthus albus NC PI632244-2 MIRL	2.06	147656862
10 (++)	Amaranthus albus NC PI632244-1 MIRL	2	147656862
11 (+)	Amaranthus californicus CALIFORNIA PI595319-1 MIRL	1.87	147656862

12<br>(+)	Amaranthus albus PI633580-3 MIRL	1.71	147656862
13<br>(-)	Amaranthus blitoides CANADA PI608663-3 MIRL	1.45	147656862
14<br>(-)	Amaranthus hybridus INDIANA PI603895-1 MIRL	1.45	147656862
15<br>(-)	Amaranthus caudatus NJ PI553073-1 MIRL	1.45	147656862
16<br>(-)	Amaranthus californicus CALIFORNIA PI595319-2 MIRL	1.44	147656862
17<br>(-)	Amaranthus blitoides HARROW 3 MIRL	1.35	147656862
18<br>(-)	Amaranthus blitoides HARROW 2 MIRL	1.34	147656862
19<br>(-)	Amaranthus hypochondriacus MEXICO SONORA PI599682-3 MIRL	1.25	147656862
20<br>(-)	Amaranthus hybridus HARROW 1 MIRL	1.22	147656862

Analyte131

Analyte Name:	Amaranthus albus WASHINGTON PI654389-3 MIRL
Analyte Description:	MSP
Analyte ID:	3c34ff87-5bcf-46dc-b3a4-a86c03c95bff
Analyte Creation Date/Time:	2023-04-28 2:36:52 PM
Applied MSP Library(ies):	
Applied Taxonomy Tree:	Bruker Taxonomy

Rank (Quality)	Matched Pattern	Score Value	NCBI Identifier
1 (+++)	Amaranthus albus WASHINGTON PI654389-3 MIRL	3	147656862
2 (+++)	Amaranthus albus WASHINGTON PI654389-1 MIRL	2.67	147656862
3 (+++)	Amaranthus albus WASHINGTON PI654389-2 MIRL	2.6	147656862
4 (+++)	Amaranthus albus HARROW 3 MIRL	2.47	147656862
5 (+++)	Amaranthus albus HARROW 1 MIRL	2.45	147656862
6 (+++)	Amaranthus albus HARROW 2 MIRL	2.33	147656862
7 (+++)	Amaranthus albus NC PI632244-3 MIRL	2.31	147656862
8 (++)	Amaranthus albus NC PI632244-1 MIRL	2.25	147656862
9 (++)	Amaranthus albus NC PI632244-2 MIRL	2.21	147656862
10 (++)	Amaranthus albus PI633580-3 MIRL	2.09	147656862
11 (++)	Amaranthus albus PI633580-2 MIRL	2	147656862

12<br>(+)	Amaranthus albus PI633580-1 MIRL	1.86	147656862
13<br>(-)	Amaranthus blitoides HARROW 3 MIRL	1.68	147656862
14<br>(-)	Amaranthus californicus CALIFORNIA PI595319-1 MIRL	1.62	147656862
15<br>(-)	Amaranthus blitoides HARROW 2 MIRL	1.58	147656862
16<br>(-)	Amaranthus californicus CALIFORNIA PI595319-2 MIRL	1.5	147656862
17<br>(-)	Amaranthus blitoides CANADA PI608663-3 MIRL	1.47	147656862
18<br>(-)	Amaranthus hybridus INDIANA PI603895-1 MIRL	1.45	147656862
19<br>(-)	Amaranthus hypochondriacus MEXICO SONORA PI599682-1 MIRL	1.38	147656862
20<br>(-)	Amaranthus hypochondriacus MEXICO SONORA PI599682-3 MIRL	1.38	147656862

Analyte132

Analyte Name:	Amaranthus albus NC PI632244-1 MIRL
Analyte Description:	MSP
Analyte ID:	31afa189-5bd0-442f-b840-a9f3a74cf1be
Analyte Creation Date/Time:	2023-04-28 3:00:33 PM
Applied MSP Library(ies):	
Applied Taxonomy Tree:	Bruker Taxonomy

Rank (Quality)	Matched Pattern	Score Value	NCBI Identifier
1 (+++)	Amaranthus albus NC PI632244-1 MIRL	3	147656862
2 (+++)	Amaranthus albus NC PI632244-2 MIRL	2.66	147656862
3 (+++)	Amaranthus albus NC PI632244-3 MIRL	2.52	147656862
4 (+++)	Amaranthus albus PI633580-3 MIRL	2.42	147656862
5 (+++)	Amaranthus albus HARROW 1 MIRL	2.42	147656862
6 (++)	Amaranthus albus HARROW 2 MIRL	2.28	147656862
7 (++)	Amaranthus albus WASHINGTON PI654389-3 MIRL	2.25	147656862
8 (++)	Amaranthus albus PI633580-1 MIRL	2.23	147656862
9 (++)	Amaranthus albus HARROW 3 MIRL	2.2	147656862
10 (++)	Amaranthus albus WASHINGTON PI654389-1 MIRL	2.17	147656862
11 (+)	Amaranthus albus WASHINGTON PI654389-2 MIRL	1.99	147656862

12<br>(-)	Amaranthus albus PI633580-2 MIRL	1.69	147656862
13<br>(-)	Amaranthus blitoides CANADA PI608663-3 MIRL	1.66	147656862
14<br>(-)	Amaranthus blitoides HARROW 3 MIRL	1.54	147656862
15<br>(-)	Amaranthus blitoides HARROW 2 MIRL	1.54	147656862
16<br>(-)	Amaranthus blitoides CANADA PI608663-1 MIRL	1.49	147656862
17<br>(-)	Amaranthus blitoides CANADA PI608663-2 MIRL	1.38	147656862
18<br>(-)	Amaranthus californicus CALIFORNIA PI595319-2 MIRL	1.35	147656862
19<br>(-)	Amaranthus hybridus INDIANA PI603895-2 MIRL	1.27	147656862
20<br>(-)	Amaranthus tuberculatus KANSAS PI 60743-2 MIRL	1.21	147656862

Analyte133

Analyte Name:	Amaranthus albus NC PI632244-2 MIRL
Analyte Description:	MSP
Analyte ID:	dd54ca13-7acd-4b86-a4d5-034047decf81
Analyte Creation Date/Time:	2023-04-28 3:07:29 PM
Applied MSP Library(ies):	
Applied Taxonomy Tree:	Bruker Taxonomy

Rank (Quality)	Matched Pattern	Score Value	NCBI Identifier
1 (+++)	Amaranthus albus NC PI632244-2 MIRL	3	147656862
2 (+++)	Amaranthus albus NC PI632244-1 MIRL	2.66	147656862
3 (+++)	Amaranthus albus NC PI632244-3 MIRL	2.61	147656862
4 (+++)	Amaranthus albus PI633580-3 MIRL	2.3	147656862
5 (++)	Amaranthus albus WASHINGTON PI654389-1 MIRL	2.27	147656862
6 (++)	Amaranthus albus HARROW 1 MIRL	2.26	147656862
7 (++)	Amaranthus albus WASHINGTON PI654389-3 MIRL	2.2	147656862
8 (++)	Amaranthus albus HARROW 2 MIRL	2.19	147656862
9 (++)	Amaranthus albus PI633580-1 MIRL	2.16	147656862
10 (++)	Amaranthus albus HARROW 3 MIRL	2.14	147656862
11 (++)	Amaranthus albus WASHINGTON PI654389-2 MIRL	2.04	147656862

12<br>(+)	Amaranthus albus PI633580-2 MIRL	1.82	147656862
13<br>(-)	Amaranthus blitoides HARROW 3 MIRL	1.54	147656862
14<br>(-)	Amaranthus blitoides CANADA PI608663-3 MIRL	1.52	147656862
15<br>(-)	Amaranthus californicus CALIFORNIA PI595319-2 MIRL	1.48	147656862
16<br>(-)	Amaranthus blitoides HARROW 2 MIRL	1.44	147656862
17<br>(-)	Amaranthus tuberculatus KANSAS PI 60743-2 MIRL	1.42	147656862
18<br>(-)	Amaranthus californicus CALIFORNIA PI595319-1 MIRL	1.42	147656862
19<br>(-)	Amaranthus blitoides CANADA PI608663-1 MIRL	1.36	147656862
20<br>(-)	Amaranthus hybridus INDIANA PI603895-2 MIRL	1.3	147656862

Analyte134

Analyte Name: Amaranthus albus NC PI632244-3 MIRL
Analyte Description: MSP
Analyte ID: c10d131a-cb84-4cd9-aa77-0e41214c667b
Analyte Creation Date/Time: 2023-04-28 3:13:29 PM
Applied MSP Library(ies):
Applied Taxonomy Tree: Bruker Taxonomy

Rank (Quality)	Matched Pattern	Score Value	NCBI Identifier
1 (+++)	Amaranthus albus NC PI632244-3 MIRL	3	147656862
2 (+++)	Amaranthus albus NC PI632244-2 MIRL	2.61	147656862
3 (+++)	Amaranthus albus NC PI632244-1 MIRL	2.52	147656862
4 (+++)	Amaranthus albus HARROW 1 MIRL	2.35	147656862
5 (++)	Amaranthus albus WASHINGTON PI654389-3 MIRL	2.29	147656862
6 (++)	Amaranthus albus HARROW 2 MIRL	2.27	147656862
7 (++)	Amaranthus albus WASHINGTON PI654389-1 MIRL	2.18	147656862
8 (++)	Amaranthus albus PI633580-3 MIRL	2.09	147656862
9 (++)	Amaranthus albus WASHINGTON PI654389-2 MIRL	2.04	147656862
10 (+)	Amaranthus albus PI633580-1 MIRL	1.92	147656862
11 (+)	Amaranthus albus HARROW 3 MIRL	1.89	147656862

12<br>(-)	Amaranthus californicus CALIFORNIA PI595319-2 MIRL	1.53	147656862
13<br>(-)	Amaranthus blitoides HARROW 3 MIRL	1.52	147656862
14<br>(-)	Amaranthus albus PI633580-2 MIRL	1.48	147656862
15<br>(-)	Amaranthus californicus CALIFORNIA PI595319-1 MIRL	1.43	147656862
16<br>(-)	Amaranthus blitoides HARROW 2 MIRL	1.42	147656862
17<br>(-)	Amaranthus caudatus CALIFORNIA PI690570-3 MIRL	1.41	147656862
18<br>(-)	Amaranthus blitoides CANADA PI608663-2 MIRL	1.38	147656862
19<br>(-)	Amaranthus blitoides CANADA PI608663-1 MIRL	1.33	147656862
20<br>(-)	Amaranthus blitoides CANADA PI608663-3 MIRL	1.3	147656862

Analyte135

Analyte Name:	Amaranthus arenicola KANSAS PI599671-1 MIRL
Analyte Description:	MSP
Analyte ID:	9759bf2d-7a88-4247-a761-3555ded4b9da
Analyte Creation Date/Time:	2023-05-10 2:43:44 PM
Applied MSP Library(ies):	
Applied Taxonomy Tree:	Bruker Taxonomy

Rank (Quality)	Matched Pattern	Score Value	NCBI Identifier
1 (+++)	Amaranthus arenicola KANSAS PI599671-1 MIRL	3	147656862
2 (+)	Amaranthus arenicola KANSAS PI599671-2 MIRL	1.86	147656862
3 (+)	Amaranthus arenicola KANSAS PI599671-3 MIRL	1.83	147656862
4 (-)	Amaranthus arenicola PI 599670-1 MIRL	1.65	147656862
5 (-)	Amaranthus arenicola PI 599670-3 MIRL	1.52	147656862
6 (-)	Amaranthus tuberculatus IOWA PI674264-3 MIRL	1.49	147656862
7 (-)	Amaranthus tuberculatus IOWA PI674264-1 MIRL	1.4	147656862
8 (-)	Amaranthus tuberculatus IOWA PI674264-2 MIRL	1.31	147656862
9 (-)	Amaranthus arenicola TEXAS PI667168-2 MIRL	1.3	147656862
10 (-)	Amaranthus tuberculatus IOWA PI 553086-3 MIRL	1.3	147656862
11 (-)	Amaranthus rudis HARROW 3 MIRL	1.25	147656862

12<br>(-)	Amaranthus watsonii PI 633593-2 MIRL	1.24	147656862
13<br>(-)	Amaranthus rudis HARROW 1 MIRL	1.22	147656862
14<br>(-)	Amaranthus tuberculatus KANSAS PI 60743-3 MIRL	1.22	147656862
15<br>(-)	Amaranthus spinosus HARROW 3 MIRL	1.2	147656862
16<br>(-)	Amaranthus watsonii MEXICO COLIMA PI633593-RE2 MIRL	1.19	147656862
17<br>(-)	Amaranthus palmeri MEXICO PUEBLA PI604557-2 MIRL	1.15	147656862
18<br>(-)	Amaranthus rudis HARROW 2 MIRL	1.13	147656862
19<br>(-)	Amaranthus tuberculatus IOWA PI604247-3 MIRL	1.11	147656862
20<br>(-)	Amaranthus arenicola TEXAS PI667168-3 MIRL	1.09	147656862

Analyte136

Analyte Name:	Amaranthus arenicola KANSAS PI599671-2 MIRL
Analyte Description:	MSP
Analyte ID:	410ba18a-e92b-4281-9fb2-eff22a7ab652
Analyte Creation Date/Time:	2023-05-10 2:49:15 PM
Applied MSP Library(ies):	
Applied Taxonomy Tree:	Bruker Taxonomy

Rank (Quality)	Matched Pattern	Score Value	NCBI Identifier
1 (+++)	Amaranthus arenicola KANSAS PI599671-2 MIRL	3	147656862
2 (++)	Amaranthus arenicola KANSAS PI599671-3 MIRL	2.28	147656862
3 (+)	Amaranthus arenicola PI 599670-1 MIRL	1.93	147656862
4 (+)	Amaranthus arenicola KANSAS PI599671-1 MIRL	1.82	147656862
5 (-)	Amaranthus arenicola PI 599670-3 MIRL	1.65	147656862
6 (-)	Amaranthus arenicola PI 599670-2 MIRL	1.53	147656862
7 (-)	Amaranthus spinosus HARROW 1 MIRL	1.32	147656862
8 (-)	Amaranthus watsonii MEXICO COLIMA PI633593-RE1 MIRL	1.24	147656862
9 (-)	Amaranthus spinosus HARROW 3 MIRL	1.18	147656862
10 (-)	Amaranthus rudis HARROW 3 MIRL	1.14	147656862
11 (-)	Amaranthus watsonii PI 633593-2 MIRL	1.12	147656862

12<br>(-)	Amaranthus retroflexus CANADA AMES 5328-2 MIRL	1.12	147656862
13<br>(-)	Amaranthus palmeri MEXICO PUEBLA PI604557-2 MIRL	1.1	147656862
14<br>(-)	Amaranthus rudis HARROW 2 MIRL	1.1	147656862
15<br>(-)	Amaranthus retroflexus CANADA AMES 5328-1 MIRL	1.08	147656862
16<br>(-)	Amaranthus spinosus HARROW 2 MIRL	1.06	147656862
17<br>(-)	Amaranthus palmeri MEXICO VERACRUZ PI667167-RE2 MIRL	1.04	147656862
18<br>(-)	Amaranthus palmeri MEXICO VERACRUZ PI667167-1 MIRL	1.03	147656862
19<br>(-)	Amaranthus watsonii MEXICO COLIMA PI633593-RE3 MIRL	1.01	147656862
20<br>(-)	Amaranthus arenicola TEXAS PI667168-2 MIRL	1.01	147656862

Analyte137

Analyte Name:	Amaranthus arenicola KANSAS PI599671-3 MIRL
Analyte Description:	MSP
Analyte ID:	2715ee21-2d02-4be4-b976-9f0430dde012
Analyte Creation Date/Time:	2023-05-10 2:56:45 PM
Applied MSP Library(ies):	
Applied Taxonomy Tree:	Bruker Taxonomy

Rank (Quality)	Matched Pattern	Score Value	NCBI Identifier
1 (+++)	Amaranthus arenicola KANSAS PI599671-3 MIRL	3	147656862
2 (++)	Amaranthus arenicola KANSAS PI599671-2 MIRL	2.27	147656862
3 (++)	Amaranthus arenicola PI 599670-1 MIRL	2.24	147656862
4 (+)	Amaranthus arenicola KANSAS PI599671-1 MIRL	1.83	147656862
5 (+)	Amaranthus arenicola PI 599670-3 MIRL	1.82	147656862
6 (-)	Amaranthus arenicola PI 599670-2 MIRL	1.69	147656862
7 (-)	Amaranthus palmeri MEXICO VERACRUZ PI667167-RE2 MIRL	1.41	147656862
8 (-)	Amaranthus arenicola TEXAS PI667168-2 MIRL	1.36	147656862
9 (-)	Amaranthus arenicola TEXAS PI667168-3 MIRL	1.26	147656862
10 (-)	Amaranthus powelli sub. powelli ME AMES 29205-3 MIRL	1.25	147656862
11 (-)	Amaranthus spinosus HARROW 3 MIRL	1.24	147656862

12<br>(-)	Amaranthus palmeri MEXICO PUEBLA PI604557-2 MIRL	1.23	147656862
13<br>(-)	Amaranthus californicus CALIFORNIA PI595319-1 MIRL	1.21	147656862
14<br>(-)	Amaranthus rudis HARROW 2 MIRL	1.21	147656862
15<br>(-)	Amaranthus rudis HARROW 3 MIRL	1.2	147656862
16<br>(-)	Amaranthus tuberculatus KANSAS PI 60743-3 MIRL	1.18	147656862
17<br>(-)	Amaranthus powelli sub. powelli ME AMES 29205-2 MIRL	1.16	147656862
18<br>(-)	Amaranthus palmeri MEXICO VERACRUZ PI667167-1 MIRL	1.15	147656862
19<br>(-)	Amaranthus palmeri MEXICO VERACRUZ PI667167-3 MIRL	1.14	147656862
20<br>(-)	Amaranthus palmeri DAKAR PI633587-2 MIRL	1.12	147656862

Analyte138

Analyte Name:	Amaranthus arenicola TEXAS PI667168-1 MIRL
Analyte Description:	MSP
Analyte ID:	2ec679bd-32ea-4f08-a3c8-b103cb6b79af
Analyte Creation Date/Time:	2023-05-10 3:03:38 PM
Applied MSP Library(ies):	
Applied Taxonomy Tree:	Bruker Taxonomy

Rank (Quality)	Matched Pattern	Score Value	NCBI Identifier
1 (+++)	Amaranthus arenicola TEXAS PI667168-1 MIRL	3	147656862
2 (++)	Amaranthus arenicola TEXAS PI667168-3 MIRL	2.25	147656862
3 (++)	Amaranthus rudis HARROW 2 MIRL	2.12	147656862
4 (++)	Amaranthus tuberculatus KANSAS PI 60743-3 MIRL	2.11	147656862
5 (++)	Amaranthus rudis HARROW 3 MIRL	2.07	147656862
6 (++)	Amaranthus arenicola TEXAS PI667168-2 MIRL	2.06	147656862
7 (+)	Amaranthus tuberculatus KANSAS PI 60743-2 MIRL	1.98	147656862
8 (+)	Amaranthus tuberculatus IOWA PI 553086-3 MIRL	1.97	147656862
9 (+)	Amaranthus tuberculatus IOWA PI 553086-1 MIRL	1.93	147656862
10 (+)	Amaranthus tuberculatus KANSAS PI 60743-1 MIRL	1.92	147656862
11 (+)	Amaranthus rudis HARROW 1 MIRL	1.88	147656862

12<br>(+)	Amaranthus tuberculatus IOWA PI604247-2 MIRL	1.76	147656862
13<br>(+)	Amaranthus tuberculatus IOWA PI604247-3 MIRL	1.75	147656862
14<br>(+)	Amaranthus tuberculatus IOWA PI674264-1 MIRL	1.71	147656862
15<br>(-)	Amaranthus tuberculatus IOWA PI674264-3 MIRL	1.64	147656862
16<br>(-)	Amaranthus tuberculatus IOWA PI 553086-2 MIRL	1.63	147656862
17<br>(-)	Amaranthus tuberculatus IOWA PI674264-2 MIRL	1.61	147656862
18<br>(-)	Amaranthus tuberculatus IOWA PI604247-1 MIRL	1.5	147656862
19<br>(-)	Amaranthus spinosus HARROW 2 MIRL	1.17	147656862
20<br>(-)	Amaranthus albus PI633580-2 MIRL	1.11	147656862

Analyte139

Analyte Name:	Amaranthus arenicola TEXAS PI667168-2 MIRL
Analyte Description:	MSP
Analyte ID:	a9418678-73fb-4ee4-b04d-e05078888dc4
Analyte Creation Date/Time:	2023-05-10 3:10:20 PM
Applied MSP Library(ies):	
Applied Taxonomy Tree:	Bruker Taxonomy

Rank (Quality)	Matched Pattern	Score Value	NCBI Identifier
1 (+++)	Amaranthus arenicola TEXAS PI667168-2 MIRL	3	147656862
2 (+++)	Amaranthus arenicola TEXAS PI667168-3 MIRL	2.48	147656862
3 (++)	Amaranthus tuberculatus KANSAS PI 60743-3 MIRL	2.09	147656862
4 (++)	Amaranthus arenicola TEXAS PI667168-1 MIRL	2.05	147656862
5 (++)	Amaranthus tuberculatus KANSAS PI 60743-1 MIRL	2.02	147656862
6 (+)	Amaranthus tuberculatus KANSAS PI 60743-2 MIRL	1.9	147656862
7 (+)	Amaranthus rudis HARROW 3 MIRL	1.84	147656862
8 (+)	Amaranthus tuberculatus IOWA PI 553086-3 MIRL	1.84	147656862
9 (+)	Amaranthus rudis HARROW 2 MIRL	1.78	147656862
10 (+)	Amaranthus tuberculatus IOWA PI674264-3 MIRL	1.72	147656862
11 (+)	Amaranthus tuberculatus IOWA PI604247-3 MIRL	1.7	147656862

12<br>**(-)**	Amaranthus rudis HARROW 1 MIRL	1.66	147656862
13<br>**(-)**	Amaranthus tuberculatus IOWA PI 553086-1 MIRL	1.57	147656862
14<br>**(-)**	Amaranthus tuberculatus IOWA PI 553086-2 MIRL	1.52	147656862
15<br>**(-)**	Amaranthus tuberculatus IOWA PI604247-2 MIRL	1.45	147656862
16<br>**(-)**	Amaranthus arenicola KANSAS PI599671-3 MIRL	1.35	147656862
17<br>**(-)**	Amaranthus tuberculatus IOWA PI604247-1 MIRL	1.34	147656862
18<br>**(-)**	Amaranthus arenicola KANSAS PI599671-1 MIRL	1.31	147656862
19<br>**(-)**	Amaranthus tuberculatus IOWA PI674264-1 MIRL	1.3	147656862
20<br>**(-)**	Amaranthus tuberculatus IOWA PI674264-2 MIRL	1.23	147656862

Analyte140

Analyte Name: Amaranthus arenicola TEXAS PI667168-3 MIRL
Analyte Description: MSP
Analyte ID: ccbd7fa4-4c73-4e5b-b2c2-2ad359554ed8
Analyte Creation Date/Time: 2023-05-10 3:16:29 PM
Applied MSP Library(ies):
Applied Taxonomy Tree: Bruker Taxonomy

Rank (Quality)	Matched Pattern	Score Value	NCBI Identifier
1 (+++)	Amaranthus arenicola TEXAS PI667168-3 MIRL	3	147656862
2 (+++)	Amaranthus arenicola TEXAS PI667168-2 MIRL	2.49	147656862
3 (++)	Amaranthus arenicola TEXAS PI667168-1 MIRL	2.24	147656862
4 (++)	Amaranthus tuberculatus KANSAS PI 60743-3 MIRL	2.15	147656862
5 (++)	Amaranthus rudis HARROW 2 MIRL	2.01	147656862
6 (+)	Amaranthus rudis HARROW 3 MIRL	1.97	147656862
7 (+)	Amaranthus tuberculatus KANSAS PI 60743-2 MIRL	1.94	147656862
8 (+)	Amaranthus tuberculatus IOWA PI 553086-3 MIRL	1.93	147656862
9 (+)	Amaranthus tuberculatus IOWA PI674264-3 MIRL	1.84	147656862
10 (+)	Amaranthus rudis HARROW 1 MIRL	1.83	147656862
11 (+)	Amaranthus tuberculatus IOWA PI604247-3 MIRL	1.77	147656862

12<br>(+)	Amaranthus tuberculatus IOWA PI 553086-1 MIRL	1.73	147656862
13<br>(+)	Amaranthus tuberculatus KANSAS PI 60743-1 MIRL	1.7	147656862
14<br>(-)	Amaranthus tuberculatus IOWA PI674264-2 MIRL	1.69	147656862
15<br>(-)	Amaranthus tuberculatus IOWA PI 553086-2 MIRL	1.57	147656862
16<br>(-)	Amaranthus tuberculatus IOWA PI604247-2 MIRL	1.54	147656862
17<br>(-)	Amaranthus tuberculatus IOWA PI674264-1 MIRL	1.5	147656862
18<br>(-)	Amaranthus tuberculatus IOWA PI604247-1 MIRL	1.37	147656862
19<br>(-)	Amaranthus arenicola KANSAS PI599671-3 MIRL	1.24	147656862
20<br>(-)	Amaranthus albus WASHINGTON PI654389-3 MIRL	1.19	147656862

Analyte141

Analyte Name:	Amaranthus californicus CALIFORNIA PI595319-1 MIRL
Analyte Description:	MSP
Analyte ID:	514f2053-38fd-43ad-9d6d-18b1c0c12cdf
Analyte Creation Date/Time:	2023-05-10 3:21:52 PM
Applied MSP Library(ies):	
Applied Taxonomy Tree:	Bruker Taxonomy

Rank (Quality)	Matched Pattern	Score Value	NCBI Identifier
1 (+++)	Amaranthus californicus CALIFORNIA PI595319-1 MIRL	3	147656862
2 (+++)	Amaranthus californicus CALIFORNIA PI595319-2 MIRL	2.54	147656862
3 (+)	Amaranthus albus WASHINGTON PI654389-2 MIRL	1.86	147656862
4 (-)	Amaranthus albus WASHINGTON PI654389-3 MIRL	1.62	147656862
5 (-)	Amaranthus albus HARROW 3 MIRL	1.54	147656862
6 (-)	Amaranthus albus HARROW 1 MIRL	1.53	147656862
7 (-)	Amaranthus albus NC PI632244-3 MIRL	1.46	147656862
8 (-)	Amaranthus albus NC PI632244-2 MIRL	1.43	147656862
9 (-)	Amaranthus albus PI633580-2 MIRL	1.4	147656862
10 (-)	Amaranthus albus HARROW 2 MIRL	1.37	147656862
11 (-)	Amaranthus albus WASHINGTON PI654389-1 MIRL	1.37	147656862

12<br>**(-)**	Amaranthus hybridus HARROW 1 MIRL	1.32	147656862
13<br>**(-)**	Amaranthus hypochondriacus PI658730-1 MIRL	1.31	147656862
14<br>**(-)**	Amaranthus caudatus NJ AMES 5687-2 MIRL	1.3	147656862
15<br>**(-)**	Amaranthus hybridus INDIANA PI603895-1 MIRL	1.28	147656862
16<br>**(-)**	Amaranthus hybridus INDIANA PI603895-3 MIRL	1.22	147656862
17<br>**(-)**	Amaranthus arenicola KANSAS PI599671-3 MIRL	1.22	147656862
18<br>**(-)**	Amaranthus blitoides CANADA PI608663-2 MIRL	1.22	147656862
19<br>**(-)**	Amaranthus hypochondriacus PI658730-2 MIRL	1.2	147656862
20<br>**(-)**	Amaranthus caudatus NJ AMES 5687-1 MIRL	1.16	147656862

Analyte142

Analyte Name:	Amaranthus californicus CALIFORNIA PI595319-2 MIRL
Analyte Description:	MSP
Analyte ID:	36ad6c87-ecf3-4640-8340-d991c492f3b9
Analyte Creation Date/Time:	2023-05-10 3:27:29 PM
Applied MSP Library(ies):	
Applied Taxonomy Tree:	Bruker Taxonomy

Rank (Quality)	Matched Pattern	Score Value	NCBI Identifier
1 (+++)	Amaranthus californicus CALIFORNIA PI595319-2 MIRL	3	147656862
2 (+++)	Amaranthus californicus CALIFORNIA PI595319-1 MIRL	2.54	147656862
3 (-)	Amaranthus albus NC PI632244-3 MIRL	1.55	147656862
4 (-)	Amaranthus albus WASHINGTON PI654389-3 MIRL	1.53	147656862
5 (-)	Amaranthus albus NC PI632244-2 MIRL	1.49	147656862
6 (-)	Amaranthus albus WASHINGTON PI654389-1 MIRL	1.49	147656862
7 (-)	Amaranthus albus WASHINGTON PI654389-2 MIRL	1.43	147656862
8 (-)	Amaranthus blitoides CANADA PI608663-2 MIRL	1.43	147656862
9 (-)	Amaranthus blitoides CANADA PI608663-3 MIRL	1.39	147656862
10 (-)	Amaranthus albus NC PI632244-1 MIRL	1.35	147656862
11 (-)	Amaranthus albus HARROW 1 MIRL	1.33	147656862

12<br>**(-)**	Amaranthus blitoides HARROW 3 MIRL	1.32	147656862
13<br>**(-)**	Amaranthus blitoides HARROW 2 MIRL	1.31	147656862
14<br>**(-)**	Amaranthus albus PI633580-3 MIRL	1.29	147656862
15<br>**(-)**	Amaranthus blitoides CANADA PI608663-1 MIRL	1.29	147656862
16<br>**(-)**	Amaranthus hybridus PUERTO RICO AMES 5152-3 MIRL	1.29	147656862
17<br>**(-)**	Amaranthus albus HARROW 3 MIRL	1.25	147656862
18<br>**(-)**	Amaranthus hybridus INDIANA PI603895-1 MIRL	1.24	147656862
19<br>**(-)**	Amaranthus caudatus NJ AMES 5687-2 MIRL	1.19	147656862
20<br>**(-)**	Amaranthus hybridus HARROW 3 MIRL	1.19	147656862
